# Supplementary material for: Paired electrocatalysis unlocks cross-dehydrogenative coupling of C(sp3)-H bonds using a pentacoordinated cobalt-salen catalyst
Source: Nat Commun. 2024 Apr 4;15:2897. doi: 10.1038/s41467-024-47220-9 (PMC10995126; doi:10.1038/s41467-024-47220-9)
Supplement: Supplementary file 1 — Supplementary Information [file 41467_2024_47220_MOESM1_ESM.pdf]

Paired Electrocatalysis Unlocks Cross-Dehydrogenative Coupling of C(sp^3)-H Bonds Using A Pentacoordinated Cobalt-Salen Catalyst

Ke Liu,[#] Mengna Lei,[#] Xin Li, Xuemei Zhang, Ying Zhang, Weigang Fan, Man-Bo Li* and Sheng Zhang*
Institutes of Physical Science and Information Technology, Key Laboratory of Structure and Functional Regulation of Hybrid Materials of Ministry of Education, Anhui University, Hefei, Anhui 230601, P. R. China.
Email: mbli@ahu.edu.cn; shengzhang@ahu.edu.cn.

Supplementary Information

Table of Contents

Part I Experimental Section S2

1. General information S2

2. Preparation and structure of pentacoordinated Co-salen catalysts S2

3. Cyclic voltammograms of Co-salen, H^-T catalysts and substrates S3-S15

4. GC analysis for the byproduct hydrogen S15

5. Optimization of reaction conditions S16-S17

6. General procedure for the electrochemical CDC reaction S18

7. Procedure for gram scale reaction and derivatization of products S19-S20

8. Procedure and details for control experiments S21-S23

9. Photophysical property investigation (**4**) S24

10. Experimental data S25-S46

Part II NMR spectra S47-S134

References S135

1. General Information

^1H NMR and ^{13}C NMR were recorded on a Bruker 400 MHz spectrometer (^1H NMR: 400 MHz, ^{13}C NMR: 101 MHz). The chemical shifts (δ) and coupling constants (*J*) were expressed in ppm and Hz respectively. ^1H NMR spectra were referenced to the solvent residual peak (TMS, δ 0 ppm) and $^{13}C\{1H\}$ NMR spectra were referenced to the solvent residual peak ($CDCl_3$, δ 77.0 ppm; CD_3CN, δ 118.0 ppm). High Resolution mass spectra were obtained using ThermoFisher LTQ Orbitrap XL mass spectrometer. Cyclic voltammograms were recorded on electrochemical workstation CHI660E (Shanghai CH Instruments Co., Ltd.). All UV-vis absorption measurements were performed on a SPECORD 210 PLUS spectrophotometer. The single crystal X-ray diffraction (SCXRD) data was obtained by using a Stoe Stadivari diffractometer. The structures were solved and refined using the SHELXT software. GC analysis was conducted using CEAULIGHT GC-7920 spectrometer. All solvents were purified and dried according to the standard procedures unless otherwise noted. Commercially substrates were purchased and used directly. Allylbenzenes[1], penta-1,4-dien-1-ylbenzene **2m**[2], Adapalene derived alcohol[3], **1a-d$_1$**[3], salen-cobalt (**cat 4-cat 8**)[4], and **2ai-d$_2$**[5], were prepared according to the literature procedures.

2. Preparation and structure of pentacoordinated Co-salen catalysts

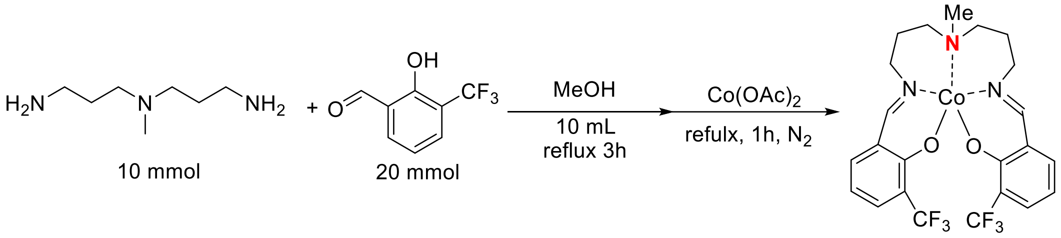

cat 1

To the solution of *N^1*-(3-aminopropyl)-*N^1*-methylpropane-1,3-diamine (1.61 mL, 10 mmol) in methanol (10 mL), 2-hydroxy-3-(trifluoromethyl)benzaldehyde (3.8 g, 20 mmol) was added. The resulting mixture was refluxed for 3 hours and the solution turned to yellow. $Co(OAc)_2$ (1.77 g) was further added to the solution under nitrogen atmosphere. After refluxing for 1 hour, an earthy yellow precipitate was collected and washed with cooled methanol to give the pure product (**cat 1**) in 58% yield (3.17 g). The single crystal can be accessed via evaporating the solution of **cat 1** in mixed ethanol and ethyl ether (8/2, v/v).

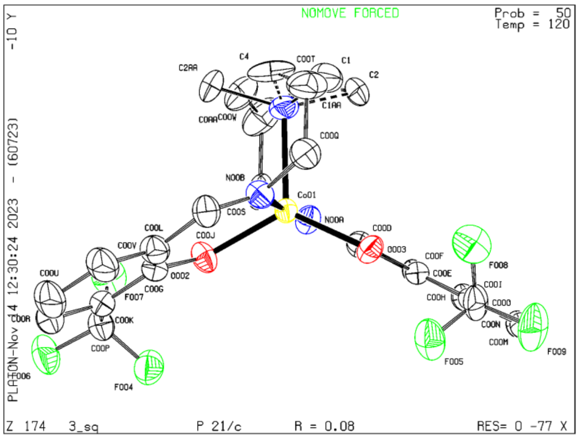

Fig. 1 Single crystal structure of **cat 1** (CCDC NO 2313154)

3. Cyclic voltammograms of Co-salen, H$^-$T catalysts and substrates

The electrochemical analysis was demonstrated with Ag wire as a reference electrode, which is not a stable reference electrode. CVs can be calibrated using ferrocene as an external reference. (Fig.S2) $E_{p/2}$ = (0.095-0.015)/2 = 0.04V.

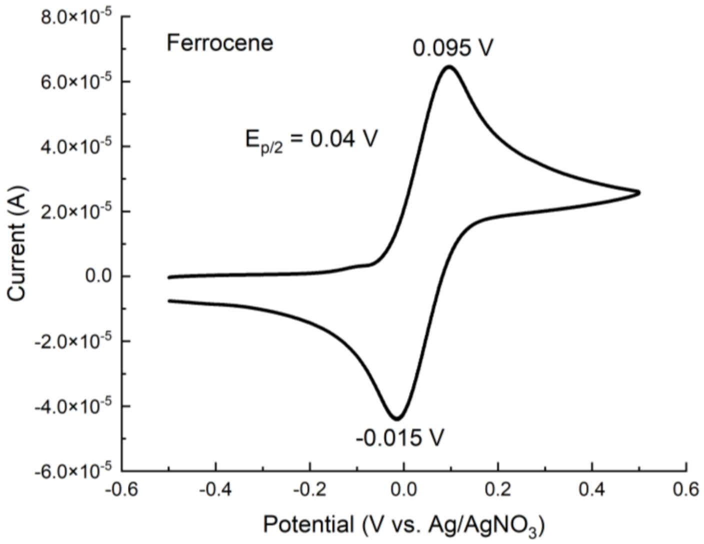

Fig. 2 Cyclic voltammogram of ferrocene (0.004 M) in 0.1 M $^nBuNClO_4$ (DMF), using a glassy carbon working electrode and Pt wire, Ag/$AgNO_3$ (0.1 M in CH_3CN) as counter and reference electrodes at a 100 mV/s scan rate.

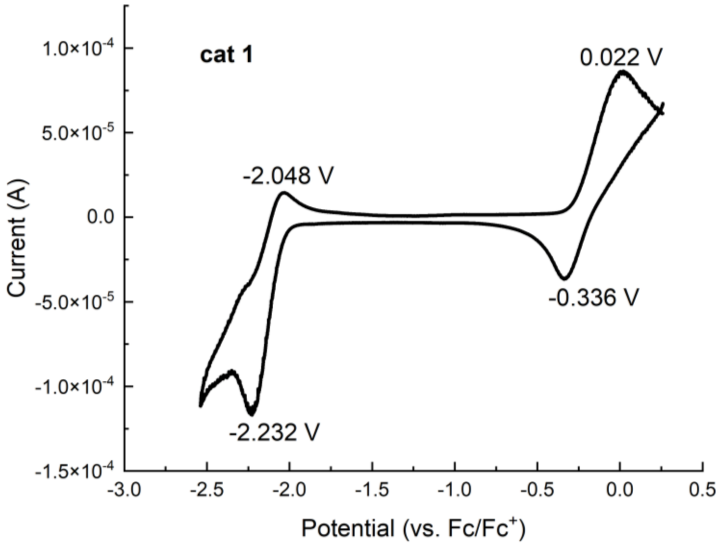

Fig. 3 Cyclic voltammogram of **cat 1** (0.004 M) in 0.1 M $^nBuNClO_4$ (DMF), using a glassy carbon working electrode and Pt wire, Ag/$AgNO_3$ (0.1 M in CH_3CN) as counter and reference electrodes at a 100 mV/s scan rate.

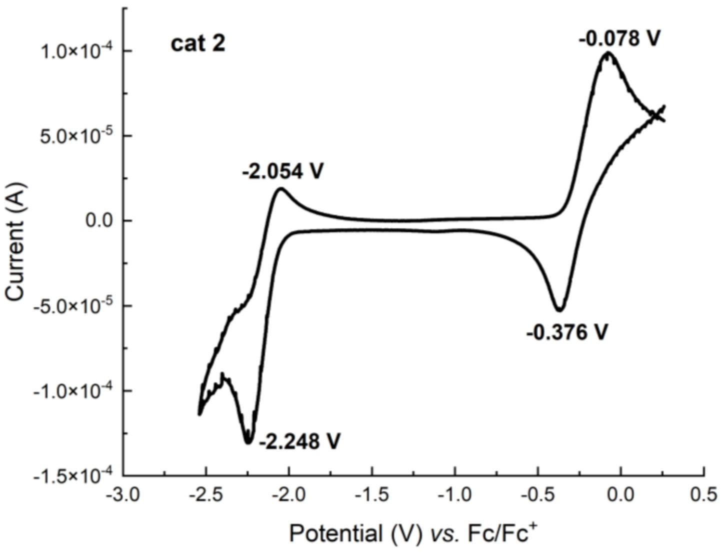

Fig. 4 Cyclic voltammogram of **cat 2** (0.004 M) in 0.1 M ^{n}BuNClO$_4$ (DMF), using a glassy carbon working electrode and Pt wire, Ag/AgNO$_3$ (0.1 M in CH$_3$CN) as counter and reference electrodes at a 100 mV/s scan rate.

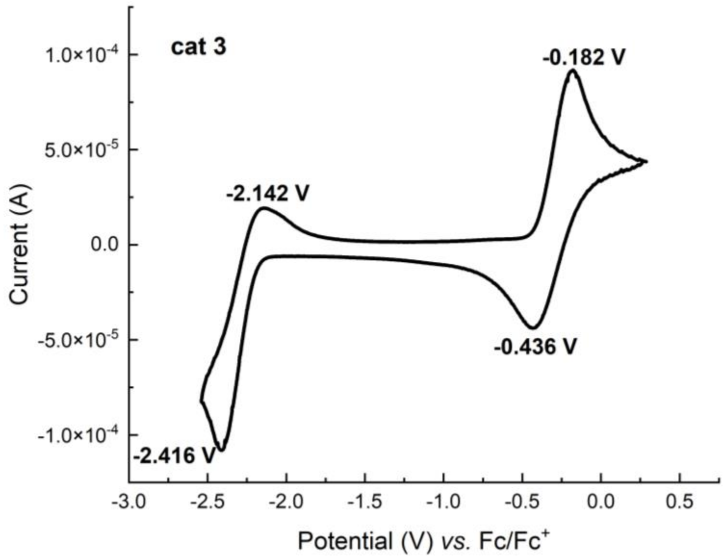

Fig. 5 Cyclic voltammogram of **cat 3** (0.004 M) in 0.1 M ^{n}BuNClO$_4$ (DMF), using a glassy carbon working electrode and Pt wire, Ag/AgNO$_3$ (0.1 M in CH$_3$CN) as counter and reference electrodes at a 100 mV/s scan rate.

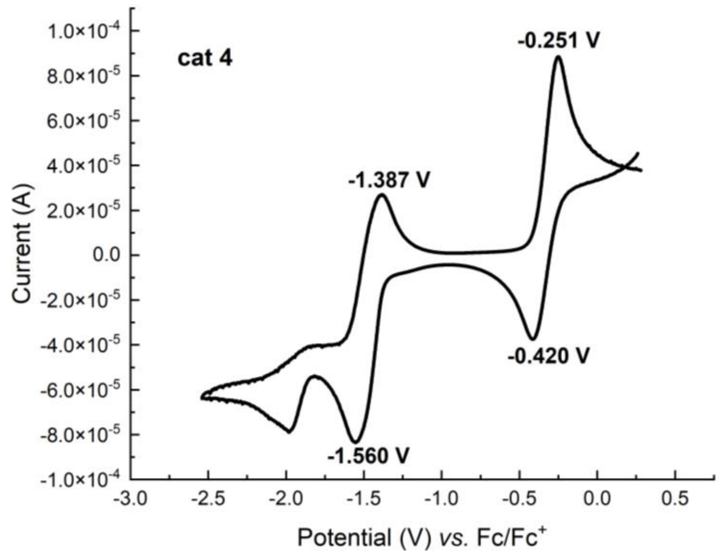

Fig. 6 Cyclic voltammogram of **cat 4** (0.004 M) in 0.1 M ^{n}BuNClO$_4$ (DMF), using a glassy carbon working electrode and Pt wire, Ag/AgNO$_3$ (0.1 M in CH$_3$CN) as counter and reference electrodes at a 100 mV/s scan rate.

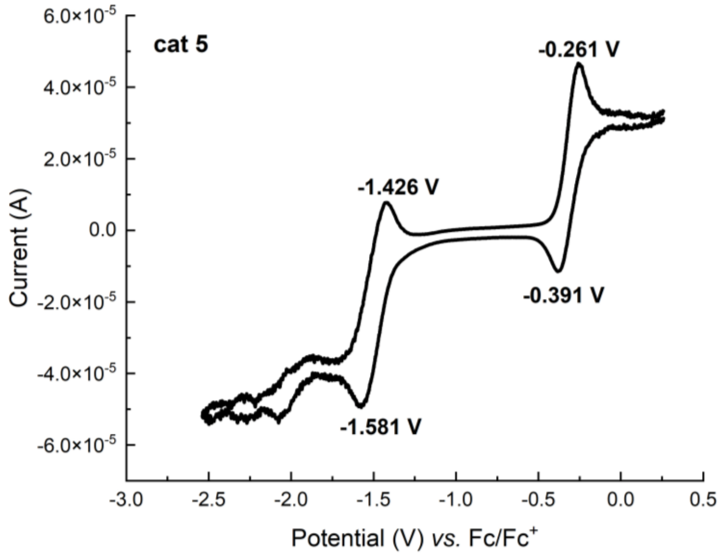

Fig. 7 Cyclic voltammogram of **cat 5** (0.004 M) in 0.1 M ^{n}BuNClO$_4$ (DMF), using a glassy carbon working electrode and Pt wire, Ag/AgNO$_3$ (0.1 M in CH$_3$CN) as counter and reference electrodes at a 100 mV/s scan rate.

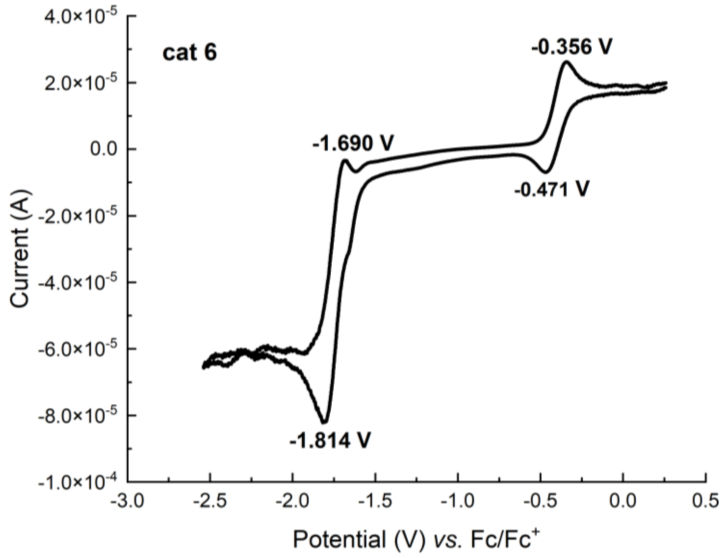

Fig. 8 Cyclic voltammogram of **cat 6** (0.004 M) in 0.1 M ^{n}BuNClO$_4$ (DMF), using a glassy carbon working electrode and Pt wire, Ag/AgNO$_3$ (0.1 M in CH$_3$CN) as counter and reference electrodes at a 100 mV/s scan rate.

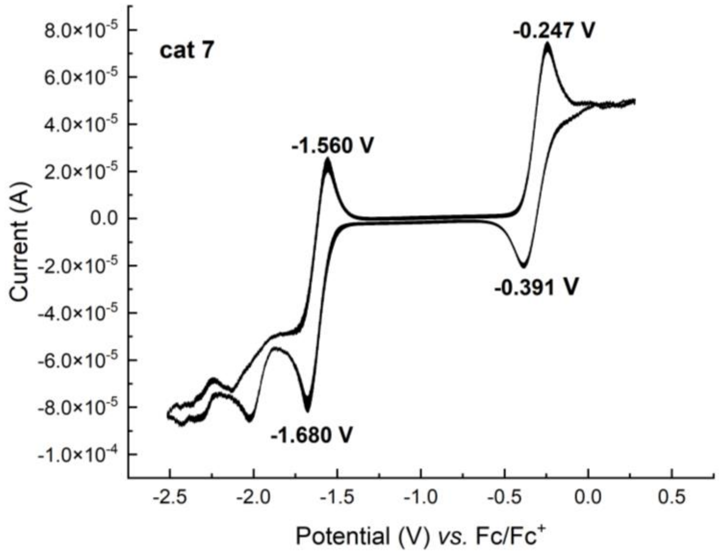

Fig. 9 Cyclic voltammogram of **cat 7** (0.004 M) in 0.1 M ^{n}BuNClO$_4$ (DMF), using a glassy carbon working electrode and Pt wire, Ag/AgNO$_3$ (0.1 M in CH$_3$CN) as counter and reference electrodes at a 100 mV/s scan rate.

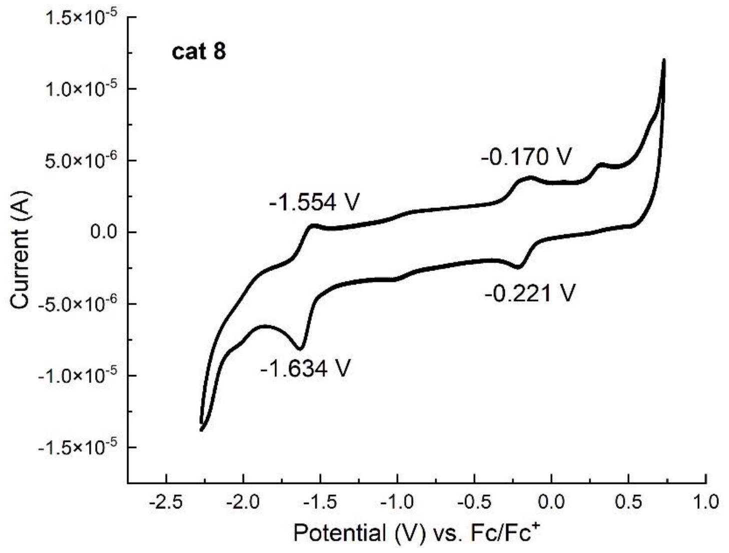

Fig. 10 Cyclic voltammogram of **cat 8** (0.004 M) in 0.1 M ^{n}BuNClO$_4$ (DMF), using a glassy carbon working electrode and Pt wire, Ag/AgNO$_3$ (0.1 M in CH$_3$CN) as counter and reference electrodes at a 100 mV/s scan rate.

We concluded the redox properties of HER catalysts as shown below (Table 1 and Fig. 11). It was found that pentacoordinated catalysts (**cat 1-cat 3,** 1.98-1.92 V) has uniformly larger redox potential gap than that of the conventional catalysts **cat 4-cat 8** (1.14-1.40 V). This result suggests that pentacoordinated Co-salen catalysts is more stable under oxidation and reduction compared with the tetracoordinated counterparts. Noteworthy, CF$_3$ group (**cat 1**) was found to significantly improve the oxidation potential of Co^{II}/Co^{III} with the most positive peak at -0.157 V (*vs.* Fc/Fc$^+$). The bulky substitution in the ligand (**cat 8**) was found to slightly improve the catalyst potential gap.

Table 1. Redox properties of **cat 1–cat 8.**

HER catalyst	$E_{p/2}$(Co^{II}/Co^{III})	$E_{p/2}$(Co^{I}/Co^{II})
**cat 1**	-0.157 V	-2.140 V
**cat 2**	-0.227 V	-2.151 V
**cat 3**	-0.309 V	-2.279 V
**cat 4**	-0.336 V	-1.474 V
**cat 5**	-0.326 V	-1.504 V
**cat 6**	-0.414 V	-1.752 V
**cat 7**	-0.319 V	-1.620 V
**cat 8**	-0.196 V	-1.594 V

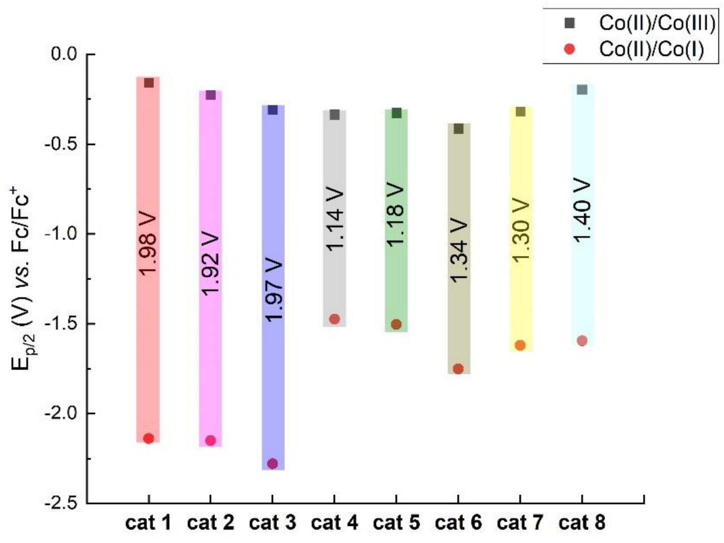

Fig. 11 Redox potential gap of **cat 1–cat 8.**

We also investigated the redox behavior of various H$^-$T catalysts with cyclic voltametric approach (Fig. 12-18).

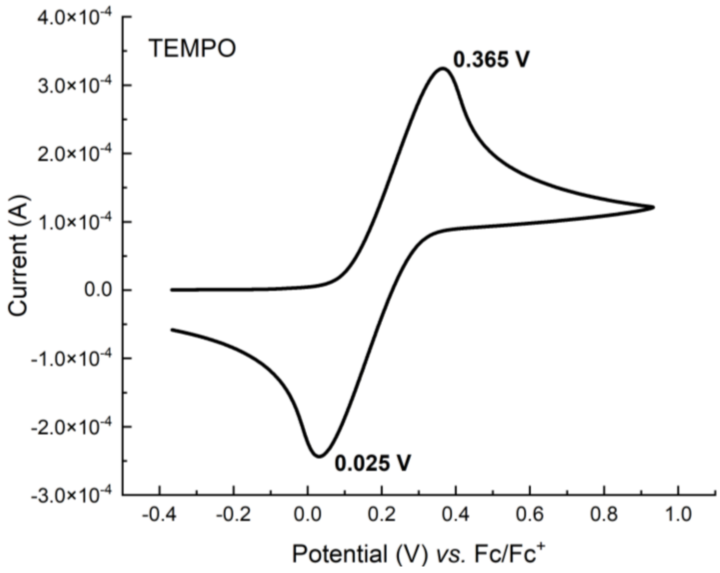

Fig. 12 Cyclic voltammogram of TEMPO (0.02 M) in 0.1 M ^{n}BuNClO$_4$ (DMF), using a glassy carbon working electrode and Pt wire, Ag/AgNO$_3$ (0.1 M in CH$_3$CN) as counter and reference electrodes at a 100 mV/s scan rate.

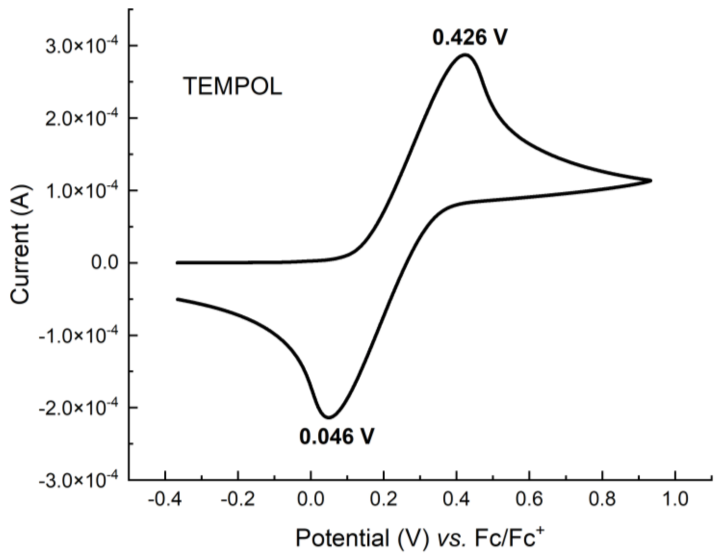

Fig. 13 Cyclic voltammogram of TEMPOL (0.02 M) in 0.1 M ^{n}BuNClO$_4$ (DMF), using a glassy carbon working electrode and Pt wire, Ag/AgNO$_3$ (0.1 M in CH_3CN) as counter and reference electrodes at a 100 mV/s scan rate.

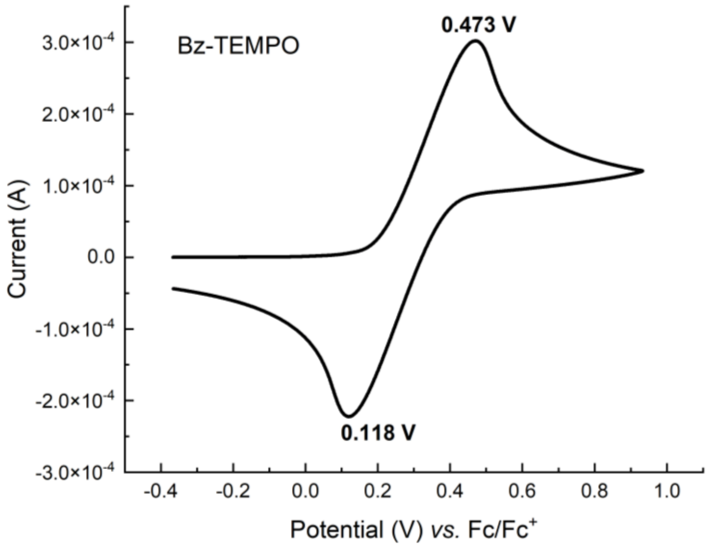

Fig. 14 Cyclic voltammogram of Bz-TEMPO (0.02 M) in 0.1 M ^{n}BuNClO$_4$ (DMF), using a glassy carbon working electrode and Pt wire, Ag/AgNO$_3$ (0.1 M in CH_3CN) as counter and reference electrodes at a 100 mV/s scan rate.

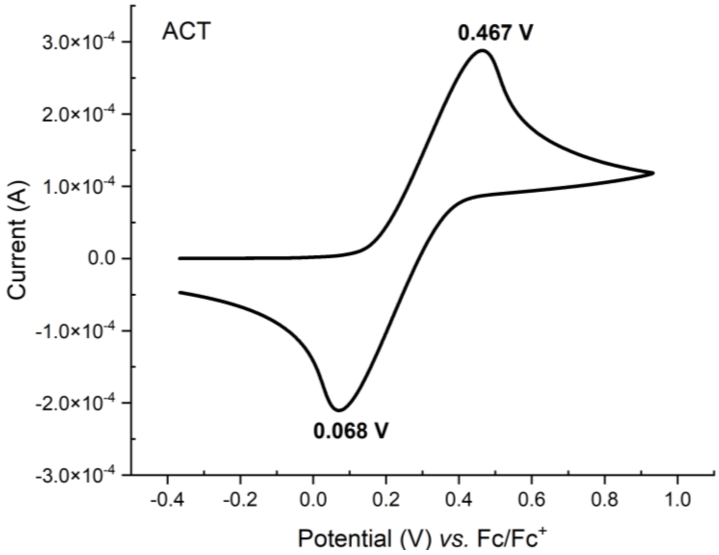

Fig. 15 Cyclic voltammogram of ACT (0.02 M) in 0.1 M ^{n}BuNClO$_4$ (DMF), using a glassy carbon working electrode and Pt wire, Ag/AgNO$_3$ (0.1 M in CH$_3$CN) as counter and reference electrodes at a 100 mV/s scan rate.

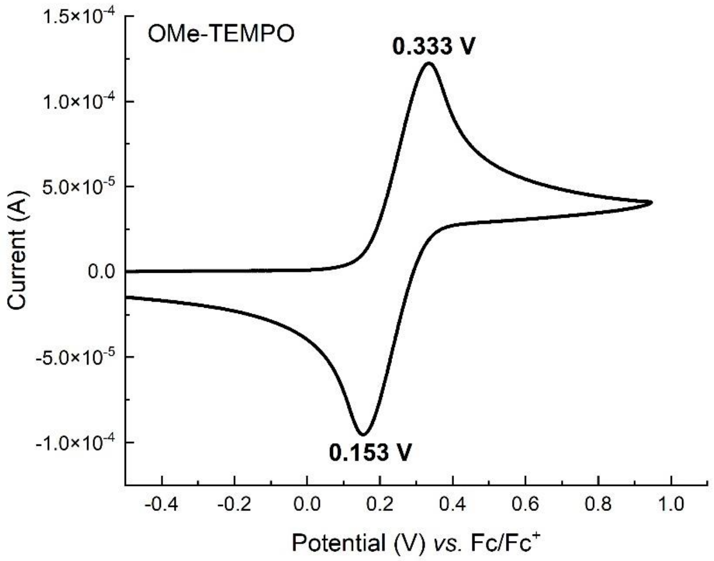

Fig. 16 Cyclic voltammogram of OMe-TEMPO (0.02 M) in 0.1 M ^{n}BuNClO$_4$ (DMF), using a glassy carbon working electrode and Pt wire, Ag/AgNO$_3$ (0.1 M in CH$_3$CN) as counter and reference electrodes at a 100 mV/s scan rate.

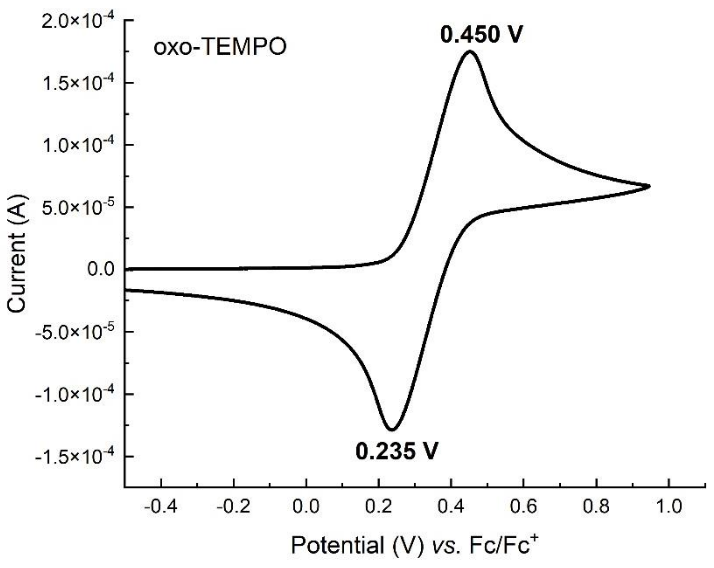

Fig. 17 Cyclic voltammogram of oxo-TEMPO (0.02 M) in 0.1 M ^{n}BuNClO$_4$ (DMF), using a glassy carbon working electrode and Pt wire, Ag/AgNO$_3$ (0.1 M in CH_3CN) as counter and reference electrodes at a 100 mV/s scan rate.

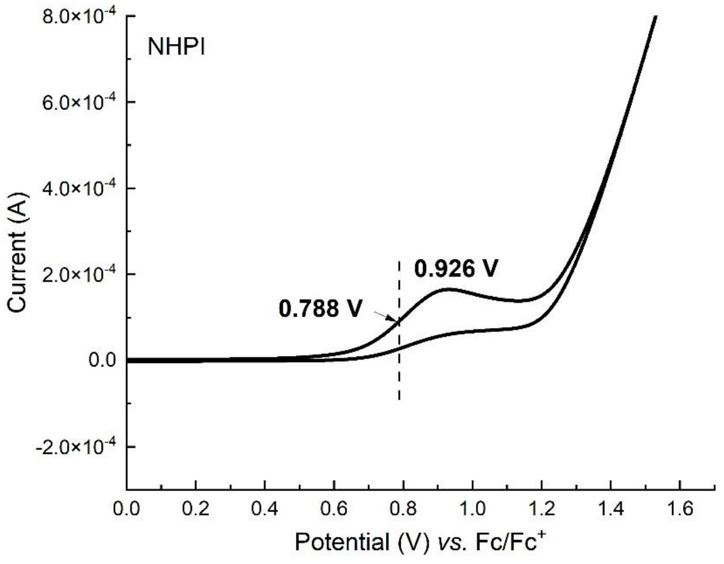

Fig. 18 Cyclic voltammogram of NHPI (0.02 M) in 0.1 M ^{n}BuNClO$_4$ (DMF), using a glassy carbon working electrode and Pt wire, Ag/AgNO$_3$ (0.1 M in CH_3CN) as counter and reference electrodes at a 100 mV/s scan rate.

By comparing the cyclic voltammograms of H-T catalysts with that of Co-salen catalyst, **cat 1** has closest oxidation potential with TEMPO, which made it possible using **cat 1** to facilitate the oxidation TEMPO. Further cyclic voltammetry experiment mixing **cat 1** with TEMPO verifies the above conclusion with the detection of obvious increase of the anodic peak (catalytic current) of **cat 1 (Fig. 19).** Nevertheless, other cobalt catalysts failed to produce catalytic current when mixing with TEMPO.

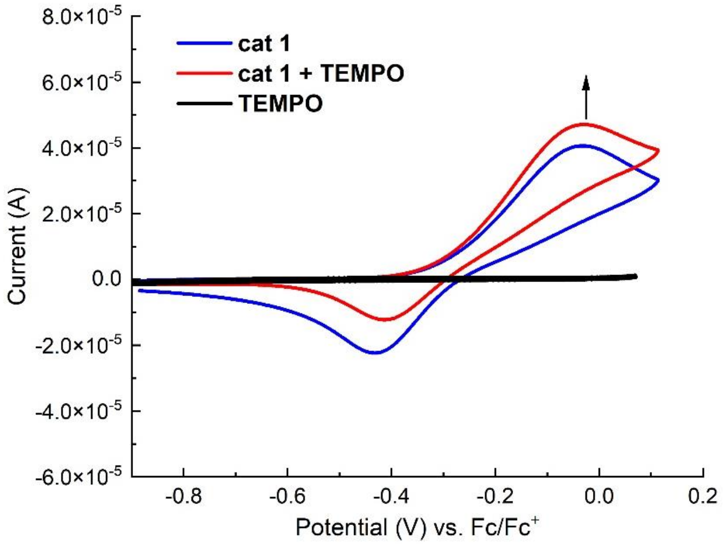

Fig. 19 Cyclic voltammogram of TEMPO (0.04 M) and **cat 1** (0.004 M) in 0.1 M ^{n}BuNClO$_4$ (DMF), using a glassy carbon working electrode and Pt wire, Ag/AgNO$_3$ (0.1 M in CH$_3$CN) as counter and reference electrodes at a 100 mV/s scan rate.

We next investigated the electrochemical behavior of substrates **1a** and **2a**. As shown in **Fig. 20**, **1a** and **2a** seem to be redox inert within the window of -2.5-2.5 V (*vs.* Fc/Fc$^+$).

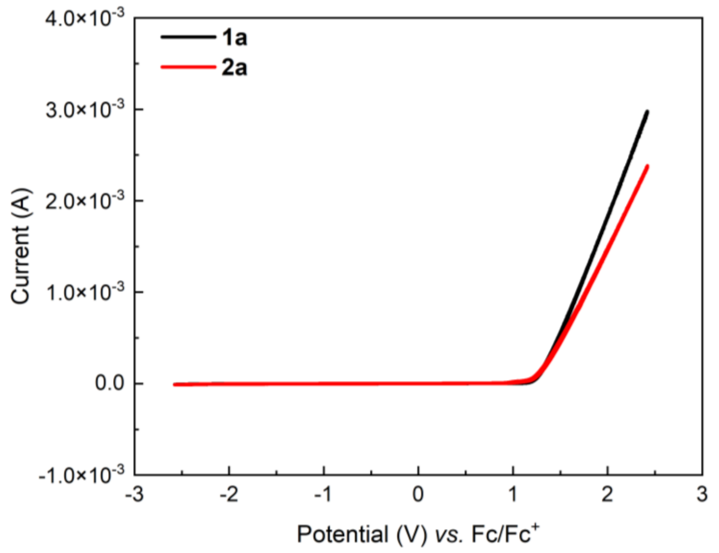

Fig. 20 Cyclic voltammogram of **1a** (0.02 M) and **2a** (0.02 M) in 0.1 M ^{n}BuNClO$_4$ (DMF) containing Cs$_2$CO$_3$ (0.02 M), using a glassy carbon working electrode and Pt wire, Ag/AgNO$_3$ (0.1 M in CH$_3$CN) as counter and reference electrodes at a 100 mV/s scan rate.

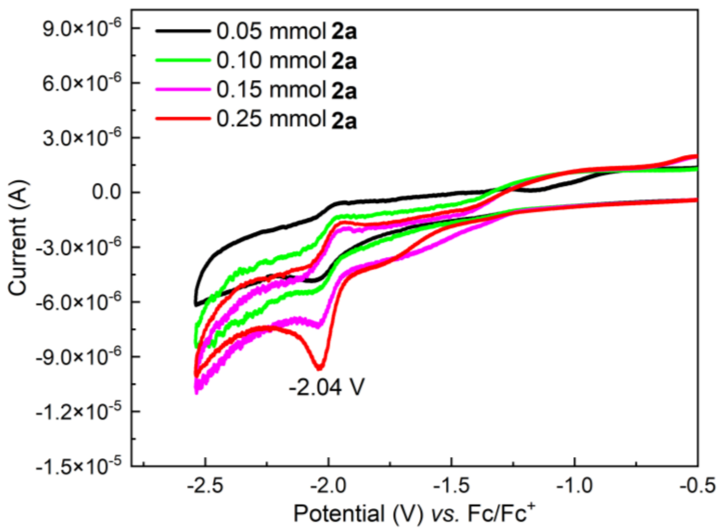

Fig. 21 Cyclic voltammogram of **2a** in 0.1 M ^{n}BuNClO$_4$ (DMF 2.5 mL) containing Cs_2CO_3 (0.05 mmol), using a glassy carbon working electrode and Pt wire, Ag/AgNO$_3$ (0.1 M in CH_3CN) as counter and reference electrodes at a 100 mV/s scan rate.

After enlarging the range from -0.5 to -2.5 V, a couple of small waves (10^{-6}~10^{-5} A) were detected at -2.04 V and their intensity increases with the concentration of **2a**, which were attributed to the hydrogen absorption and desorption of **2a.**

The catalytic role of **cat 1** and TEMPO was explored as shown in the **Fig. 22-25.**

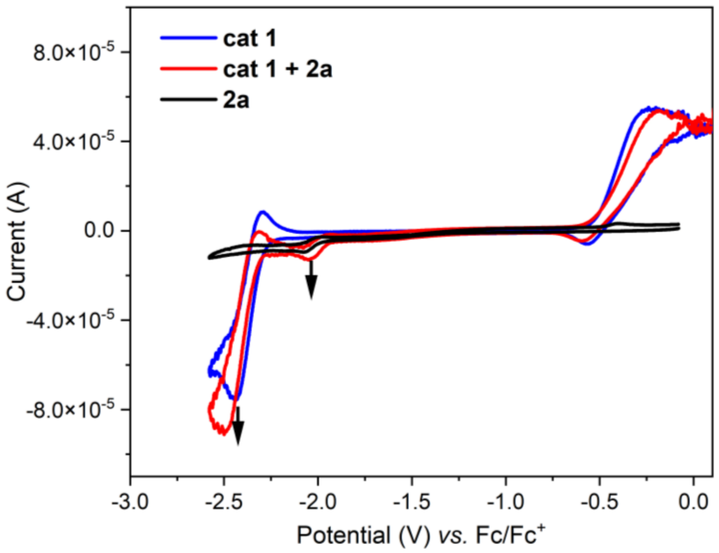

Fig. 22 Cyclic voltammogram of **2a** (0.3 mmol) and **cat 1** (0.01 mmol) in 0.1 M ^{n}BuNClO$_4$ (DMF 2.5 mL) containing Cs_2CO_3 (0.05 mmol), using a glassy carbon working electrode and Pt wire, Ag/AgNO$_3$ (0.1 M in CH_3CN) as counter and reference electrodes at a 100 mV/s scan rate.

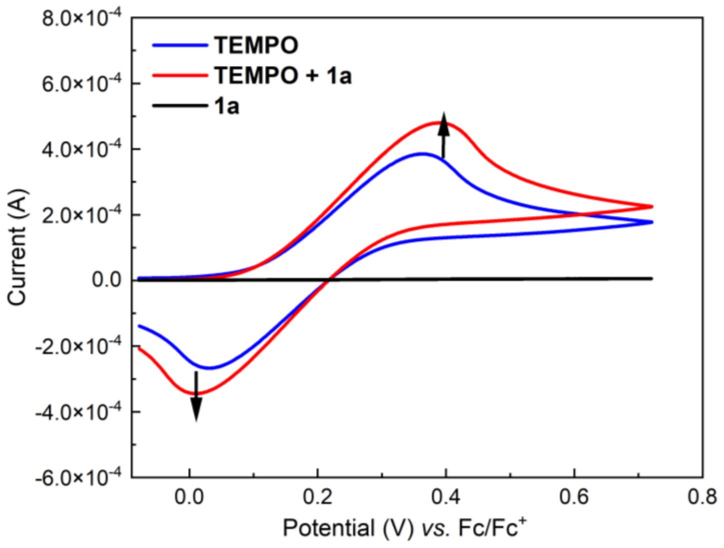

Fig. 23 Cyclic voltammogram of **1a** (0.04 M) and TEMPO (0.02 mmol) in 0.1 M ^{n}BuNClO$_4$ (DMF 2.5 mL), using a glassy carbon working electrode and Pt wire, Ag/AgNO$_3$ (0.1 M in CH$_3$CN) as counter and reference electrodes at a 100 mV/s scan rate.

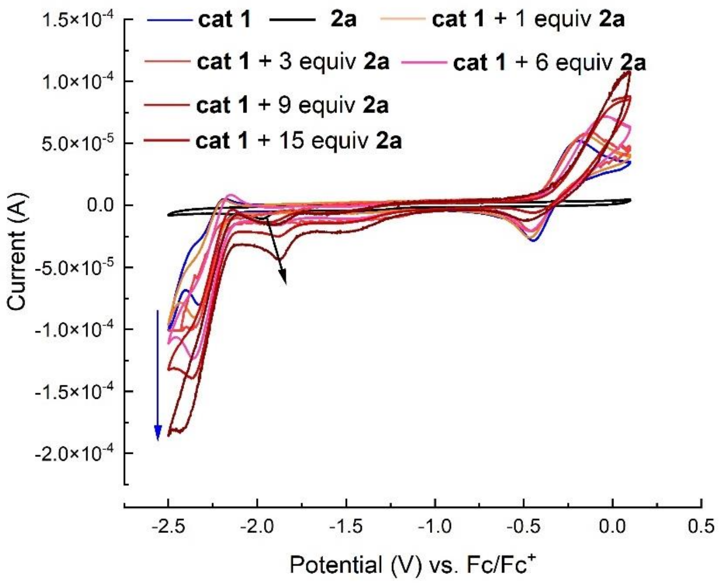

Fig. 24 Titration experiment: cyclic voltammogram of **2a** (0.01-0.15 mmol) and **cat 1** (0.01 mmol) in 0.1 M ^{n}BuNClO$_4$ (DMF 2.0 mL) containing Cs$_2$CO$_3$ (0.05 mmol), using a glassy carbon working electrode and Pt wire, Ag/AgNO$_3$ (0.1 M in CH$_3$CN) as counter and reference electrodes at a 100 mV/s scan rate.

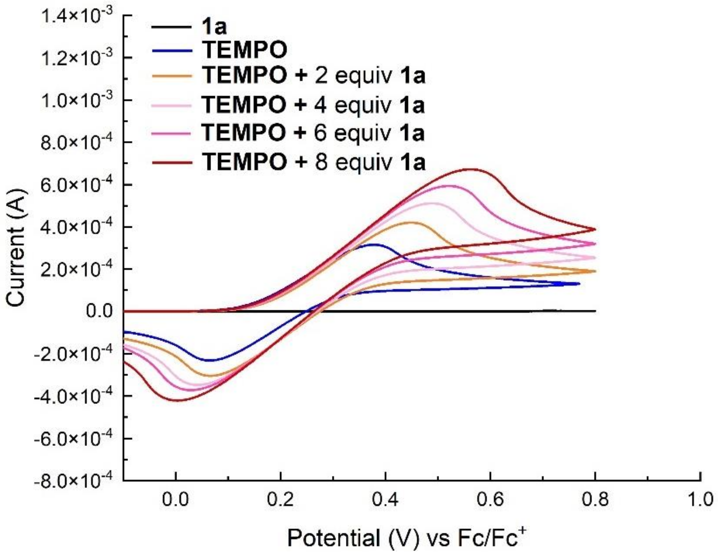

Fig. 25 Titration experiment: cyclic voltammogram of **1a** (0.1-0.4 mmol) and **TEMPO** (0.05 mmol) in 0.1 M ^{n}BuNClO$_4$ (DMF 2.5 mL), using a glassy carbon working electrode and Pt wire, Ag/AgNO$_3$ (0.1 M in CH$_3$CN) as counter and reference electrodes at a 100 mV/s scan rate.

4. GC analysis for the byproduct hydrogen

To verify the hydrogen byproduct during reaction, we also conducted a GC analysis for the reaction atmosphere. After electrolysis, the headspace atmosphere of the reaction tube was subjected to GC analysis. As shown in the GC spectra, substantial amount of hydrogen was detected by comparing with the standard sample containing H_2, O_2, N_2. The result clearly confirmed the HER process and the hydrogen byproduct.

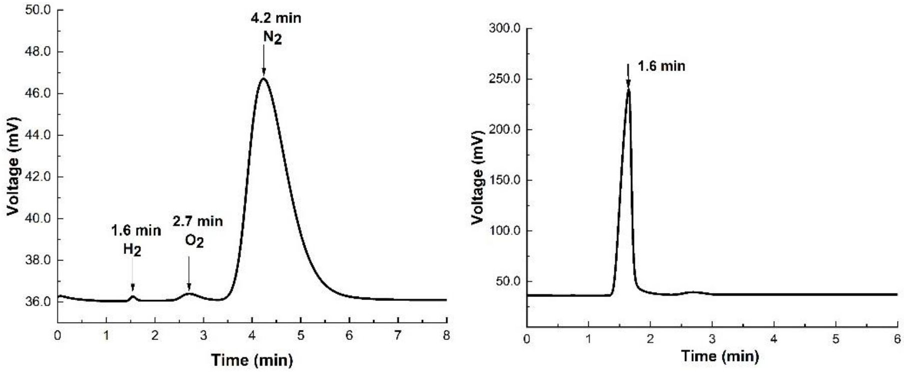

Fig. 26 GC spectrum of standard sample (containing H_2, O_2, N_2) and the reaction atmosphere (after reaction)

5. Optimization of reaction conditions

Table 2. Optimization of cross-coupling reaction between benzyl alcohol **1a** and allylbenzene **2a**[a]

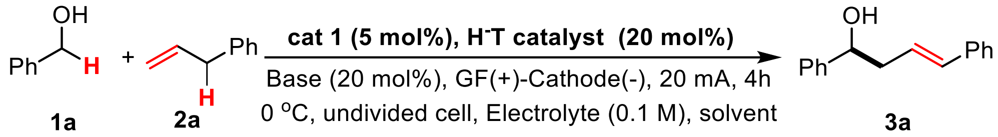

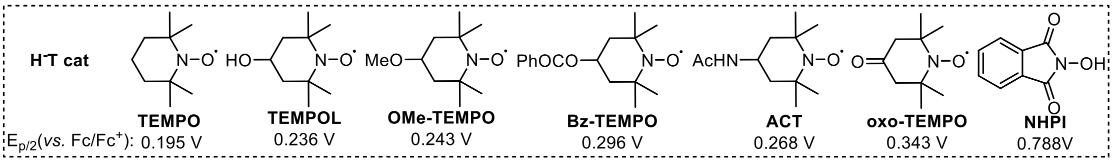

Entry	Electrolyte	Cathodes	Solvent	Base	H·T catalyst	Yield (%)[b]
1	^{n}Bu$_4$NClO$_4$	Cu	DMF	Cs_2CO_3	**TEMPO**	85
2	^{n}Bu$_4$NBF$_4$	Cu	DMF	Cs_2CO_3	**TEMPO**	80
3	^{n}Bu$_4$NPF$_6$	Cu	DMF	Cs_2CO_3	**TEMPO**	76
4	^{n}Bu$_4$NBr	Cu	DMF	Cs_2CO_3	**TEMPO**	trace
5	^{n}Bu$_4$NOAc	Cu	DMF	Cs_2CO_3	**TEMPO**	trace
6	^{n}Bu$_4$NClO$_4$	Pt	DMF	Cs_2CO_3	**TEMPO**	50
7	^{n}Bu$_4$NClO$_4$	Ni	DMF	Cs_2CO_3	**TEMPO**	78
8	^{n}Bu$_4$NClO$_4$	Zn	DMF	Cs_2CO_3	**TEMPO**	70
9	^{n}Bu$_4$NClO$_4$	Cu	CH_3CN	Cs_2CO_3	**TEMPO**	trace
10	^{n}Bu$_4$NClO$_4$	Cu	DMA	Cs_2CO_3	**TEMPO**	62
11	^{n}Bu$_4$NClO$_4$	Cu	DMSO	Cs_2CO_3	**TEMPO**	32
12	^{n}Bu$_4$NClO$_4$	Cu	DMF	K_2CO_3	**TEMPO**	68
13	^{n}Bu$_4$NClO$_4$	Cu	DMF	Na_2CO_3	**TEMPO**	65
14	^{n}Bu$_4$NClO$_4$	Cu	DMF	Li_2CO_3	**TEMPO**	43
15	^{n}Bu$_4$NClO$_4$	Cu	DMF	none	**TEMPO**	25
16	^{n}Bu$_4$NClO$_4$	Cu	DMF	Cs_2CO_3	**TEMPOL**	38
17	^{n}Bu$_4$NClO$_4$	Cu	DMF	Cs_2CO_3	**OMe-TEMPO**	74
18	^{n}Bu$_4$NClO$_4$	Cu	DMF	Cs_2CO_3	**Bz-TEMPO**	39
19	^{n}Bu$_4$NClO$_4$	Cu	DMF	Cs_2CO_3	**ACT**	55
20	^{n}Bu$_4$NClO$_4$	Cu	DMF	Cs_2CO_3	**oxo-TEMPO**	60
21	^{n}Bu$_4$NClO$_4$	Cu	DMF	Cs_2CO_3	**NHPI**	20

[a] Reaction conditions: **1a** (0.5 mmol), **2a** (1.5 mmol), electrolyte (1 mmol), base (0.1 mmol), HER catalyst (0.025 mmol), TEMPO (0.1 mmol), graphite felt anode, metal cathode, CCE (20 mA, 4h), 0 ˚C; [b] Isolated yield.

We obtained the optimal conditions by screening electrolytes (Entries 1-5), cathodes (Entries 6-8), reaction solvents (Entries 9-11), base additives (Entries 12-15) and H·T catalysts (Entries 16-21). It was found that halide, acetate electrolytes (Entries 4-5) failed to give desired products. Weak acidic acetonitrile (Entry 9) also led to diminished yields, as it would interfere the HER process of substrate **2a**. Base additives played a role to promote the proton dissociation of **2a** and it directly affected the reaction performance. Variations of H·T catalyst suggests that the catalyst with lower oxidation peak would benefit the reaction performance, and dysoxidizable mediator NHPI (Entry 21) lead to diminished yield.

Table 3. Screening conventional CDC conditions for the reaction between **1a** and **2a**

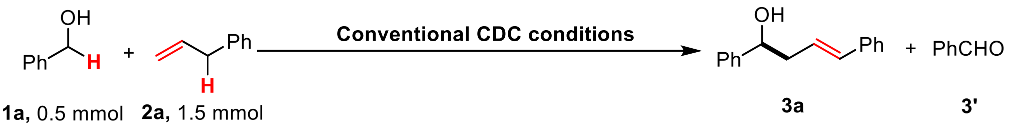

Entry	Conventional CDC conditions	Yield of 3a/3' (%)^b
1	$K_2S_2O_8$ (1 mmol), **cat 1** (5 mol%), Cs_2CO_3 (20 mol%), TEMPO (20 mol%), DMF (2 mL), rt	0/82
2	DDQ (1 mmol), **cat 1** (5 mol%), Cs_2CO_3 (20 mol%), TEMPO (20 mol%), DMF (2 mL), rt	0/0
3	$PhI(OAc)_2$ (1 mmol), **cat 1** (5 mol%), Cs_2CO_3 (20 mol%), TEMPO (20 mol%), DMF (2 mL), rt	0/47
4	CuBr (10 mol%), ^{t}BuOOH (1 mmol), toluene (2 mL), 80 °C	0/56
5	CuBr (10 mol%), DDQ (1 mmo), toluene (2 mL), 80 °C	0/0
6	$Pd(OAc)_2$ (5 mol%), ^{t}BuOOH (1 mmol), toluene (2 mL), 80 °C	0/71
7	$Pd(OAc)_2$ (5 mol%), DDQ (1 mmo), toluene (2 mL), 80 °C	0/0
8	$Pd(OAc)_2$ (5 mol%), ^{t}BuOOH (1 mmol), TEMPO (20 mol%), toluene (2 mL), 80 °C	0/78

Various conventional CDC conditions were also tested in the reactions (**Table 3**). No desired product **3a** was detected although direct oxidation product benzaldehyde **3a** was observed in some cases. Notably, increasing the reaction temperature led to mixture of various overoxidation byproducts (Entries 4-8). *trans*-Cinnamaldehyde arising from the oxidation of **2a** was uniformly detected (Entries 4-8) and the ^{1}H NMR spectra of the crude product was shown below (Fig. 27).

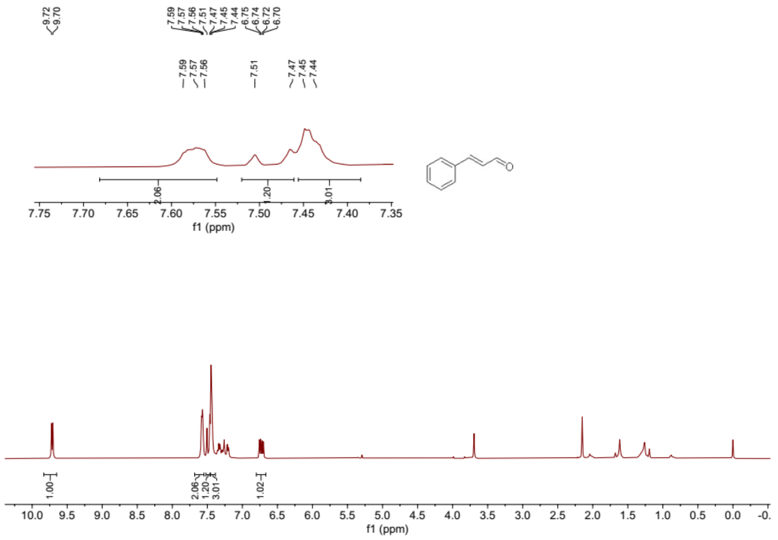

Fig. 27 ^{1}H NMR spectra of the crude *trans*-Cinnamaldehyde.

6. General procedure for the electrochemical CDC reaction

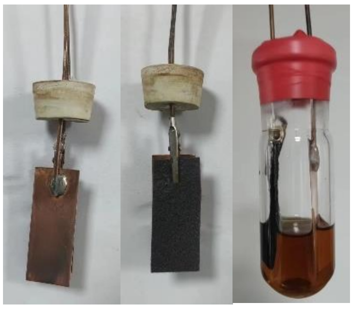

Fig. 28 Electrolysis setup (graphite felt, copper plate: width 1.8 cm, immersion depth 1.5 cm)

3a as example

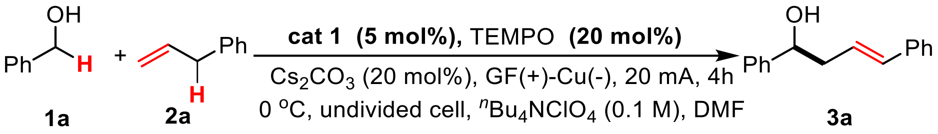

An undivided cell was equipped with a magnet stirrer, copper plate (1.8 *1.5 cm^2), graphite felt (1.8 *1.5 cm^2), as cathode and anode, respectively (the electrolysis setup is shown in Fig. 28). The substrate benzyl alcohol (52 μL, 0.5 mmol), allylbenzene **2a** (199 μL, 1.5 mmol), Cs_2CO_3 (31 mg, 0.1 mmol), **cat 1** (14 mg, 0.025 mmol), TEMPO (16 mg, 0.1 mmol) and $^n Bu_4NClO_4$ (341 mg, 1 mmol) were added to the solvent DMF (10 mL). The resulting mixture was allowed to stir and electrolyze under constant current condition (20 mA, J = 7.4 mA·cm^{-2}) at 0 °C for 4 hours. The reaction mixture was subsequently poured into water (100 mL) and extracted with ethyl acetate (40 mL×3). The combined organic phases were washed with saturated brine solution (100 mL). The volatile solvent was then removed with a rotary evaporator, and the residue was purified by column chromatography (PE/ EA= 8/1-5/1, v/v) on silica gel to afford the desired product **3a** (95 mg) in 85 % yield.

The reaction of electron-deficient benzyl alcohols was conducted with the modified conditions, in which the amount of Cs_2CO_3 was reduced to (17 mg, 0.05 mmol).

Safety warning: Perchlorates ($^n Bu_4NClO_4$) are energetic materials and can be shock-sensitive when used in the presence of organic substances or metals, and are associated with health hazards (potent thyroid hormone disruptors)

7. Procedure for gram scale reaction and derivatization of products

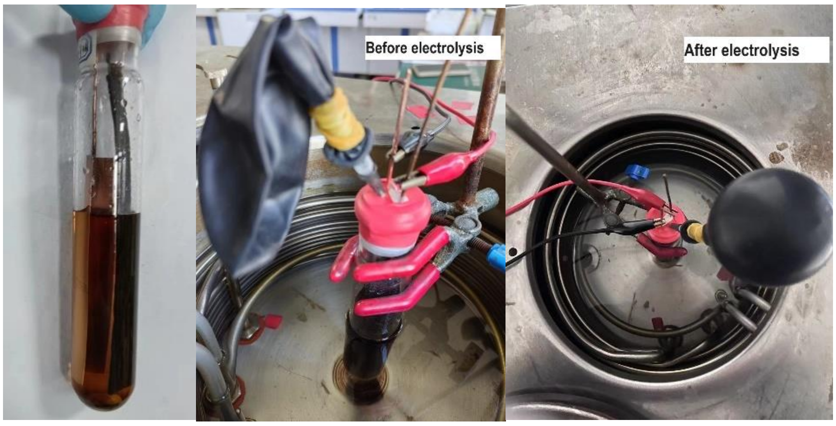

Fig. 29 Gram electrolysis device (graphite felt: immersion depth 7.5 cm, width 2.0 cm; copper plate: immersion depth 7.5 cm, width 3.0 cm)

Gram scale reaction

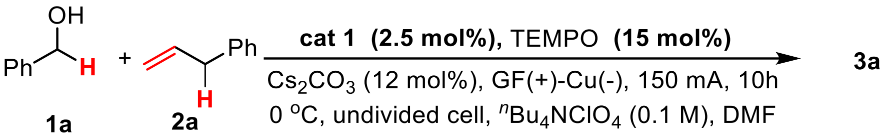

An undivided cell was equipped with a magnet stirrer, copper plate (7.5 *3.0 cm^2), graphite felt (7.5 *2 cm^2), as cathode and anode, respectively (the electrolysis setup is shown in Fig. 29). The substrate benzyl alcohol (1.1 mL, 10 mmol), allylbenzene **2a** (2.7 mL, 20 mmol), Cs_2CO_3 (400 g, 1.2 mmol), **cat 1** (140 mg, 0.25 mmol), TEMPO (240 mg, 1.5 mmol) and $^{n}Bu_4NClO_4$ (1.36 g, 4 mmol) were added to the solvent DMF (40 mL). The resulting mixture was allowed to stir and electrolyze at constant current condition (150 mA) at 0 °C for 10 hours. Then the reaction mixture was poured into water (300 mL) and extracted with ethyl acetate (80 mL×3). Combined organic phase was washed with saturated brine solution (250 mL). The volatile solvent was then removed with a rotary evaporator, and the residue was purified by column chromatography (PE/EA = 8/1-5/1, v/v) on silica gel to afford the desired product **3a** (1.57 g) in 70 % yield.

Experiment using solar cell as power supply

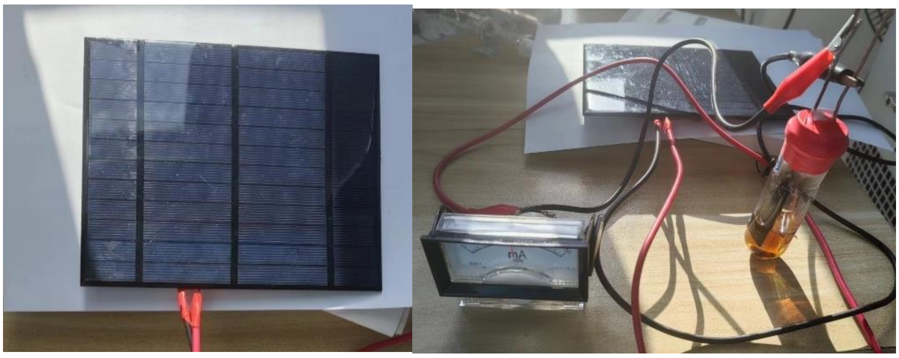

Fig. 30 Electrolysis setup with a single solar panel (size: 135 mm*165mm, purchased from Ningbo Aike Electronic Technology Co., Ltd) as electricity supply (9:00-13:00, Nov 18, 2023 at Hefei, China)

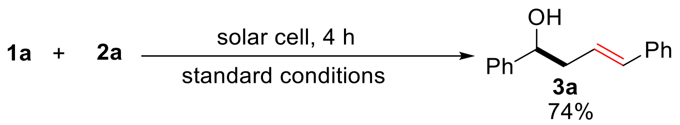

An undivided cell was equipped with a magnet stirrer, copper plate (1.8 *1.5 cm^2), graphite felt (1.8 *1.5 cm^2), as cathode and anode, respectively (the electrolysis setup is shown in Fig. 30). The substrate benzyl alcohol (52 µL, 0.5 mmol), allylbenzene **2a** (199 µL, 1.5 mmol), Cs_2CO_3 (31 mg, 0.1 mmol), **cat 1** (14 mg, 0.025 mmol), TEMPO (16 mg, 0.1 mmol) and $^{n}Bu_4NClO_4$ (341 mg, 1 mmol) were added to the solvent DMF (10 mL). The resulting mixture was allowed to stir and electrolyze using a solar cell as electricity supply under sunlight for 4 hours (current 30 ~ 15 mA). The reaction mixture was subsequently poured into water (100 mL) and extracted with ethyl acetate (40 mL×3). The combined organic phases were washed with saturated brine solution (100 mL). The volatile solvent was then removed with a rotary evaporator, and the residue was purified by column chromatography (PE/ EA= 8/1-5/1, v/v) on silica gel to afford the desired product **3a** (83 mg) in 74 % yield.

Dehydration of product 3l

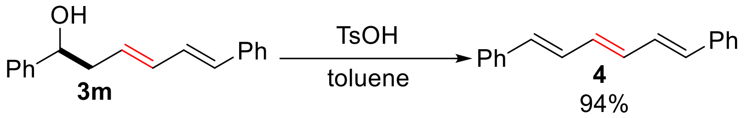

A flask was charged with **3m** (0.5 mmol, 125 mg), *p*-toluenesulfonic acid monohydrate (0.05 mmol, 10 mg) and toluene (3 mL). The resulted mixture was heated to 100 °C. After 5 hours, the volatile solvent was removed with a rotary evaporator. The residue was purified by column chromatography (PE/ EA= 100/1-50/1, v/v) on silica gel to afford the desired product **4** (110 mg) in 94 % yield.

Cyclization of product 3a

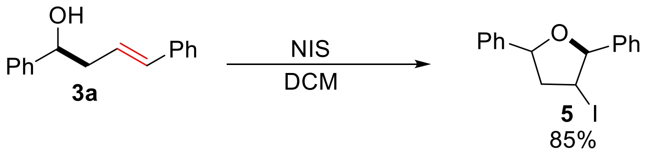

A flask was charged with **3a** (1 mmol, 224 mg), *N*-iodosuccinimide (NIS) (1.0 mmol, 224 mg) and DCM (3 mL). After stirring at room temperature 6 hours, the volatile solvent was removed with a rotary evaporator. The residue was purified by column chromatography (PE/ EA= 50/1-20/1, v/v) on silica gel to afford the desired product **5** (298 mg) with two isomers.

8. Procedure and details for control experiments

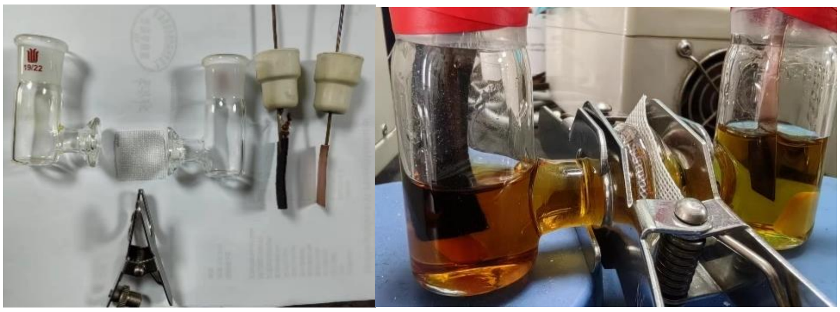

Fig. 31 Electrolysis setup of divided cell

Electrolysis in divided cell

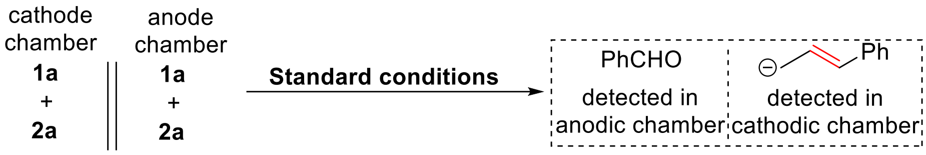

A divided cell was equipped with a magnet stirrer, copper plate (1.2 *1.0 cm^2), graphite felt (1.2 *1.0 cm^2), as cathode and anode, respectively (the electrolysis setup is shown in Fig. 31). The substrate benzyl alcohol (52 µL, 0.5 mmol), allylbenzene **2a** (199 µL, 1.5 mmol), Cs_2CO_3 (31 mg, 0.1 mmol), **cat 1** (14 mg, 0.025 mmol), TEMPO (16 mg, 0.1 mmol) and $^nBu_4NClO_4$ (341 mg, 1 mmol) were added to the solvent DMF (10 mL). The resulting mixture was divided equally between anodic and cathodic chambers. The divided cell system was allowed to stir and electrolyze at constant current condition (20 mA) at room temperature. In the anodic chamber, benzaldehyde **1a** (14 mg) was detected, while a brown species was detected over surface of cathode. To identify the species, UV-vis experiment was conducted as shown below (Fig. 32). By comparing with the carbanions[5] generating from ^nBuLi, we speculated that the species over cathode might be the carbanion species with characteristic absorption peak at 284, 294 and 330 nm.

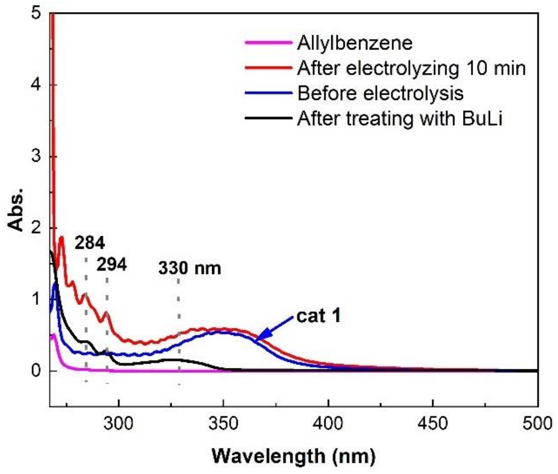

Fig. 32 UV-vis spectra of substrate **2a** (0.0025 M in DMF), carbanion generated by using ^nBuLi (in THF), electrolysis solution in cathodic chamber.

Replacing electrolysis with a stoichiometric oxidant

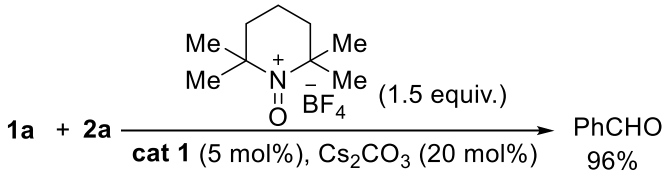

A flask was charged with **1a** (52 μL, 0.5 mmol), **2a** (199 μL, 1.5 mmol), **cat 1** (14 mg, 0.025 mmol), Cs_2CO_3 (31 mg, 0.1 mmol), 2,2,6,6-tetramethyl-1-oxopiperidinium tetrafluoroborate (182 mg, 0.75 mmol) and DMF (10 mL). After stirring at room temperature 24 hours, the reaction mixture was poured into water (100 mL) and extracted with ethyl acetate (40 mL×3). Combined organic phase was washed with saturated brine solution (200 mL). The volatile solvent was then removed with a rotary evaporator, and the residue was purified by column chromatography (PE/EA = 50/1-30/1, v/v) on silica gel to afford benzaldehyde (51 mg) in 96 % yield.

Kinetic isotope effect study using 1a-d$_1$ as substrate

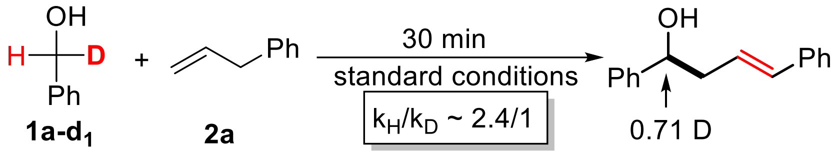

We conducted KIE study by using **1a-d$_1$** as substrate. Under standard conditions, we isolated product **3a** (18 mg, 16% yield) after electrolyzing 30 min. The deuteration was detected by ^{1}H NMR spectra as shown below (**Fig. 33**).

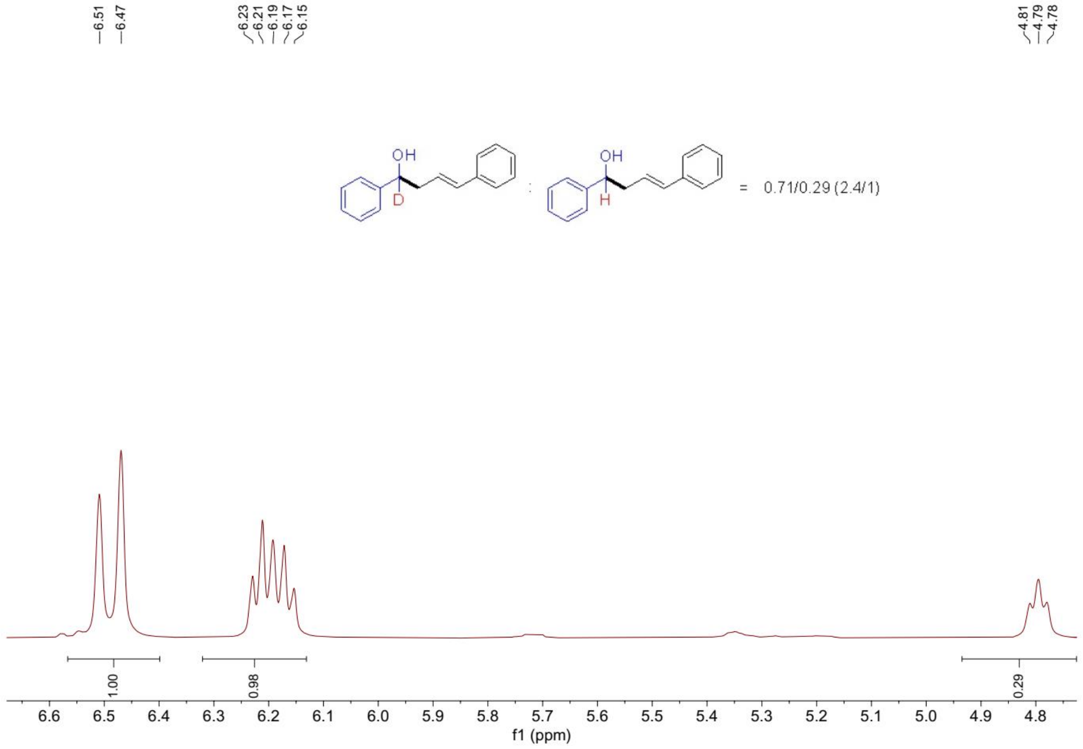

Fig. 33 ^{1}H NMR spectra of product **3a**

Kinetic isotope effect study with parallel experiment

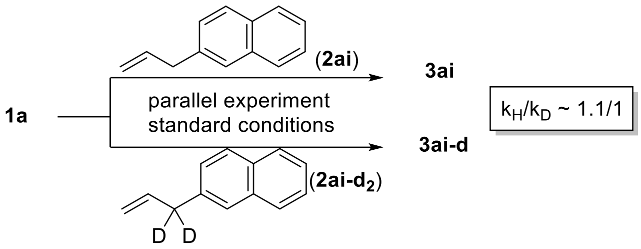

2ai *as substrate*

Reaction time	0 min	10 min	20 min	40 min	60 min
Reaction yield	0%	7%	12%	27%	39%

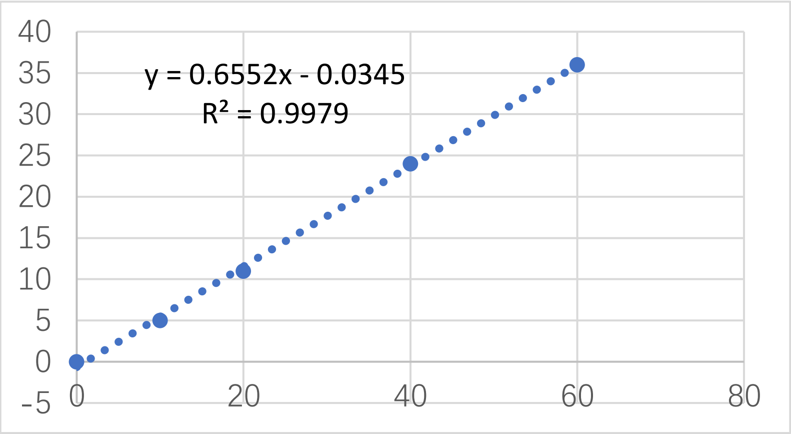

Fig. 34 Reaction rate testing for **3ai**

2ai-d$_2$ *as substrate*

Reaction time	0 min	10 min	20 min	40 min	60 min
Reaction yield	0%	5%	11%	24%	36%

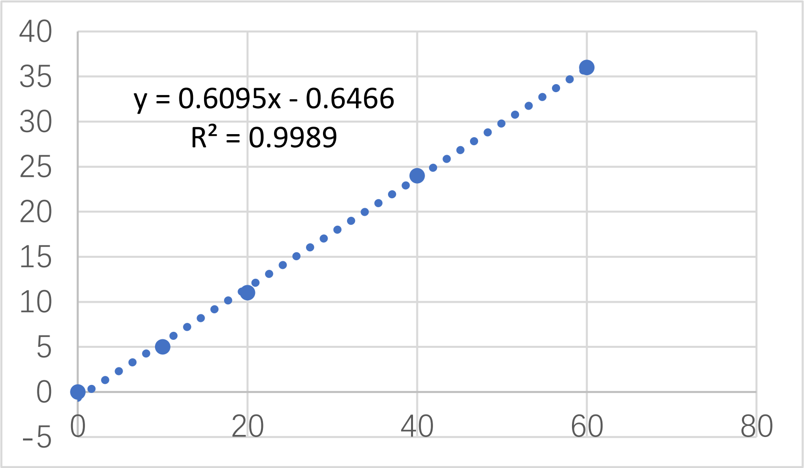

Fig. 35 Reaction rate testing for **3ai-d**

The KIE is determined to 1.1/1 (k_H/k_D = 0.6552/0.6095) in the electrochemical CDC.

9. Photophysical property investigation (4)

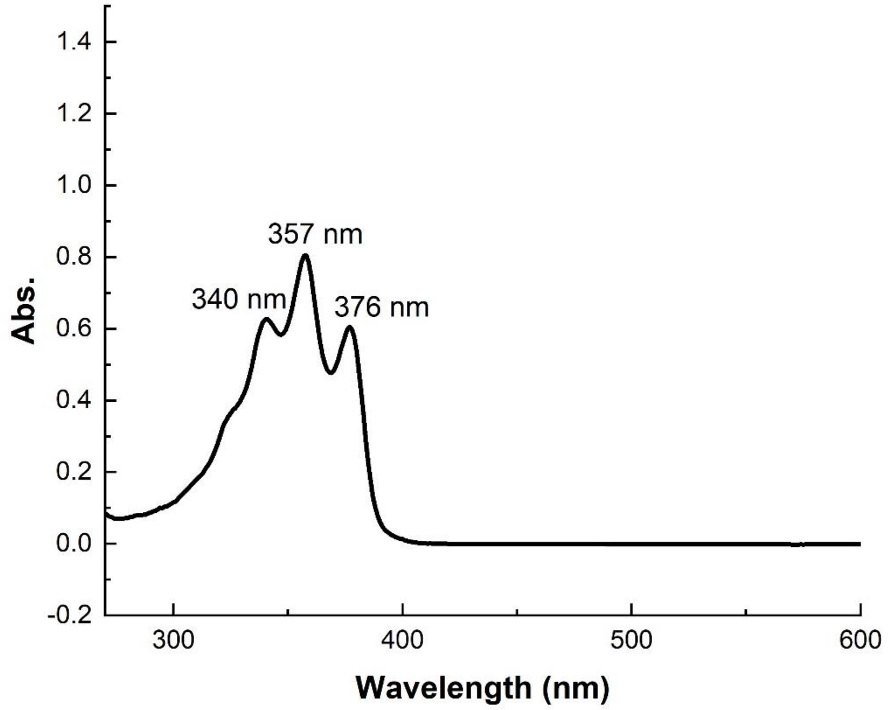

Fig. 36 UV-vis spectra of the solution **4** ($1*10^{-5}$ M) in DCM

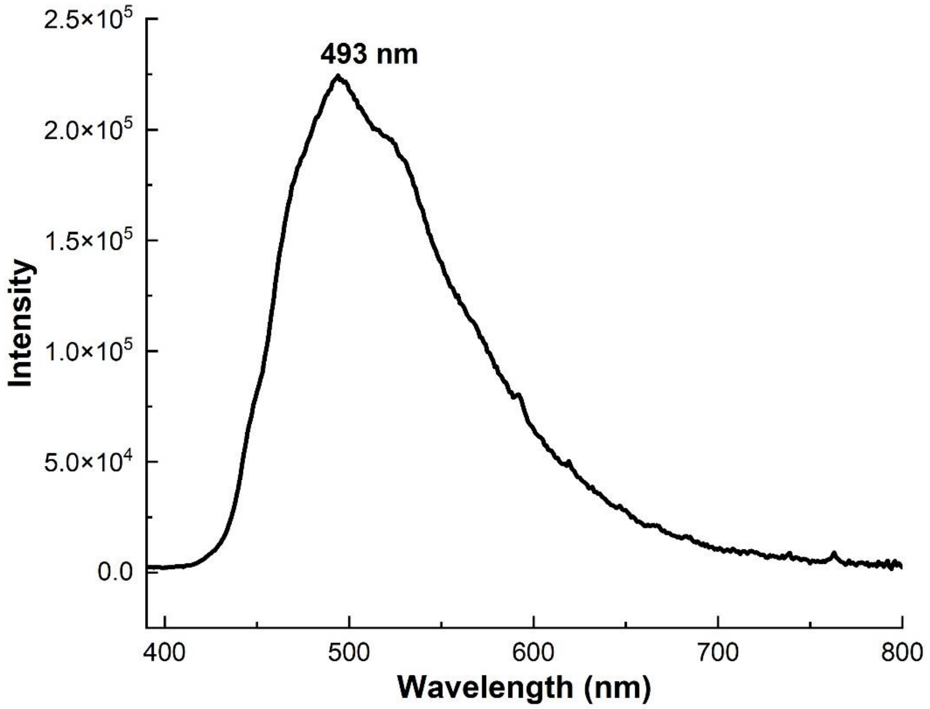

Fig. 37 Photoluminescence spectroscopy of **4** in solid state under 350 nm irritation

10. Experimental data

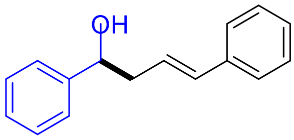

(*E*)-1,4-Diphenylbut-3-en-1-ol (**3a, 3j**): **3a** 85% yield, 95 mg; **3j** 54% yield, 60 mg; white solid; ^{1}H NMR (400 MHz, $CDCl_3$) δ 7.33 (m, 9H), 7.21 (m, 1H), 6.50 (d, *J* = 20.0 Hz, 1H), 6.20 (m, 1H), 4.80 (t, *J* = 8.0 Hz, 1H), 2.66 (t, *J* = 8.0 Hz, 2H), 2.09 (br, 1H); ^{13}C NMR (101 MHz, $CDCl_3$) δ 143.9, 137.2, 133.4, 128.50, 128.45, 127.6, 127.3, 126.1, 125.9, 125.8, 73.7, 43.1. These data are in accordance with the literature.[5]

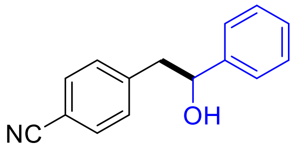

4-(2-Hydroxy-2-phenylethyl)benzonitrile (**3b**): 65% yield, 73 mg; colorless oil; ^{1}H NMR (400 MHz, $CDCl_3$) δ 7.55 (d, *J* = 8.0 Hz, 2H), 7.30 (m, 7H), 4.91 (t, *J* = 8.0 Hz, 1H), 3.08 (m, 2H), 2.03 (br, 1H); ^{13}C NMR (101 MHz, $CDCl_3$) δ 143.8, 143.3, 132.0, 130.4, 128.6, 128.0, 125.8, 119.0, 110.3, 74.9, 45.7. These data are in accordance with the literature.[6]

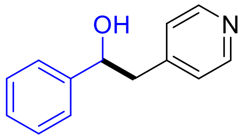

1-Phenyl-2-(pyridin-4-yl)ethan-1-ol (**3c**): 60% yield, 60 mg; white solid; ^{1}H NMR (400 MHz, $CDCl_3$) δ 8.28 (s, 2H), 7.29 (m, 5H), 7.05 (d, *J* = 8.0 Hz, 2H), 4.89 (q, *J* = 4.0 Hz, 1H), 3.88 (br, 1H), 2.97 (m, 2H); ^{13}C NMR (101 MHz, $CDCl_3$) δ 149.0, 147.8, 143.8, 128.4, 127.6, 125.8, 125.0, 74.1, 45.1. These data are in accordance with the literature.[5]

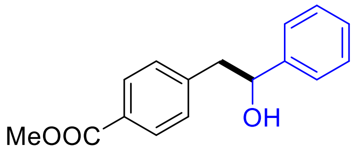

Methyl 4-(2-hydroxy-2-phenylethyl)benzoate (**3d**): 47% yield, 60 mg; yellow solid; ^{1}H NMR (400 MHz, $CDCl_3$) δ 7.94 (d, *J* = 8.0 Hz, 2H), 7.32 (m, 5H), 7.23 (d, *J* = 8.0 Hz, 2H), 4.91 (m, 1H), 3.89 (s, 3H), 3.05 (m, 2H), 2.08 (br, 1H); ^{13}C NMR (101 MHz, $CDCl_3$) δ 167.0, 143.52, 143.48, 129.63, 129.55, 128.5, 128.4, 127.8, 125.8, 75.1, 52.0, 45.8. These data are in accordance with the literature.[6]

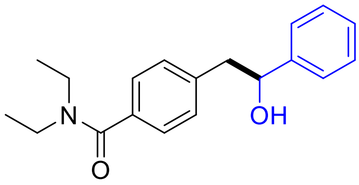

N,*N*-Diethyl-4-(2-hydroxy-2-phenylethyl)benzamide (**3e**): 48% yield, 71 mg; pale yellow oil; ^{1}H NMR (400 MHz, $CDCl_3$) δ 7.32 (d, *J* = 4.0 Hz, 4H), 7.27 (d, *J* = 8.0 Hz, 3H), 7.17 (d, *J* = 8.0 Hz, 2H), 4.87 (t, *J* = 6.0 Hz, 1H), 3.52 (s, 2H), 3.25 (s,2H), 3.02 (d, *J* = 8.0 Hz, 2H), 2.43 (s, 1H), 1.22 (s, 3H), 1.10 (s, 3H); ^{13}C NMR (101 MHz, $CDCl_3$) δ 171.3, 143.7, 139.3, 135.3, 129.5, 128.3, 127.6, 126.4, 125.9, 75.1, 45.7, 43.2, 39.2, 14.1, 12.9; HRMS (ESI): calcd for $C_{19}H_{23}NO_2$ $(M+H)^+$ 298.1802, found 298.1802.

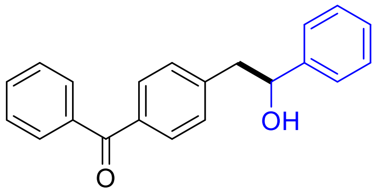

(4-(2-Hydroxy-2-phenylethyl)phenyl)(phenyl)methanone (**3f**): 31% yield, 47 mg; white solid; ^{1}H NMR (400 MHz, $CDCl_3$) δ 7.78 (d, *J* = 8.0 Hz, 2H), 7.74 (d, *J* = 8.0 Hz, 2H), 7.58 (t, *J* = 8.0 Hz, 1H), 7.47 (t, *J* = 8.0 Hz, 2H), 7.36 (d, *J* = 4.0 Hz, 4H), 7.29 (d, *J* = 8.0 Hz, 3H), 4.96 (t, *J* = 6.0 Hz, 1H), 3.11 (d, *J* = 8.0 Hz, 2H), 2.04 (br, 1H); ^{13}C NMR (101 MHz, $CDCl_3$) δ 196.5, 143.6, 143.2, 137.7, 135.8, 132.3, 130.3, 130.0, 129.5, 128.5, 128.2, 127.9, 125.9, 75.1, 45.8. These data are in accordance with the literature.[7]

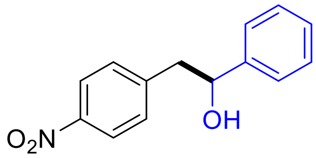

2-(4-Nitrophenyl)-1-phenylethan-1-ol (**3g**): 46% yield, 56 mg; yellow solid; ^{1}H NMR (400 MHz, CD_3CN) δ 8.18 (t, *J* = 6.0 Hz, 2H), 7.42 (m, 7H), 4.99 (t, *J* = 8.0 Hz, 1H), 3.16 (m, 2H), 2.03 (br, 1H); ^{13}C NMR (101 MHz, CD_3CN) δ 148.0, 147.3, 145.3, 131.3, 128.9, 128.0, 126.6, 123.7, 74.5, 45.8. These data are in accordance with the literature.[8]

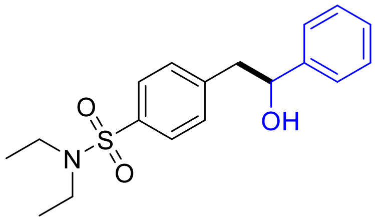

N,*N*-Diethyl-4-(2-hydroxy-2-phenylethyl)benzenesulfonamide (**3h**): 51% yield, 85 mg; pale yellow solid; ^{1}H NMR (400 MHz, $CDCl_3$) δ 7.66 (d, *J* = 8.0 Hz, 2H), 7.29 (m, 7H), 4.89 (t, *J* = 6.0 Hz, 1H), 3.20 (q, *J* = 8.0 Hz, 4H), 3.06 (m, 2H), 2.20 (br, 1H), 1.09 (t, *J* = 8.0 Hz, 6H); ^{13}C NMR (101 MHz, $CDCl_3$) δ 143.3, 143.0, 138.3, 130.1, 128.4, 127.8, 126.9, 125.8, 74.9, 45.5, 41.9, 14.0. These data are in accordance with the literature.[9]

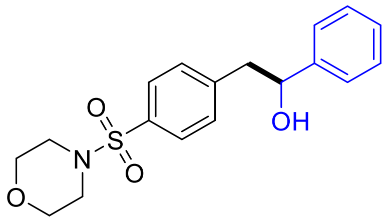

2-(4-(Morpholinosulfonyl)phenyl)-1-phenylethan-1-ol (**3i**): 58% yield, 101 mg; white solid, m.p. 157-159 °C; ^{1}H NMR (400 MHz, $CDCl_3$) δ 7.63 (d, *J* = 8.0 Hz, 2H), 7.31 (m, 7H), 4.93 (t, *J* = 8.0 Hz, 1H), 3.72 (t, *J* = 4.0 Hz, 4H), 3.10 (m, 2H), 2.97 (t, *J* = 6.0 Hz, 4H), 2.10 (br, 1H); ^{13}C NMR (101 MHz, $CDCl_3$) δ 144.0, 143.3, 133.0, 130.3, 128.5, 128.0, 127.8, 125.8, 74.9, 66.1, 46.0, 45.5; HRMS (ESI): calcd for $C_{18}H_{21}NO_4S$ $(M+H)^+$ 348.1264, found 348.1263.

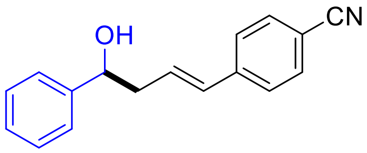

(*E*)-4-(4-Hydroxy-4-phenylbut-1-en-1-yl)benzonitrile (**3k, 3ad**): **3k** 75% yield, 93 mg; **3ad** 65% yield, 81 mg; white solid; ^{1}H NMR (400 MHz, $CDCl_3$) δ 7.56 (d, *J* = 8.0 Hz, 2H), 7.38 (m, 6H), 7.30 (m, 1H), 6.48 (d, *J* = 16.0 Hz, 1H), 6.36 (m, 1H), 4.84 (t, *J* = 8.0 Hz, 1H), 2.70 (m, 2H), 2.03 (br, 1H); ^{13}C NMR (101 MHz, $CDCl_3$) δ 143.7, 141.7, 132.3, 131.5, 130.5, 128.6, 127.8, 126.6, 125.7, 119.0, 110.4, 73.7, 42.8. These data are in accordance with the literature.[5]

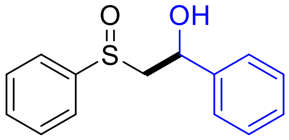

1-Phenyl-2-(phenylsulfinyl)ethan-1-ol (**3l**): 21% yield, 26 mg; *dr* 1/1; colorless oil; ^{1}H NMR (400 MHz, $CDCl_3$) δ 7.67 (m, 2H), 7.55 (m, 3H), 7.33 (m, 5H), 5.40 (dd, *J* = 4.0 Hz, *J* = 12.0 Hz, 0.6H), 5.27 (dd, *J* = 4.0 Hz, *J* = 12.0 Hz, 0.5H), 4.34 (s, 1H), 3.25 (m, 1H), 2.96 (dd, *J* = 4.0 Hz, *J* = 12.0 Hz, 0.6H), 2.86 (dd, *J* = 4.0 Hz, *J* = 12.0 Hz, 0.5H); ^{13}C NMR (101 MHz, $CDCl_3$) δ 143.6, 142.7, 142.0, 131.5, 131.2, 129.5, 129.4, 128.7, 128.6, 128.2, 128.0, 125.7, 125.6, 124.0, 123.8, 71.3, 68.9, 64.2, 63.4. These data are in accordance with the literature.[5]

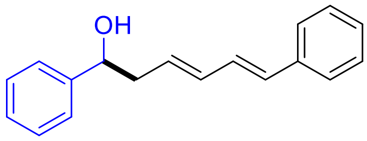

(3*E*,5*E*)-1,6-Diphenylhexa-3,5-dien-1-ol (**3m**): 66% yield, 83 mg; pale yellow solid; ^{1}H NMR (400 MHz, $CDCl_3$) δ 7.36 (m, 6H), 7.30 (t, *J* = 6.0 Hz, 3H), 7.20 (t, *J* = 8.0 Hz, 1H), 6.75 (dd, *J* = 16.0 Hz, *J* = 12.0 Hz, 1H), 6.48 (d, *J* = 16.0 Hz, 1H), 6.32 (dd, *J* = 16.0, *J* = 12.0 Hz, 1H), 5.78 (m, 1H), 4.76 (t, *J* = 8.0 Hz, 1H), 2.60 (t, *J* = 8.0 Hz, 2H), 2.07 (br, 1H); ^{13}C NMR (101 MHz, $CDCl_3$) δ 143.8, 137.3, 134.0, 131.4, 130.2, 128.7, 128.6, 128.4, 127.6, 127.4, 126.2, 125.8, 73.70, 42.9. These data are in accordance with the literature.[10]

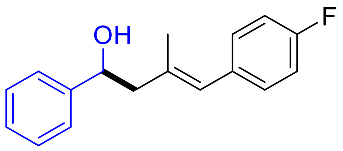

(*E*)-4-(4-Fluorophenyl)-3-methyl-1-phenylbut-3-en-1-ol (**3n**): 36% yield, 46 mg; colorless oil; ^{1}H NMR (400 MHz, $CDCl_3$) δ 7.32 (m, 5H), 7.18(t, *J* = 6.0 Hz, 2H), 6.96 (t, *J* = 8.0 Hz, 2H), 6.42 (s, 1H), 4.91 (m, 1H), 2.85 (m, 1H), 2.41 (m, 1H), 1.95 (s, 4H); ^{13}C NMR (101 MHz, $CDCl_3$) δ 161.4 (d, $J_{F\text{-}C}$ = 246.4 Hz), 144.1, 135.2, 133.8 (d, $J_{F\text{-}C}$ = 4.0 Hz), 130.3 (d, $J_{F\text{-}C}$ = 7.1 Hz), 128.4, 128.1, 127.6, 125.7 (d, $J_{F\text{-}C}$ = 5.1 Hz), 114.9 (d, $J_{F\text{-}C}$ = 21.2 Hz), 72.3, 42.2, 24.0. These data are in accordance with the literature.[5]

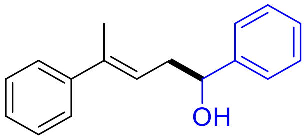

(*E*)-1,4-Diphenylpent-3-en-1-ol (**3o**): 66% yield, 79 mg; pale yellow oil; ^{1}H NMR (400 MHz, $CDCl_3$) δ 7.33 (m, 9H), 7.21 (t, *J* = 6.0 Hz, 1H), 5.78 (m, 1H), 4.78 (m, 1H), 2.65 (m, 2H), 2.16 (br, 1H), 1.97 (s, 3H); ^{13}C NMR (101 MHz, $CDCl_3$) δ 144.1, 143.5, 137.9, 128.4, 128.1, 127.5, 126.8, 125.8, 125.6, 123.3, 74.1, 38.7, 16.0; These data are in accordance with the literature.[5]

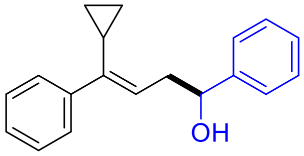

(*E*)-4-Cyclopropyl-1,4-diphenylbut-3-en-1-ol (**3p**): 64% yield, 84 mg; pale yellow oil; ^{1}H NMR (400 MHz, $CDCl_3$) δ 7.40 (d, *J* = 8.0 Hz, 2H), 7.35 (t, *J* = 8.0 Hz, 2H), 7.27 (m, 5H), 7.19 (m, 1H), 5.70 (t, *J* = 8.0 Hz, 1H), 4.82 (t, *J* = 6.0 Hz, 1H), 2.90 (m, 1H), 2.79 (m, 1H), 2.11 (br, 1H), 1.66 (m, 1H), 0.75 (m, 2H), 0.25 (m, 2H); ^{13}C NMR (101 MHz, $CDCl_3$) δ 144.2, 143.8, 142.1, 128.4, 127.7, 127.5, 127.4, 126.5, 126.4, 125.9, 74.2, 38.4, 11.6, 6.59, 6.56; These data are in accordance with the literature.[5]

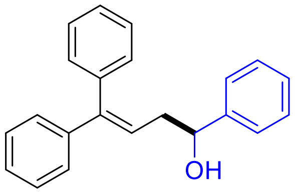

1,4,4-Triphenylbut-3-en-1-ol (**3q**): 91% yield, 137 mg; colorless oil; ^{1}H NMR (400 MHz, $CDCl_3$) δ 7.29 (m, 7H), 7.20 (m, 6H), 7.05 (d, *J* = 8.0 Hz, 2H), 6.10 (t, *J* = 8.0 Hz, 1H), 4.75 (t, *J* = 6.0 Hz, 1H), 2.57 (m, 2H), 2.12 (br, 1H); ^{13}C NMR (101 MHz, $CDCl_3$) δ 144.1, 143.9, 142.3, 139.7, 129.8, 128.3, 128.1, 128.0, 127.5, 127.2, 127.02, 126.97, 125.8, 124.7, 74.3, 39.4; These data are in accordance with the literature.[5]

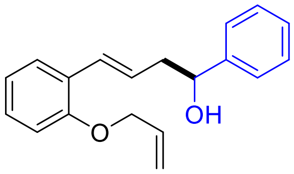

(*E*)-4-(2-(Allyloxy)phenyl)-1-phenylbut-3-en-1-ol (**3r**): 46% yield, 64 mg; colorless oil; ^{1}H NMR (400 MHz, $CDCl_3$) δ 7.36 (m, 5H), 7.27 (t, *J* = 8.0 Hz, 1H), 7.16 (t, *J* = 8.0 Hz, 1H), 6.87 (m, 3H), 6.19 (m, 1H), 6.05 (m, 1H), 5.39 (d, *J* = 16.0 Hz, 1H), 5.27 (d, *J* = 12.0 Hz, 1H), 4.78 (t, *J* = 6.0 Hz, 1H), 4.54 (d, *J* = 4.0 Hz, 2H), 2.67 (m, 2H), 2.19 (br, 1H); ^{13}C NMR (101 MHz, $CDCl_3$) δ 155.4, 144.0, 133.4, 128.4, 128.2, 127.5, 126.7, 126.6, 126.5, 125.8, 120.8, 117.3, 112.3, 73.7, 69.1, 43.5; These data are in accordance with the literature.[5]

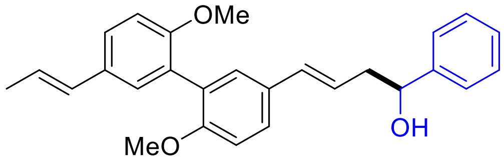

(*E*)-4-(2',6-Dimethoxy-5'-((*E*)-prop-1-en-1-yl)-[1,1'-biphenyl]-3-yl)-1-phenylbut-3-en-1-ol (**3s**): 33% yield, 66 mg; brown oil; ^{1}H NMR (400 MHz, $CDCl_3$) δ 7.34 (m, 8H), 7.21 (s, 1H), 6.89 (d, *J* = 8.0 Hz, 2H), 6.47 (d, *J* = 16.0 Hz, 1H), 6.36 (d, *J* = 16.0 Hz, 1H), 6.08 (m, 2H), 4.77 (t, *J* = 6.0 Hz, 1H), 3.75 (s, 6H), 2.62 (m, 2H), 2.11 (br, 1H), 1.85 (d, *J* = 8.0 Hz, 3H); ^{13}C NMR (101 MHz, $CDCl_3$) δ 156.7, 156.1, 143.9, 133.1, 130.5, 130.3, 129.7, 129.1, 128.7, 128.5, 128.4, 128.1, 127.7, 127.5, 127.0, 126.6, 126.2, 125.8, 123.61, 123.58, 111.1, 73.7, 55.8, 43.2, 18.4; HRMS (ESI): calcd for $C_{27}H_{28}O_3$ $(M+H)^+$ 401.2111, found 401.2110.

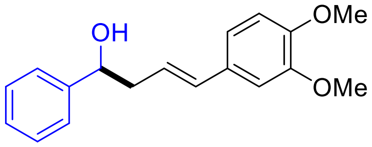

(*E*)-4-(3,4-Dimethoxyphenyl)-1-phenylbut-3-en-1-ol (**3t**): 51% yield, 73 mg; colorless oil; ^{1}H NMR (400 MHz, $CDCl_3$) δ 7.36 (m, 4H), 7.28 (t, *J* = 8.0 Hz, 1H), 6.88 (d, *J* = 12.0 Hz, 2H), 6.79 (d, *J* = 8.0 Hz, 1H), 6.43 (d, *J* = 16.0 Hz, 1H), 6.08 (m, 1H), 4.78 (t, *J* = 6.0 Hz, 1H), 3.87 (s, 3H), 3.85 (s, 3H), 2.63 (m, 2H), 2.25 (br, 1H); ^{13}C NMR (101 MHz, $CDCl_3$) δ 148.9, 148.5, 143.9, 133.0, 130.3, 128.4, 127.5, 125.7, 123.9, 119.1, 111.0, 108.6, 73.7, 55.8, 55.7, 43.0; These data are in accordance with the literature.[5]

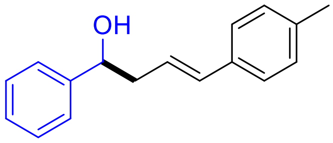

(*E*)-1-Phenyl-4-(*p*-tolyl)but-3-en-1-ol (**3u**): 52% yield, 62 mg; white solid; ^{1}H NMR (400 MHz, $CDCl_3$) δ 7.36 (m, 4H), 7.28 (m, 1H), 7.23 (d, *J* = 8.0 Hz, 2H), 7.09 (d, *J* = 8.0 Hz, 2H), 6.46 (d, *J* = 16.0 Hz, 1H), 6.13 (m, 1H), 4.77 (t, *J* = 6.0 Hz, 1H), 2.63 (m, 2H), 2.32 (s, 3H), 2.15 (br, 1H); ^{13}C NMR (101 MHz, $CDCl_3$) δ 143.9, 137.1, 134.4, 133.3, 129.2, 128.4, 127.5, 126.0, 125.8, 124.7, 73.7, 43.1, 21.1; These data are in accordance with the literature.[5]

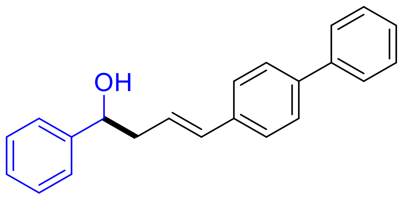

(*E*)-4-([1,1'-Biphenyl]-4-yl)-1-phenylbut-3-en-1-ol (**3v**): 88% yield, 132 mg; white solid; ^{1}H NMR (400 MHz, $CDCl_3$) δ 7.57 (m, 4H), 7.37 (m, 10H), 6.54 (d, *J* = 16.0 Hz, 1H), 6.25 (m, 1H), 4.84 (t, *J* = 6.0 Hz, 1H), 2.68 (m, 2H), 2.07 (br, 1H); ^{13}C NMR (101 MHz, $CDCl_3$) δ 143.9, 140.7, 140.1, 136.2, 133.0, 128.8, 128.5, 127.6, 127.24, 127.21, 126.9, 126.6, 126.0, 125.8, 73.8, 43.1. These data are in accordance with the literature.[5]

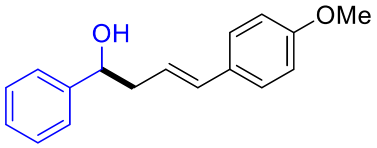

(*E*)-4-(4-Methoxyphenyl)-1-phenylbut-3-en-1-ol (**3w**): 67% yield, 85 mg; white solid; ^{1}H NMR (400 MHz, $CDCl_3$) δ 7.36 (m, 4H), 7.27 (m, 3H), 6.83 (d, *J* = 8.0 Hz, 2H), 6.44 (d, *J* = 16.0 Hz, 1H), 6.05 (m, 1H), 4.78 (t, *J* = 6.0 Hz, 1H), 3.79 (s, 3H), 2.65 (m, 2H), 2.16 (br, 1H); ^{13}C NMR (101 MHz, $CDCl_3$) δ 159.0, 143.9, 132.9, 130.0, 128.4, 127.5, 127.3, 125.8, 123.5, 113.9, 73.7, 55.2, 43.1; These data are in accordance with the literature.[5]

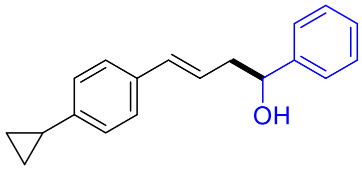

(*E*)-4-(4-Cyclopropylphenyl)-1-phenylbut-3-en-1-ol (**3x**): 67% yield, 89 mg; white solid; ^{1}H NMR (400 MHz, $CDCl_3$) δ 7.35 (m, 4H), 7.27 (t, *J* = 6.0 Hz, 1H), 7.22 (d, *J* = 8.0 Hz, 2H), 6.99 (d, *J* = 8.0 Hz, 2H), 6.45 (d, *J* = 16.0 Hz, 1H), 6.12 (m, 1H), 4.77 (t, *J* = 8.0 Hz, 1H), 2.63 (t, *J* = 8.0 Hz, 2H), 2.14 (s, 1H), 1.86 (m, 1H), 0.94 (dd, *J* = 4.0 Hz, *J* = 12.0 Hz, 2H), 0.67 (m, 2H); ^{13}C NMR (101 MHz, $CDCl_3$) δ 143.9, 143.3, 134.4, 133.2, 128.4, 127.5, 126.1, 125.8, 125.7, 124.6, 73.7, 43.1, 15.2, 9.3; HRMS (ESI): calcd for $C_{19}H_{20}O$ $(M+H)^+$ 247.1481, found 247.1484. These data are in accordance with the literature.[5]

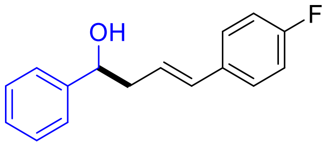

(*E*)-4-(4-Fluorophenyl)-1-phenylbut-3-en-1-ol (**3y**): 68% yield, 82 mg; white solid; ^{1}H NMR (400 MHz, $CDCl_3$) δ 7.36 (m, 4H), 7.28 (m, 3H), 6.97 (t, *J* = 8.0 Hz, 2H), 6.44 (d, *J* = 16.0 Hz, 1H), 6.10 (m, 1H), 4.78 (t, *J* = 6.0 Hz, 1H), 2.63 (t, *J* = 6.0 Hz, 2H), 2.17 (br, 1H); ^{13}C NMR (101 MHz, $CDCl_3$) δ 162.1 (d, $J_{F\text{-}C}$ = 247.5 Hz), 143.8, 133.3 (d, $J_{F\text{-}C}$ = 3.0 Hz), 132.1, 128.5, 127.6, 127.5, 125.8, 125.7 (d, $J_{F\text{-}C}$ = 2.0 Hz) , 115.3 (d, $J_{F\text{-}C}$ = 21.2 Hz), 73.7, 42.9; These data are in accordance with the literature.[5]

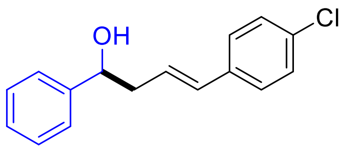

(*E*)-4-(4-Chlorophenyl)-1-phenylbut-3-en-1-ol (**3z**): 62% yield, 80 mg; white solid; ^{1}H NMR (400 MHz, $CDCl_3$) δ 7.36 (m, 4H), 7.28 (m, 5H), 6.43 (d, *J* = 16.0 Hz, 1H), 6.18 (m, 1H), 4.81 (t, *J* = 6.0 Hz, 1H), 2.65 (t, *J* = 8.0 Hz, 2H), 2.06 (br, 1H); ^{13}C NMR (101 MHz, $CDCl_3$) δ 143.8, 135.7, 132.9, 132.0, 128.6, 128.5, 127.7, 127.3, 126.7, 125.8, 73.8, 42.9. These data are in accordance with the literature.[5]

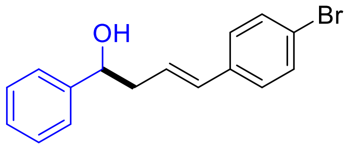

(*E*)-4-(4-Bromophenyl)-1-phenylbut-3-en-1-ol (**3aa**): 75% yield, 114 mg; white solid; ^{1}H NMR (400 MHz, $CDCl_3$) δ 7.34 (m, 7H), 7.19 (d, *J* = 12.0 Hz, 2H), 6.41 (d, *J* = 16.0 Hz, 1H), 6.19 (m, 1H), 4.80 (t, *J* = 6.0 Hz, 1H), 2.64 (t, *J* = 8.0 Hz, 2H), 2.09 (br, 1H); ^{13}C NMR (101 MHz, $CDCl_3$) δ 143.8, 136.1, 132.1, 131.6, 128.5, 127.7, 126.9, 125.9, 125.8, 121.0, 73.7, 42.9; These data are in accordance with the literature.[5]

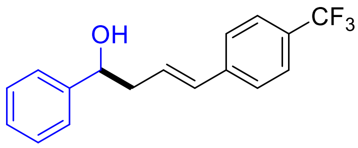

(*E*)-1-Phenyl-4-(4-(trifluoromethyl)phenyl)but-3-en-1-ol (**3ab**): 53% yield, 77mg; colorless oil; ^{1}H NMR (400 MHz, $CDCl_3$) δ 7.53 (d, *J* = 8.0 Hz, 2H), 7.38 (m, 6H), 7.30 (m, 1H), 6.50 (d, *J* = 16.0 Hz, 1H), 6.31 (m, 1H), 4.83 (t, *J* = 8.0 Hz, 1H), 2.69 (t, *J* = 6.0 Hz, 2H), 2.08 (br, 1H); ^{13}C NMR (101 MHz, $CDCl_3$) δ 143.7, 140.7, 131.9, 129.0 (q, $J_{F\text{-}C}$ = 32.3Hz), 128.9, 128.5, 127.8, 126.2, 125.8, 125.5 (q, $J_{F\text{-}C}$ = 4.0Hz), 124.2 (q, $J_{F\text{-}C}$ = 273.7 Hz), 73.8, 42.9. These data are in accordance with the literature.[5]

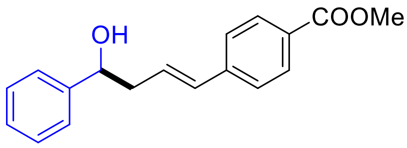

Methyl (*E*)-4-(4-hydroxy-4-phenylbut-1-en-1-yl)benzoate (**3ac**): 60% yield, 85 mg; white solid, m.p. 86-88 °C;^{1}H NMR (400 MHz, $CDCl_3$) δ 7.95 (d, *J* = 8.0 Hz, 2H), 7.34 (m, 7H), 6.51 (d, *J* = 16.0 Hz, 1H), 6.34 (m, 1H), 4.83 (t, *J* = 6.0 Hz, 1H), 3.90 (s, 3H), 2.68 (t, *J* = 6.0 Hz, 2H), 2.17 (br, 1H); ^{13}C NMR (101 MHz, $CDCl_3$) δ 166.9, 143.8, 141.7, 132.3, 129.9, 129.1, 128.7, 128.5, 127.7, 126.0, 125.8, 73.7, 52.0, 43.0; HRMS (ESI): calcd for $C_{18}H_{18}O_3$ $(M+H)^+$ 283.1329, found 283.1328.

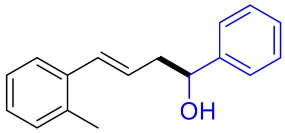

(*E*)-1-Phenyl-4-(*o*-tolyl)but-3-en-1-ol (**3ae**): 66% yield, 79 mg; white solid; ^{1}H NMR (400 MHz, $CDCl_3$) δ 7.36 (m, 5H), 7.27 (t, *J* = 8.0 Hz, 1H), 7.13 (m, 3H), 6.65 (d, *J* = 16.0 Hz, 1H), 6.04 (m, 1H), 4.79 (t, *J* = 6.0 Hz, 1H), 2.68 (t, *J* = 6.0 Hz, 2H), 2.28 (s, 3H), 2.15 (br, 1H); ^{13}C NMR (101 MHz, $CDCl_3$) δ 143.8, 136.3, 135.1, 131.3, 130.2, 128.4, 127.6, 127.2, 127.1, 126.0, 125.8, 125.6, 73.7, 43.2, 19.7. These data are in accordance with the literature.[5]

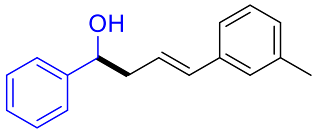

(*E*)-1-Phenyl-4-(*m*-tolyl)but-3-en-1-ol (**3af**): 60% yield, 71 mg; white solid; ^{1}H NMR (400 MHz, $CDCl_3$) δ 7.38 (t, *J* = 12.0 Hz, 4H), 7.30 (t, *J* = 6.0 Hz, 1H), 7.18 (m, 3H), 7.05 (d, *J* = 8.0 Hz, 1H), 6.48 (d, *J* = 16.0 Hz, 1H), 6.20 (m, 1H), 4.80 (t, *J* = 6.0 Hz, 1H), 2.66 (t, *J* = 8.0 Hz, 2H), 2.35 (s, 3H), 2.18 (br, 1H); ^{13}C NMR (101 MHz, $CDCl_3$) δ 143.9, 138.0, 137.1, 133.5, 128.42, 128.39, 128.1, 127.5, 126.9, 125.8, 125.6, 123.3, 73.7, 43.1, 21.3. These data are in accordance with the literature.[5]

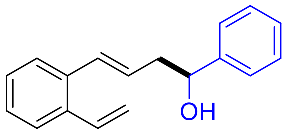

(*E*)-1-Phenyl-4-(2-vinylphenyl)but-3-en-1-ol (**3ag**): 54% yield, 68 mg; colorless oil; ^{1}H NMR (400 MHz, $CDCl_3$) δ 7.38 (m, 5H), 7.29 (m,1H), 7.14 (m, 3H), 6.92 (m, 1H), 6.73 (d, *J* = 16.0 Hz, 1H), 6.04 (m, 1H), 5.58 (d, *J* = 16.0 Hz, 1H), 5.29 (d, *J* = 12.0 Hz, 1H), 4.82 (t, *J* = 6.0 Hz, 1H), 2.70 (t, *J* = 6.0 Hz, 2H), 2.07 (br, 1H); ^{13}C NMR (101 MHz, $CDCl_3$) δ 143.8, 135.8, 135.6, 134.9, 131.2, 128.5, 128.4, 127.7, 127.6, 127.4, 126.5, 126.2, 125.9, 116.1, 73.8, 43.2. These data are in accordance with the literature.[5]

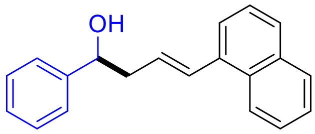

(*E*)-4-(Naphthalen-1-yl)-1-phenylbut-3-en-1-ol (**3ah**): 70% yield, 96 mg; colorless oil; ^{1}H NMR (400 MHz, $CDCl_3$) δ 8.00 (d, *J* = 8.0 Hz, 1H), 7.82 (d, *J* = 12.0 Hz, 1H), 7.73 (d, *J* = 8.0 Hz, 1H), 7.43 (m, 8H), 7.29 (t, *J* = 8.0 Hz, 1H), 7.16 (d, *J* = 16.0 Hz, 1H), 6.18 (m, 1H), 4.85 (t, *J* = 6.0 Hz, 1H), 2.77 (t, *J* = 8.0 Hz, 2H), 2.17 (br, 1H); ^{13}C NMR (101 MHz, $CDCl_3$) δ 143.9, 135.0, 133.5, 131.0, 130.7, 129.1, 128.5, 128.4, 127.7, 127.6, 125.9, 125.7, 125.6, 123.9, 123.7, 73.8, 43.3; These data are in accordance with the literature.[5]

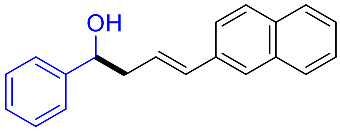

(*E*)-4-(Naphthalen-2-yl)-1-phenylbut-3-en-1-ol (**3ai**): 61% yield, 84 mg; white solid; ^{1}H NMR (400 MHz, $CDCl_3$) δ 7.77 (m, 3H), 7.68 (s, 1H), 7.55 (d, *J* = 8.0 Hz, 1H), 7.41 (m, 6H), 7.29 (t, *J* = 6.0 Hz, 1H), 6.65 (d, *J* = 16.0 Hz, 1H), 6.33 (m, 1H), 4.84 (t, *J* = 8.0 Hz, 1H), 2.71 (t, *J* = 6.0 Hz, 2H), 2.13 (br, 1H); ^{13}C NMR (101 MHz, $CDCl_3$) δ 143.9, 134.6, 133.6, 133.5, 132.8, 128.5, 128.1, 127.9, 127.6, 126.3, 126.2, 125.9, 125.8, 125.7, 123.5, 73.8, 43.2; These data are in accordance with the literature.[5]

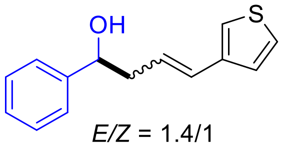

1-Phenyl-4-(thiophen-3-yl)but-3-en-1-ol (**3aj**): 53% yield, 61 mg; white solid; ^{1}H NMR (400 MHz, $CDCl_3$) δ 7.36 (m, 6.4H), 7.28 (m, 3.3 H), 7.17 (s, 1.6H), 7.09 (s, 1.6H), 6.50 (d, *J* = 16.0 Hz, 1.4H) (major isomer), 6.04 (m, 1H) (minor isomer), 5.65 (m, 0.7 H) (minor isomer), 4.80 (m, 1.6 H), 2.88 (m, 0.7 H) (minor isomer), 2.74 (m, 0.7 H) (minor isomer), 2.60 (m, 2H) (major isomer), 2.11 (br, 1.9 H) ; ^{13}C NMR (101 MHz, $CDCl_3$) δ 143.9, 143.9, 139.8, 138.2, 128.5, 128.4, 127.7, 127.60, 127.56, 126.9, 125.90, 125.83, 125.75, 125.70, 125.1, 124.9, 123.1, 121.4, 74.0, 73.7, 43.0, 38.7; These data are in accordance with the literature.[5]

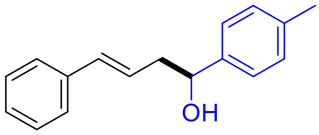

(*E*)-4-Phenyl-1-(*p*-tolyl)but-3-en-1-ol (**3ak**): 48% yield, 57 mg; white solid; ^{1}H NMR (400 MHz, $CDCl_3$): δ 7.29 (m, 6H), 7.19 (m, 3H), 6.48 (d, *J* = 16.0 Hz, 1H), 6.18 (m, 1H), 4.75 (t, *J* = 6.0 Hz, 1H), 2.64 (t, *J* = 8.0 Hz, 2H), 2.34 (s, 3H), 2.08 (br, 1H); ^{13}C NMR (101 MHz, $CDCl_3$) δ 141.0, 137.24, 137.20, 133.2, 129.1, 128.5, 127.2, 126.12, 126.06, 125.7, 73.6, 43.0, 21.1. These data are in accordance with the literature.[5]

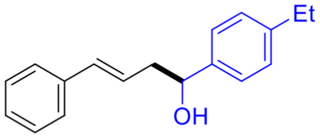

(*E*)-1-(4-Ethylphenyl)-4-phenylbut-3-en-1-ol (**3al**): 74% yield, 93 mg; white solid; ^{1}H NMR (400 MHz, $CDCl_3$) δ 7.31 (m, 6H), 7.20 (t, *J* = 6.0 Hz, 3H), 6.50 (d, *J* = 16.0 Hz, 1H), 6.21 (m, 1H), 4.77 (t, *J* = 8.0 Hz, 1H), 2.65 (m, 4H), 2.06 (br, 1H), 1.24 (t, *J* = 8.0 Hz, 3H); ^{13}C NMR (101 MHz, $CDCl_3$) δ 143.6, 141.2, 137.2, 133.2, 128.5, 127.9, 127.2, 126.12, 126.10, 125.8, 73.6, 43.0, 28.5, 15.6. These data are in accordance with the literature.[5]

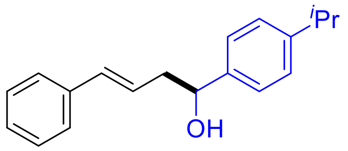

(*E*)-1-(4-Isopropylphenyl)-4-phenylbut-3-en-1-ol (**3am**): 70% yield, 93 mg; white solid; ^{1}H NMR (400 MHz, $CDCl_3$): δ 7.31 (m, 6H), 7.21 (m, 3H), 6.50 (d, *J* = 16.0 Hz, 1H), 6.22 (m, 1H), 4.76 (t, *J* = 6.0 Hz, 1H), 2.90 (m, 1H), 2.65 (t, *J* = 8.0 Hz, 2H), 2.06 (br, 1H), 1.25 (d, *J* = 8.0 Hz, 6H); ^{13}C NMR (101 MHz, $CDCl_3$) δ 148.3, 141.3, 137.3, 133.2, 128.5, 127.2, 126.5, 126.2, 126.1, 125.8, 73.6, 42.9, 33.8, 24.0. These data are in accordance with the literature.[5]

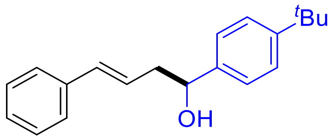

(*E*)-1-(4-(*tert*-Butyl)phenyl)-4-phenylbut-3-en-1-ol (**3an**): 78% yield, 109 mg; white solid; ^{1}H NMR (400 MHz, $CDCl_3$) δ 7.38 (d, *J* = 8.0 Hz, 2H), 7.31 (m, 6H), 7.20 (t, *J* = 8.0 Hz, 1H), 6.50 (d, *J* = 16.0 Hz, 1H), 6.22 (m, 1H), 4.77 (t, *J* = 6.0 Hz, 1H), 2.66 (t, *J* = 6.0 Hz, 2H), 2.07 (br, 1H), 1.32 (s, 9H); ^{13}C NMR (101 MHz, $CDCl_3$) δ 150.5, 140.9, 137.3, 133.2, 128.5, 127.2, 126.2, 126.1, 125.5, 125.4, 73.6, 42.9, 34.5, 31.3. These data are in accordance with the literature.[5]

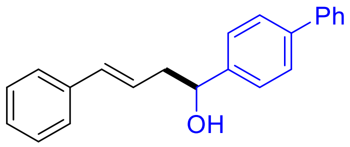

(*E*)-1-([1,1'-Biphenyl]-4-yl)-4-phenylbut-3-en-1-ol (**3ao**): 70% yield, 105 mg; white solid; ^{1}H NMR (400 MHz, $CDCl_3$) δ 7.60 (d, *J* = 8.0 Hz, 4H), 7.45 (m, 4H), 7.31(m, 5H), 7.22 (t, *J* = 8.0 Hz, 1H), 6.53 (d, *J* = 16.0 Hz, 1H), 6.24 (m, 1H), 4.87 (t, *J* = 8.0 Hz, 1H), 2.71 (t, *J* = 8.0 Hz, 2H), 2.09 (br, 1H); ^{13}C NMR (101 MHz, $CDCl_3$) δ 142.9, 140.8, 140.5, 137.2, 133.5, 128.8, 128.5, 127.34, 127.27, 127.2, 127.1, 126.3, 126.2, 125.8, 73.5, 43.0. These data are in accordance with the literature.[5]

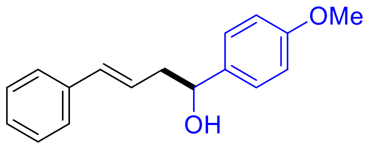

(*E*)-1-(4-Methoxyphenyl)-4-phenylbut-3-en-1-ol (**3ap**): 71% yield, 90 mg; white solid; ^{1}H NMR (400 MHz, $CDCl_3$) δ 7.31 (m, 6H), 7.20 (t, *J* = 8.0 Hz, 1H), 6.89 (d, *J* = 8.0 Hz, 2H), 6.48 (d, *J* = 16.0 Hz, 1H), 6.18 (m, 1H), 4.75 (t, *J* = 6.0 Hz, 1H), 3.80 (s, 3H), 2.64 (t, *J* = 8.0 Hz, 2H), 2.03 (br, 1H); ^{13}C NMR (101 MHz, $CDCl_3$) δ 159.1, 137.2, 136.1, 133.2, 128.5, 127.3, 127.1, 126.12, 126.06, 113.8, 73.4, 55.3, 43.0. These data are in accordance with the literature.[5]

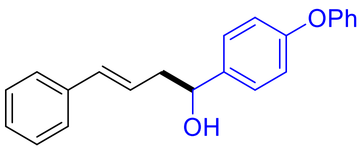

(*E*)-1-(4-Phenoxyphenyl)-4-phenylbut-3-en-1-ol (**3aq**): 58% yield, 92 mg; colorless oil; ^{1}H NMR (400 MHz, $CDCl_3$) δ 7.31 (m, 8H), 7.21 (t, *J* = 8.0 Hz, 1H), 7.10 (t, *J* = 6.0 Hz, 1H), 7.00 (d, *J* = 8.0 Hz, 4H), 6.50 (d, *J* = 16.0 Hz, 1H), 6.20 (m, 1H), 4.79 (t, *J* = 8.0 Hz, 1H), 2.66 (t, *J* = 6.0 Hz, 2H), 2.09 (br, 1H); ^{13}C NMR (101 MHz, $CDCl_3$) δ 157.2, 156.7, 138.7, 137.1, 133.5, 129.7, 128.5, 127.4, 127.3, 126.1, 125.8, 123.2, 118.84, 118.80, 73.3, 43.1. These data are in accordance with the literature.[5]

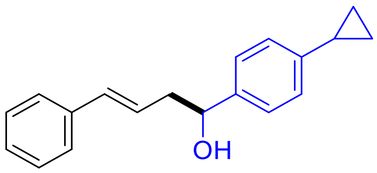

(*E*)-1-(4-Cyclopropylphenyl)-4-phenylbut-3-en-1-ol (**3ar**): 62% yield, 82 mg; white solid; ^{1}H NMR (400 MHz, $CDCl_3$) δ 7.29 (m, 6H), 7.20 (t, *J* = 6.0 Hz, 1H), 7.06 (d, *J* = 12.0 Hz, 2H), 6.48 (d, *J* = 16.0 Hz, 1H), 6.18 (m, 1H), 4.74 (t, *J* = 6.0 Hz, 1H), 2.63 (t, *J* = 8.0 Hz, 2H), 2.06 (br, 1H), 1.88 (m, 1H), 0.95 (m, 2H), 0.68 (m, 2H); ^{13}C NMR (101 MHz, $CDCl_3$) δ 143.4, 141.0, 137.2, 133.2, 128.5, 127.2, 126.12, 126.07, 125.8, 125.7, 73.6, 42.9, 15.1, 9.20, 9.15. These data are in accordance with the literature.[5]

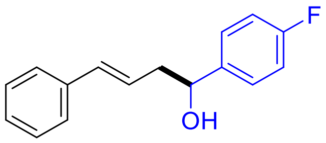

(*E*)-1-(4-Fluorophenyl)-4-phenylbut-3-en-1-ol (**3as**): 63% yield, 76 mg; white solid; ^{1}H NMR (400 MHz, $CDCl_3$) δ 7.32 (m, 6H), 7.22 (m, 1H), 7.03 (t, *J* = 8.0 Hz, 2H), 6.48 (d, *J* = 16.0 Hz, 1H), 6.16 (m, 1H), 4.78 (t, *J* = 6.0 Hz, 1H), 2.62 (m, 2H), 2.15 (br, 1H); ^{13}C NMR (101 MHz, $CDCl_3$) δ 162.2 (d, $J_{F\text{-}C}$ = 246.4 Hz), 139.6 (d, $J_{F\text{-}C}$ = 3.0 Hz), 137.1, 133.6, 128.5, 127.5, 127.4, 126.1, 125.5, 115.2 (d, $J_{F\text{-}C}$ = 21.2 Hz), 73.1, 43.1. These data are in accordance with the literature.[5]

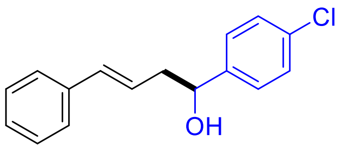

(*E*)-1-(4-Chlorophenyl)-4-phenylbut-3-en-1-ol (**3at**): 60% yield, 78 mg; white solid; ^{1}H NMR (400 MHz, $CDCl_3$) δ 7.31 (m, 8H), 7.22 (t, *J* = 6.0 Hz, 1H), 6.48 (d, *J* = 16.0 Hz, 1H), 6.15 (m, 1H), 4.78 (t, *J* = 8.0 Hz, 1H), 2.63 (m, 2H), 2.13 (br, 1H); ^{13}C NMR (101 MHz, $CDCl_3$) δ 142.3, 137.0, 133.8, 133.2, 128.6, 128.5, 127.4, 127.2, 126.2, 125.3, 73.0, 43.1. These data are in accordance with the literature.[5]

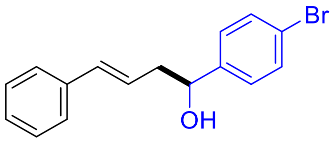

(*E*)-1-(4-Bromophenyl)-4-phenylbut-3-en-1-ol (**3au**): 57% yield, 86 mg; white solid; ^{1}H NMR (400 MHz, $CDCl_3$) δ 7.47 (d, *J* = 8.0 Hz, 2H), 7.28 (m, 7H), 6.48 (d, *J* = 16.0 Hz, 1H), 6.15 (m, 1H), 4.75 (t, *J* = 6.0 Hz, 1H), 2.60 (m, 2H), 2.18 (br, 1H); ^{13}C NMR (101 MHz, $CDCl_3$) δ 142.8, 137.0, 133.8, 131.5, 128.5, 127.5, 127.4, 126.1, 125.2, 121.3, 73.0, 43.0. These data are in accordance with the literature.[5]

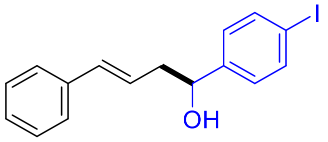

(*E*)-1-(4-Iodophenyl)-4-phenylbut-3-en-1-ol (**3av**): 58% yield, 102 mg; white solid; ^{1}H NMR (400 MHz, $CDCl_3$): δ 7.68 (d, *J* = 8.0 Hz, 2H), 7.32 (m, 4H), 7.22 (m, 1H), 7.12 (d, *J* = 8.0 Hz, 2H), 6.48 (d, *J* = 16.0 Hz, 1H), 6.15 (m, 1H), 4.75 (t, *J* = 6.0 Hz, 1H), 2.60 (m, 2H), 2.15 (br, 1H); ^{13}C NMR (101 MHz, $CDCl_3$) δ 143.5, 137.5, 137.0, 133.8, 128.5, 127.8, 127.4, 126.1, 125.2, 92.9, 73.1, 43.0. These data are in accordance with the literature.[5]

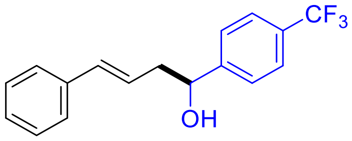

(*E*)-4-Phenyl-1-(4-(trifluoromethyl)phenyl)but-3-en-1-ol (**3aw**): 51% yield, 75 mg; white solid; ^{1}H NMR (400 MHz, $CDCl_3$) δ 7.62 (d, *J* = 8.0 Hz, 2H), 7.50 (d, *J* = 8.0 Hz, 2H), 7.32 (m, 4H), 7.23 (t, *J* = 6.0 Hz, 1H), 6.51 (d, *J* = 16.0 Hz, 1H), 6.17 (m, 1H), 4.88 (q, *J* = 4.0 Hz, 1H), 2.65 (m, 2H), 2.21 (br, 1H); ^{13}C NMR (101 MHz, $CDCl_3$) δ 147.8, 136.9, 134.1, 129.7 (q, $J_{F\text{-}C}$ = 33.3 Hz), 128.6, 127.5, 126.2, 126.1, 125.4 (q, $J_{F\text{-}C}$ = 4.0 Hz), 124.2 (q, $J_{F\text{-}C}$ = 272.7 Hz), 124.9, 73.0, 43.1. These data are in accordance with the literature.[5]

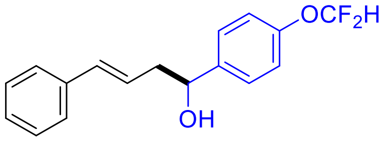

(*E*)-1-(4-(Difluoromethoxy)phenyl)-4-phenylbut-3-en-1-ol (**3ax**): 34% yield, 49 mg; white solid; ^{1}H NMR (400 MHz, $CDCl_3$) δ 7.33 (m, 6H), 7.22 (t, *J* = 8.0 Hz, 1H), 7.11 (d, *J* = 8.0 Hz, 2H), 6.50 (t, $J_{F\text{-}H}$ = 74.0 Hz, 1H), 6.50 (d, *J* = 16.0 Hz, 1H), 6.18 (m, 1H), 4.81 (t, *J* = 6.0 Hz, 1H), 2.64 (m, 2H), 2.13 (br, 1H); ^{13}C NMR (101 MHz, $CDCl_3$) δ 150.5, 141.0, 137.0, 133.8, 128.6, 127.4, 127.3, 126.2, 125.4, 119.5, 115.9 (t, $J_{F\text{-}C}$ = 260.6 Hz), 73.0, 43.1. These data are in accordance with the literature.[5]

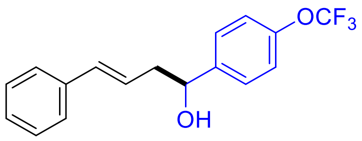

(*E*)-4-Phenyl-1-(4-(trifluoromethoxy)phenyl)but-3-en-1-ol (**3ay**): 50% yield, 77 mg; white solid; ^{1}H NMR (400 MHz, $CDCl_3$): δ 7.40 (d, *J* = 8.0 Hz, 2H), 7.31 (m, 4H), 7.22 (m, 3H), 6.49 (d, *J* = 16.0 Hz, 1H), 6.17 (m, 1H), 4.82 (q, *J* = 4.0 Hz, 1H), 2.63 (m, 2H), 2.16 (br, 1H); ^{13}C NMR (101 MHz, $CDCl_3$) δ 148.5 (q, $J_{F\text{-}C}$ = 2.0 Hz), 142.5, 137.0, 133.9, 128.6, 127.5, 127.2, 126.2, 125.2, 120.9, 120.5 (q, $J_{F\text{-}C}$ = 258.6 Hz), 72.9, 43.1. These data are in accordance with the literature.[5]

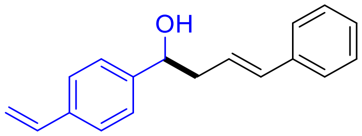

(*E*)-4-Phenyl-1-(4-vinylphenyl)but-3-en-1-ol (**3az**): 51% yield, 64 mg; pale yellow solid, m.p. 110-114 °C; ^{1}H NMR (400 MHz, $CDCl_3$) δ 7.40 (d, *J* = 8.0 Hz, 2H), 7.30 (m, 6H), 7.20 (t, *J* = 6.0 Hz, 1H), 6.71 (m, 1H), 6.49 (d, *J* = 20.0 Hz, 1H), 6.18 (m, 1H), 5.75 (d, *J* = 20.0 Hz, 1H), 5.24 (d, *J* = 8.0 Hz, 1H), 4.79 (t, *J* = 6.0 Hz, 1H), 2.65 (t, *J* = 8.0 Hz, 2H), 2.11 (br, 1H); ^{13}C NMR (101 MHz, $CDCl_3$) δ 143.5, 137.2, 137.0, 136.5, 133.5, 128.5, 127.3, 126.3, 126.1, 126.0, 125.8, 113.8, 73.5, 43.0; HRMS (ESI): calcd for $C_{18}H_{18}O$ $(M+H)^+$ 251.1430, found 251.1436.

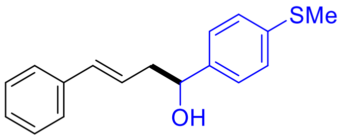

(*E*)-1-(4-(Methylthio)phenyl)-4-phenylbut-3-en-1-ol (**3ba**): 53% yield, 72 mg; white solid; ^{1}H NMR (400 MHz, $CDCl_3$) δ 7.27 (m, 9H), 6.50 (d, *J* = 16.0 Hz, 1H), 6.18 (m, 1H), 4.78(t, *J* = 8.0 Hz, 1H), 2.65 (t, *J* = 6.0 Hz, 2H), 2.49 (s, 3H), 2.03 (br, 1H); ^{13}C NMR (101 MHz, $CDCl_3$) δ 140.8, 137.6, 137.1, 133.5, 128.5, 127.3, 126.7, 126.4, 126.1, 125.7, 73.3, 43.0, 15.9. These data are in accordance with the literature.[5]

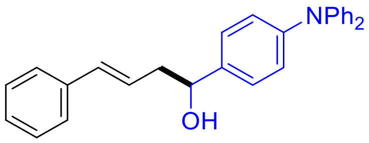

(*E*)-1-(4-(Diphenylamino)phenyl)-4-phenylbut-3-en-1-ol (**3bb**): 54% yield, 106 mg; yellow oil; ^{1}H NMR (400 MHz, $CDCl_3$): δ 7.35 (d, *J* = 12.0 Hz, 2H), 7.29 (t, *J* = 8.0 Hz, 2H), 7.23 (t, *J* = 8.0 Hz, 7H), 7.07 (t, *J* = 8.0 Hz, 6H), 7.00 (t, *J* = 8.0 Hz, 2H), 6.50 (d, *J* = 16.0 Hz, 1H), 6.22 (m, 1H), 4.74 (t, *J* = 6.0 Hz, 1H), 2.65 (t, *J* = 8.0 Hz, 2H), 2.12 (br, 1H); ^{13}C NMR (101 MHz, $CDCl_3$) δ 147.7, 147.2, 138.0, 137.1, 133.3, 129.2, 128.5, 127.3, 126.8, 126.1, 126.0, 124.1, 123.9, 122.7, 73.4, 42.9. These data are in accordance with the literature.[5]

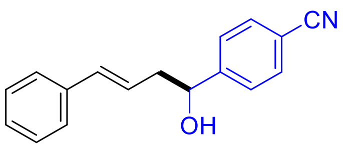

(*E*)-4-(1-Hydroxy-4-phenylbut-3-en-1-yl)benzonitrile (**3bc**): 33% yield, 41 mg; white solid; ^{1}H NMR (400 MHz, $CDCl_3$) δ 7.64 (d, *J* = 8.0 Hz, 2H), 7.50 (d, *J* = 12.0 Hz, 2H), 7.27 (m, 5H), 6.49 (d, *J* = 16.0 Hz, 1H), 6.15 (m, 1H), 4.88 (dd, *J* = 8.0 Hz, *J* = 4.0 Hz, 1H), 2.63 (m, 2H), 2.35 (br, 1H); ^{13}C NMR (101 MHz, $CDCl_3$) δ 149.1, 136.7, 134.4, 132.3, 128.6, 127.6, 126.5, 126.2, 124.5, 118.8, 111.2, 72.8, 43.1. These data are in accordance with the literature.[5]

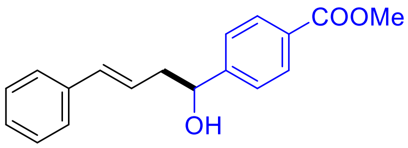

Methyl (*E*)-4-(1-hydroxy-4-phenylbut-3-en-1-yl)benzoate (**3bd**): 30% yield, 42 mg; white solid; ^{1}H NMR (400 MHz, $CDCl_3$) δ 8.02 (d, *J* = 8.0 Hz, 2H), 7.45 (d, *J* = 8.0 Hz, 2H), 7.30 (m, 4H), 7.22 (t, *J* = 10.0 Hz, 1H), 6.49 (d, *J* = 16.0 Hz, 1H), 6.16 (m, 1H), 4.87 (dd, *J* = 8.0 Hz, *J* = 4.0 Hz, 1H), 3.90 (s, 3H), 2.65 (m, 3H); ^{13}C NMR (101 MHz, $CDCl_3$) δ 166.9, 149.0, 136.9, 133.9, 129.8, 129.3, 128.5, 127.4, 126.1, 125.7, 125.1, 73.2, 52.1, 43.0. These data are in accordance with the literature.[5]

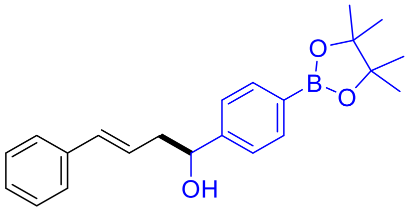

(*E*)-4-Phenyl-1-(4-(4,4,5,5-tetramethyl-1,3,2-dioxaborolan-2-yl)phenyl)but-3-en-1-ol (**3be**): 21% yield, 37 mg; yellow oil; ^{1}H NMR (400 MHz, $CDCl_3$) δ 7.81 (d, *J* = 8.0 Hz, 2H), 7.39 (d, *J* = 8.0 Hz, 2H), 7.29 (m, 4H), 7.20 (t, *J* = 6.0 Hz, 1H), 6.49 (d, *J* = 16.0 Hz, 1H), 6.17 (m, 1H), 4.81 (t, *J* = 6.0 Hz, 1H), 2.65 (m, 2H), 2.16 (br, 1H), 1.34 (s, 12H); ^{13}C NMR (101 MHz, $CDCl_3$) δ 147.0, 137.1, 134.9, 133.5, 128.5, 127.3, 126.1, 125.7, 125.1, 83.8, 73.7, 43.0, 24.8. These data are in accordance with the literature.[5]

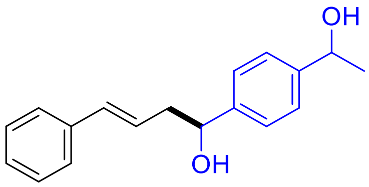

(*E*)-1-(4-(1-Hydroxyethyl)phenyl)-4-phenylbut-3-en-1-ol (**3bf**): 27% yield, 36 mg; white solid, m.p. 110-112 °C; ^{1}H NMR (400 MHz, $CDCl_3$) δ 7.31 (m, 8H), 7.21 (t, *J* = 8.0 Hz, 1H), 6.50 (d, *J* = 16.0 Hz, 1H), 6.19(m, 1H), 4.90 (m, 1H), 4.80 (t, *J* = 6.0 Hz, 1H), 2.65 (t, *J* = 8.0 Hz, 2H), 2.14 (br, 1H), 1.49 (d, *J* = 8.0 Hz, 3H); ^{13}C NMR (101 MHz, $CDCl_3$) δ 145.1, 143.1, 137.1, 133.4, 128.5, 127.3, 126.1, 126.0, 125.8, 125.5, 73.5, 70.1, 43.0, 25.1; HRMS (ESI): calcd for $C_{18}H_{20}O_2$ $(M+H)^+$ 269.1536, found 269.1545.

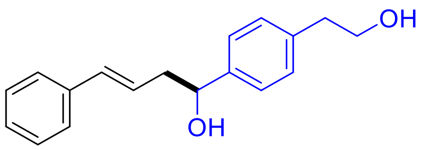

(*E*)-1-(4-(2-Hydroxyethyl)phenyl)-4-phenylbut-3-en-1-ol (**3bg**): 40% yield, 54 mg; pale yellow solid, 117-119 °C; ^{1}H NMR (400 MHz, $CDCl_3$) δ 7.27 (m, 9H), 6.50 (d, *J* = 16.0 Hz, 1H), 6.20 (m, 1H), 4.78 (t, *J* = 8.0 Hz, 1H), 3.85 (t, *J* = 6.0 Hz, 2H), 2.86 (t, *J* = 8.0 Hz, 2H), 2.65 (t, *J* = 6.0 Hz, 2H), 2.21 (s, 2H); ^{13}C NMR (101 MHz, $CDCl_3$) δ 142.1, 137.9, 137.2, 133.4, 129.1, 128.5, 127.3, 126.12, 126.08, 125.9, 73.5, 63.6, 43.0, 38.8; HRMS (ESI): calcd for $C_{18}H_{20}O_2$ $(M+H)^+$ 269.1536, found 269.1543.

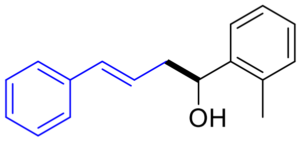

(*E*)-4-Phenyl-1-(*o*-tolyl)but-3-en-1-ol (**3bh**): 42% yield, 50 mg; colorless oil; ^{1}H NMR (400 MHz, $CDCl_3$) δ 7.52 (d, *J* = 8.0 Hz, 1H), 7.35 (d, *J* = 8.0 Hz, 2H), 7.29 (t, *J* = 8.0 Hz, 2H), 7.23 (m, 2H), 7.15 (t, *J* = 8.0 Hz, 2H), 6.51 (d, *J* = 16.0 Hz, 1H), 6.24 (m, 1H), 5.04 (m, 1H), 2.61 (m, 2H), 2.35 (s, 3H), 2.03 (br, 1H); ^{13}C NMR (101 MHz, $CDCl_3$) δ 141.9, 137.2, 134.3, 133.2, 130.4, 128.5, 127.28, 127.26, 126.3, 126.14, 126.12, 125.1, 70.1, 41.9, 19.1; These data are in accordance with the literature.[5]

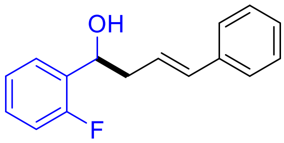

(*E*)-1-(2-Fluorophenyl)-4-phenylbut-3-en-1-ol (**3bi**): 66% yield, 80 mg; yellow solid, m.p. 55-57 °C; ^{1}H NMR (400 MHz, $CDCl_3$) δ 7.49 (t, *J* = 8.0 Hz, 1H), 7.26 (m, 6H), 7.14 (t, *J* = 8.0 Hz, 1H), 7.02 (t, *J* = 10.0 Hz, 1H), 6.48 (d, *J* = 16.0 Hz, 1H), 6.21 (m, 1H), 5.12 (t, *J* = 8.0 Hz, 1H), 2.67 (m, 2H), 2.21 (br, 1H); ^{13}C NMR (101 MHz, $CDCl_3$) δ 159.7 (d, $J_{F\text{-}C}$ = 246.4 Hz), 137.1, 133.6, 130.8 (d, $J_{F\text{-}C}$ = 13.1 Hz), 128.8 (d, $J_{F\text{-}C}$ = 9.1 Hz), 128.5, 127.3, 127.2 (d, $J_{F\text{-}C}$ = 4.0 Hz), 126.2, 125.5, 124.2 (d, $J_{F\text{-}C}$ = 4.0 Hz), 115.2 (d, $J_{F\text{-}C}$ = 21.2 Hz), 67.6, 41.8; HRMS (ESI): calcd for $C_{16}H_{15}FO$ $(M+H)^+$ 243.1180, found 243.1187.

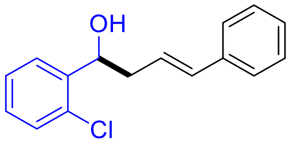

(*E*)-1-(2-Chlorophenyl)-4-phenylbut-3-en-1-ol (**3bj**): 45% yield, 58 mg; white solid; ^{1}H NMR (400 MHz, $CDCl_3$) δ 7.59 (m, 1H), 7.35 (t, *J* = 6.0 Hz, 3H), 7.30 (t, *J* = 6.0 Hz, 3H), 7.21 (m, 2H), 6.51 (d, *J* = 16.0 Hz, 1H), 6.26 (m, 1H), 5.24 (m, 1H), 2.76 (m, 1H), 2.54 (m, 1H), 2.20 (br, 1H); ^{13}C NMR (101 MHz, $CDCl_3$) δ 141.2, 137.2, 133.7, 131.7, 129.4, 128.6, 128.5, 127.4, 127.11, 127.09, 126.2, 125.7, 70.1, 41.3. These data are in accordance with the literature.[5]

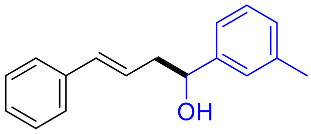

(*E*)-4-Phenyl-1-(*m*-tolyl)but-3-en-1-ol (**3bk**): 70% yield, 83 mg; colorless oil; ^{1}H NMR (400 MHz, $CDCl_3$) δ 7.30 (m, 4H), 7.20 (m, 4H), 7.09 (d, *J* = 8.0 Hz, 1H), 6.49 (d, *J* = 16.0 Hz, 1H), 6.19 (m, 1H), 4.74 (t, *J* = 6.0 Hz, 1H), 2.64 (t, *J* = 6.0 Hz, 2H), 2.36 (s, 3H), 2.12 (br, 1H); ^{13}C NMR (101 MHz, $CDCl_3$) δ 143.9, 138.1, 137.2, 133.3, 128.5, 128.33, 128.31, 127.3, 126.5, 126.1, 126.0, 122.8, 73.7, 43.0, 21.4. These data are in accordance with the literature.[5]

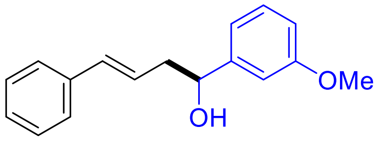

(*E*)-1-(3-Methoxyphenyl)-4-phenylbut-3-en-1-ol (**3bl**): 71% yield, 90 mg; white solid; ^{1}H NMR (400 MHz, $CDCl_3$) δ 7.30 (m, 5H), 7.20 (t, *J* = 8.0 Hz, 1H), 6.94 (m, 2H), 6.82 (d, *J* = 12.0 Hz, 1H), 6.48 (d, *J* = 16.0 Hz, 1H), 6.19 (m, 1H), 4.76 (t, *J* = 8.0 Hz, 1H), 3.79 (s, 3H), 2.63 (m, 2H), 2.20 (br, 1H); ^{13}C NMR (101 MHz, $CDCl_3$) δ 159.7, 145.6, 137.2, 133.3, 129.4, 128.5, 127.3, 126.1, 125.9, 118.1, 113.1, 111.2, 73.6, 55.2, 43.0. These data are in accordance with the literature.[11]

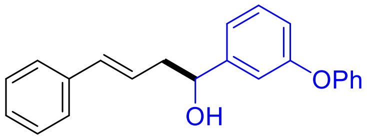

(*E*)-1-(3-Phenoxyphenyl)-4-phenylbut-3-en-1-ol (**3bm**): 52% yield, 82 mg; colorless oil; ^{1}H NMR (400 MHz, $CDCl_3$) δ 7.31 (m, 7H), 7.22 (m, 1H), 7.09 (m, 3H), 6.99 (d, *J* = 8.0 Hz, 2H), 6.93(dd, *J* = 8.0 Hz, *J* = 4.0 Hz, 1H), 6.47 (d, *J* = 16.0 Hz, 1H), 6.17 (m, 1H), 4.78 (t, *J* = 6.0 Hz, 1H), 2.64 (m, 2H), 2.09 (br, 1H); ^{13}C NMR (101 MHz, $CDCl_3$) δ 157.4, 157.1, 146.0, 137.1, 133.6, 129.8, 129.7, 128.5, 127.4, 126.2, 125.5, 123.2, 120.6, 118.9, 117.9, 116.3, 73.3, 43.0. These data are in accordance with the literature.[5]

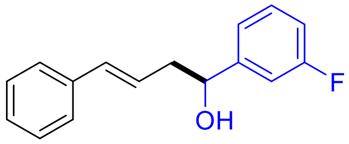

(*E*)-1-(3-Fluorophenyl)-4-phenylbut-3-en-1-ol (**3bn**): 59% yield, 71 mg; yellow solid, m.p. 53-56 °C; ^{1}H NMR (400 MHz, $CDCl_3$): δ 7.31 (m, 5H), 7.21 (m, 1H), 7.12 (m, 2H), 6.96 (m, 1H), 6.49 (d, *J* = 16.0 Hz, 1H), 6.17 (m, 1H), 4.79 (m, 1H), 2.63 (m, 2H), 2.17 (br, 1H); ^{13}C NMR (101 MHz, $CDCl_3$): δ 163.0 (d, $J_{F\text{-}C}$ = 247.5 Hz), 146.6 (d, $J_{F\text{-}C}$ = 7.1 Hz), 137.0, 133.8, 129.9 (d, $J_{F\text{-}C}$ = 8.1 Hz), 128.5, 127.4, 126.2, 125.3, 121.4 (d, $J_{F\text{-}C}$ = 2.0 Hz), 114.4 (d, $J_{F\text{-}C}$ = 21.1 Hz), 112.7 (d, $J_{F\text{-}C}$ = 21.1 Hz), 73.0 (d, $J_{F\text{-}C}$ = 1.0 Hz), 43.0; HRMS (ESI): calcd for $C_{16}H_{15}FO$ $(M+H)^+$ 243.1180, found 243.1182.

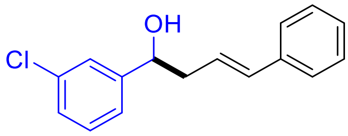

(*E*)-1-(3-Chlorophenyl)-4-phenylbut-3-en-1-ol (**3bo**): 56% yield, 72 mg; colorless oil; ^{1}H NMR (400 MHz, $CDCl_3$) δ 7.39 (s, 1H), 7.28 (m, 8H), 6.49 (d, *J* = 16.0 Hz, 1H), 6.16 (m, 1H), 4.77 (m, 1H), 2.62 (m, 2H), 2.22 (br, 1H). ^{13}C NMR (101 MHz, $CDCl_3$) δ 145.9, 136.9, 134.3, 133.9, 129.7, 128.5, 127.6, 127.4, 126.2, 126.0, 125.2, 123.9, 73.0, 43.0. These data are in accordance with the literature.[5]

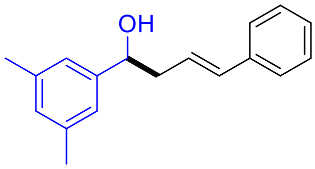

(*E*)-1-(3,5-Dimethylphenyl)-4-phenylbut-3-en-1-ol (**3bp**): 52% yield, 66 mg; colorless oil; ^{1}H NMR (400 MHz, $CDCl_3$) δ 7.34 (d, *J* = 8.0 Hz, 2H), 7.29 (t, *J* = 6.0 Hz, 2H), 7.18 (m, 1H), 6.99 (s, 2H), 6.92 (s, 1H), 6.50 (d, *J* = 16.0 Hz, 1H), 6.21 (m, 1H), 4.72 (t, *J* = 6.0 Hz, 1H), 2.63 (t, *J* = 6.0 Hz, 2H), 2.32 (s, 6H), 2.06 (br, 1H); ^{13}C NMR (101 MHz, $CDCl_3$) δ 143.9, 138.0, 137.2, 133.2, 129.2, 128.5, 127.2, 126.2, 126.1, 123.6, 73.8, 43.0, 21.3; HRMS (ESI): calcd for $C_{18}H_{20}O$ $(M+H)^+$ 253.1587, found 253.1590.

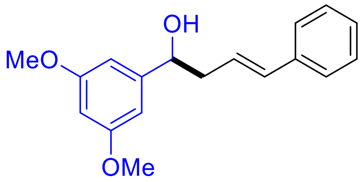

(*E*)-1-(3,5-Dimethoxyphenyl)-4-phenylbut-3-en-1-ol (**3bq**): 65% yield, 92 mg; colorless oil; ^{1}H NMR (400 MHz, $CDCl_3$) δ 7.33 (d, *J* = 8.0 Hz, 2H), 7.28 (t, *J* = 8.0 Hz, 2H), 7.20 (t, *J* = 6.0 Hz, 1H), 6.54 (d, *J* = 4.0 Hz, 2H), 6.48 (d, *J* = 16.0 Hz, 1H), 6.37 (t, *J* = 2.0 Hz, 1H), 6.19 (m, 1H), 4.71 (t, *J* = 6.0 Hz, 1H), 3.77 (s, 6H), 2.62 (t, *J* = 8.0 Hz, 2H), 2.21 (br, 1H); ^{13}C NMR (101 MHz, $CDCl_3$) δ 160.8, 146.5, 137.2, 133.3, 128.5, 127.2, 126.1, 125.9, 103.7, 99.5, 73.7, 55.3, 42.9. These data are in accordance with the literature.[5]

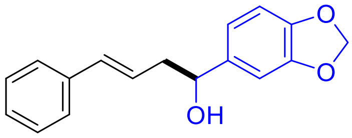

(*E*)-1-(Benzo[*d*][1,3]dioxol-5-yl)-4-phenylbut-3-en-1-ol (**3br**): 60% yield, 81 mg; white solid; ^{1}H NMR (400 MHz, $CDCl_3$) δ 7.31 (m, 4H), 7.21 (t, *J* = 8.0 Hz, 1H), 6.91 (s, 1H), 6.82 (d, *J* = 8.0 Hz, 1H), 6.77 (d, *J* = 8.0 Hz, 1H), 6.49 (d, *J* = 16.0 Hz, 1H), 6.17 (m, 1H), 5.95 (s, 2H), 4.71 (t, *J* = 6.0 Hz, 1H), 2.62 (t, *J* = 6.0 Hz, 2H), 2.09 (br, 1H); ^{13}C NMR (101 MHz, $CDCl_3$) δ 147.8, 146.9, 138.0, 137.1, 133.3, 128.5, 127.3, 126.1, 125.8, 119.2, 108.1, 106.3, 101.0, 73.6, 43.0. These data are in accordance with the literature.[5]

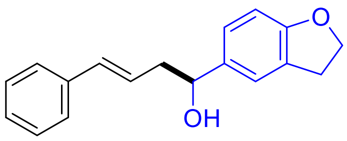

(E)-1-(2,3-Dihydrobenzofuran-5-yl)-4-phenylbut-3-en-1-ol (**3bs**): 71% yield, 95 mg; white solid; ^{1}H NMR (400 MHz, $CDCl_3$) δ 7.26 (m, 6H), 7.10 (d, J = 12.0 Hz, 1H), 6.75 (d, J = 8.0 Hz, 1H), 6.49 (d, J = 12.0 Hz, 1H), 6.19 (m, 1H), 4.72 (t, J = 8.0 Hz, 1H), 4.56 (t, J = 8.0 Hz, 2H), 3.19 (t, J = 10.0 Hz, 2H), 2.63 (t, J = 6.0 Hz, 2H), 2.03 (br, 1H); ^{13}C NMR (101 MHz, $CDCl_3$) δ 159.6, 137.3, 136.1, 133.1, 128.5, 127.24, 127.23, 126.2, 126.1, 125.8, 122.5, 109.0, 73.7, 71.3, 43.1, 29.7. These data are in accordance with the literature.[5]

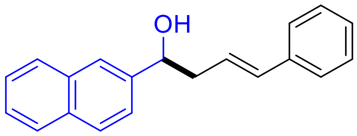

(E)-1-(Naphthalen-2-yl)-4-phenylbut-3-en-1-ol (**3bt**): 73% yield, 100 mg; white solid; ^{1}H NMR (400 MHz, $CDCl_3$) δ 7.83 (m, 4H), 7.48 (m, 3H), 7.32 (d, J = 8.0 Hz, 2H), 7.27 (t, J = 8.0 Hz, 2H), 7.20 (t, J = 6.0 Hz, 1H), 6.51 (d, J = 16.0 Hz, 1H), 6.21 (m, 1H), 4.95 (t, J = 6.0 Hz, 1H), 2.73 (m, 2H), 2.22 (br, 1H); ^{13}C NMR (101 MHz, $CDCl_3$) δ 141.3, 137.1, 133.5, 133.3, 133.0, 128.5, 128.2, 128.0, 127.7, 127.3, 126.2, 126.1, 125.83, 125.77, 124.5, 124.0, 73.8, 43.0; These data are in accordance with the literature.[5]

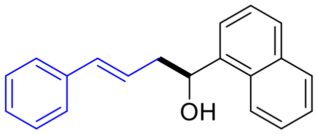

(E)-1-(Naphthalen-1-yl)-4-phenylbut-3-en-1-ol (**3bu**): 60% yield, 82 mg; colorless oil; ^{1}H NMR (400 MHz, $CDCl_3$) δ 8.07 (d, J = 8.0 Hz, 1H), 7.86 (d, J = 8.0 Hz, 1H), 7.76 (d, J = 8.0 Hz, 1H), 7.66 (d, J = 8.0 Hz, 1H), 7.48 (m, 3H), 7.33 (d, J = 4.0 Hz, 2H), 7.27 (t, J = 8.0 Hz, 2H), 7.19 (t, J = 6.0 Hz, 1H), 6.51 (d, J = 16.0 Hz, 1H), 6.28 (m, 1H), 5.55 (m, 1H) 2.89 (m, 1H), 2.72 (m, 1H), 2.30 (br, 1H); ^{13}C NMR (101 MHz, $CDCl_3$) δ 139.4, 137.2, 133.8, 133.2, 130.2, 128.9, 128.5, 128.0, 127.3, 126.2, 126.1, 126.0, 125.5, 125.4, 123.0, 122.8, 70.4, 42.1. These data are in accordance with the literature.[5]

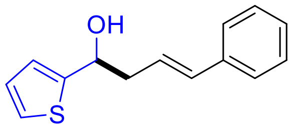

(E)-4-Phenyl-1-(thiophen-2-yl)but-3-en-1-ol (**3bv**): 51% yield, 59 mg; colorless oil; ^{1}H NMR (400 MHz, $CDCl_3$) δ 7.26 (m, 6H), 7.00 (d, J = 4.0 Hz , 1H), 6.97 (t, J = 4.0 Hz, 1H), 6.51 (d, J = 16.0 Hz, 1H), 6.21 (m, 1H) 5.04 (t, J = 6.0 Hz, 1H), 2.76 (t, J = 8.0 Hz, 2H), 2.28 (br, 1H); ^{13}C NMR (101 MHz, $CDCl_3$) δ 147.8, 137.1, 133.7, 128.5, 127.4, 126.6, 126.2, 125.2, 124.6, 123.7, 69.8, 43.0. These data are in accordance with the literature.[5]

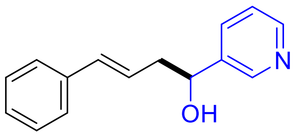

(E)-4-Phenyl-1-(pyridin-3-yl)but-3-en-1-ol (**3bw**): 44% yield, 50 mg; yellow solid, m.p. 97-100 °C; ^{1}H NMR (400 MHz, $CDCl_3$) δ 8.53 (s, 2H), 7.74 (d, J = 8.0 Hz, 1H), 7.31 (m, 5H), 7.22 (t, J = 8.0 Hz, 1H), 6.49 (d, J = 16.0 Hz, 1H), 6.18 (m, 1H), 4.85 (m, 1H), 3.07 (br, 1H), 2.66 (m, 2H); ^{13}C NMR (101 MHz, $CDCl_3$) δ 148.7, 147.7, 136.9, 134.0, 133.6, 128.6, 128.5, 128.2, 127.5, 126.2, 125.0, 71.4, 43.0; HRMS (ESI): calcd for $C_{15}H_{15}NO$ $(M+H)^+$ 226.1226, found 226.1226.

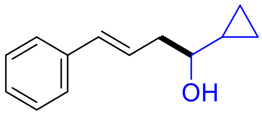

(E)-1-Cyclopropyl-4-phenylbut-3-en-1-ol (**3bx**): 61% yield, 57 mg; colorless oil; ^{1}H NMR (400 MHz, $CDCl_3$) δ 7.36 (d, J = 8.0 Hz, 2H), 7.29 (t, J = 8.0 Hz, 2H), 7.20 (t, J = 8.0 Hz, 1H), 6.49 (d, J = 16.0 Hz, 1H), 6.30 (m, 1H), 3.01 (m, 1H), 2.53 (m, 2H), 1.77 (br, 1H), 0.97 (m, 1H), 0.54 (m, 2H), 0.30 (m, 2H); ^{13}C NMR (101 MHz, $CDCl_3$) δ 137.4, 132.6, 128.5, 127.1, 126.5, 126.1, 76.2, 40.9, 17.4, 2.8, 2.5. These data are in accordance with the literature.[5]

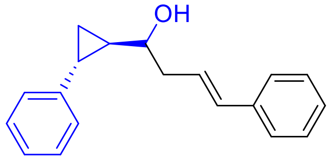

(E)-4-Phenyl-1-(2-phenylcyclopropyl)but-3-en-1-ol (**3by**): 65% yield, 86 mg; colorless oil; Isomers of products can be isolated by column. **Isomer I** (40 mg): ^{1}H NMR (400 MHz, $CDCl_3$) δ 7.30 (m, 7H), 7.12 (m, 3H), 6.50 (d, J = 16.0 Hz, 1H), 6.30(m, 1H), 3.35 (t, J = 8.0 Hz, 1H), 2.57(m, 2H), 1.96 (m, 1H), 1.74 (br, 1H), 1.31 (m, 1H), 0.95 (m, 2H); ^{13}C NMR (101 MHz, $CDCl_3$) δ 142.5, 137.3, 133.0, 128.5, 128.3, 127.2, 126.1, 126.0, 125.9, 125.6, 74.7, 40.7, 29.0, 21.0, 13.4; **Isomer II** (46 mg): ^{1}H NMR (400 MHz, $CDCl_3$) δ 7.26 (m, 7H), 7.15 (t, J = 8.0 Hz, 1H), 7.05 (d, J = 4.0 Hz, 2H), 6.46 (d, J = 16.0 Hz, 1H), 6.27 (m, 1H), 3.32 (dd, J = 12.0 Hz, J = 8.0 Hz, 1H), 2.57 (m, 2H), 1.86 (m, 1H), 1.77 (br, 1H), 1.29 (m, 1H), 1.02 (m, 2H); ^{13}C NMR (101 MHz, $CDCl_3$) δ 142.2, 137.2, 133.0, 128.5, 128.3, 127.2, 126.1, 126.0, 125.9, 125.6, 75.0, 41.1, 29.1, 21.0, 13.5. These data are in accordance with the literature.[5]

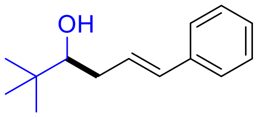

(E)-2,2-Dimethyl-6-phenylhex-5-en-3-ol (**3bz**): 30% yield, 31 mg; colorless oil; ^{1}H NMR (400 MHz, $CDCl_3$) δ 7.36 (d, J = 8.0 Hz, 2H), 7.30 (t, J = 8.0 Hz, 2H), 7.21 (t, J = 8.0 Hz, 1H), 6.49 (d, J = 16.0 Hz, 1H), 6.27 (m, 1H), 3.35 (d, J = 12.0 Hz, 1H), 2.51 (dd, J = 16.0 Hz, J = 8.0 Hz, 1H), 2.16 (m, 1H), 1.64 (br, 1H), 0.96 (s, 9H); ^{13}C NMR (101 MHz, $CDCl_3$) δ 137.3, 132.8, 128.5, 128.1, 127.2, 126.1, 78.7, 35.7, 34.8, 25.8. These data are in accordance with the literature.[5]

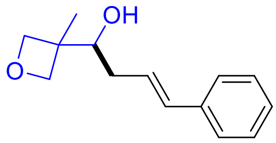

(*E*)-1-(3-Methyloxetan-3-yl)-4-phenylbut-3-en-1-ol (**3ca**): 44% yield, 48 mg; colorless oil; ^{1}H NMR (400 MHz, CDCl$_3$) δ 7.29 (m, 5H), 6.51 (d, *J* = 16.0 Hz, 1H), 6.20 (m, 1H), 4.69 (d, *J* = 4.0 Hz, 1H), 4.58 (d, *J* = 4.0 Hz, 1H), 4.36 (d, *J* = 4.0 Hz, 1H), 4.31 (d, *J* = 8.0 Hz, 1H), 3.99 (d, *J* = 12.0 Hz, 1H), 2.26 (m, 2H), 2.04 (br, 1H), 1.35 (s, 3H); ^{13}C NMR (101 MHz, CDCl$_3$) δ 136.9, 133.5, 128.6, 127.4, 126.0, 125.9, 80.8, 79.7, 74.7, 43.1, 35.9, 18.7; HRMS (ESI): calcd for $C_{14}H_{18}O_2$ (M+H)$^+$ 219.1380, found 219.1388.

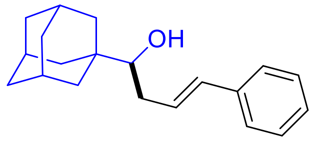

(*E*)-1-((3r,5r,7r)-Adamantan-1-yl)-4-phenylbut-3-en-1-ol (**3cb**): 23% yield, 32 mg; pale yellow solid, m.p. 80-82 °C; ^{1}H NMR (400 MHz, CDCl$_3$) δ 7.36 (d, *J* = 8.0 Hz, 2H), 7.29 (t, *J* = 8.0 Hz, 2H), 7.20 (t, *J* = 8.0 Hz, 1H), 6.48 (d, *J* = 16.0 Hz, 1H), 6.27 (m, 1H), 3.17 (d, *J* = 8.0 Hz, 1H), 2.50 (m, 1H), 2.18 (m, 1H), 2.01 (s, 3H), 1.67 (m, 13H); ^{13}C NMR (101 MHz, CDCl$_3$) δ 137.3, 132.7, 128.5, 128.3, 127.1, 126.0, 78.9, 38.1, 37.2, 36.5, 34.3, 28.4; HRMS (ESI): calcd for $C_{20}H_{26}O$ (M+H)$^+$ 283.2056, found 283.2054.

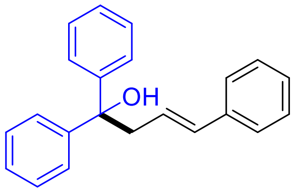

(*E*)-1,1,4-Triphenylbut-3-en-1-ol (**3cc**): 37% yield, 56 mg; white solid; ^{1}H NMR (400 MHz, CDCl$_3$) δ 7.47 (d, *J* = 8.0 Hz, 4H), 7.32 (t, *J* = 8.0 Hz, 4H), 7.21 (m, 7H), 6.56 (d, *J* = 16.0 Hz, 1H), 6.04 (m, 1H), 3.22 (d, *J* = 8.0 Hz, 2H), 2.58 (br, 1H) ; ^{13}C NMR (101 MHz, CDCl$_3$) δ 146.4, 136.8, 135.3, 128.4, 128.1, 127.4, 126.8, 126.2, 125.9, 124.5, 77.3, 45.9; These data are in accordance with the literature.[5]

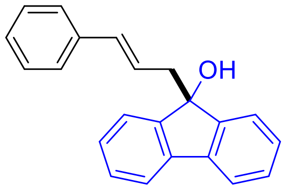

9-Cinnamyl-9*H*-fluoren-9-ol (**3cd**): 32% yield, 48 mg; colorless oil; ^{1}H NMR (400 MHz, CDCl$_3$) δ 7.59 (d, *J* = 8.0 Hz, 2H), 7.52 (d, *J* = 8.0 Hz, 2H), 7.34 (t, *J* = 8.0 Hz, 2H), 7.27 (t, *J* = 8.0 Hz, 2H), 7.18 (m, 5H), 6.30 (d, *J* = 16.0 Hz, 1H), 6.13 (m, 1H), 2.90 (d, *J* = 8.0 Hz, 2H), 2.25 (br, 1H); ^{13}C NMR (101 MHz, CDCl$_3$) δ 148.4, 139.3, 137.4, 133.7, 129.0, 128.4, 127.8, 127.1, 126.1, 124.6, 123.9, 120.0, 81.8, 43.4; HRMS (ESI): calcd for $C_{22}H_{18}O$ (M+H)$^+$ 299.1430, found 299.1439.

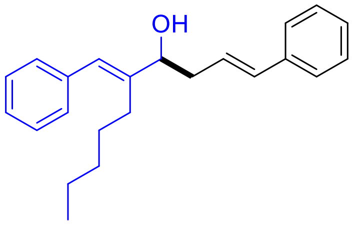

(*E*)-5-((*E*)-Benzylidene)-1-phenyldec-1-en-4-ol (**3ce**): 24% yield, 38 mg; colorless oil; ^{1}H NMR (400 MHz, $CDCl_3$) δ 7.33 (m, 6H), 7.23 (m, 4H), 6.60 (s, 1H), 6.53 (d, *J* = 16.0 Hz, 1H), 6.27 (m, 1H), 4.35 (q, *J* = 4.0 Hz, 1H), 2.64 (m, 1H), 2.53 (m, 1H), 2.41 (m, 1H), 2.17 (m, 1H), 1.83 (br, 1H), 1.54 (m, 2H), 1.30 (m, 4H), 0.87 (t, *J* = 6.0 Hz, 3H); ^{13}C NMR (101 MHz, $CDCl_3$) δ 144.7, 137.7, 137.2, 133.2, 128.6, 128.5, 128.2, 127.3, 126.4, 126.3, 126.1, 125.4, 74.9, 40.1, 32.2, 28.8, 22.4, 14.0; HRMS (ESI): calcd for $C_{23}H_{28}O$ $(M+H)^+$ 321.2213, found 321.2223.

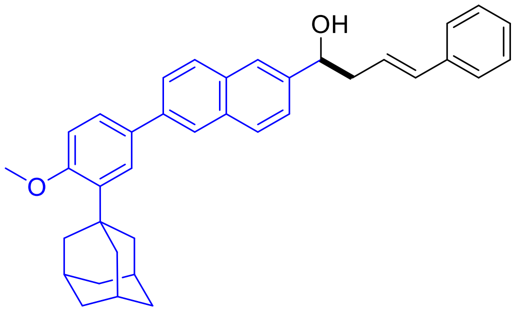

(*E*)-1-(6-(3-((3r,5r,7r)-Adamantan-1-yl)-4-methoxyphenyl)naphthalen-2-yl)-4-phenylbut-3-en-1-ol (**3cf**): 41% yield, 106 mg; white solid; ^{1}H NMR (400 MHz, $CDCl_3$) δ 7.98 (s, 1H), 7.88 (t, *J* = 8.0 Hz, 2H), 7.83 (s, 1H), 7.74 (d, *J* = 8.0 Hz, 1H), 7.59 (s, 1H), 7.52 (d, *J* = 8.0 Hz, 2H), 7.34 (d, *J* = 8.0 Hz, 2H), 7.29 (t, *J* = 8.0 Hz, 2H), 7.21 (t, *J* = 6.0 Hz, 1H), 6.98 (d, *J* = 8.0 Hz, 1H), 6.54 (d, *J* = 16.0 Hz, 1H), 6.21 (m, 1H), 4.98 (t, *J* = 6.0 Hz, 1H), 3.89 (s, 3H), 2.76 (t, *J* = 6.0 Hz, 2H), 2.19 (s, 7H), 2.10 (s, 3H), 1.80 (s, 6H); ^{13}C NMR (101 MHz, $CDCl_3$) δ 158.6, 140.9, 139.0, 138.8, 137.1, 133.5, 133.3, 133.1, 132.1, 128.5, 128.4, 128.3, 127.3, 126.2, 126.0, 125.9, 125.8, 125.6, 124.8, 124.3, 124.2, 112.1, 73.8, 55.2, 43.0, 40.6, 37.2, 37.1, 29.1. These data are in accordance with the literature.[5]

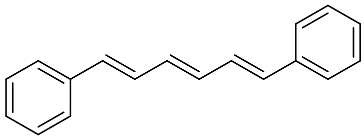

(*1E,3E,5E*)-1,6-diphenylhexa-1,3,5-triene (**4**): 94% yield, 110 mg; yellow solid; ^{1}H NMR (400 MHz, $CDCl_3$) δ 7.41 (d, *J* = 8.0 Hz, 4H), 7.32 (t, *J* = 6.0 Hz, 4H), 7.22 (t, *J* = 6.0 Hz, 2H), 6.88 (m, 2H), 6.60 (d, *J* = 12.0 Hz, 2H), 6.52 (dd, *J* = 8.0 Hz, *J* = 4.0 Hz, 2H); ^{13}C NMR (101 MHz, $CDCl_3$) δ 137.4, 133.6, 132.7, 129.1, 128.6, 127.5, 126.4. These data are in accordance with the literature.[12]

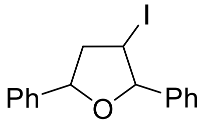

3-Iodo-2,5-diphenyltetrahydrofuran (**5**): 85% yield, 298 mg; colorless oil; Isomers of products can

be isolated by column. **Isomer I** (140 mg): ^{1}H NMR (400 MHz, $CDCl_3$) δ 7.51 (d, *J* = 4.0 Hz, 2H), 7.45 (d, *J* = 4.0 Hz, 2H), 7.35 (m, 6H), 5.38 (d, *J* = 4.0 Hz, 1H), 5.28 (t, *J* = 8.0 Hz, 2H), 4.15 (q, *J* = 8.0 Hz, 1H), 2.79 (m, 1H), 2.57 (m, 1H); ^{13}C NMR (101 MHz, $CDCl_3$) δ 140.9, 139.2, 128.57, 128.55, 128.4, 127.8, 126.3, 126.0, 90.7, 80.2, 46.4, 26.8; **Isomer II** (158 mg): ^{1}H NMR (400 MHz, $CDCl_3$) δ 7.52 (d, *J* = 8.0 Hz, 2H), 7.34 (m, 8H), 5.25 (t, *J* = 8.0 Hz, 2H), 4.09 (q, *J* = 9.3 Hz, 1H), 3.09 (m, 1H), 2.51 (q, *J* = 10.7 Hz, 1H); ^{13}C NMR (101 MHz, $CDCl_3$) δ 141.9, 138.9, 128.54, 128.50, 128.4, 127.7, 126.6, 125.6, 89.5, 81.0, 48.2, 25.6; HRMS (ESI): calcd for $C_{16}H_{15}IO$ $(M+H)^+$ 351.0240, found 351.0250.

Part II NMR spectra

3a ^{1}H NMR

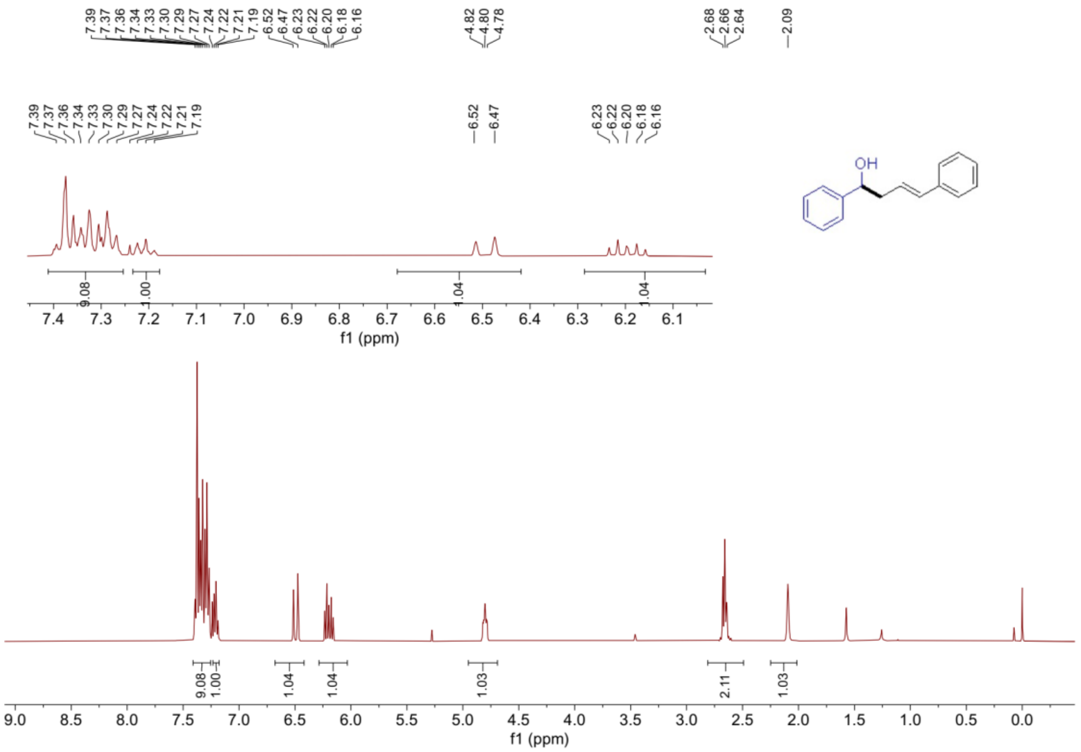

3a ^{13}C NMR

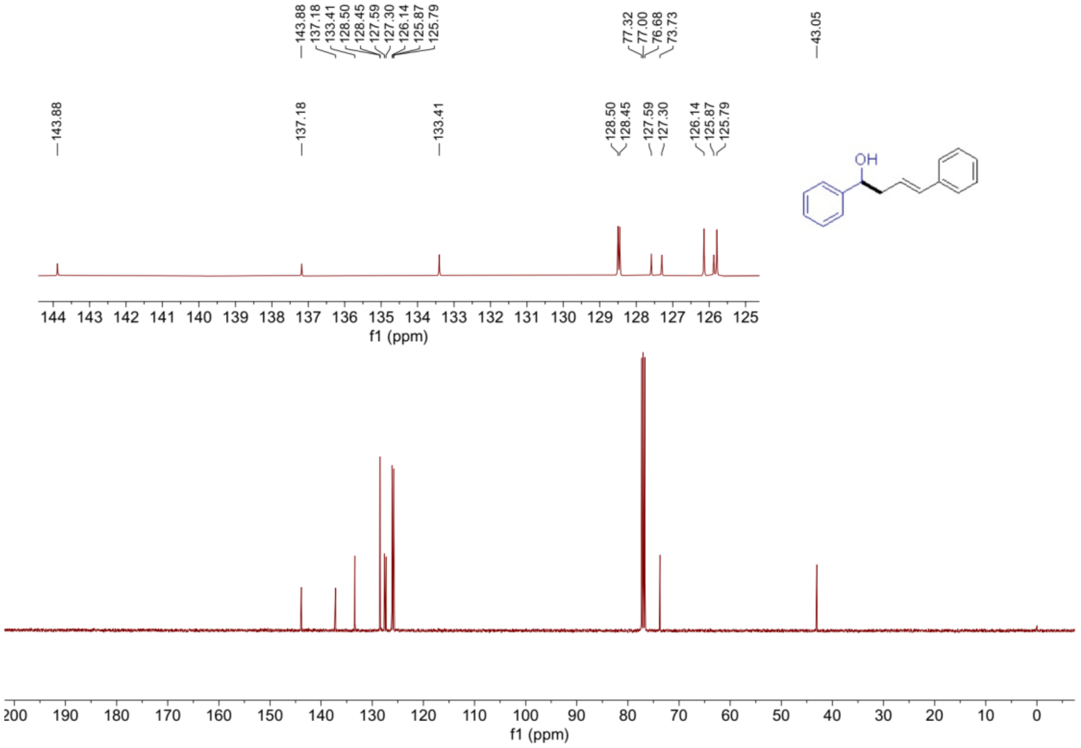

3b ^{1}H NMR

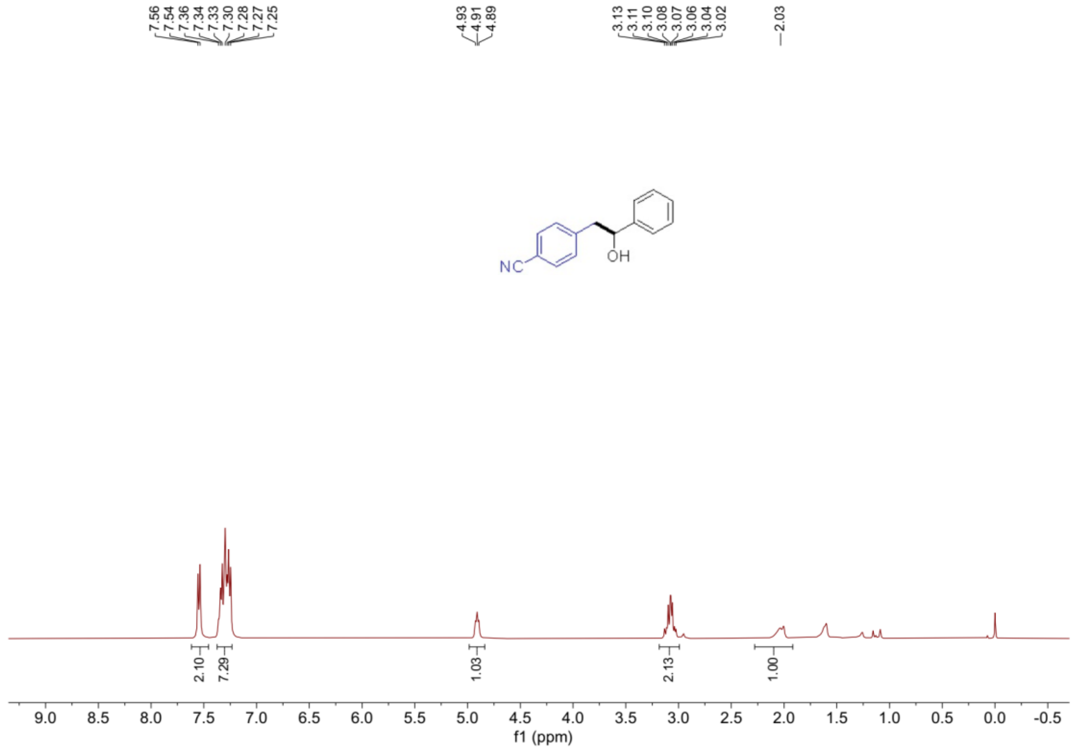

3b ^{13}C NMR

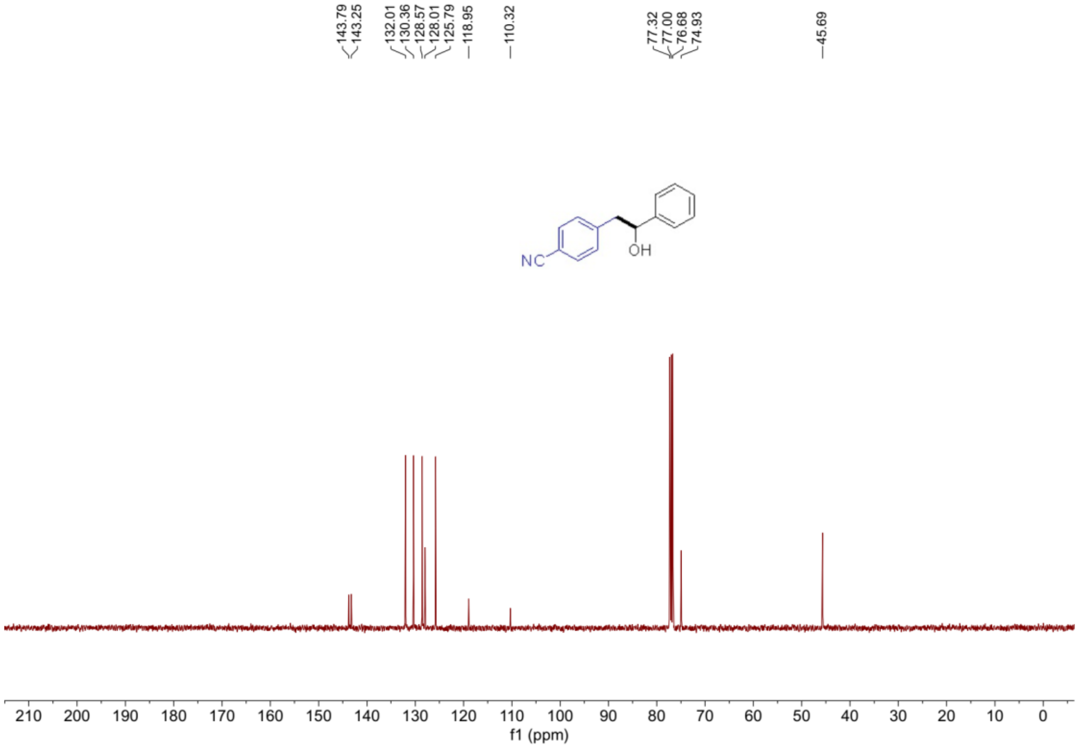

3c ^{1}H NMR

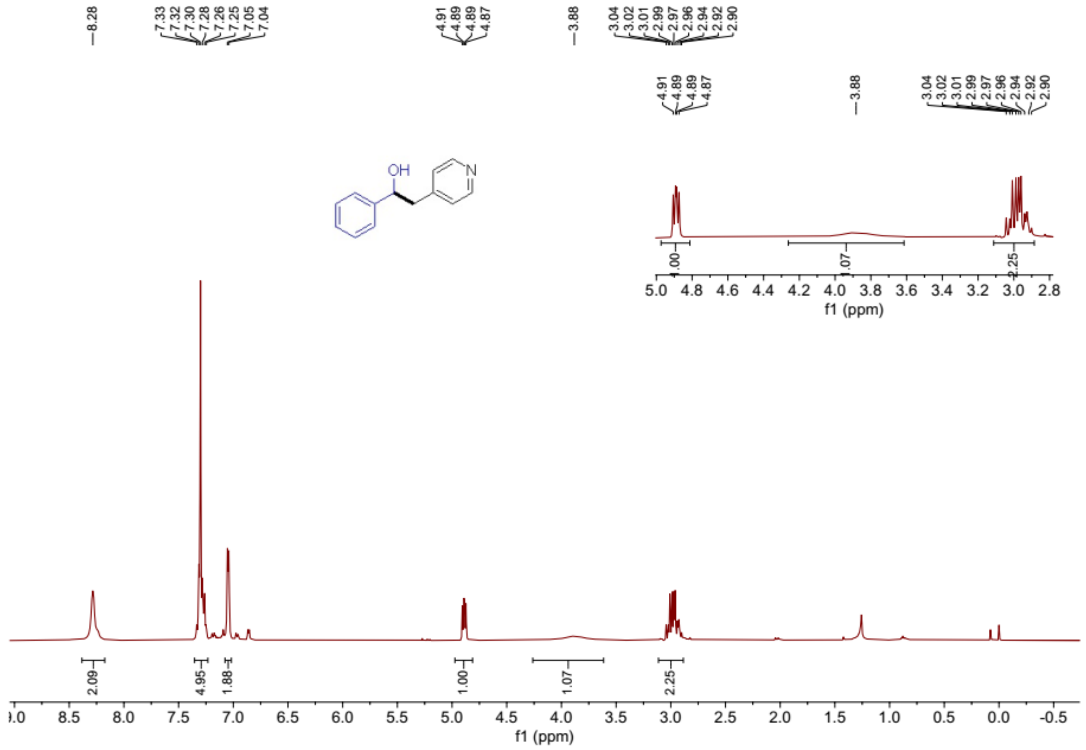

3c ^{13}C NMR

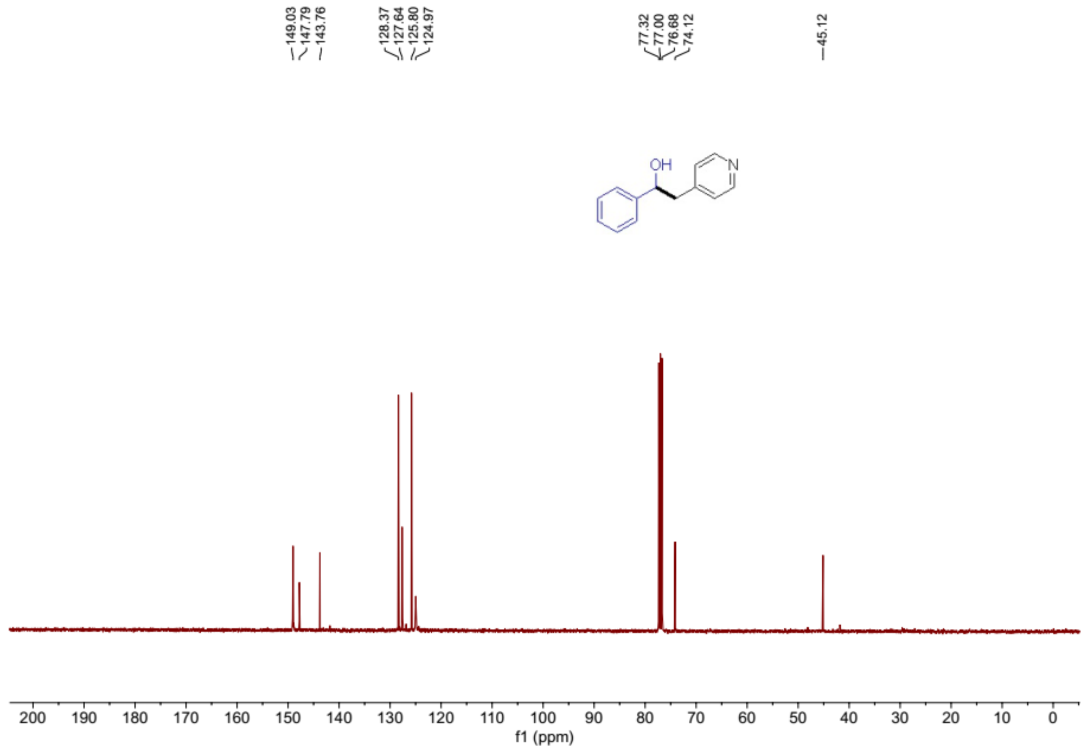

3d ^{1}H NMR

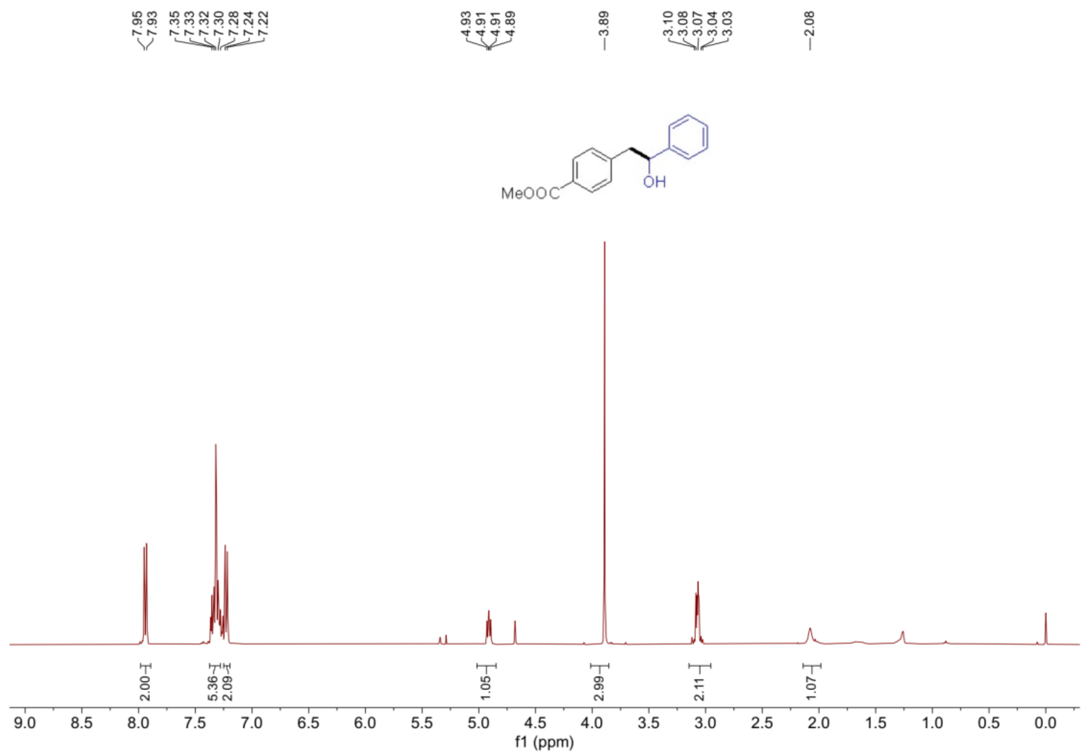

3d ^{13}C NMR

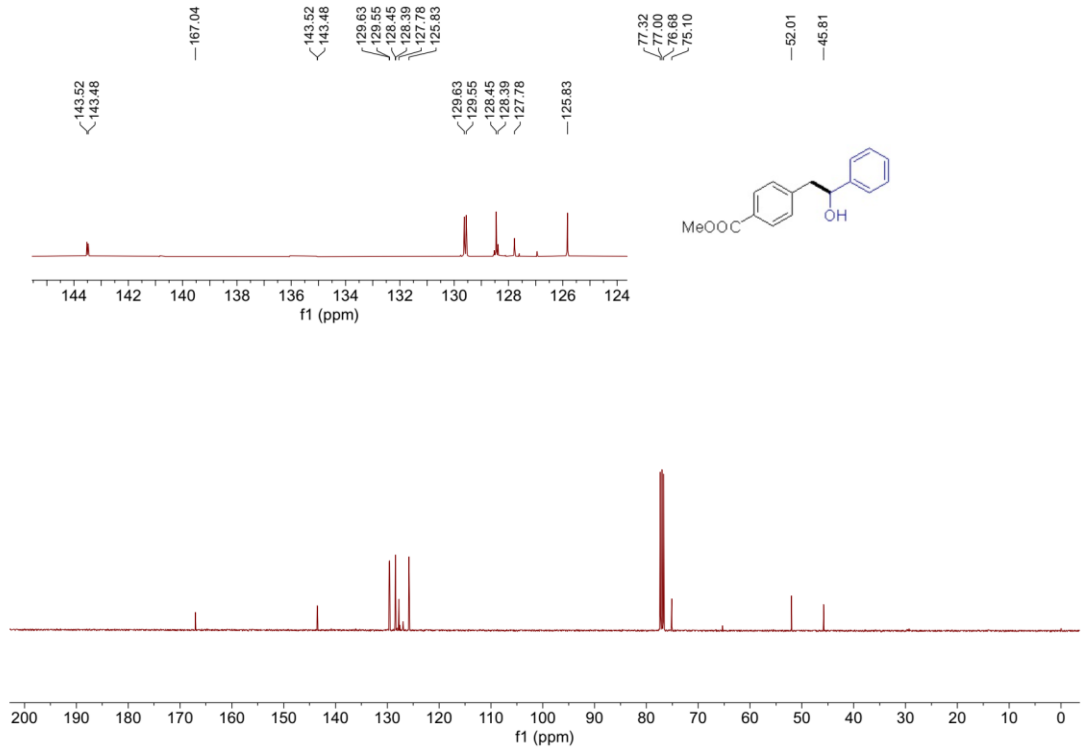

3e ^{1}H NMR

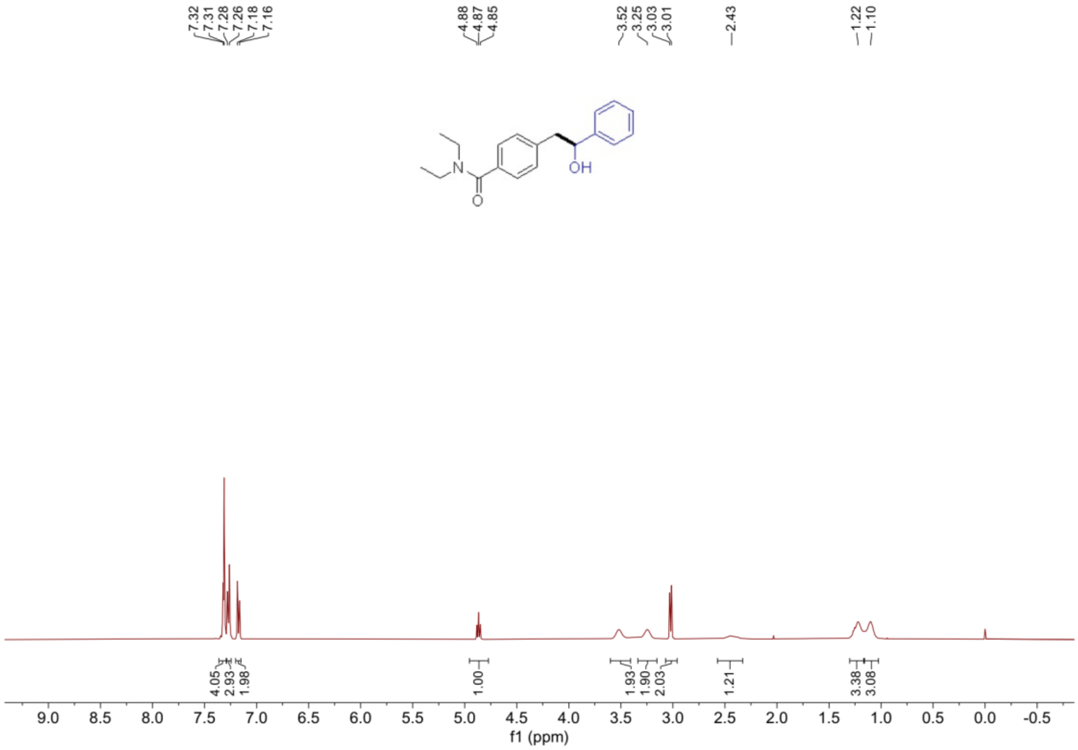

3e ^{13}C NMR

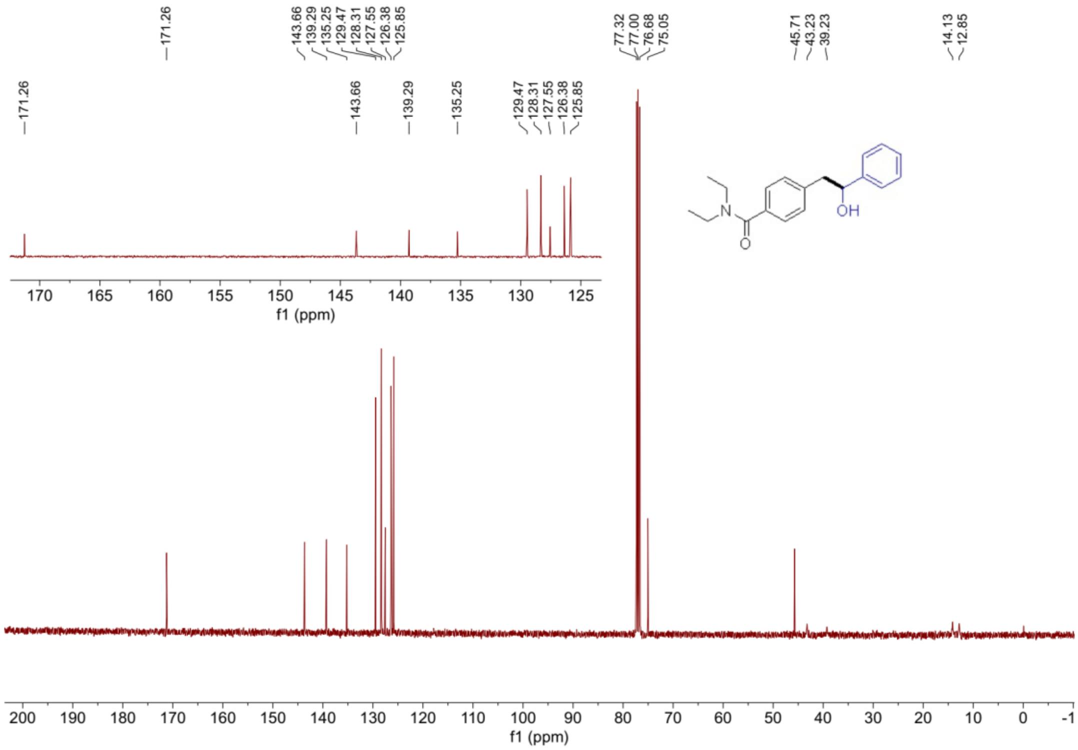

3f ^{1}H NMR

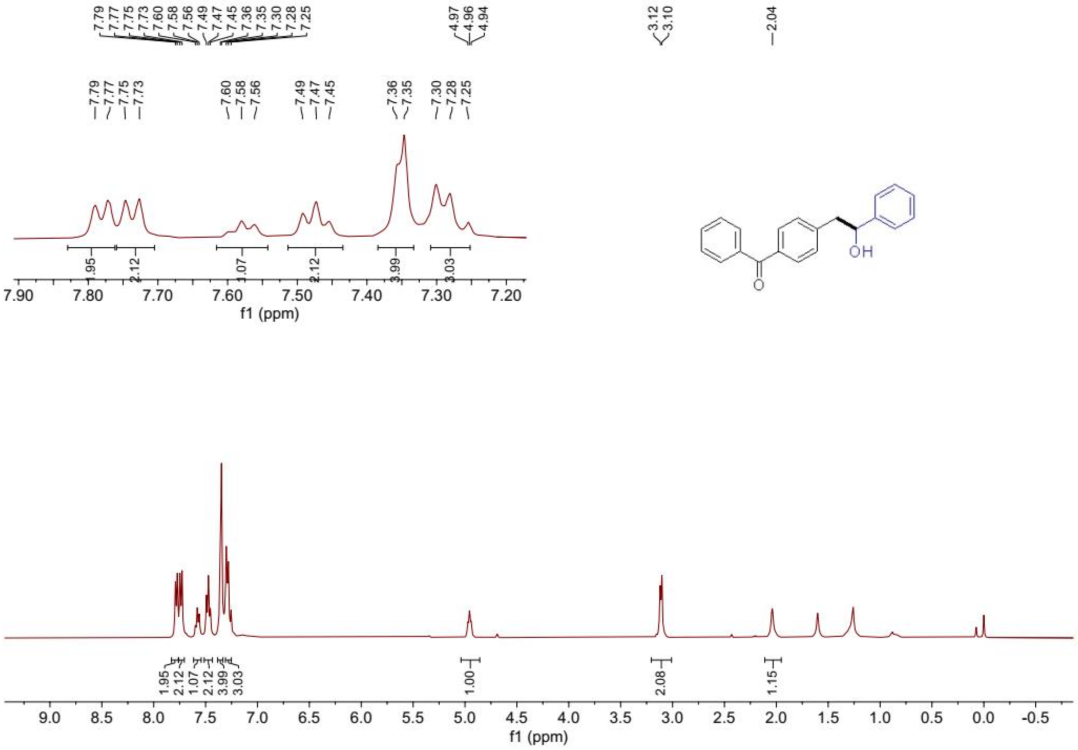

3f ^{13}C NMR

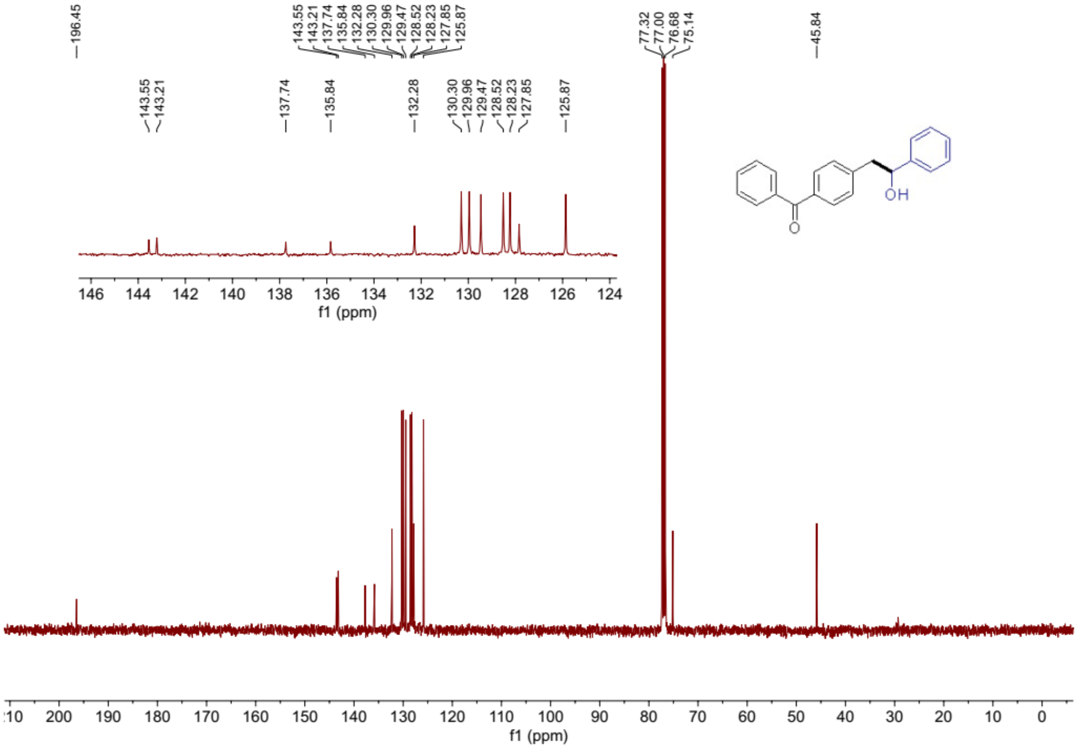

3g ¹H NMR

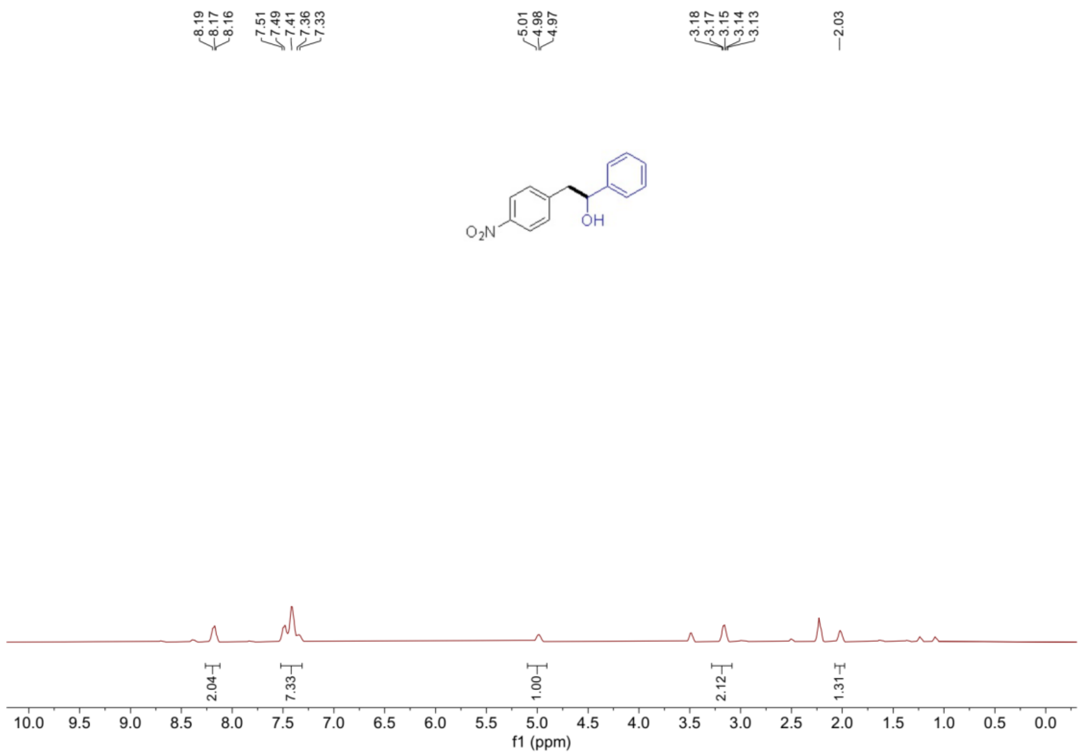

3g ¹³C NMR

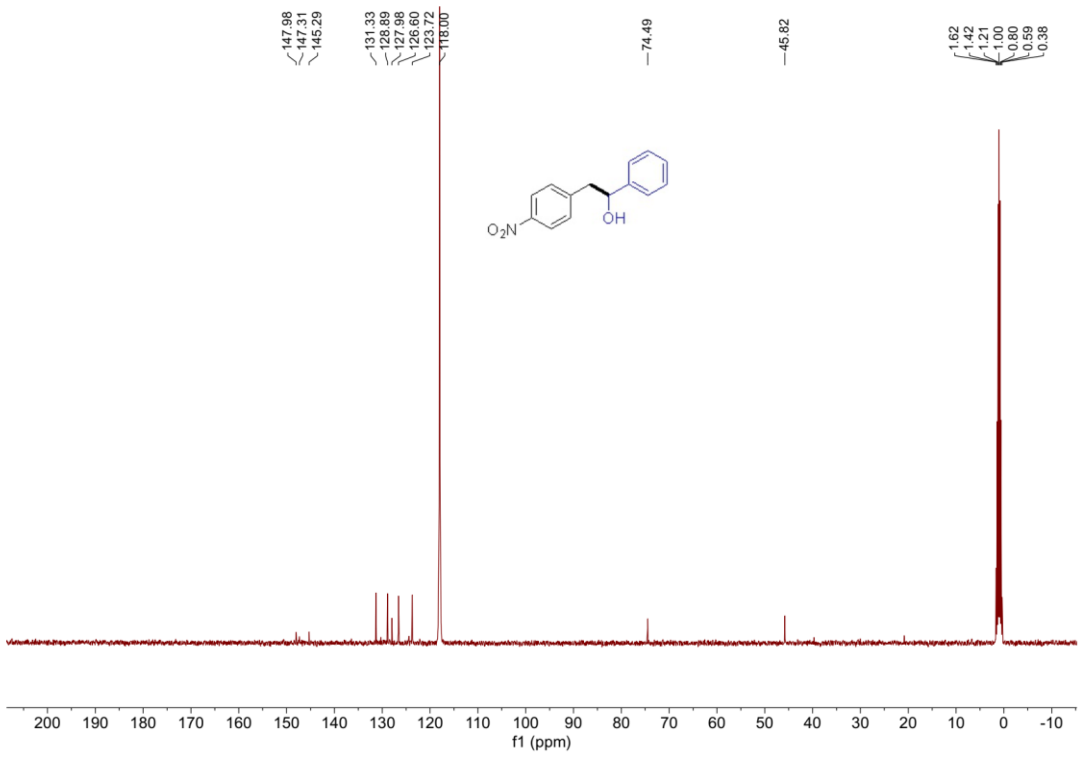

3h ^{1}H NMR

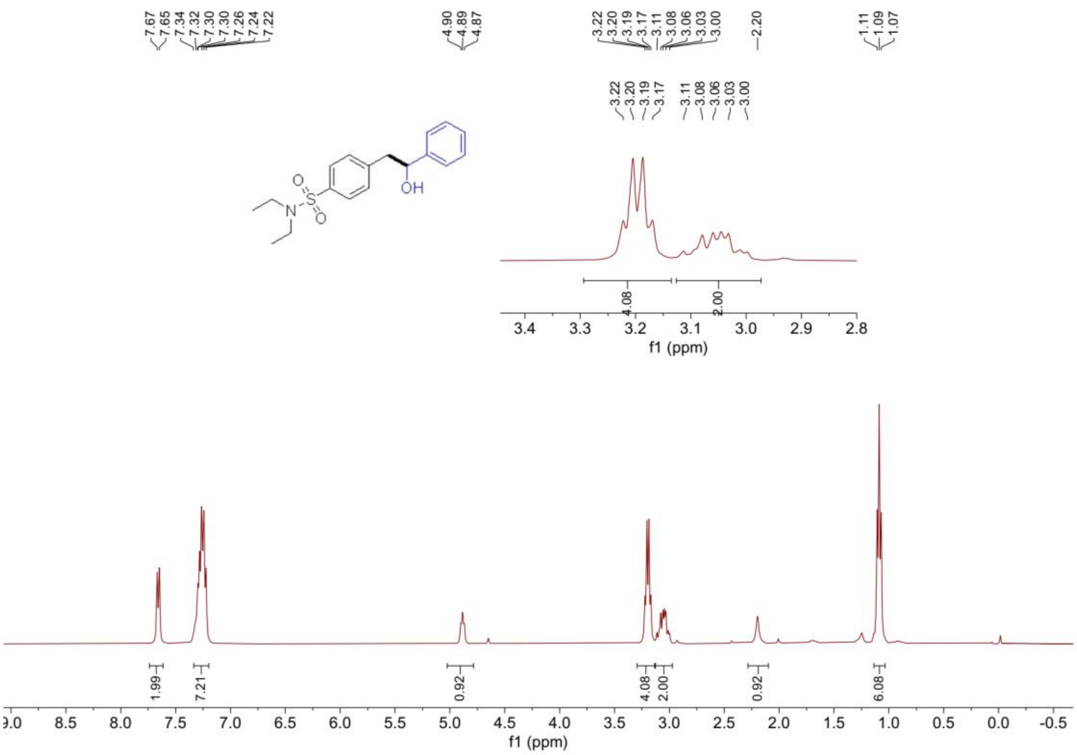

3h ^{13}C NMR

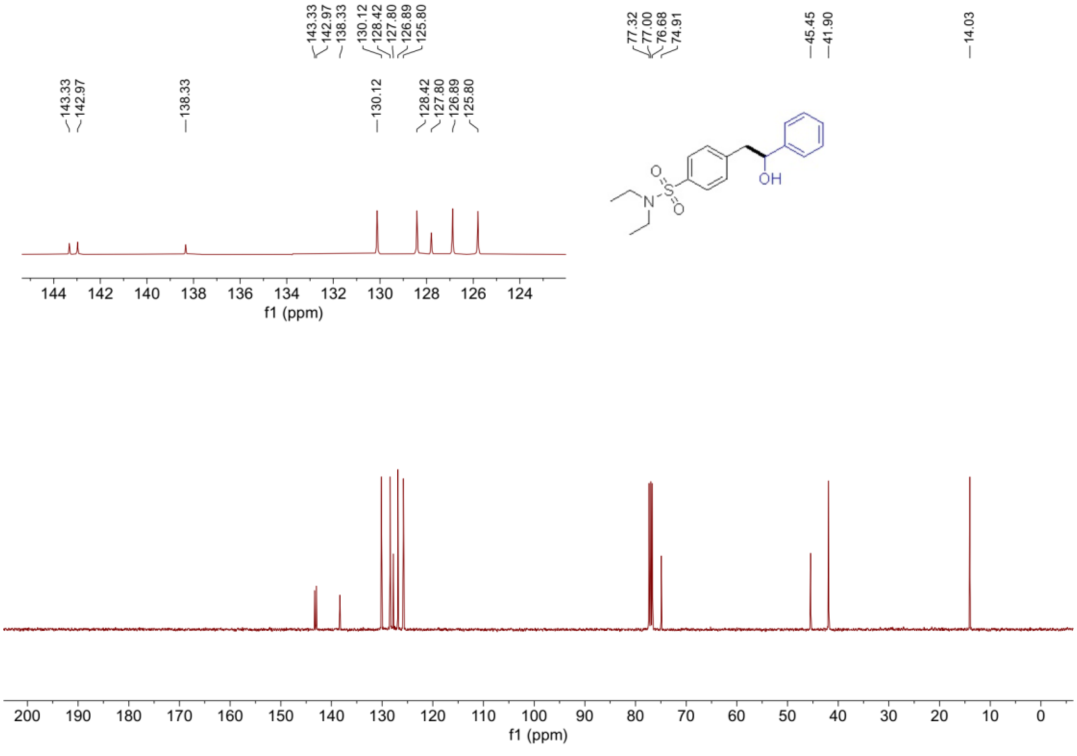

3i ^{1}H NMR

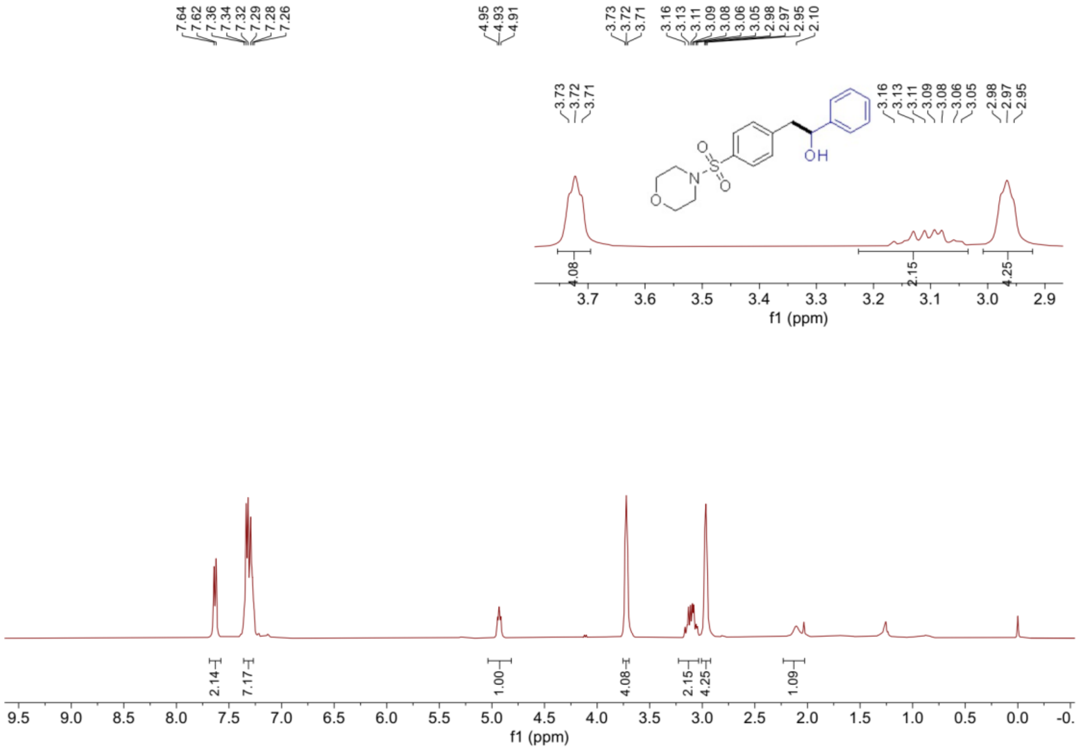

3i ^{13}C NMR

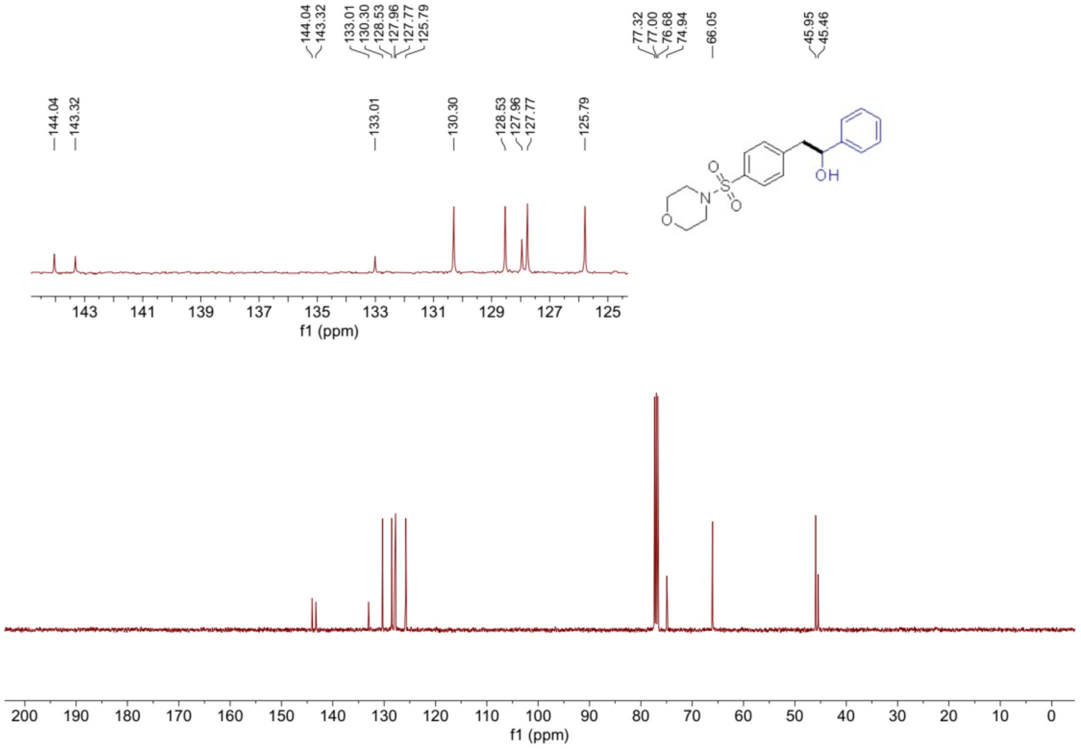

3j ^{1}H NMR (is in accordance with 3a)

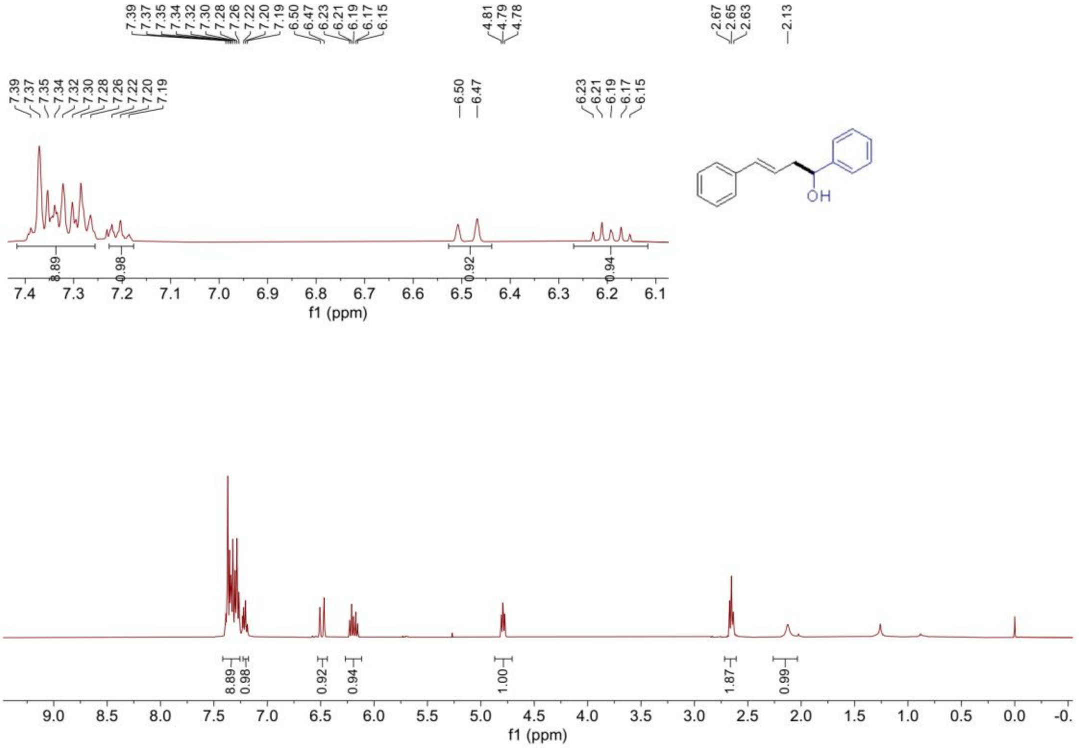

3j *E/Z* selectivity

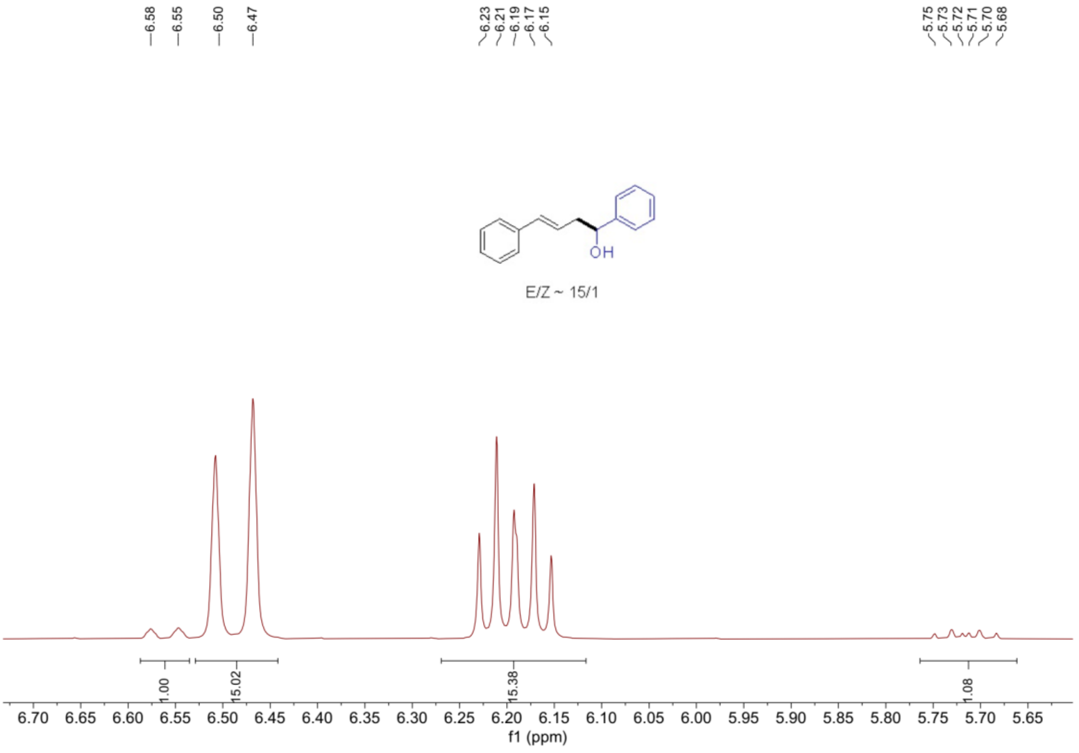

3k ^{1}H NMR (is in accordance with 3ad)

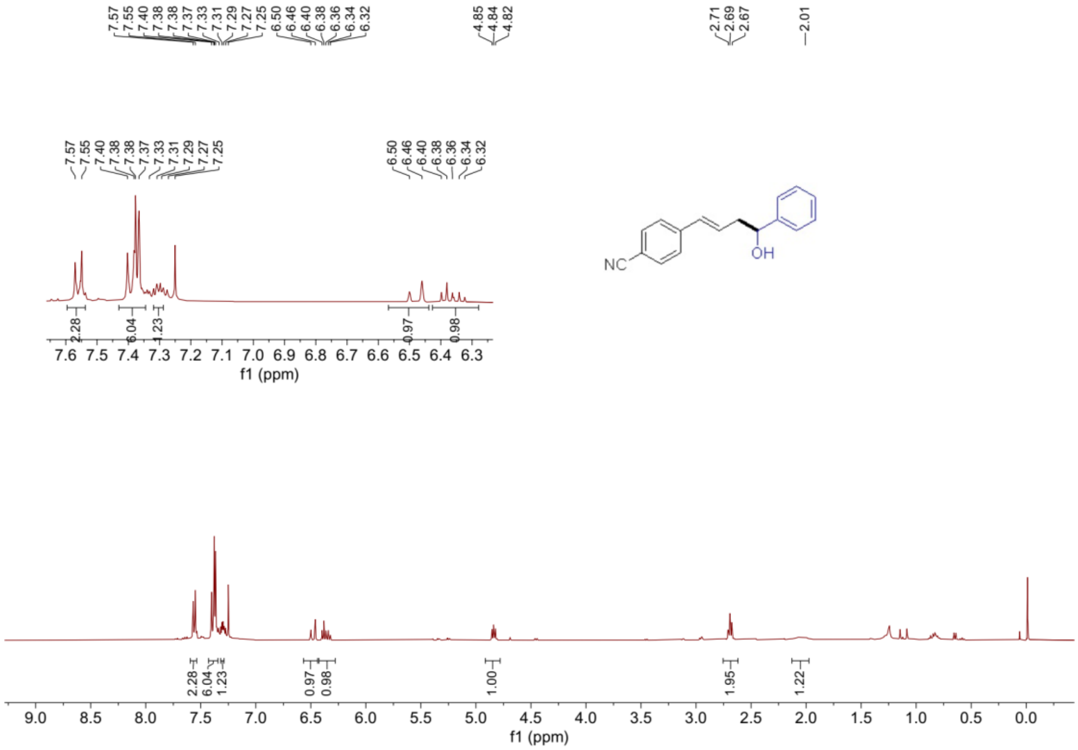

3k *E/Z* selectivity

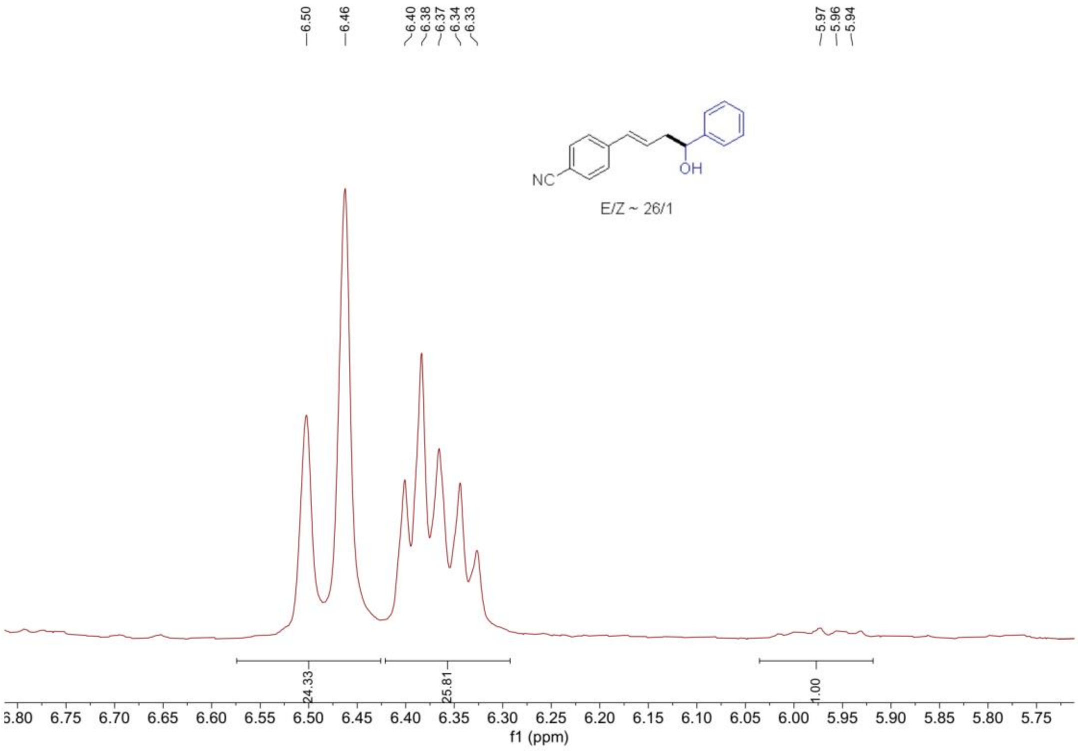

3l ¹H NMR

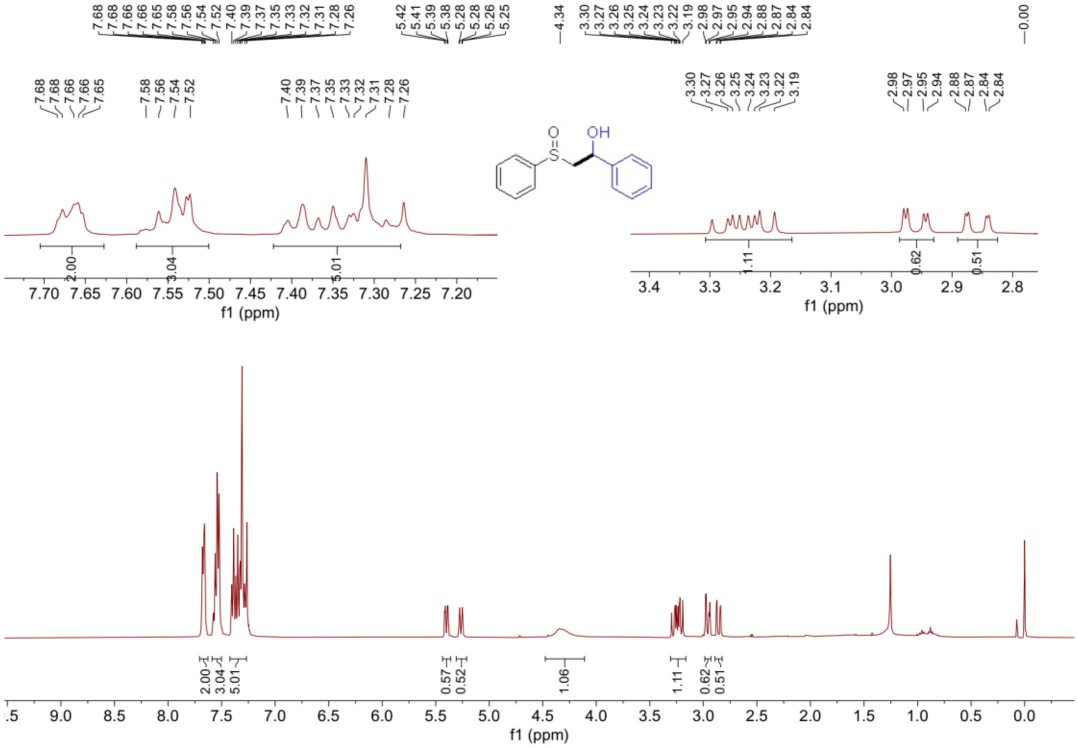

3l ¹³C NMR

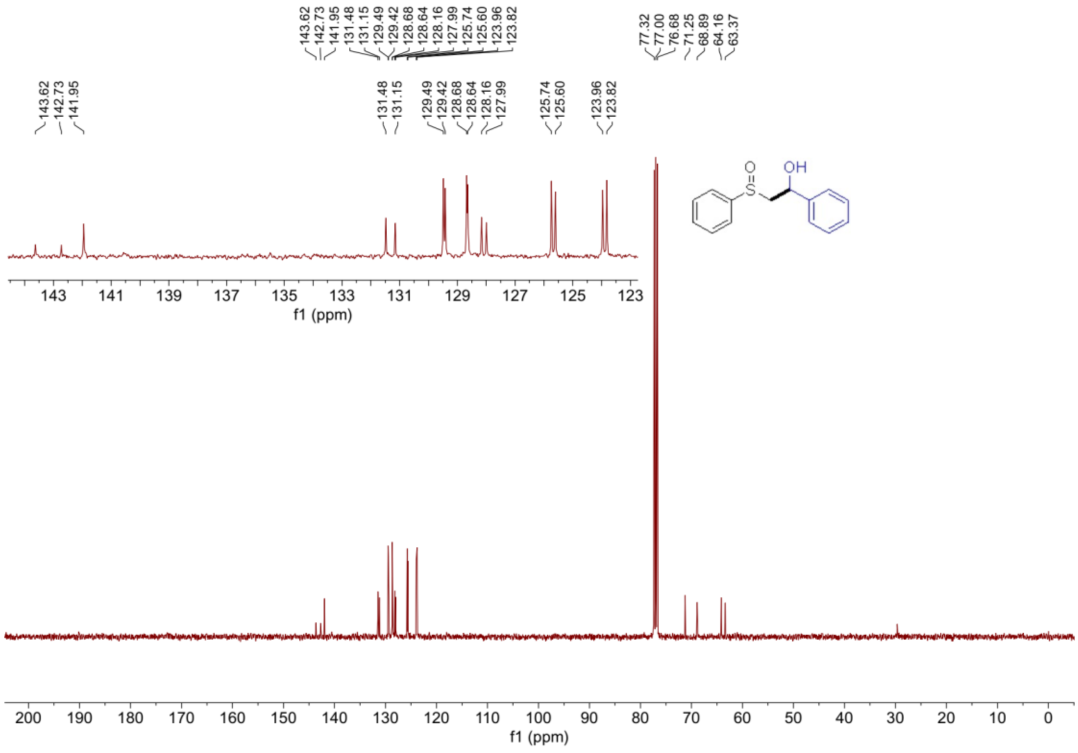

3m ^{1}H NMR

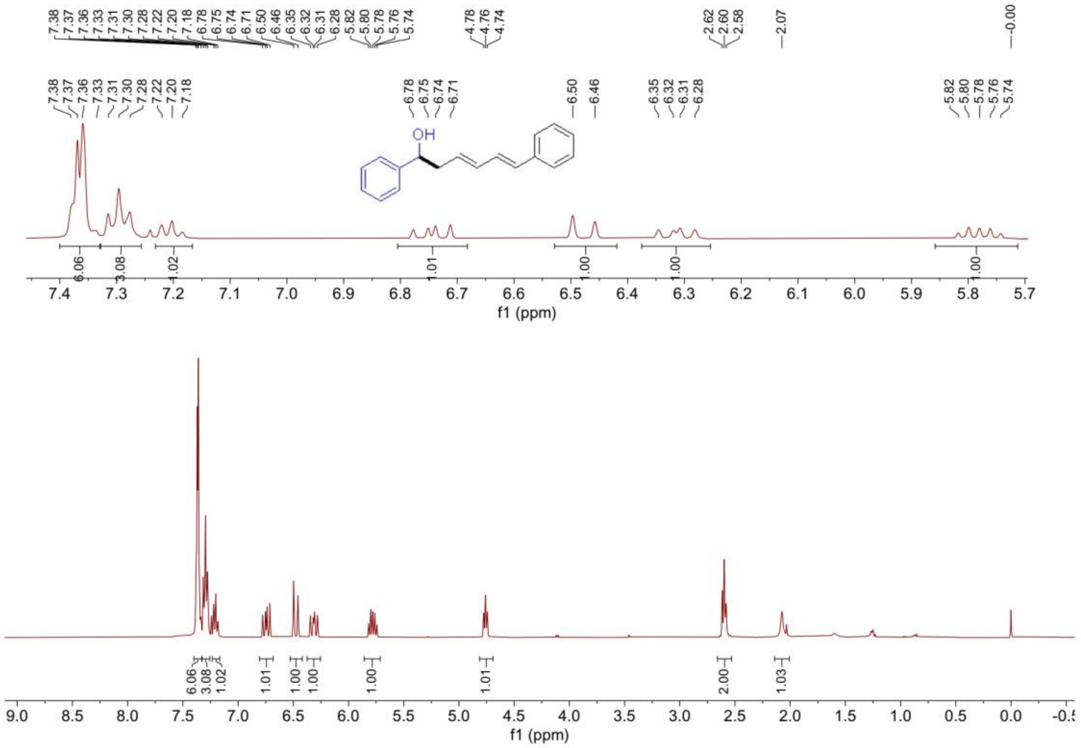

3m ^{13}C NMR

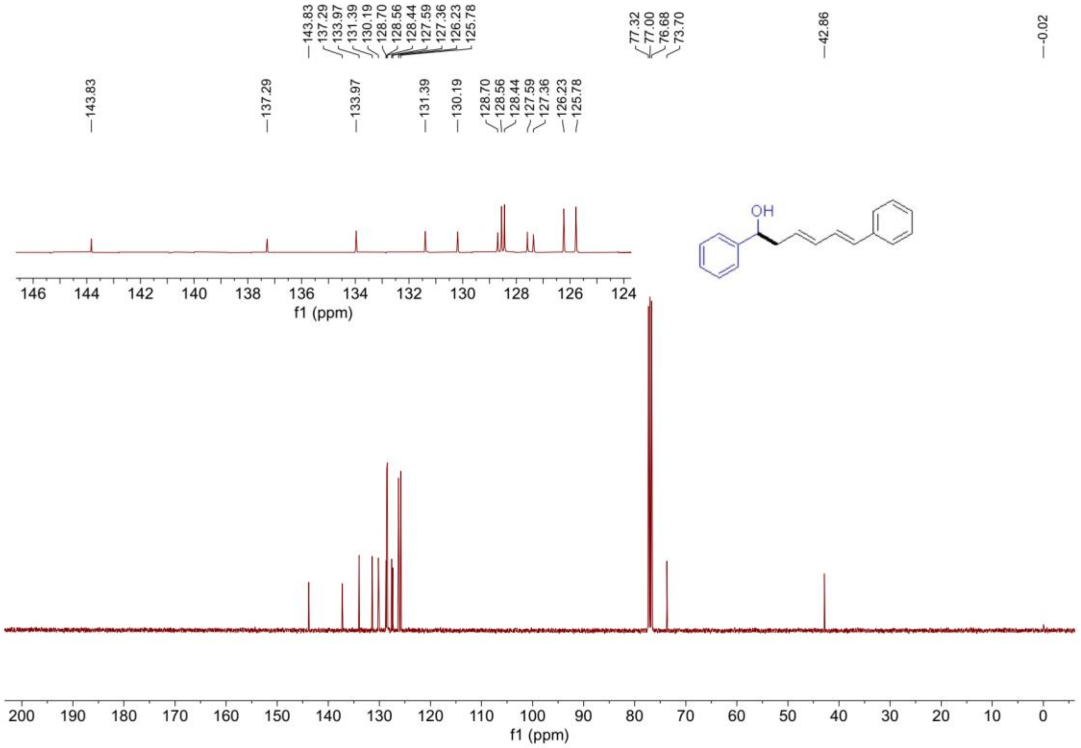

3n ^{1}H NMR

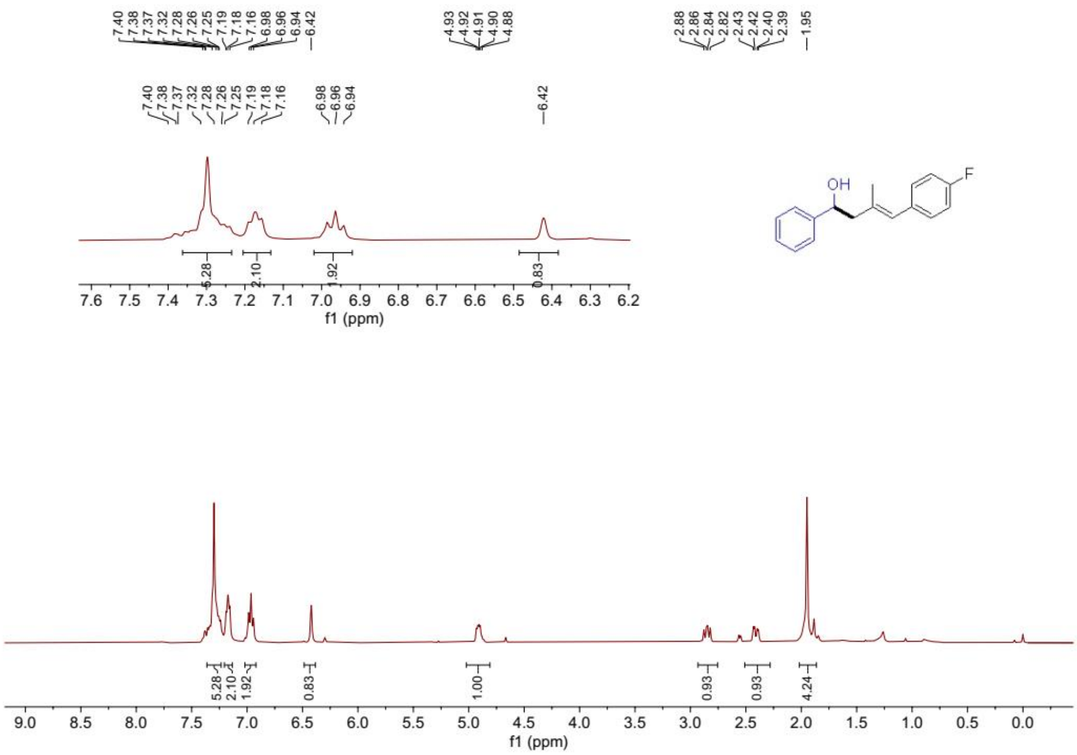

3n^{13}C NMR

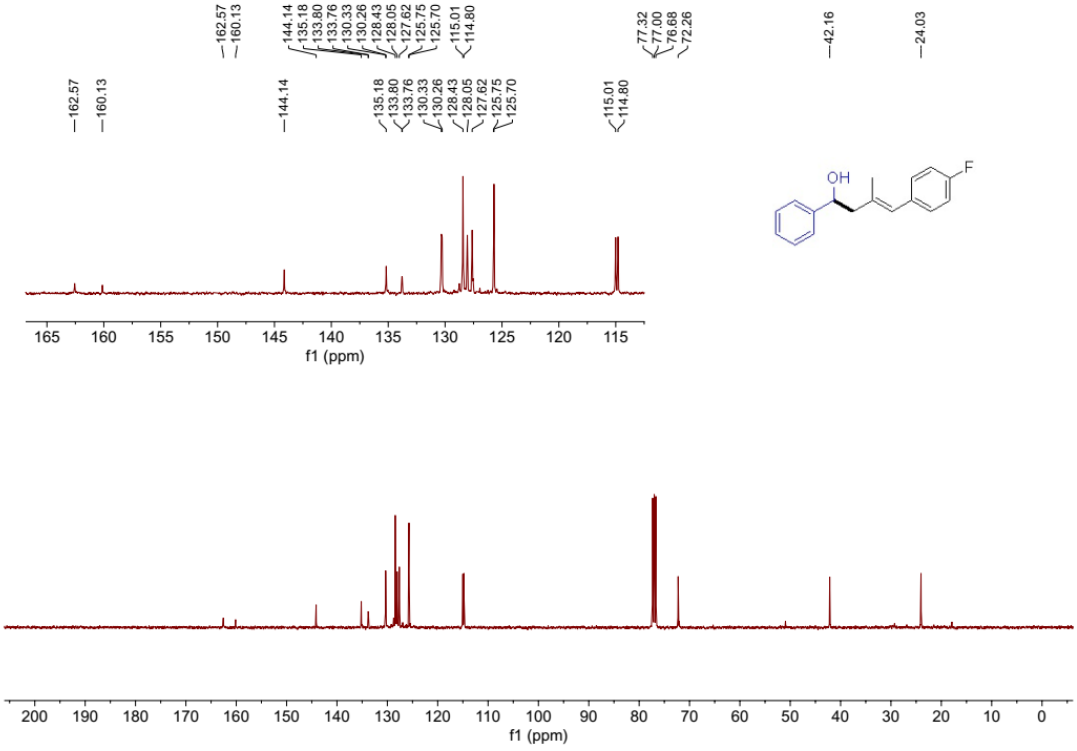

3o ^{1}H NMR

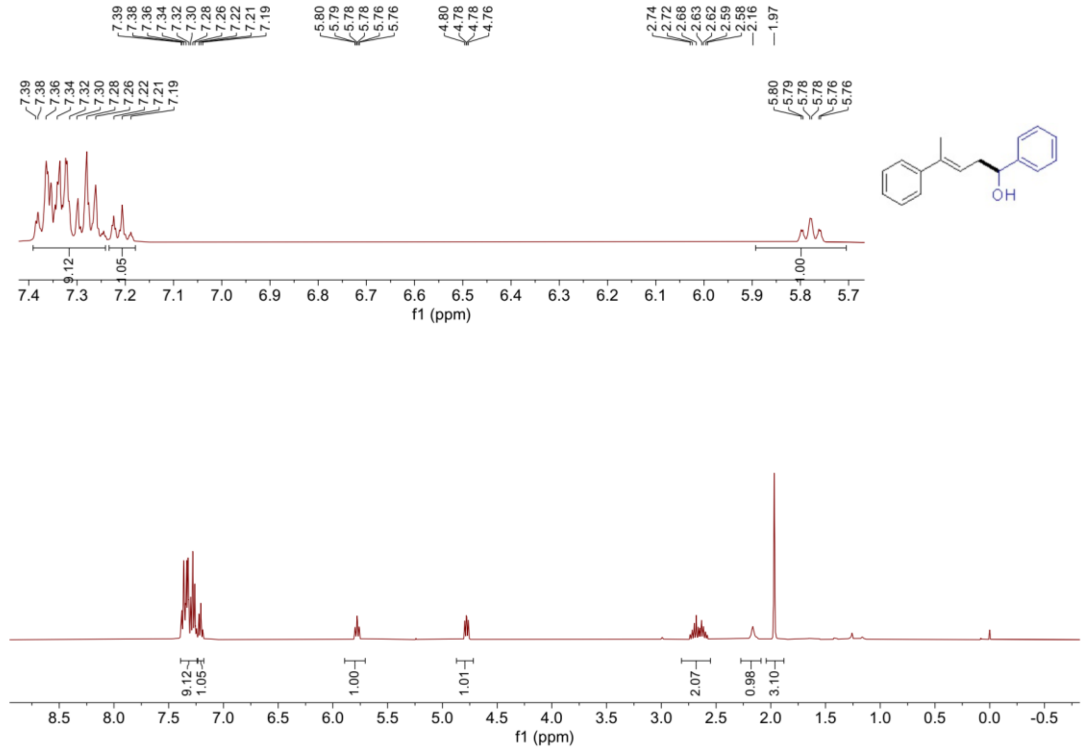

3o ^{13}C NMR

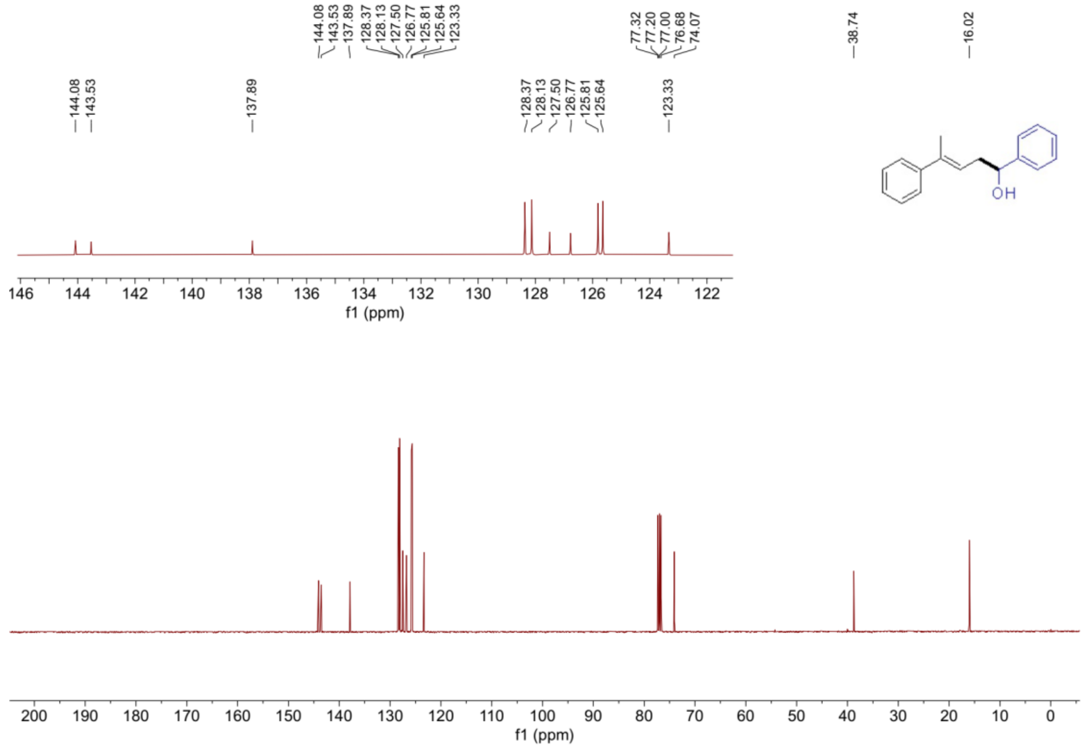

3p ^{1}H NMR

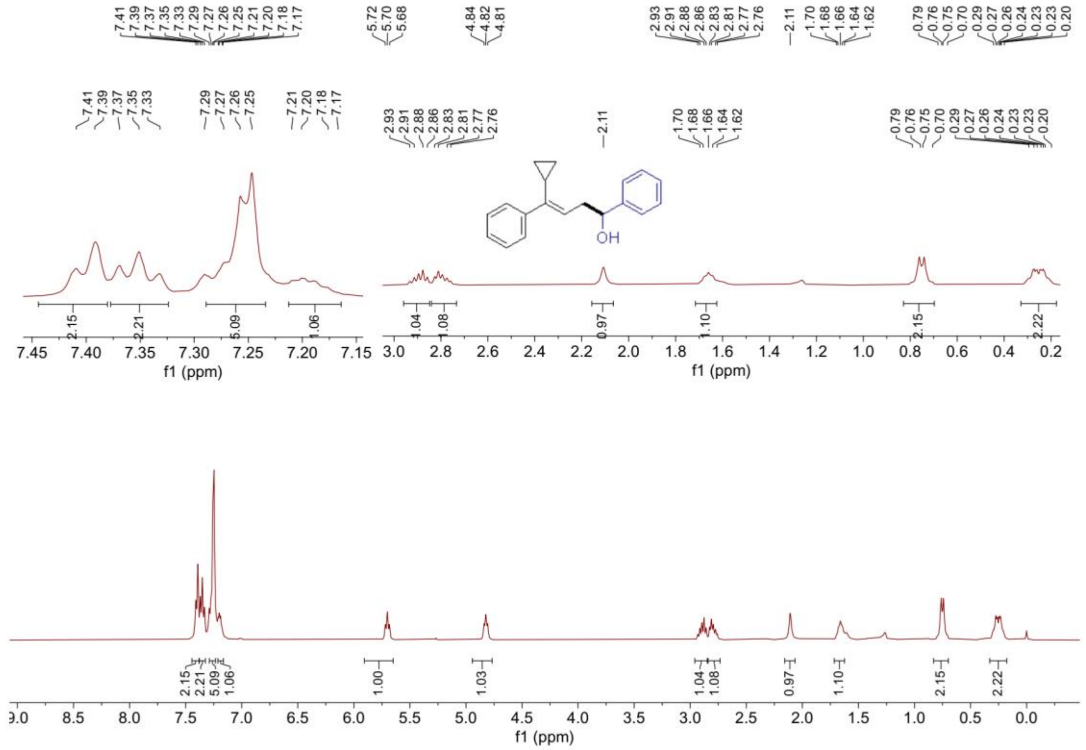

3p ^{13}C NMR

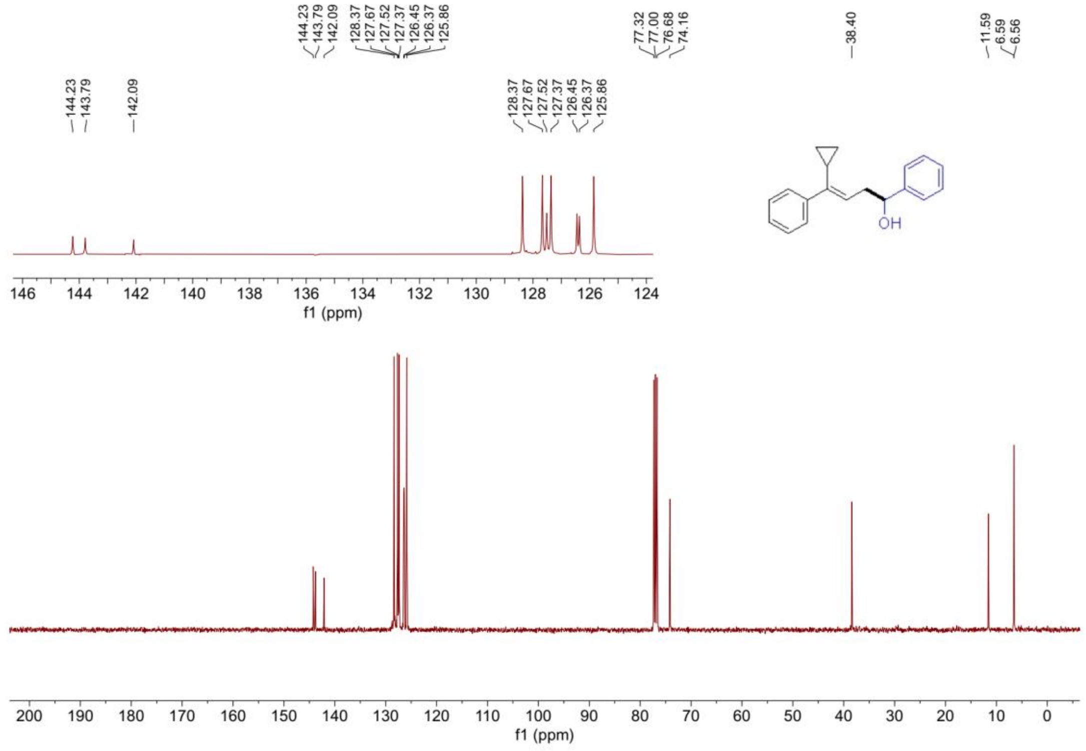

3q ^{1}H NMR

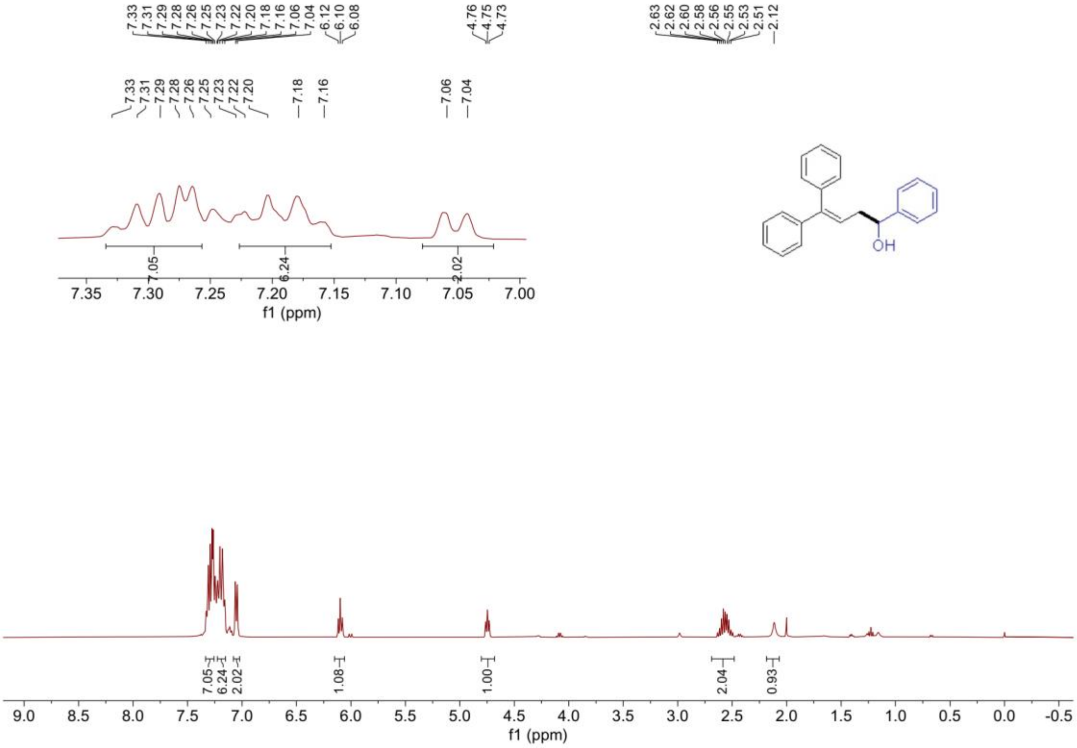

3q ^{13}C NMR

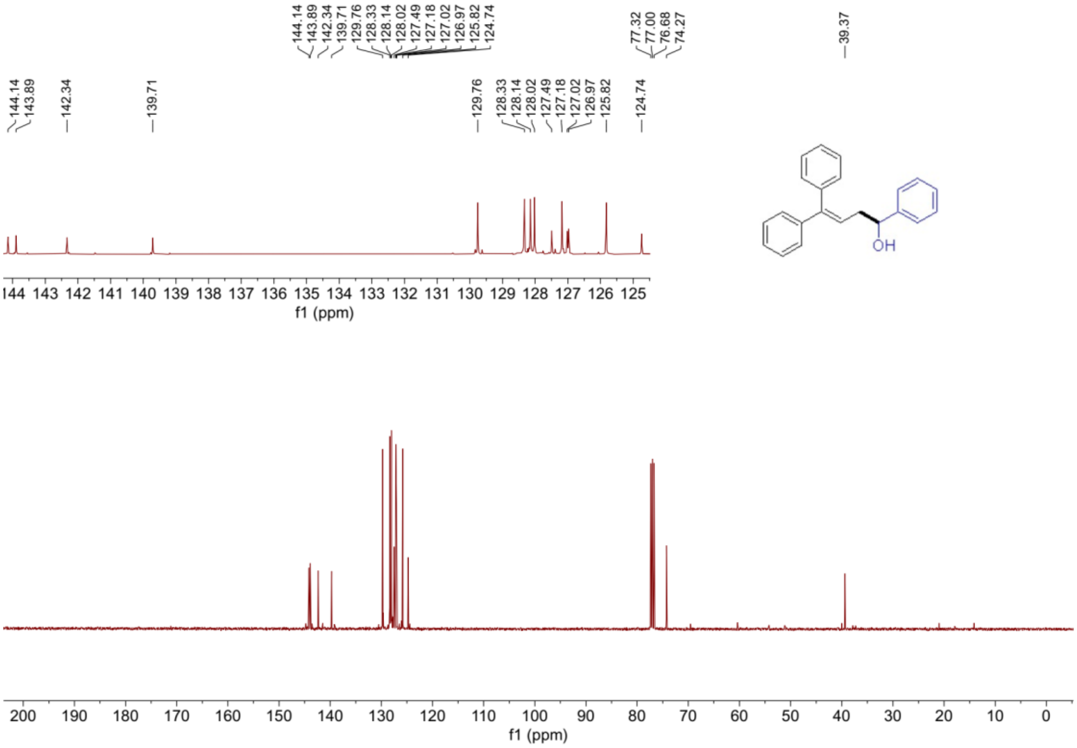

3r ^{1}H NMR

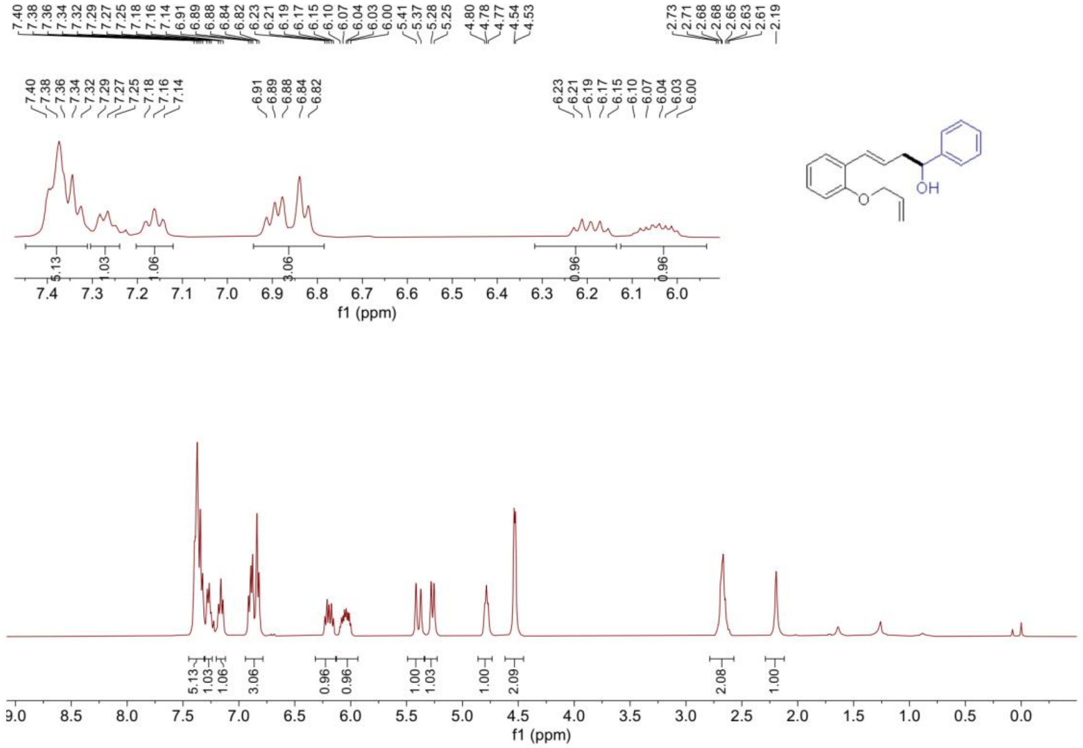

3r ^{13}C NMR

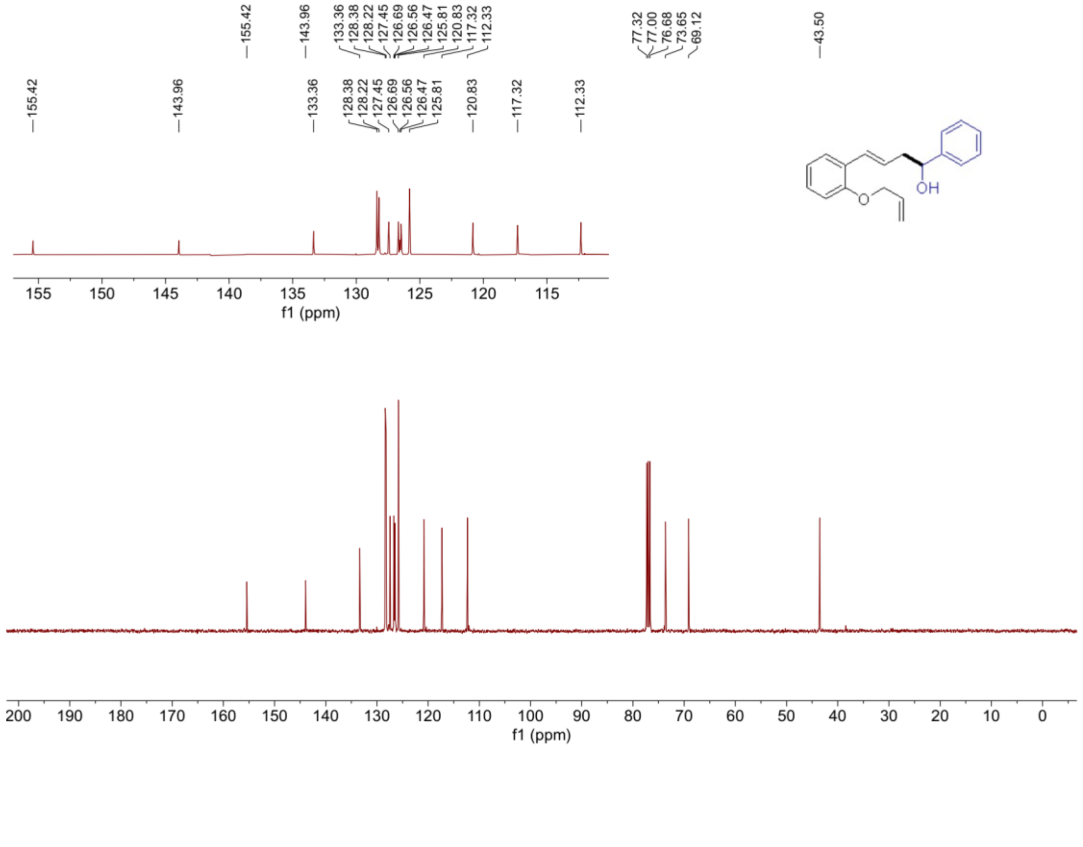

3s ^{1}H NMR

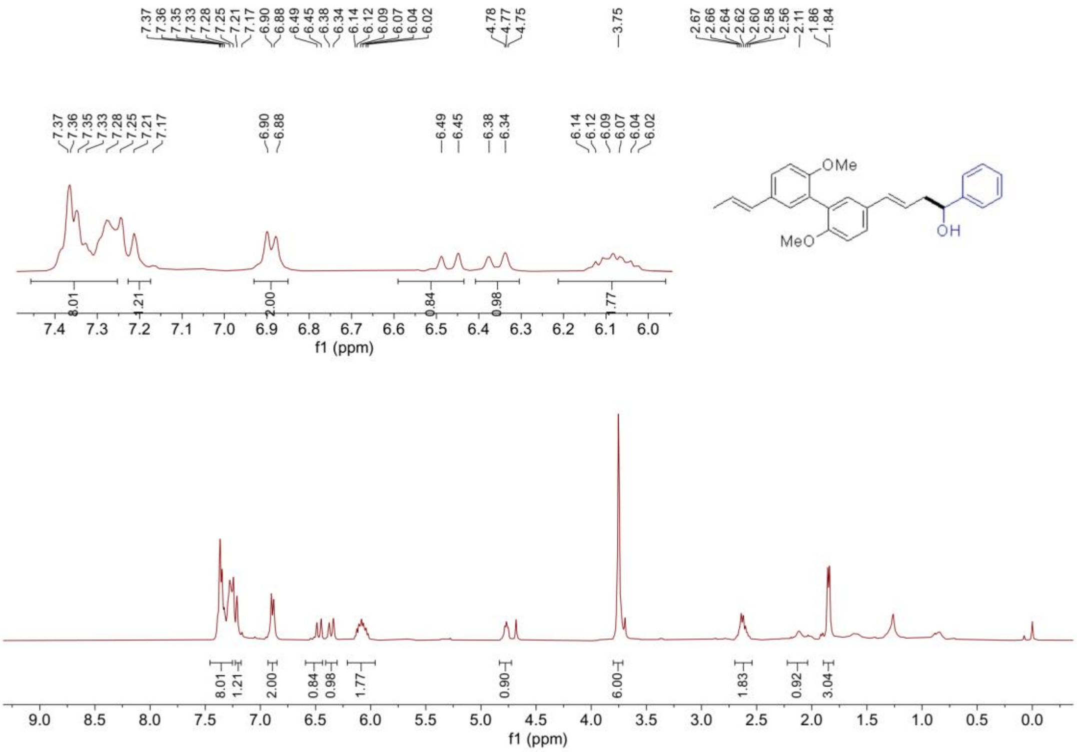

3s ^{13}C NMR

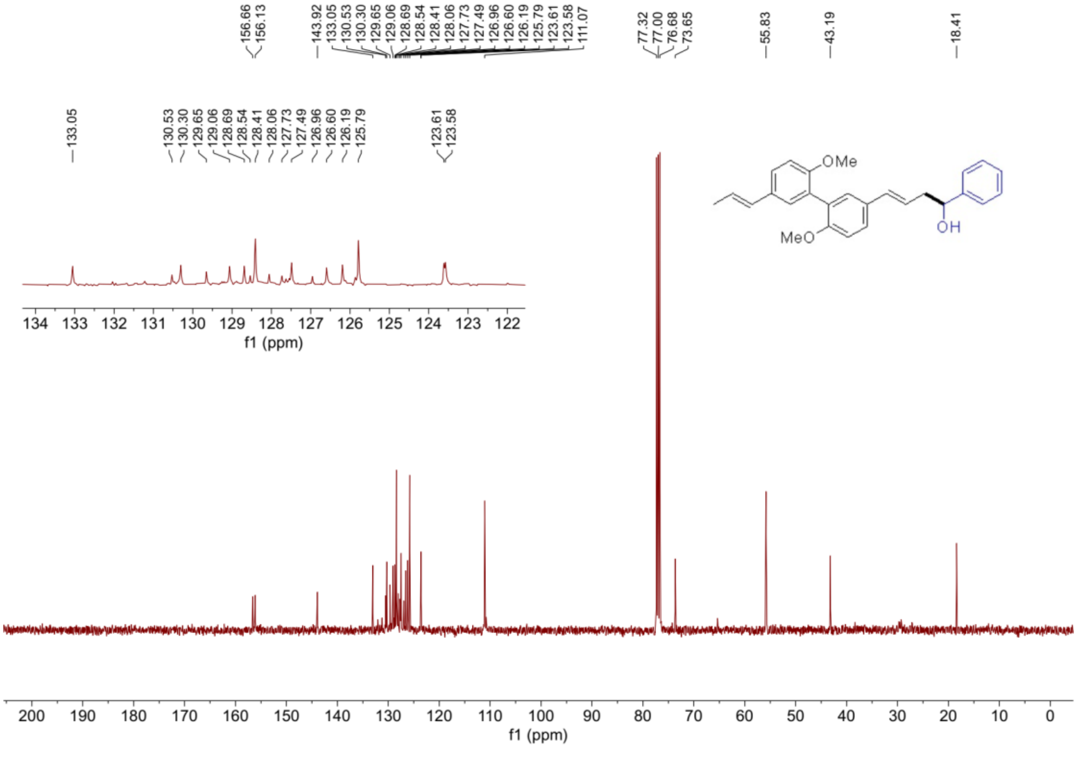

3t ^{1}H NMR

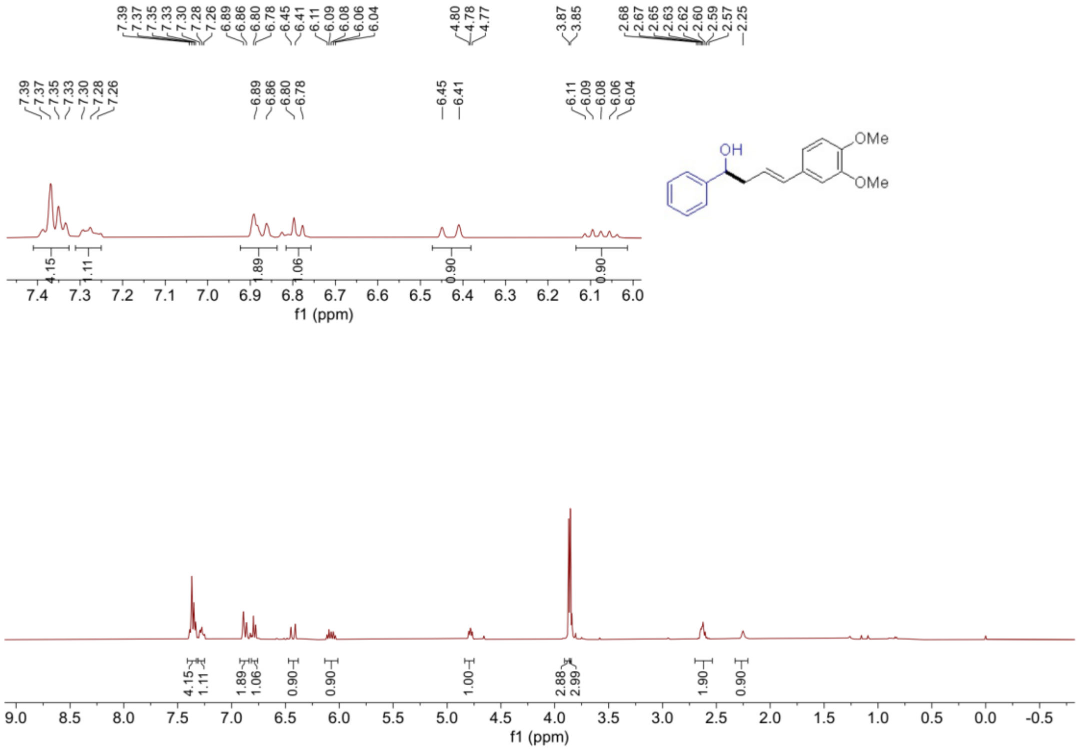

3t ^{13}C NMR

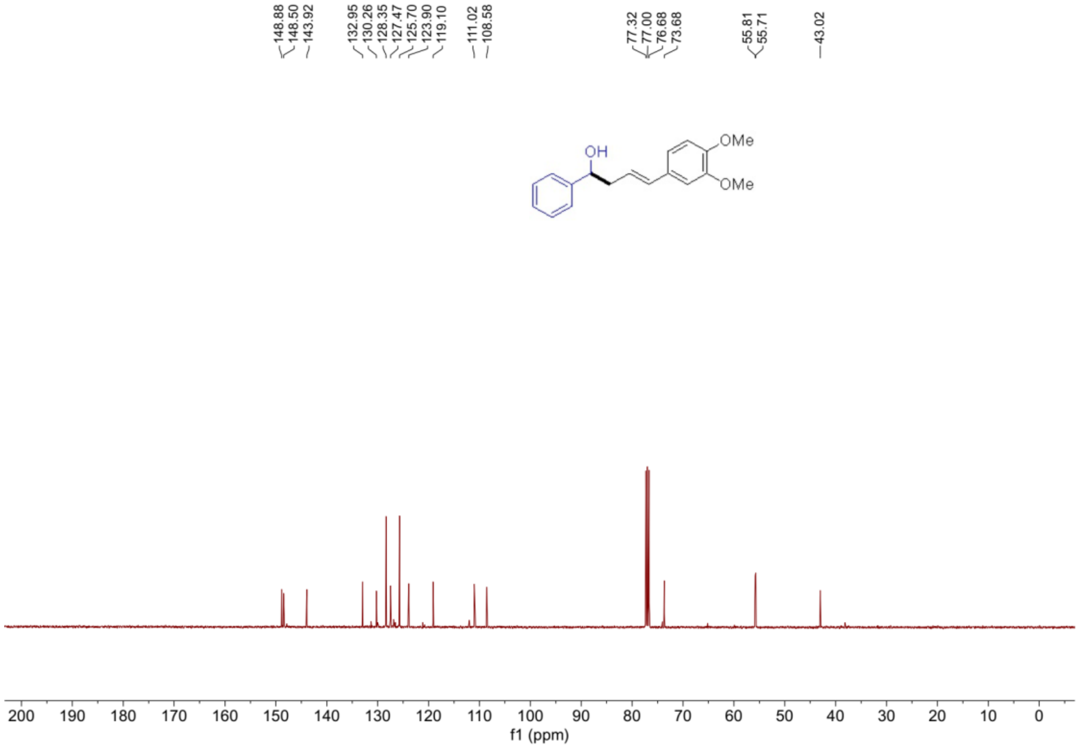

3u ^{1}H NMR

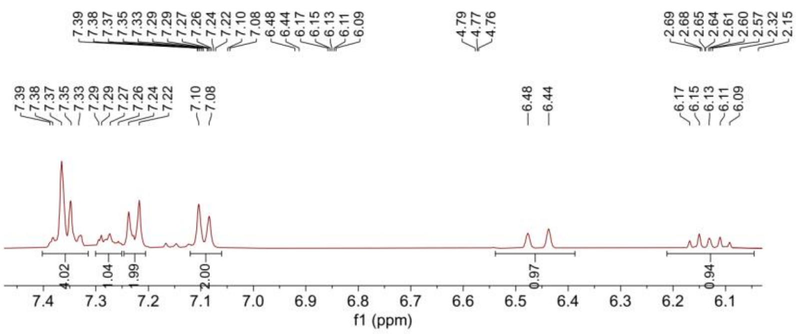

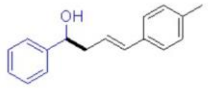

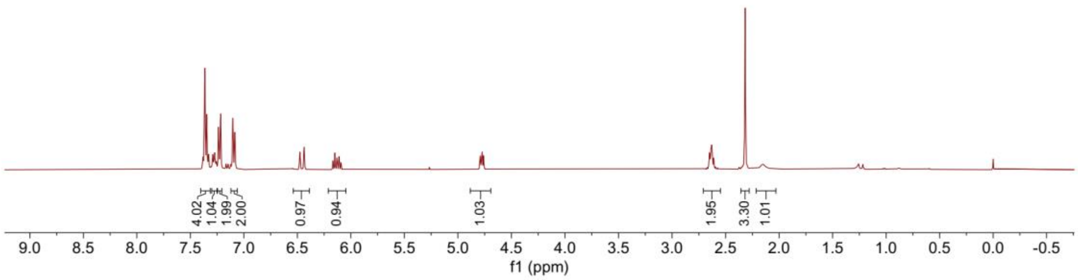

3u ^{13}C NMR

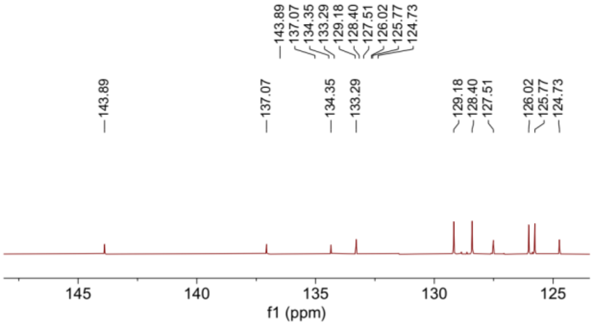

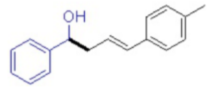

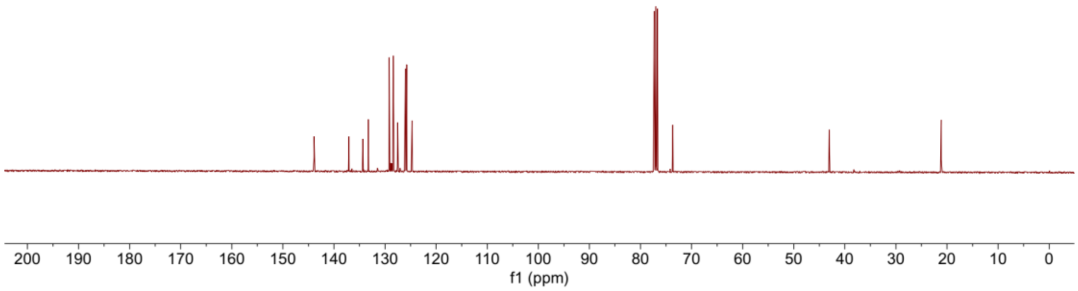

3v ^{1}H NMR

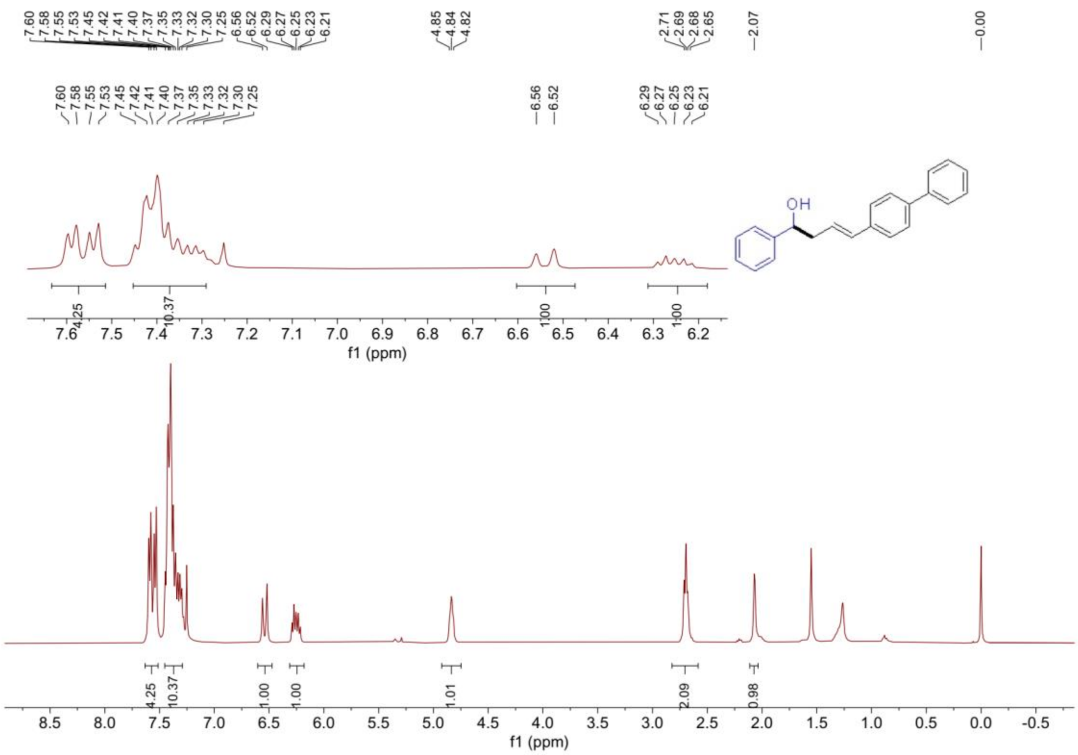

3v ^{13}C NMR

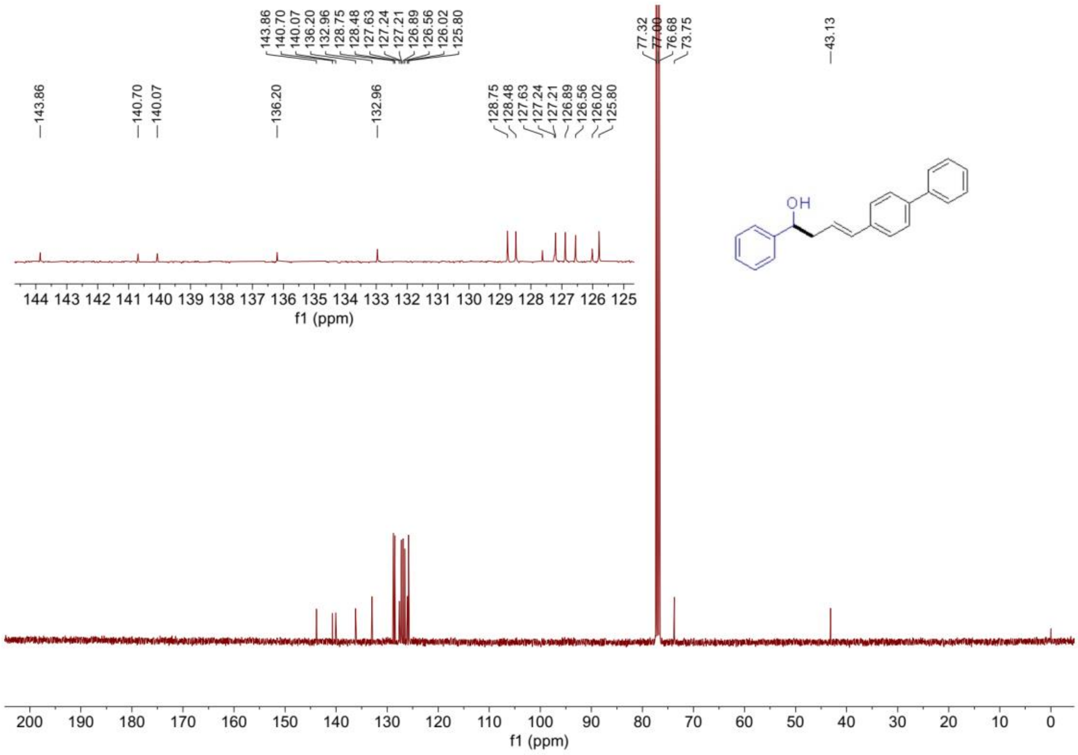

3w ^{1}H NMR

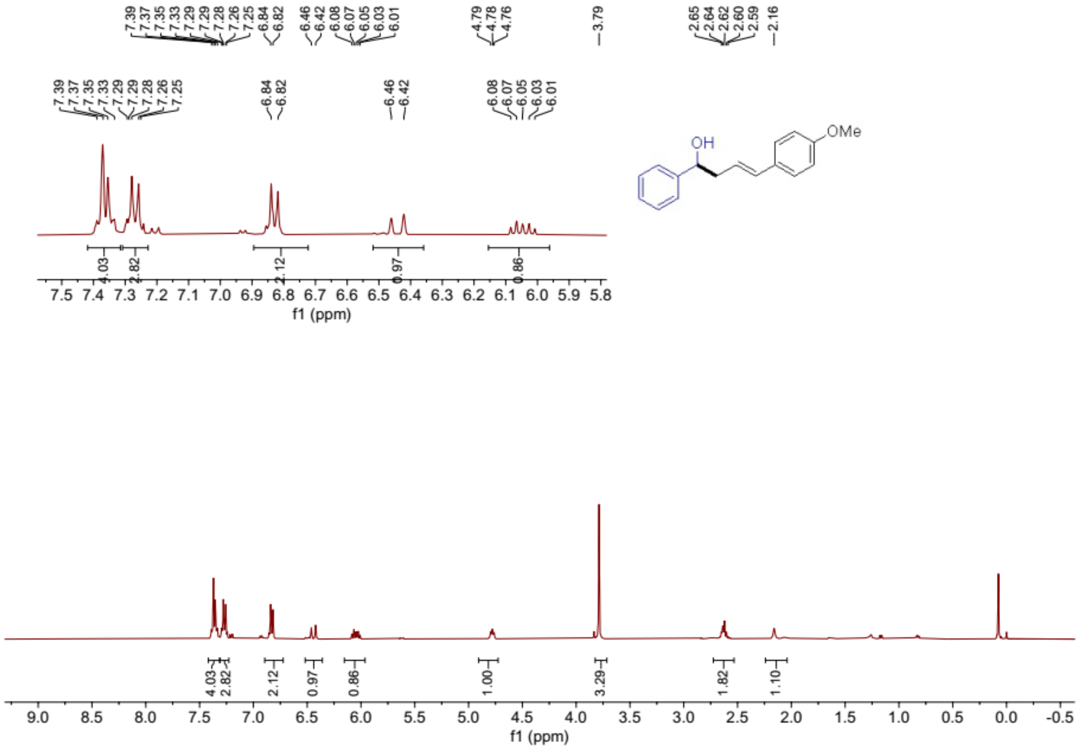

3w ^{13}C NMR

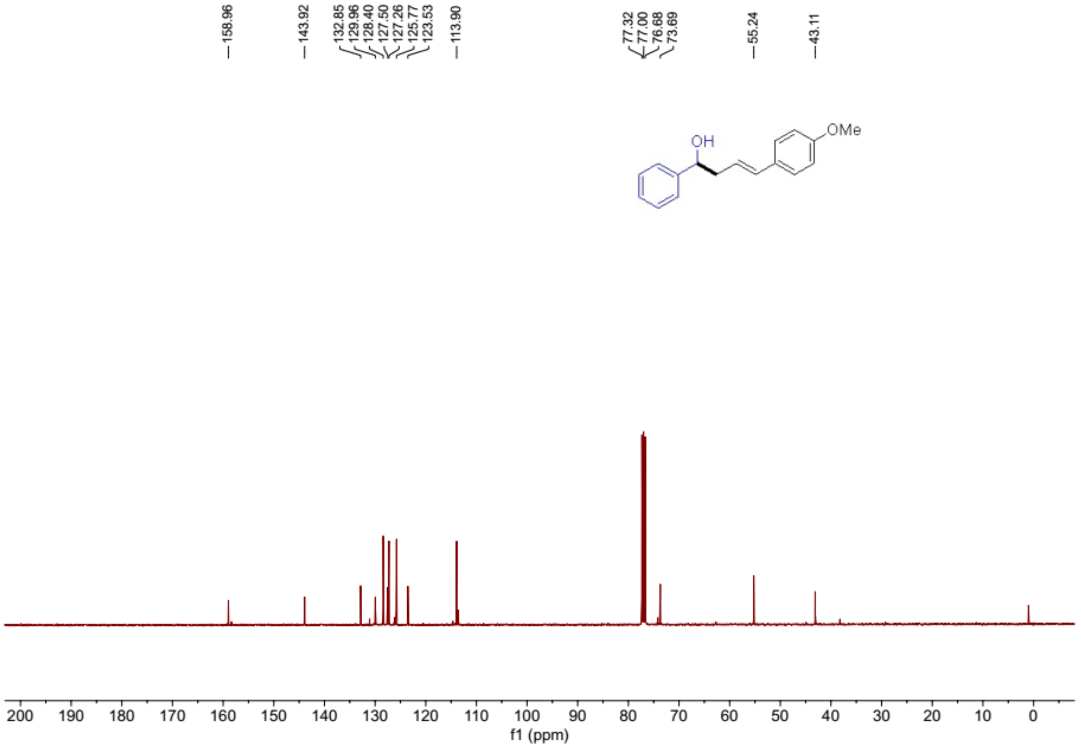

3x ^{1}H NMR

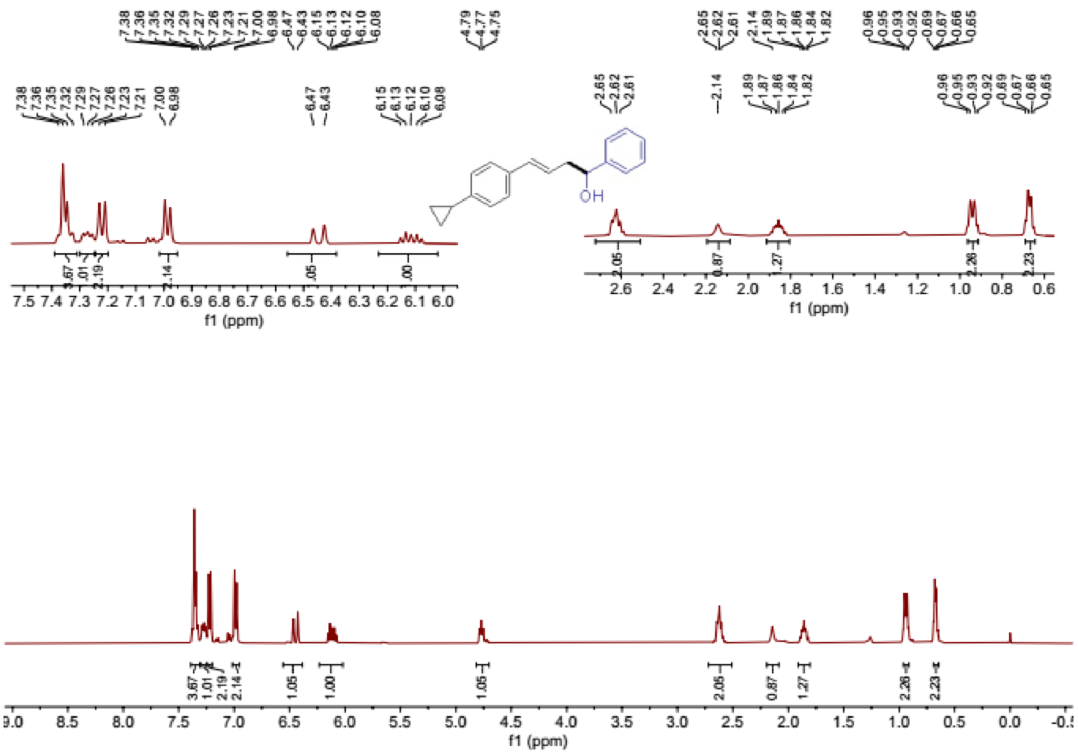

3x ^{13}C NMR

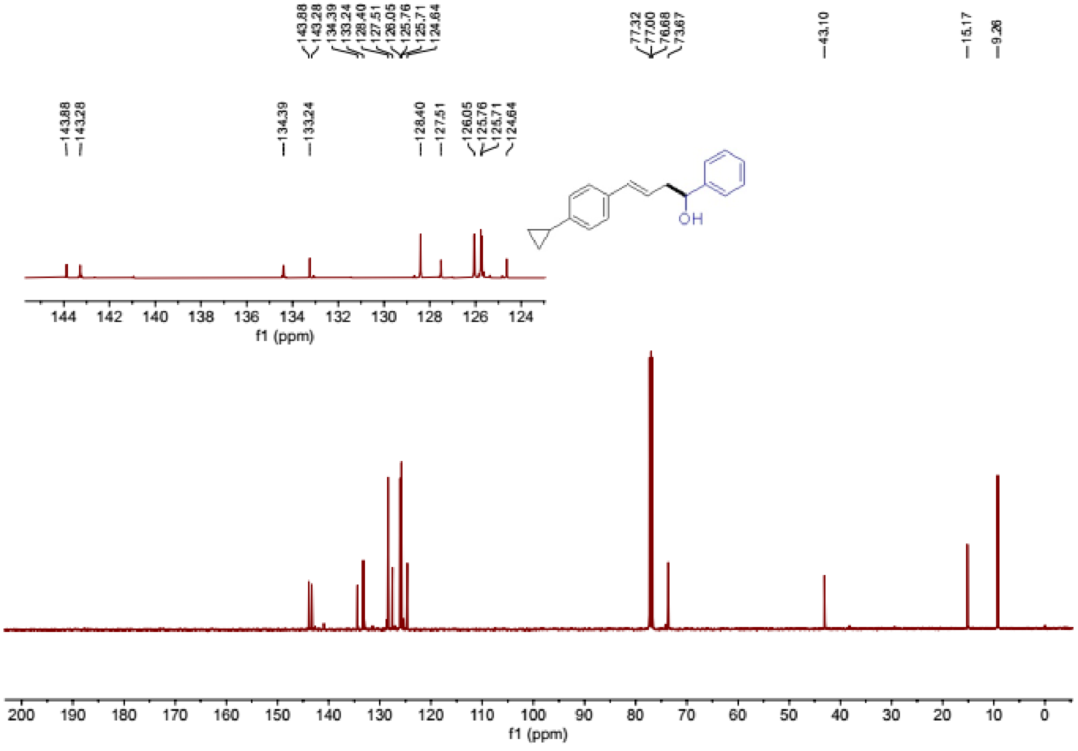

3y ^{1}H NMR

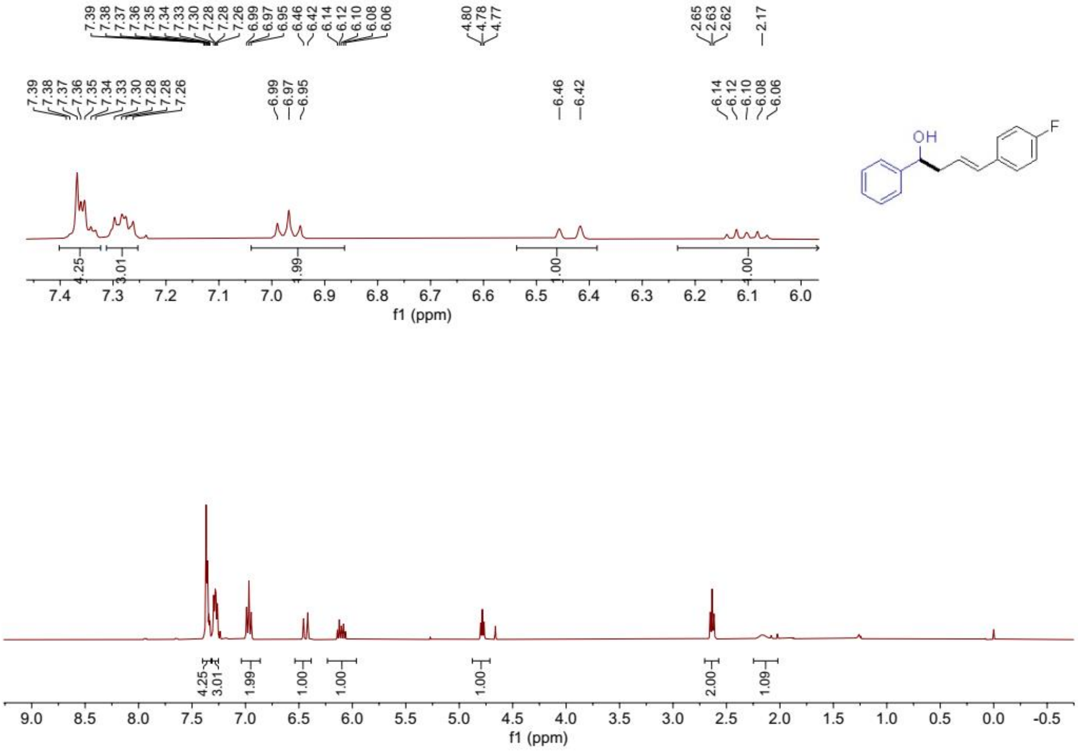

3y ^{13}C NMR

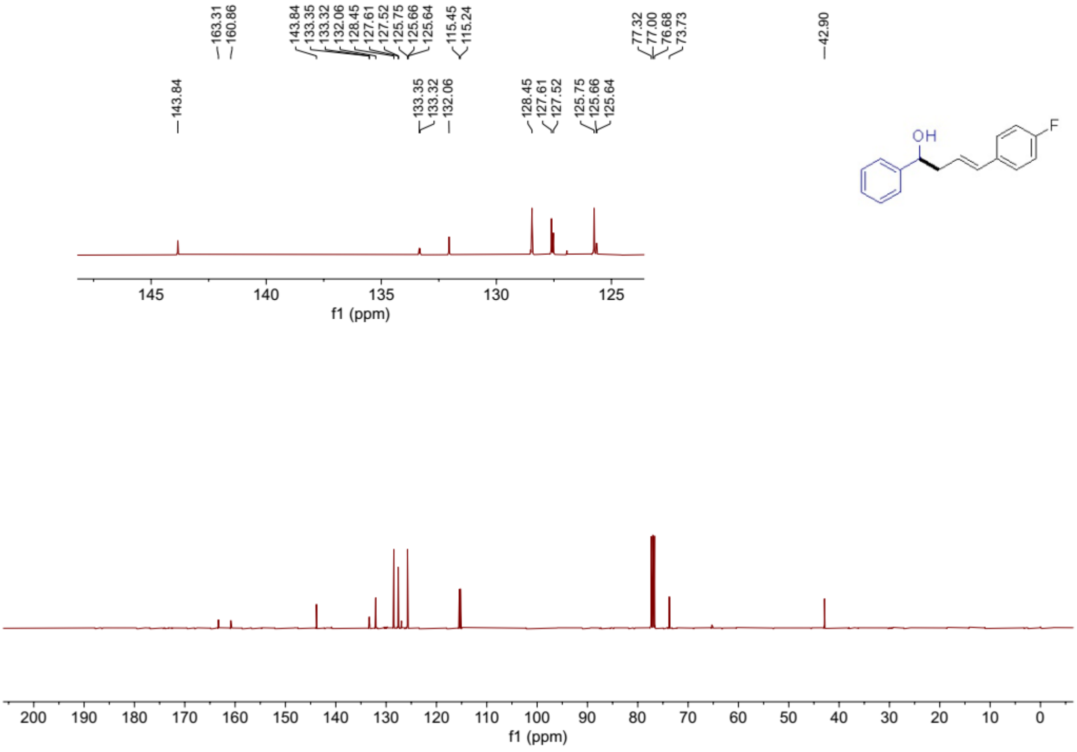

3z ^{1}H NMR

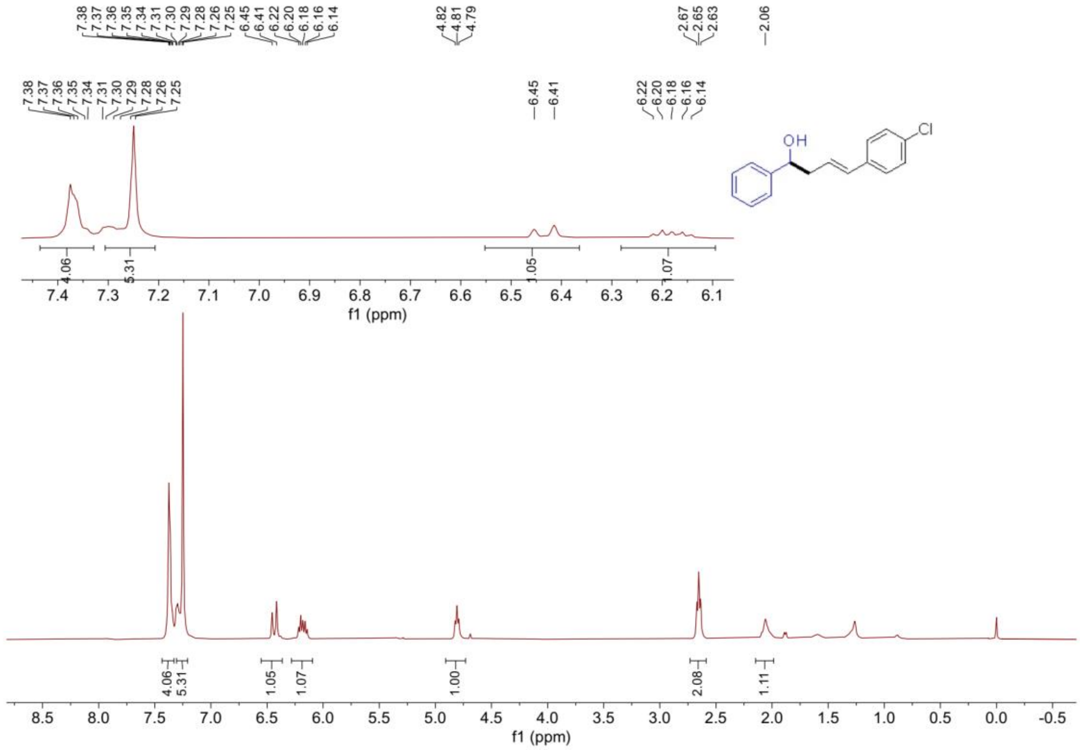

3z ^{13}C NMR

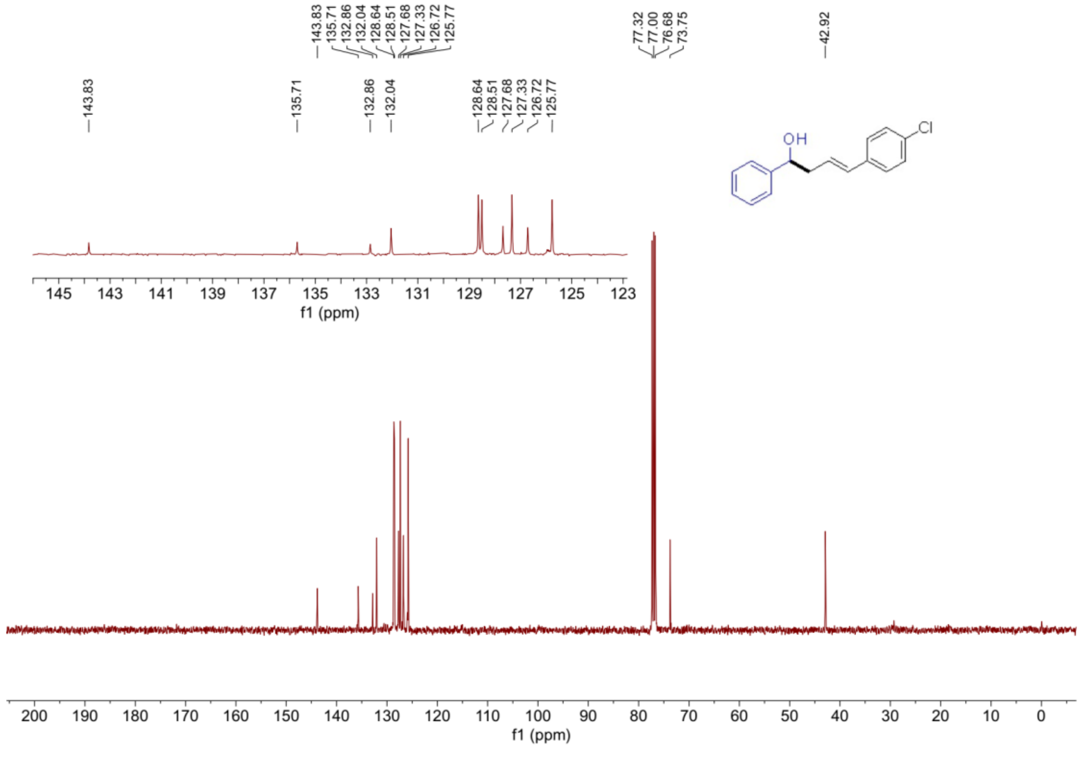

3aa ^{1}H NMR

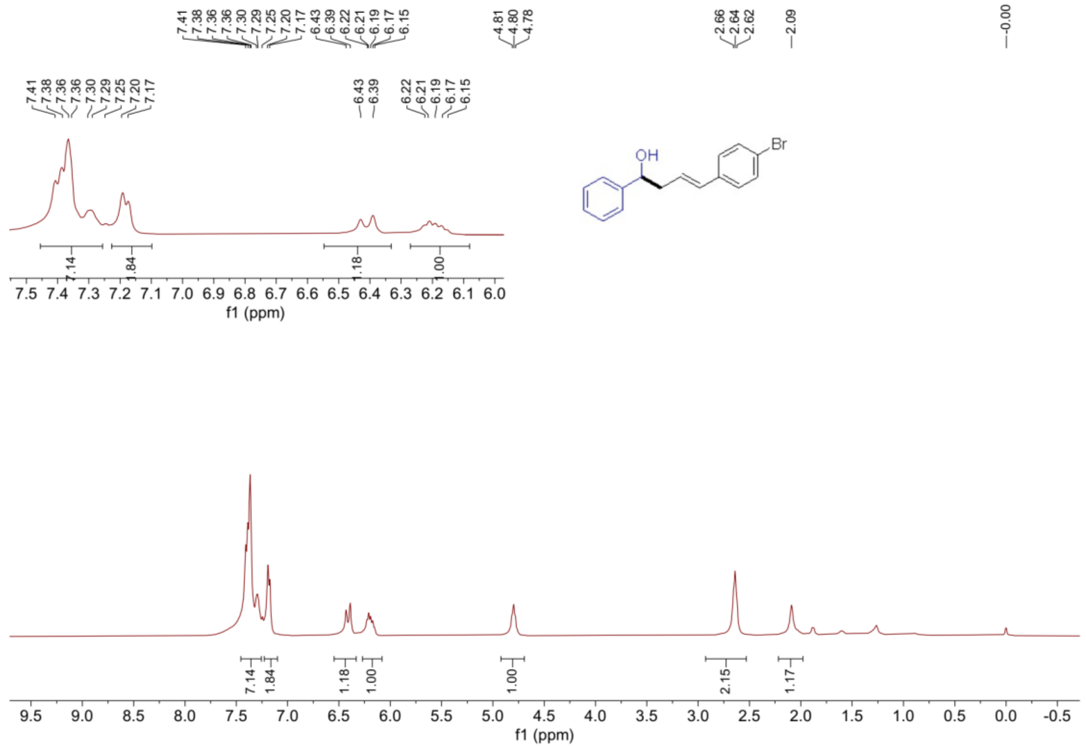

3aa ^{13}C NMR

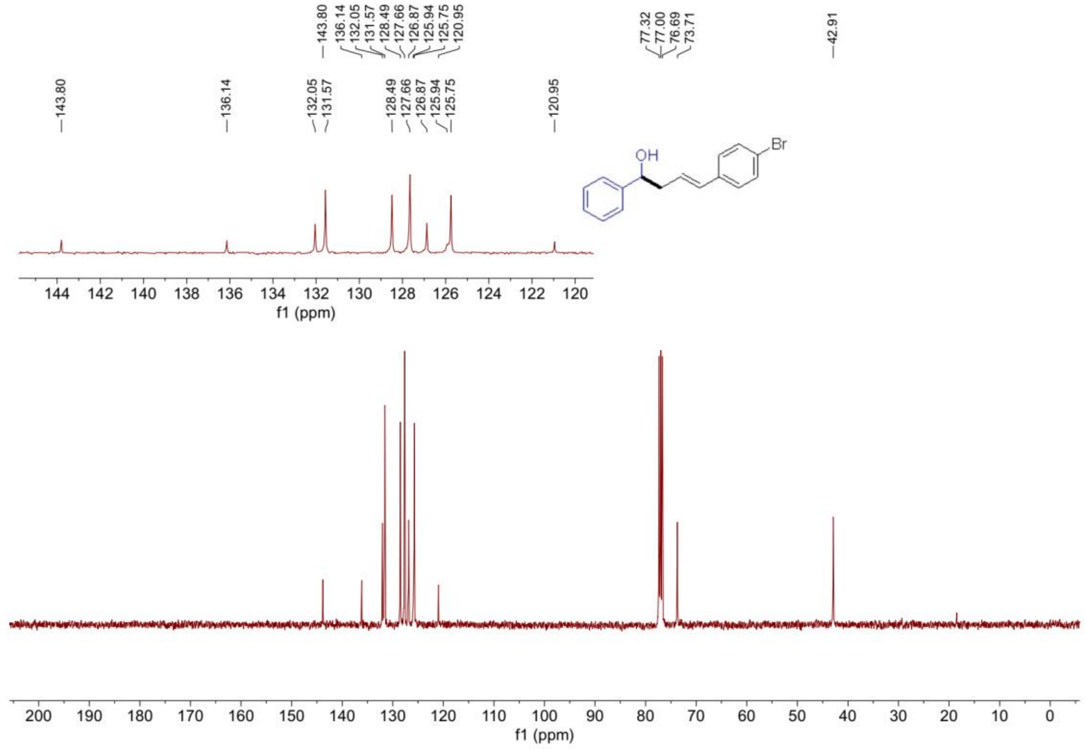

3ab ^{1}H NMR

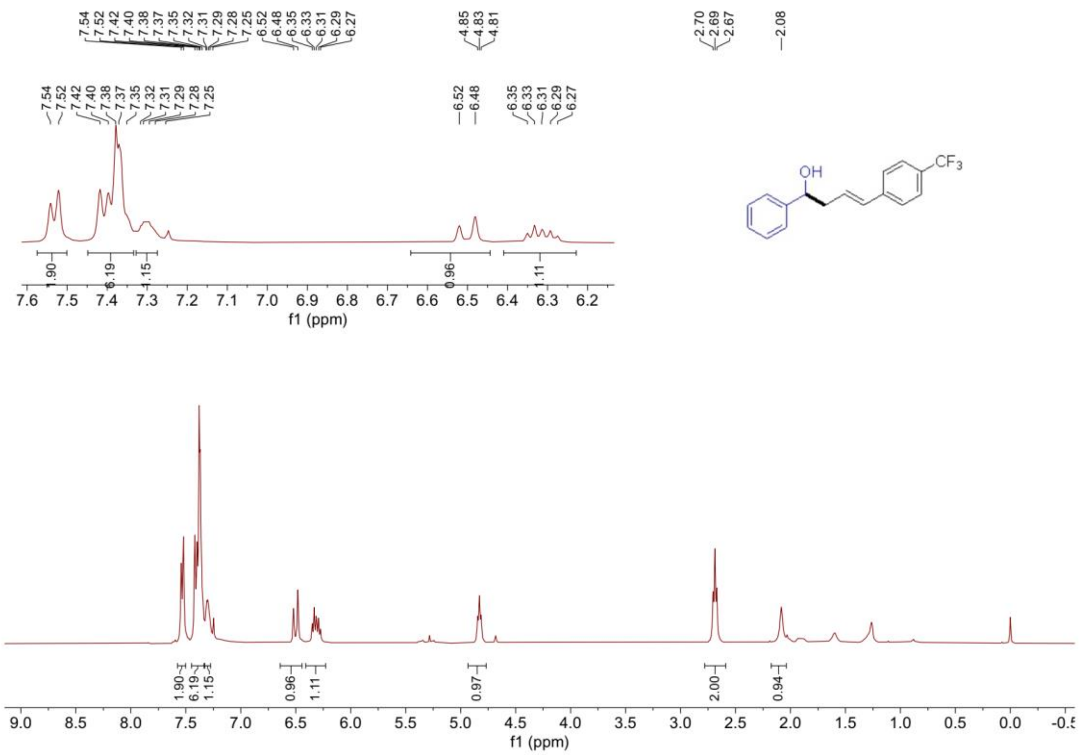

3ab ^{13}C NMR

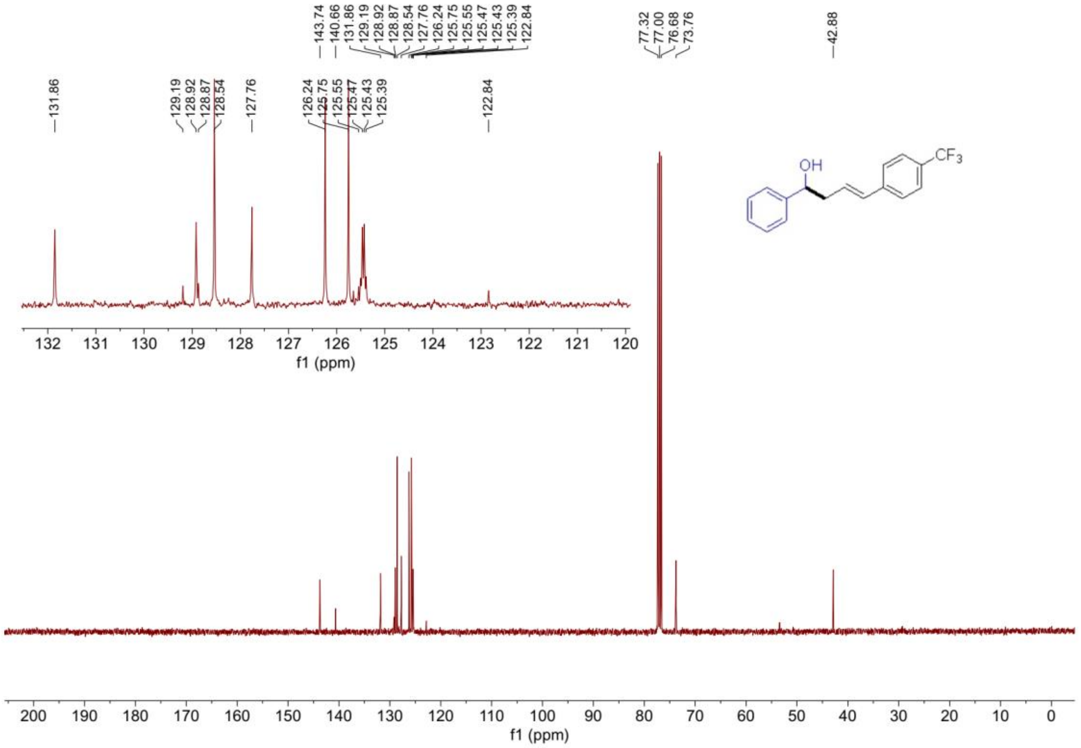

3ac ^{1}H NMR

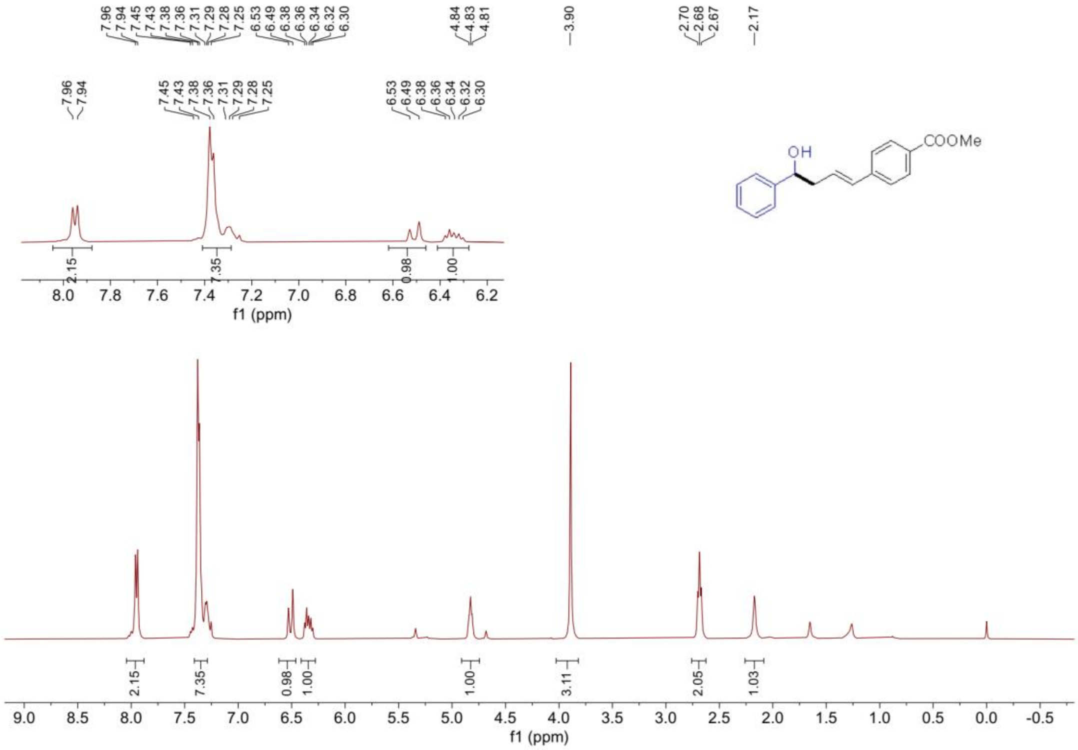

3ac ^{13}C NMR

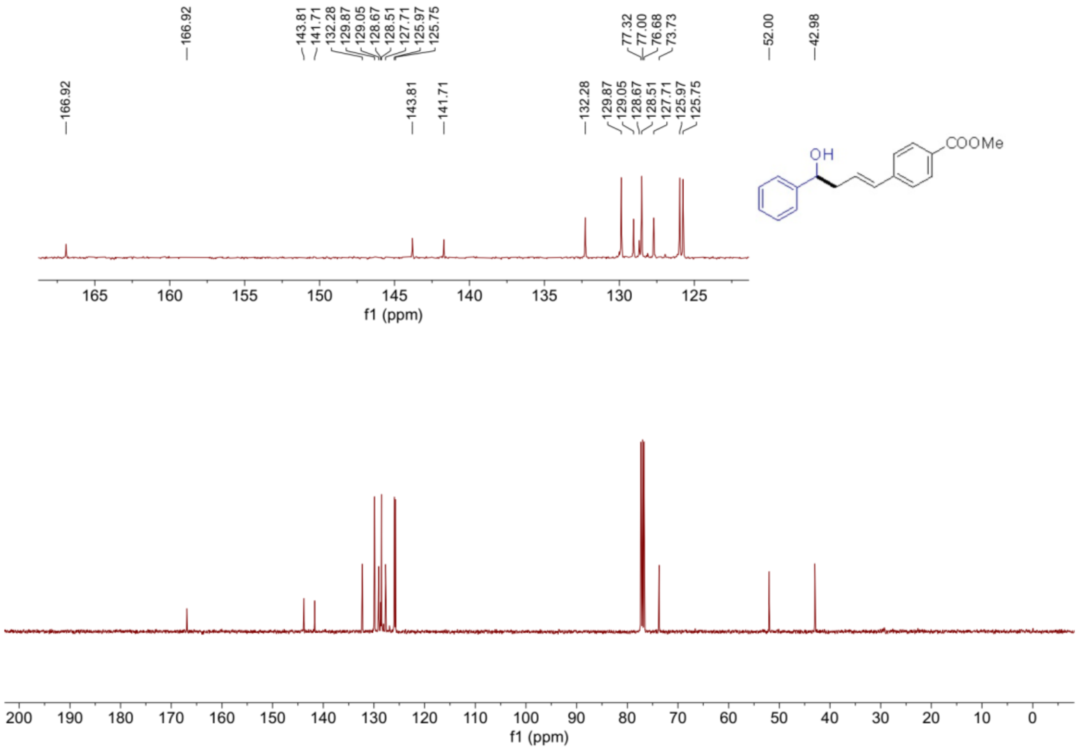

3ad ^{1}H NMR

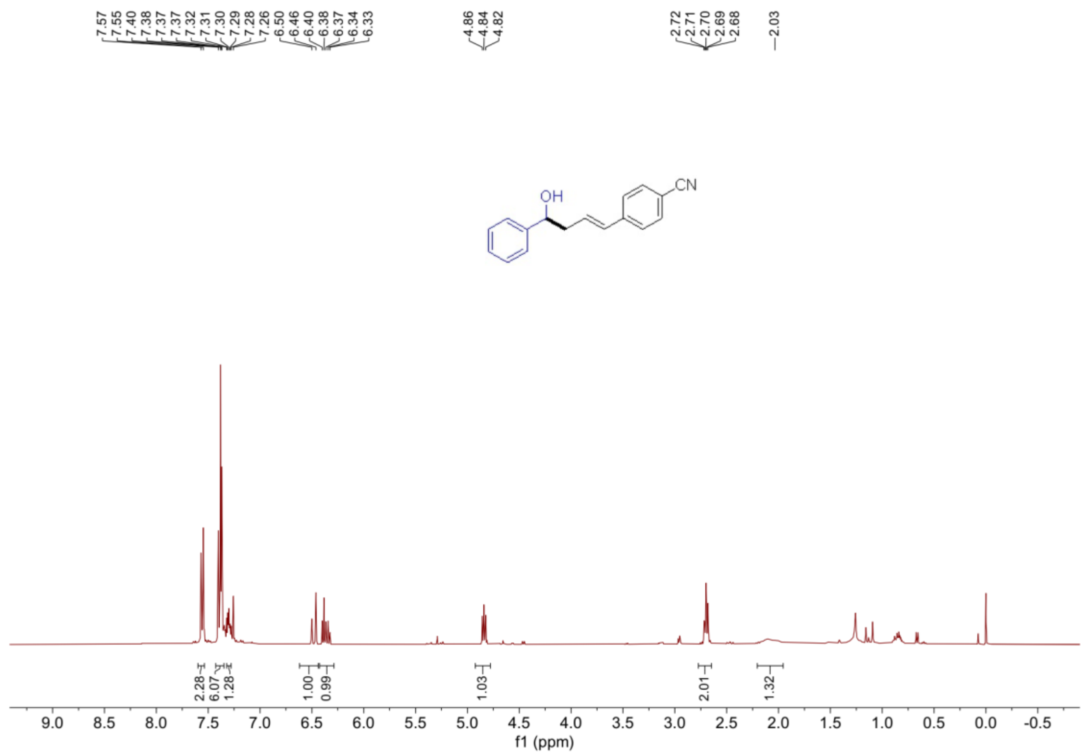

3ad ^{13}C NMR

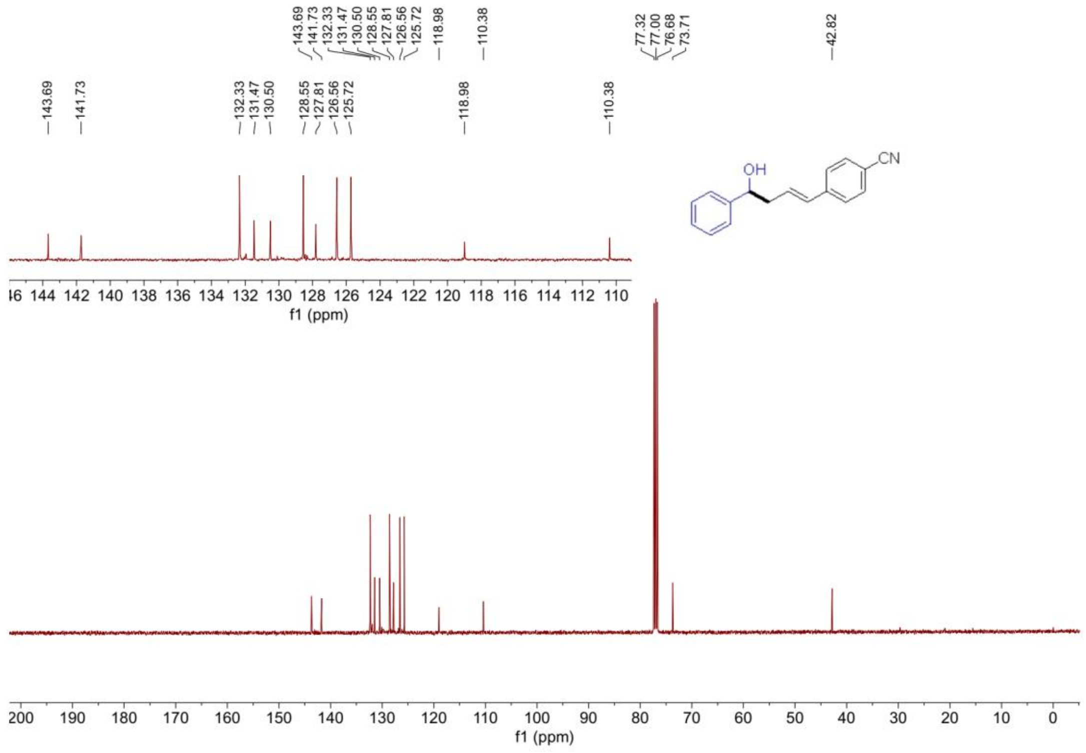

3ae ^{1}H NMR

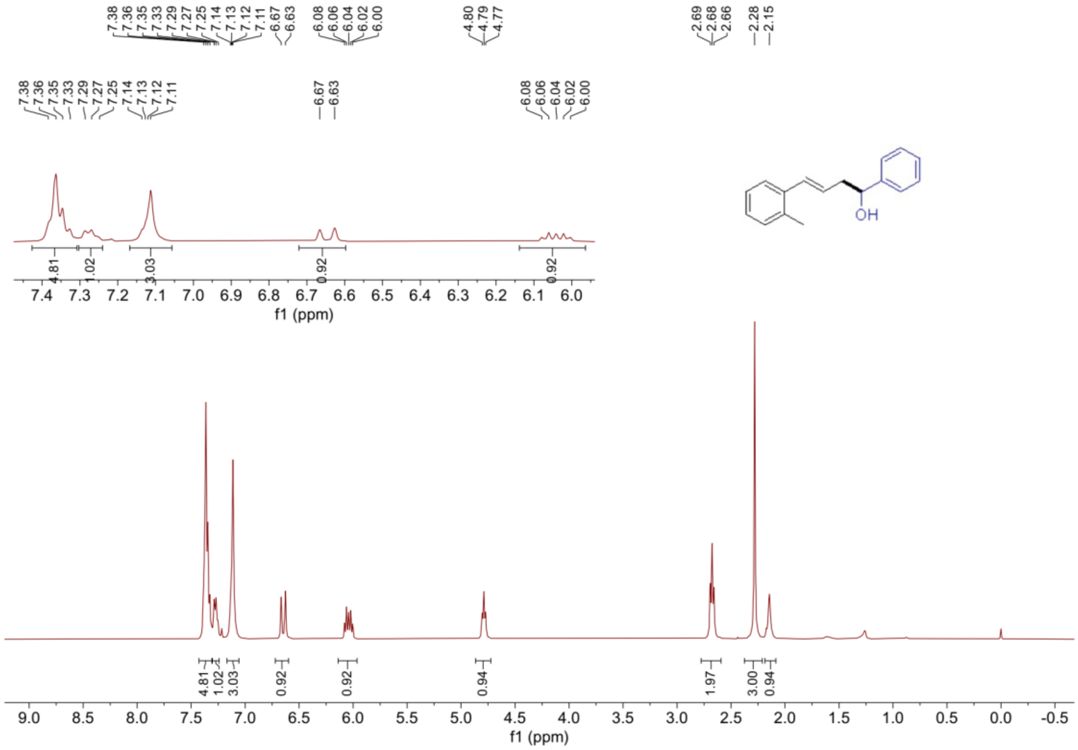

3ae ^{13}C NMR

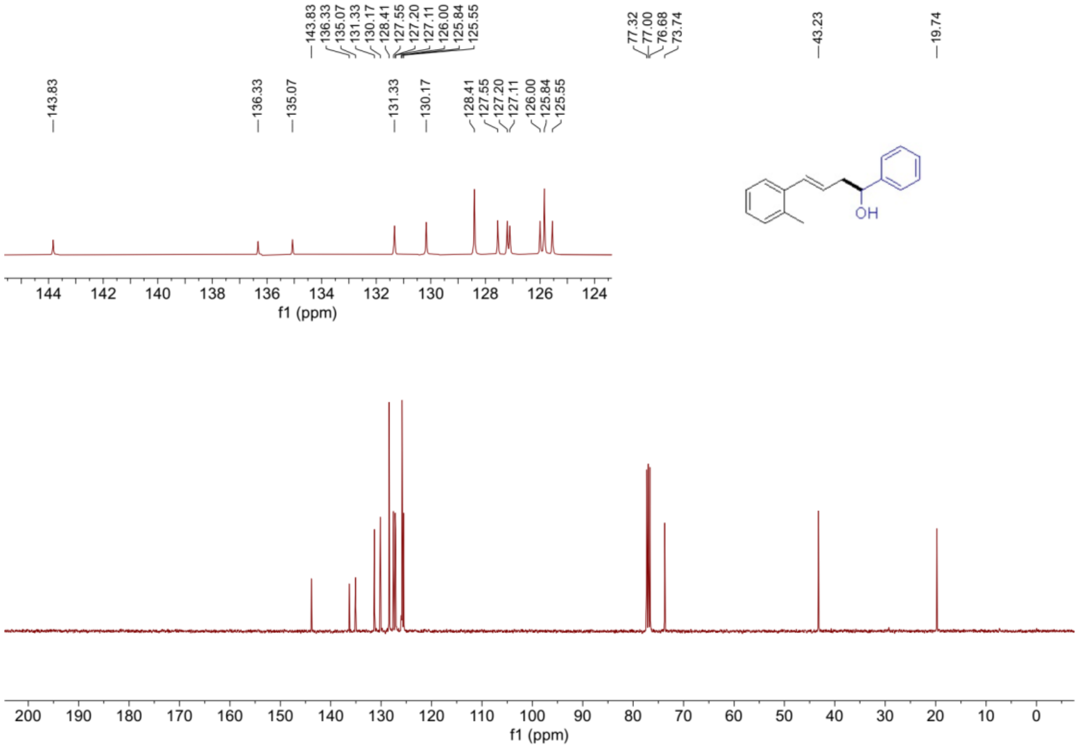

3af ^{1}H NMR

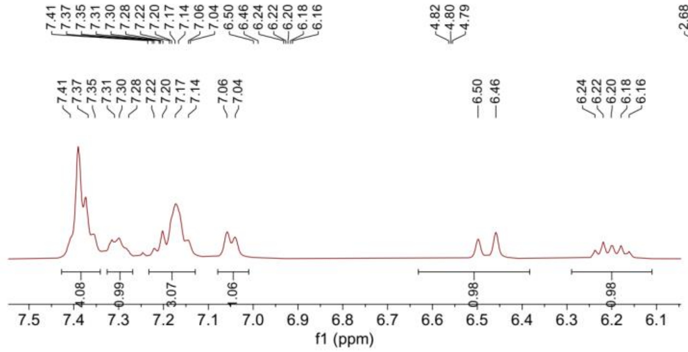

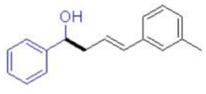

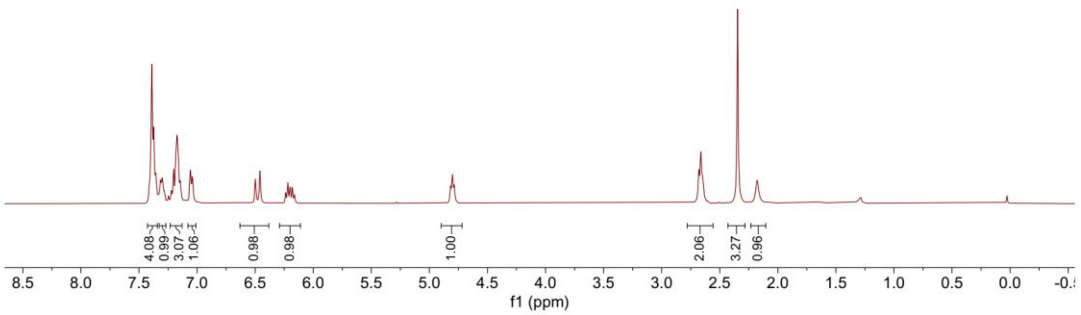

3af ^{13}C NMR

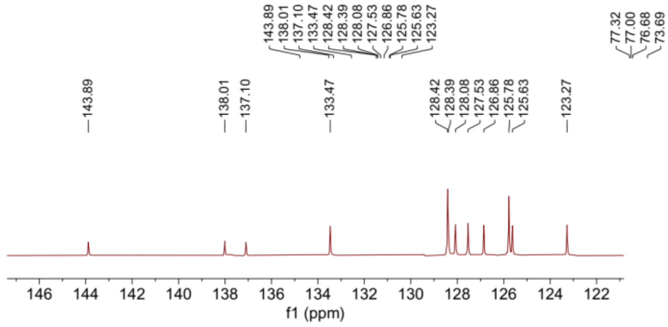

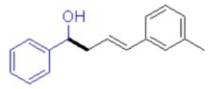

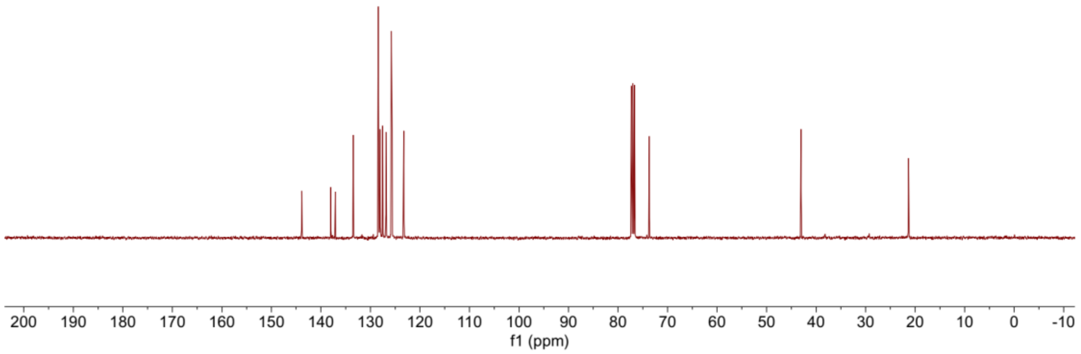

3ag ^{1}H NMR

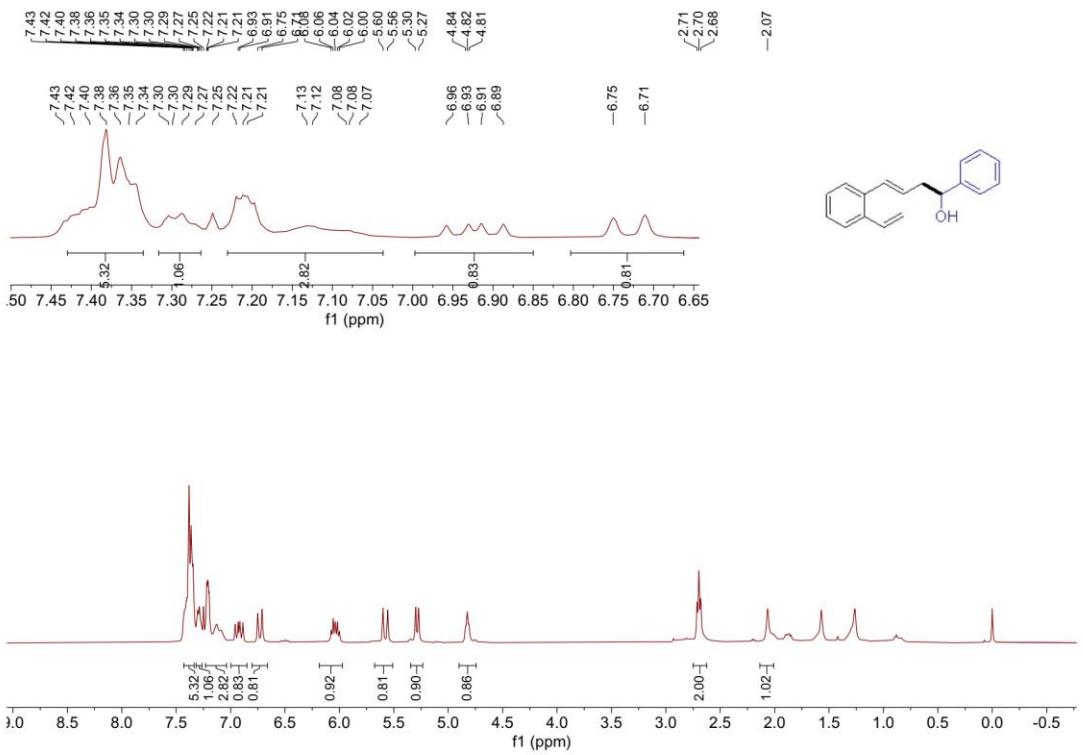

3ag ^{13}C NMR

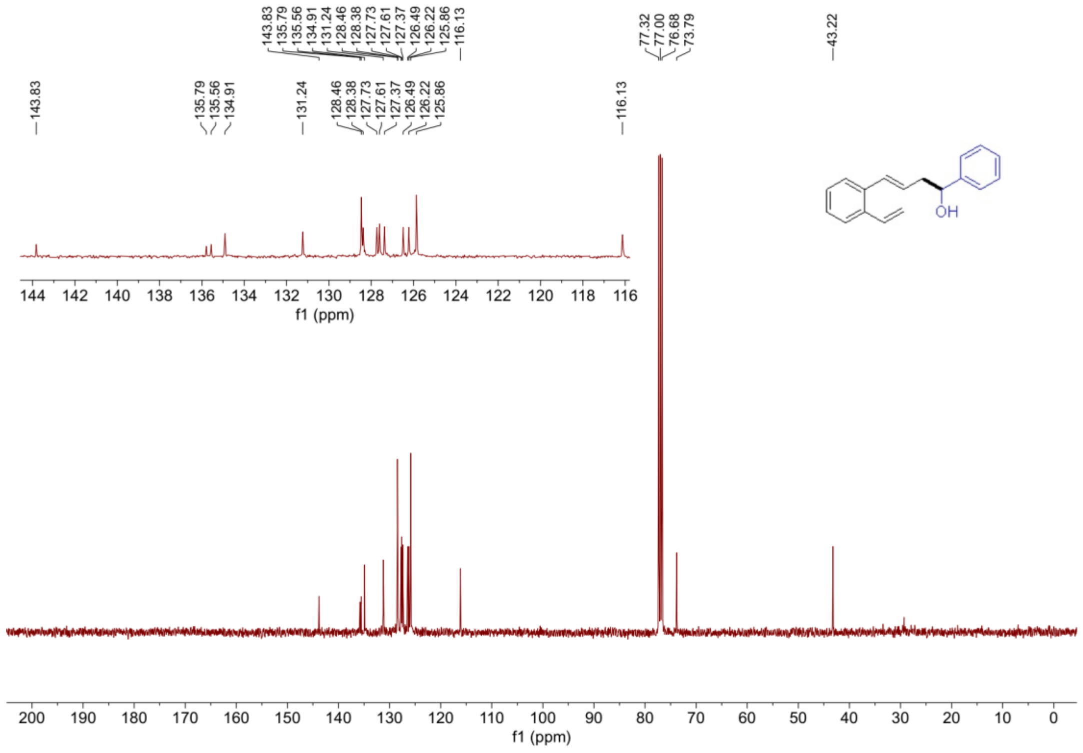

3ah ^{1}H NMR

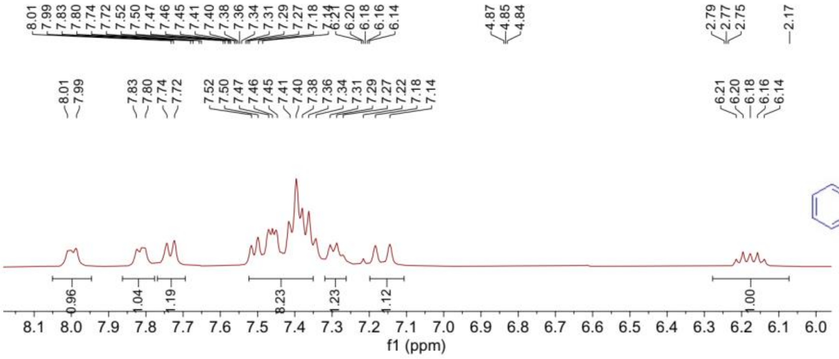

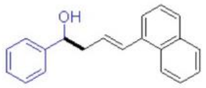

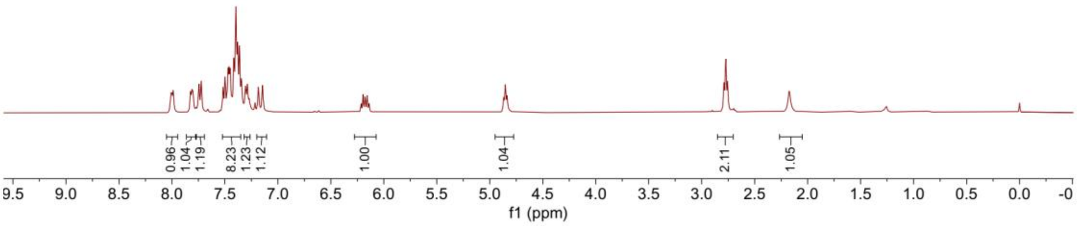

3ah ^{13}C NMR

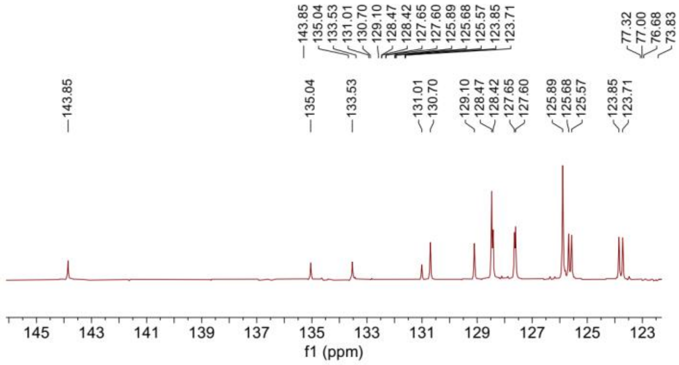

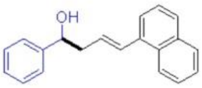

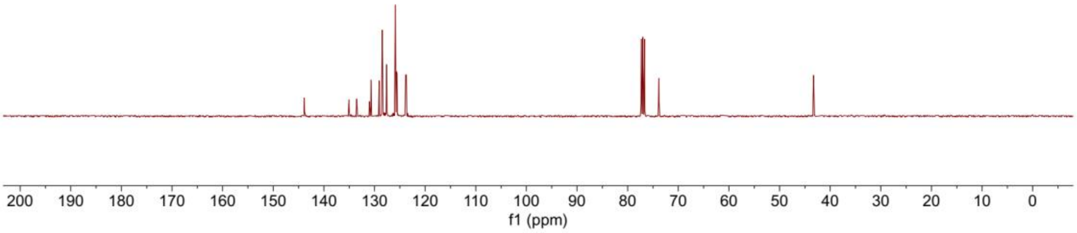

3ai ^{1}H NMR

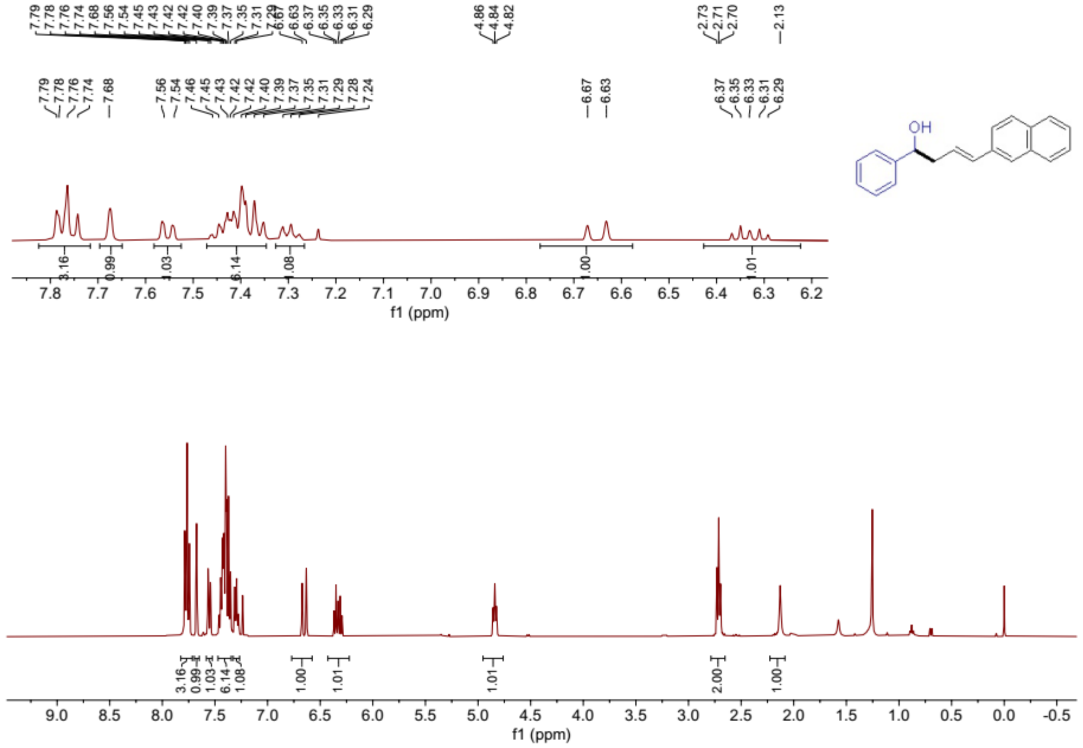

3ai ^{13}C NMR

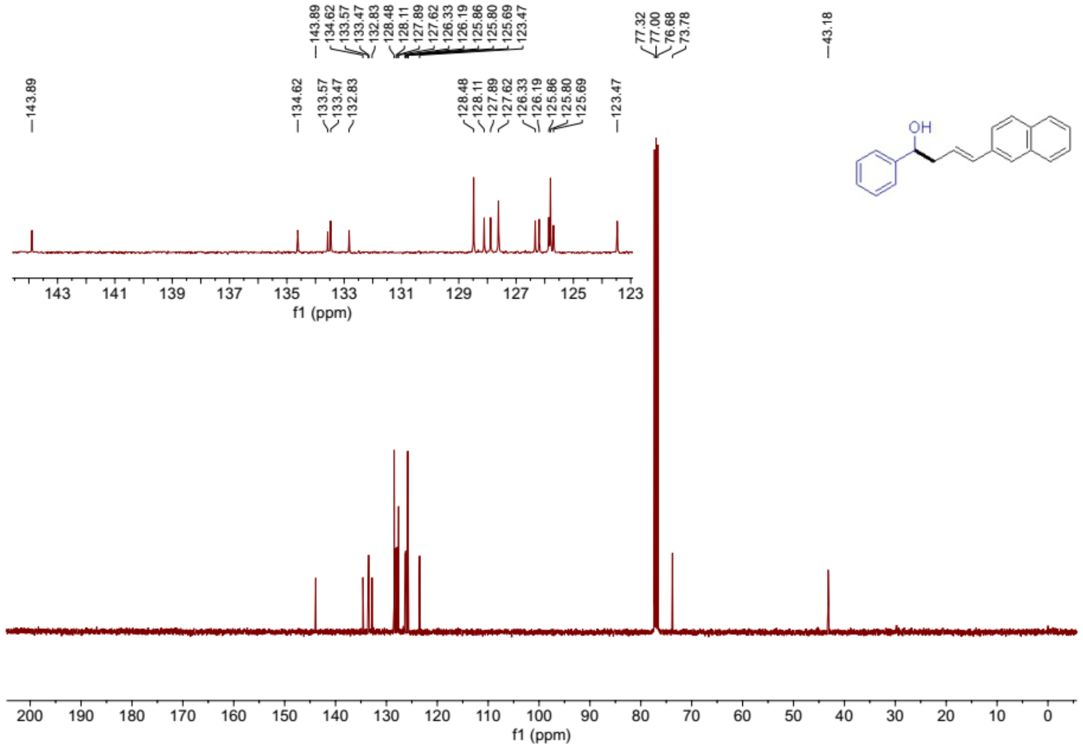

3aj ^{1}H NMR

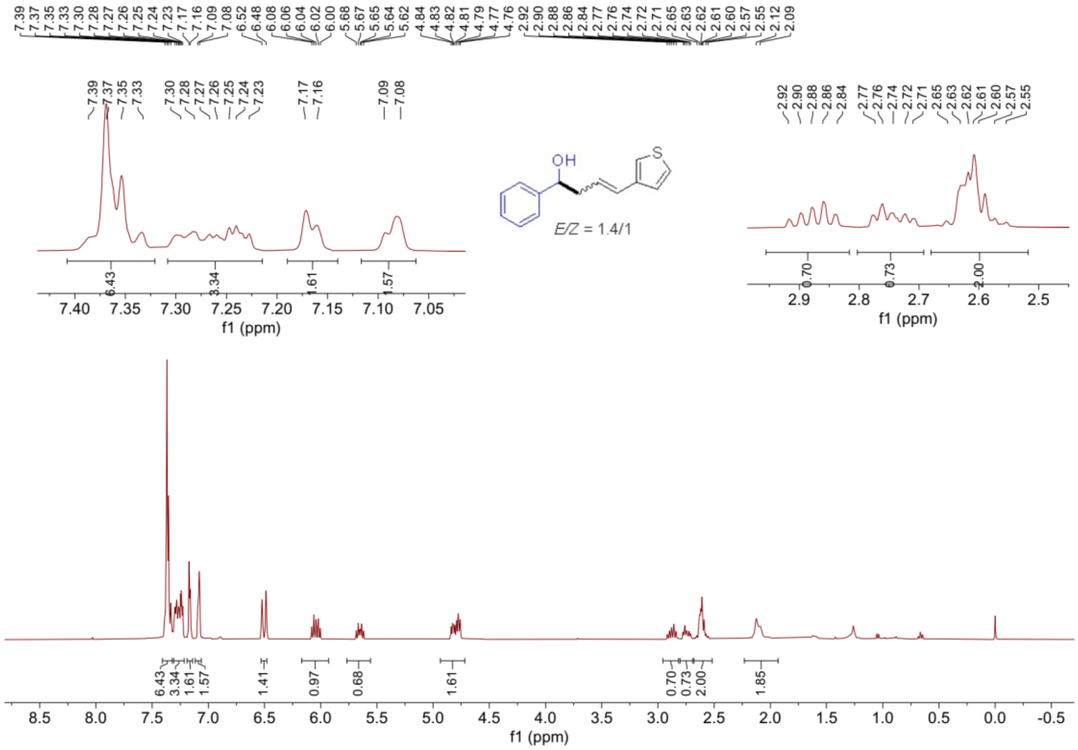

3aj ^{13}C NMR

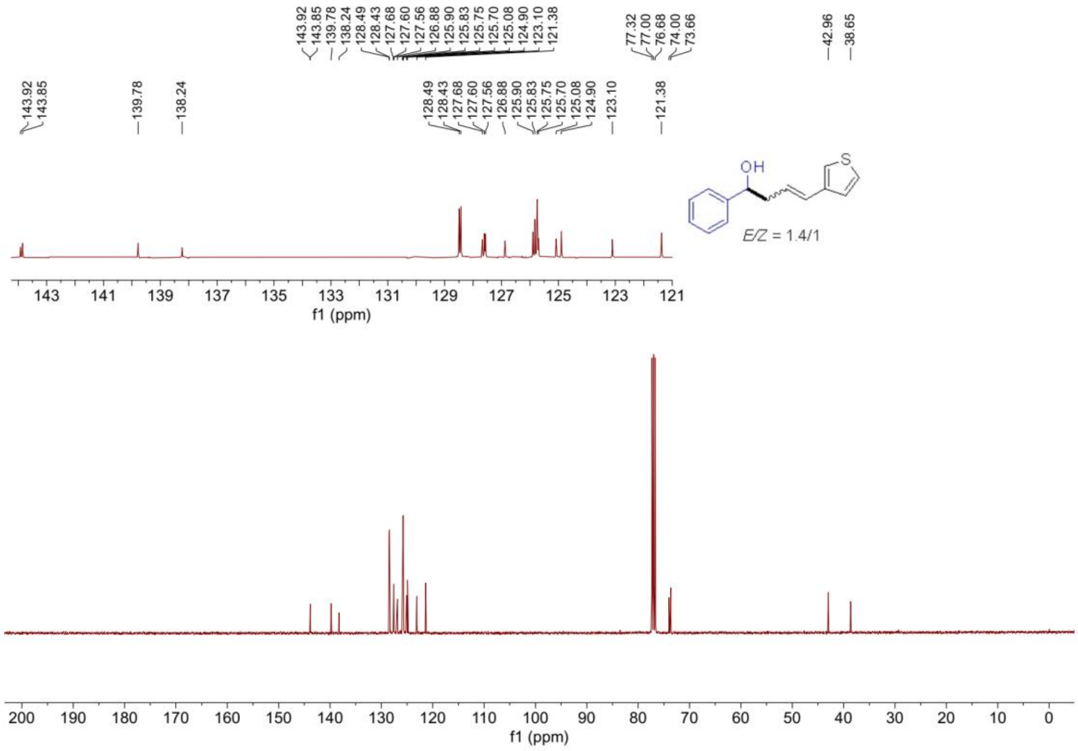

3ak ^{1}H NMR

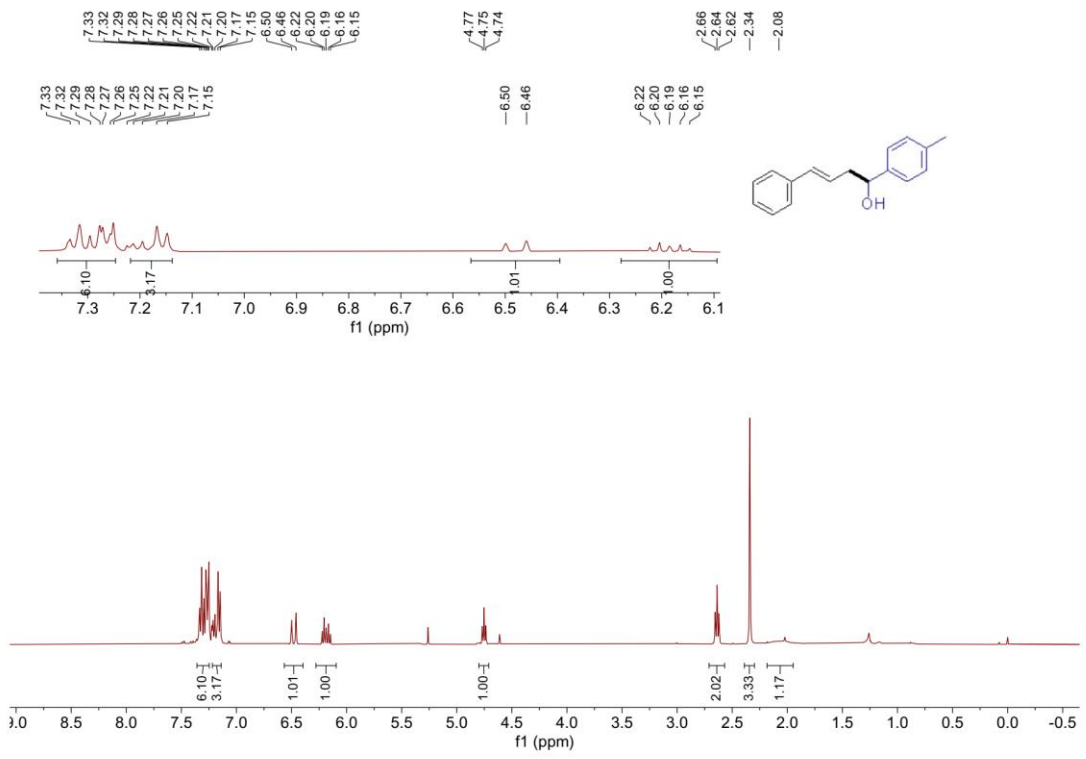

3ak ^{13}C NMR

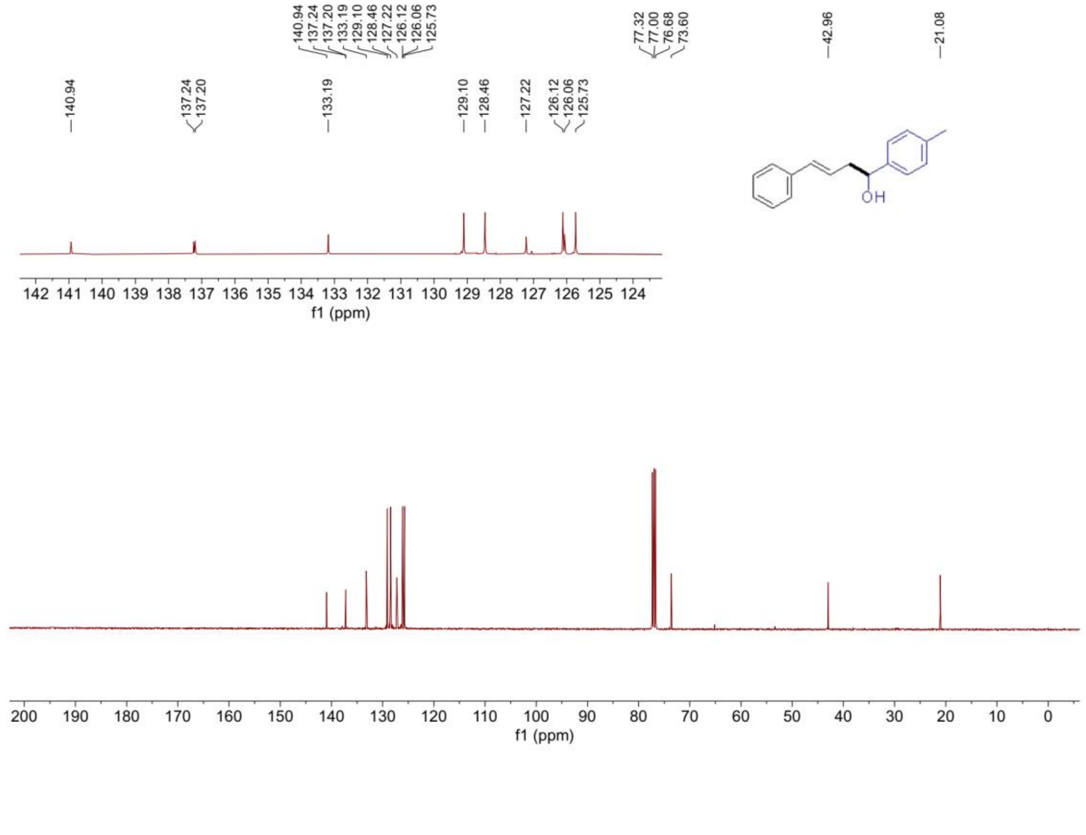

3al ^{1}H NMR

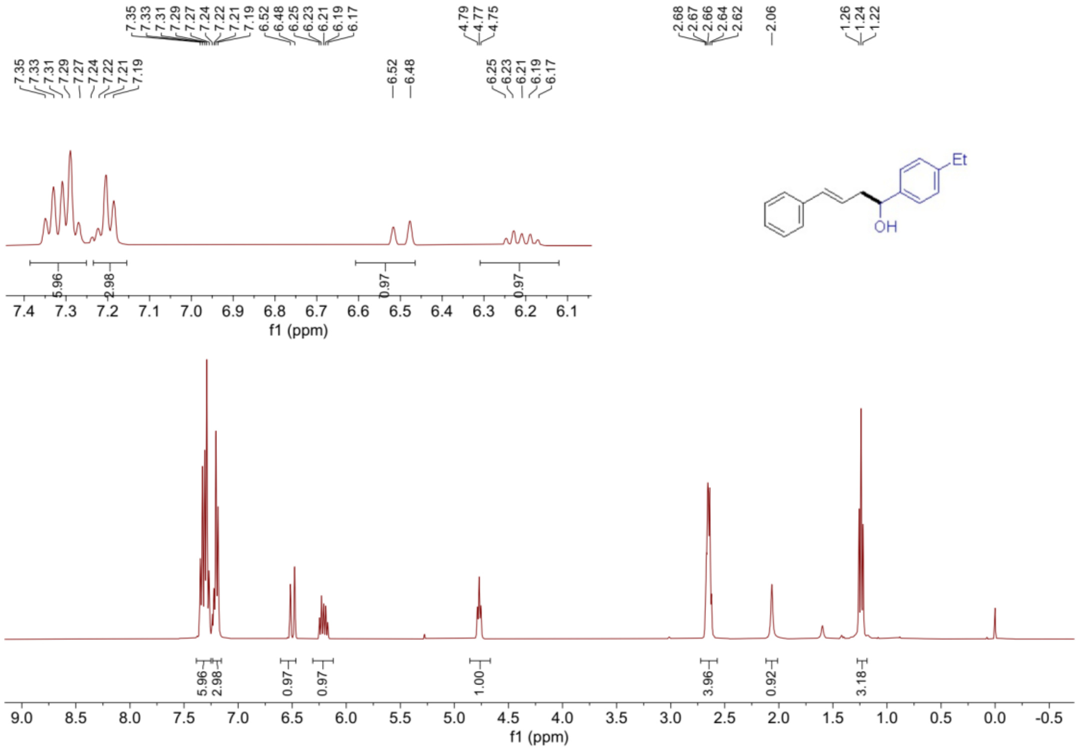

3al ^{13}C NMR

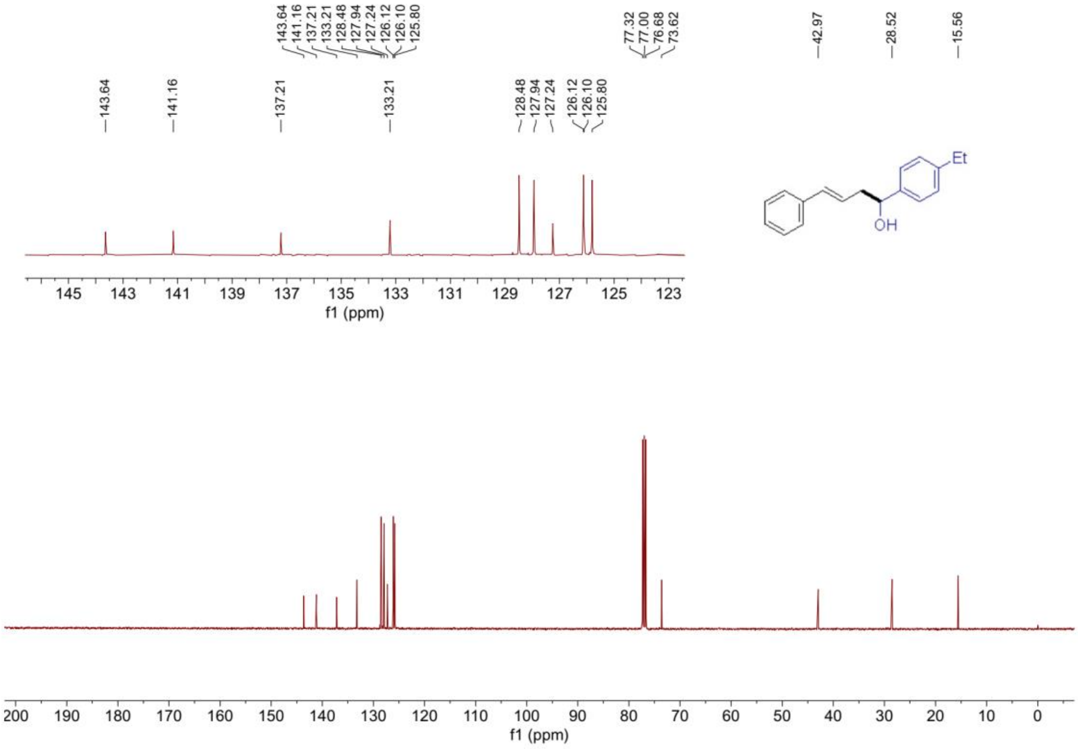

3am ^{1}H NMR

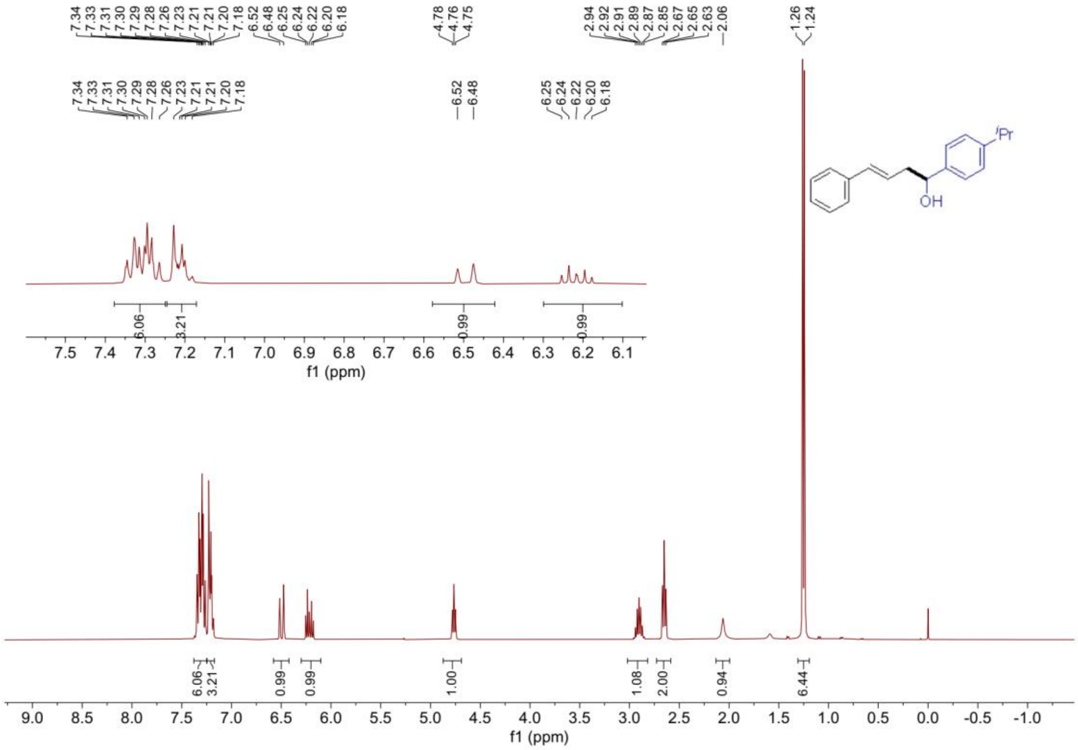

3am ^{13}C NMR

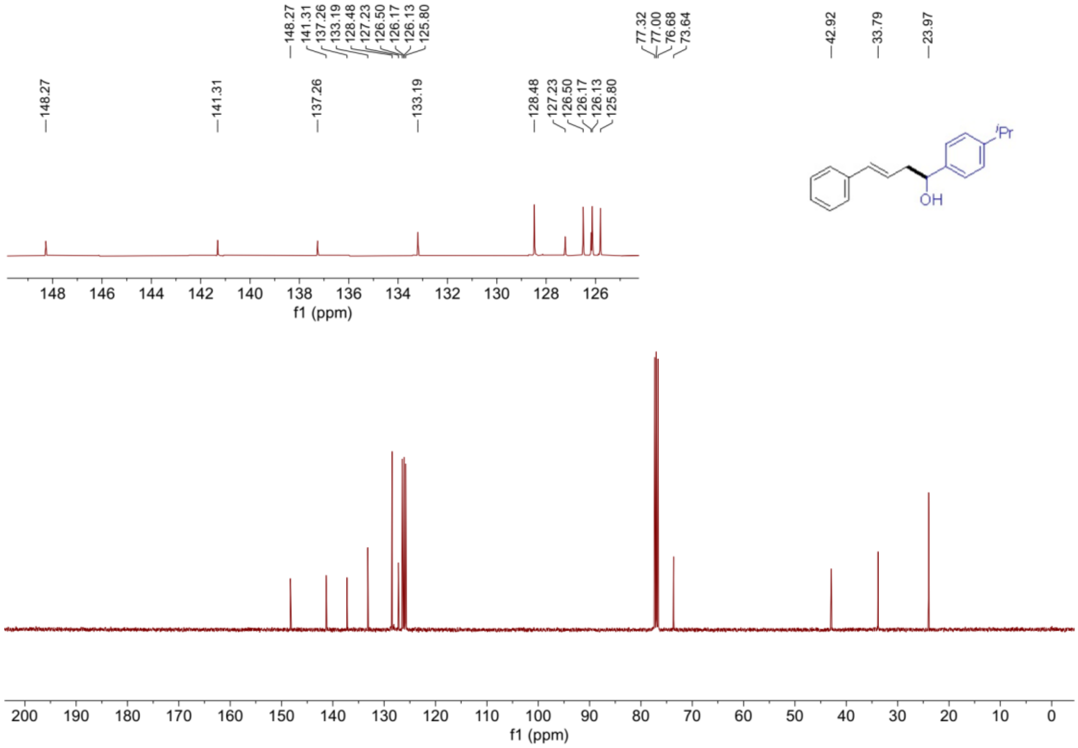

3an ^{1}H NMR

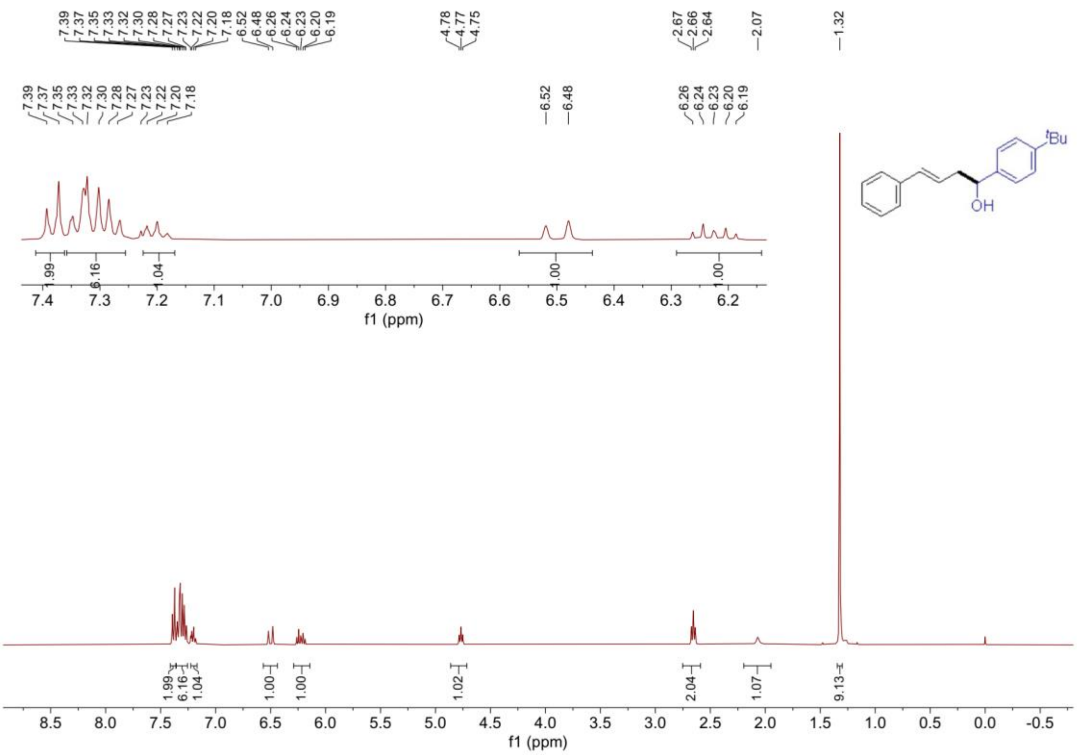

3an ^{13}C NMR

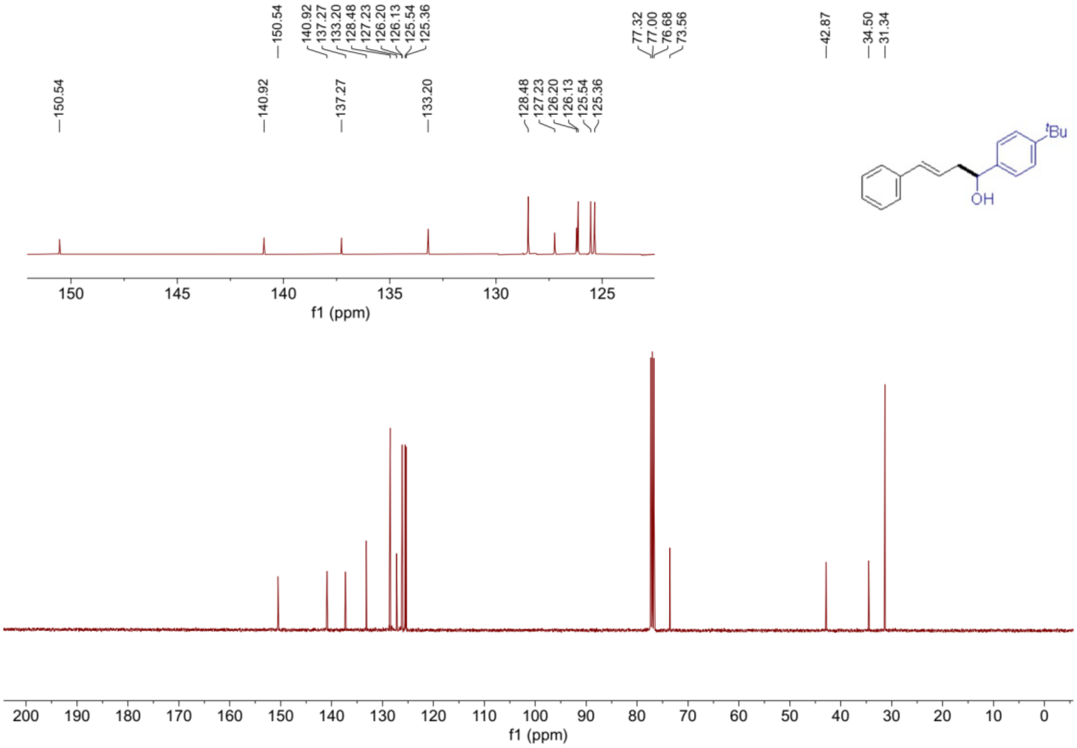

3ao ^{1}H NMR

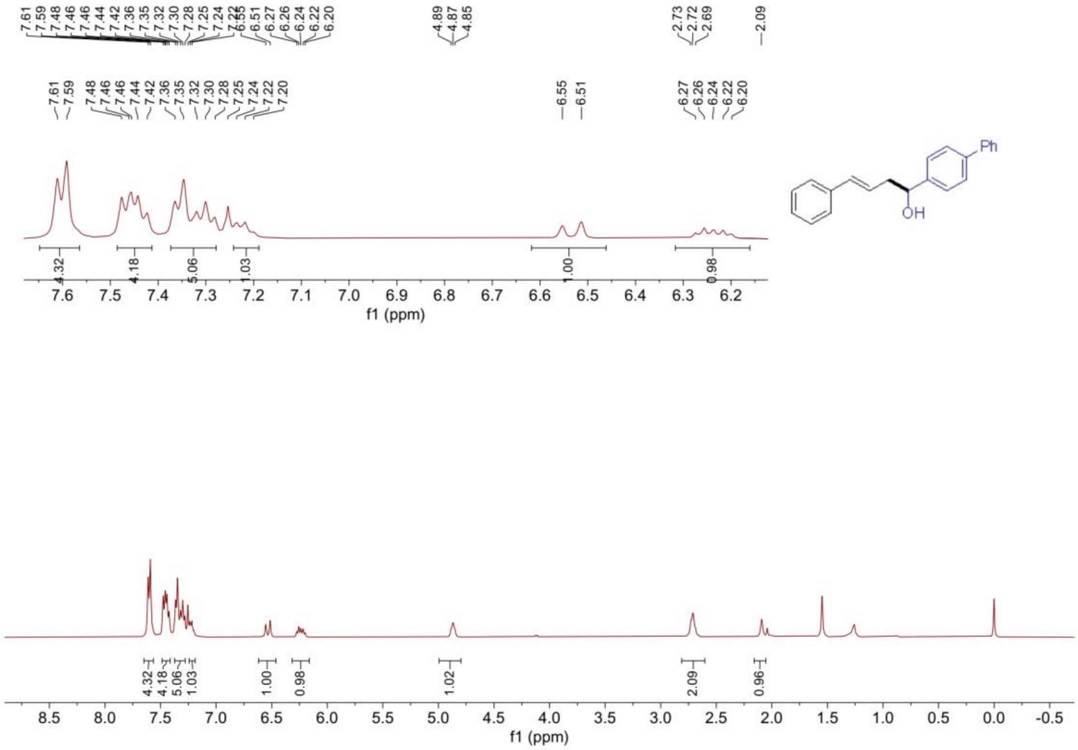

3ao ^{13}C NMR

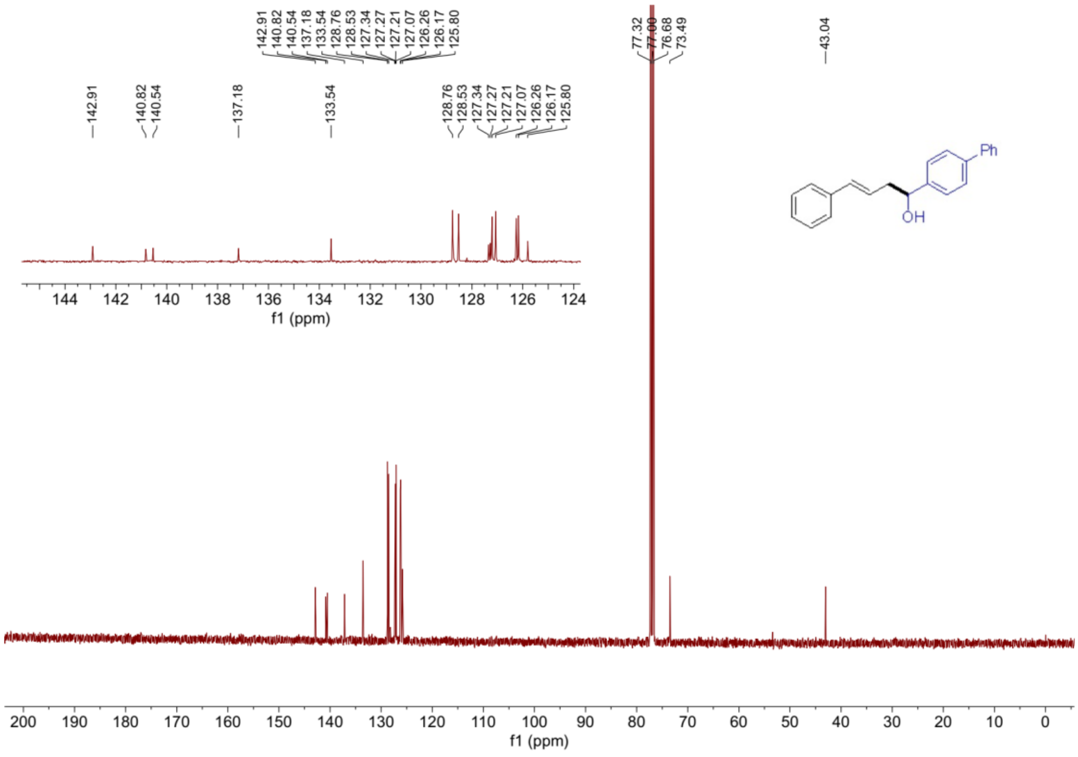

3ap ^{1}H NMR

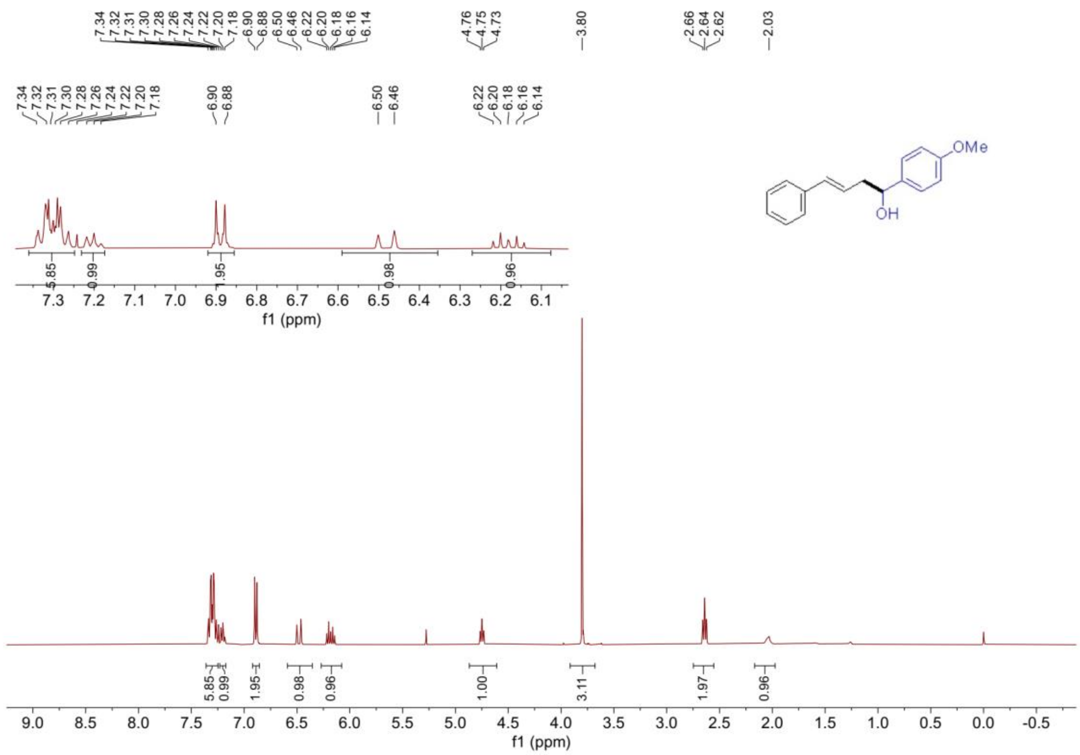

3ap ^{13}C NMR

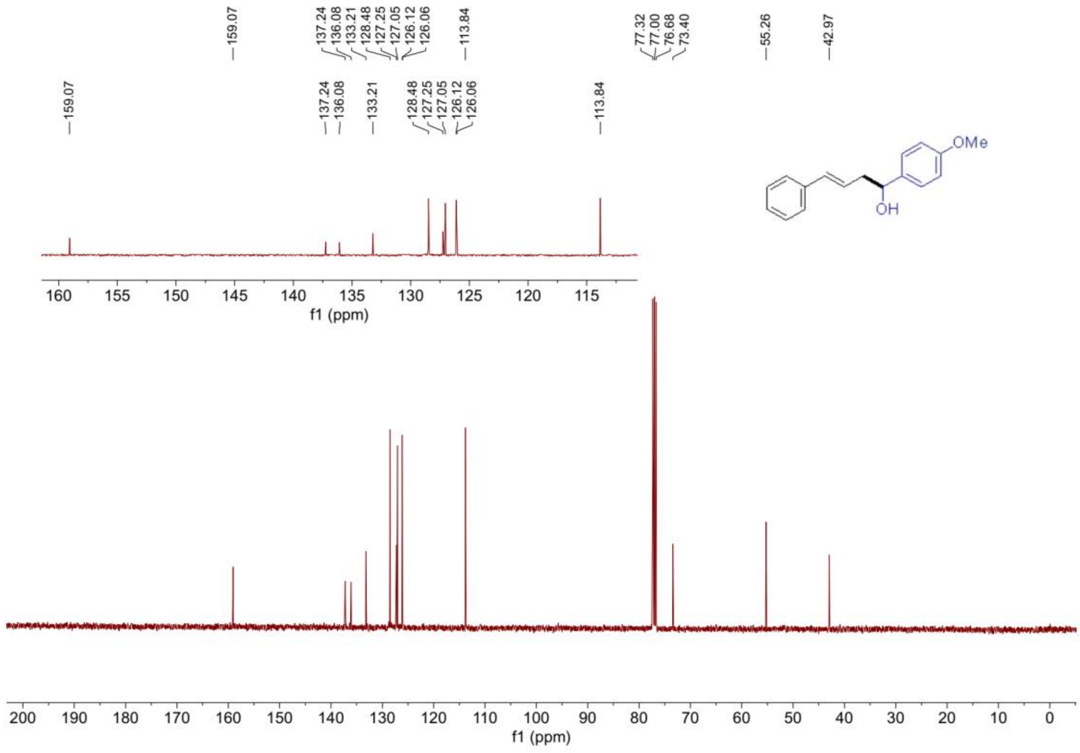

3aq ^{1}H NMR

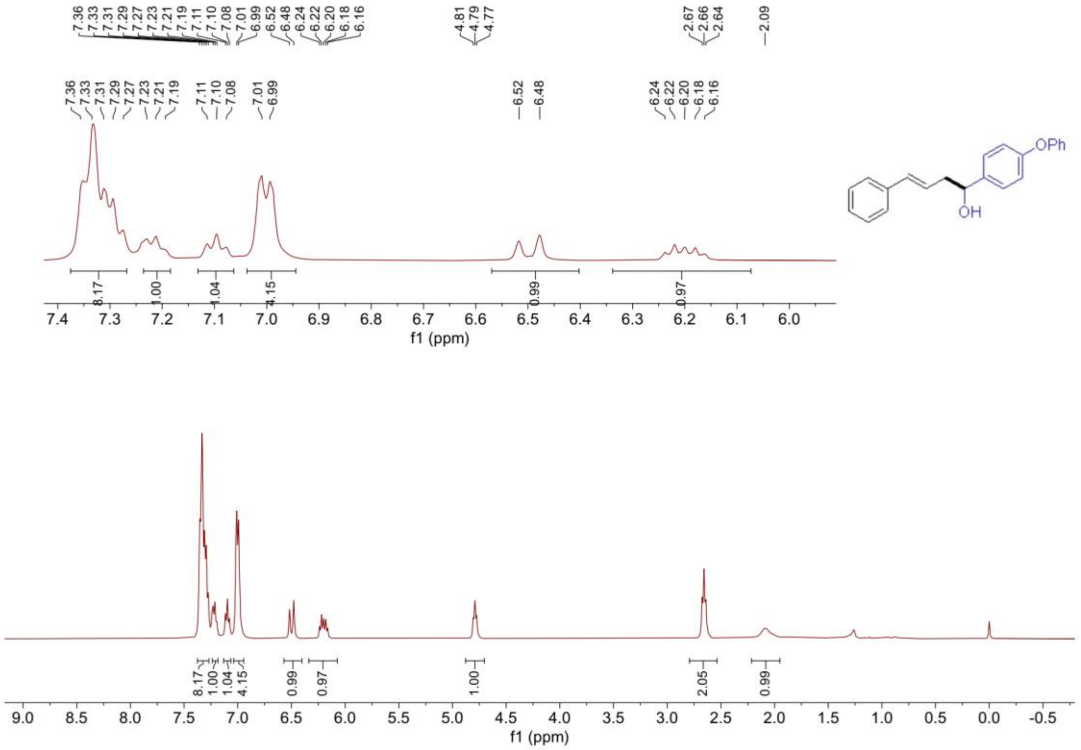

3aq ^{13}C NMR

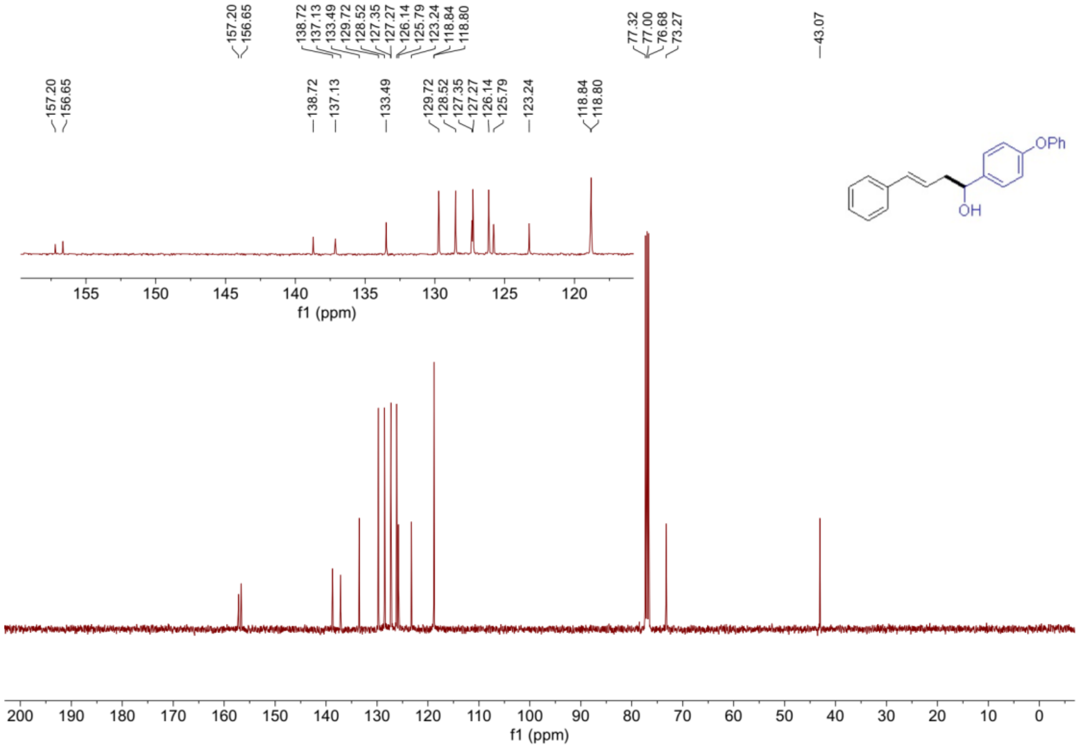

3ar ^{1}H NMR

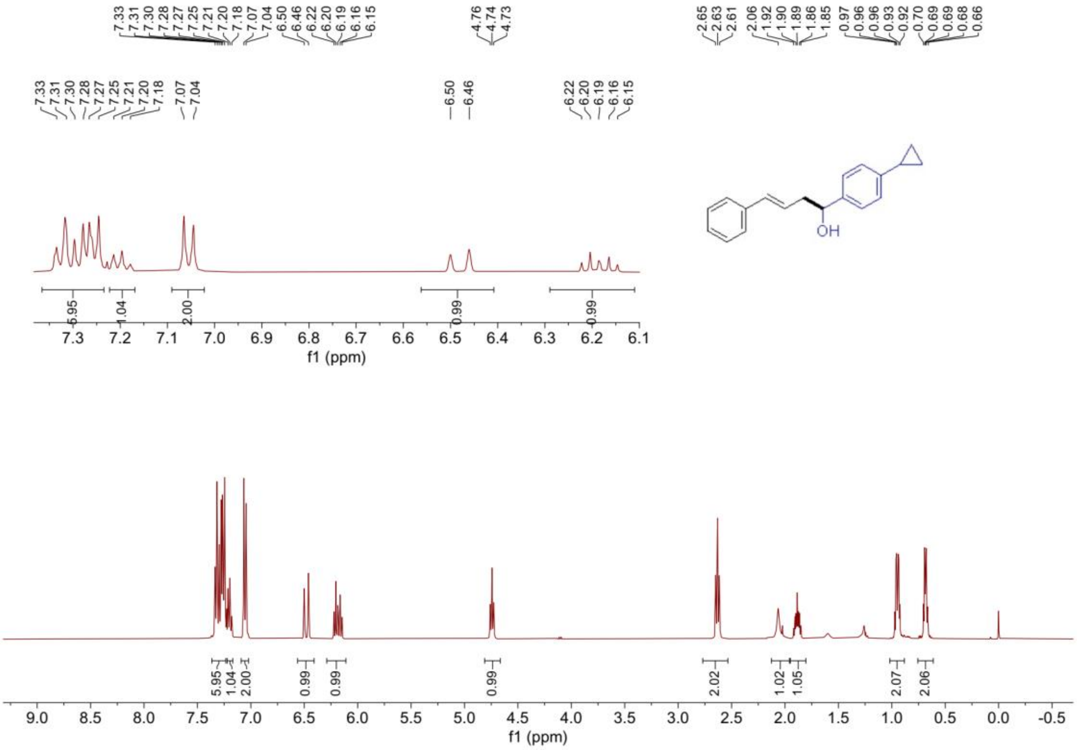

3ar ^{13}C NMR

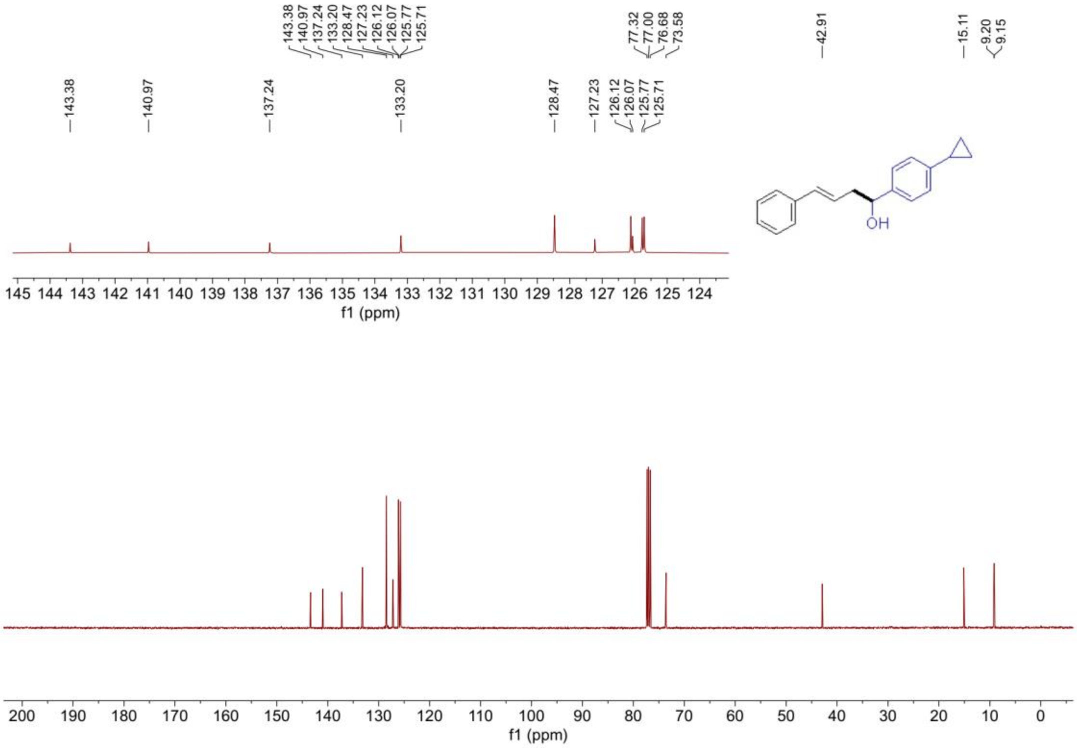

3as ^{1}H NMR

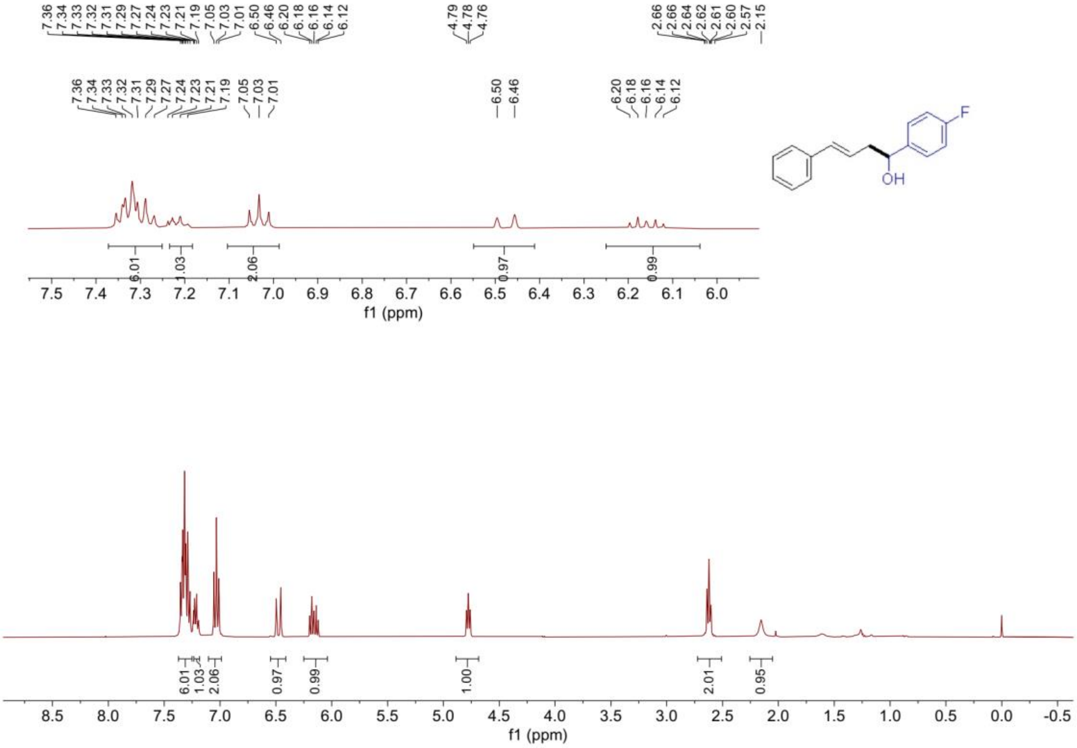

3as ^{13}C NMR

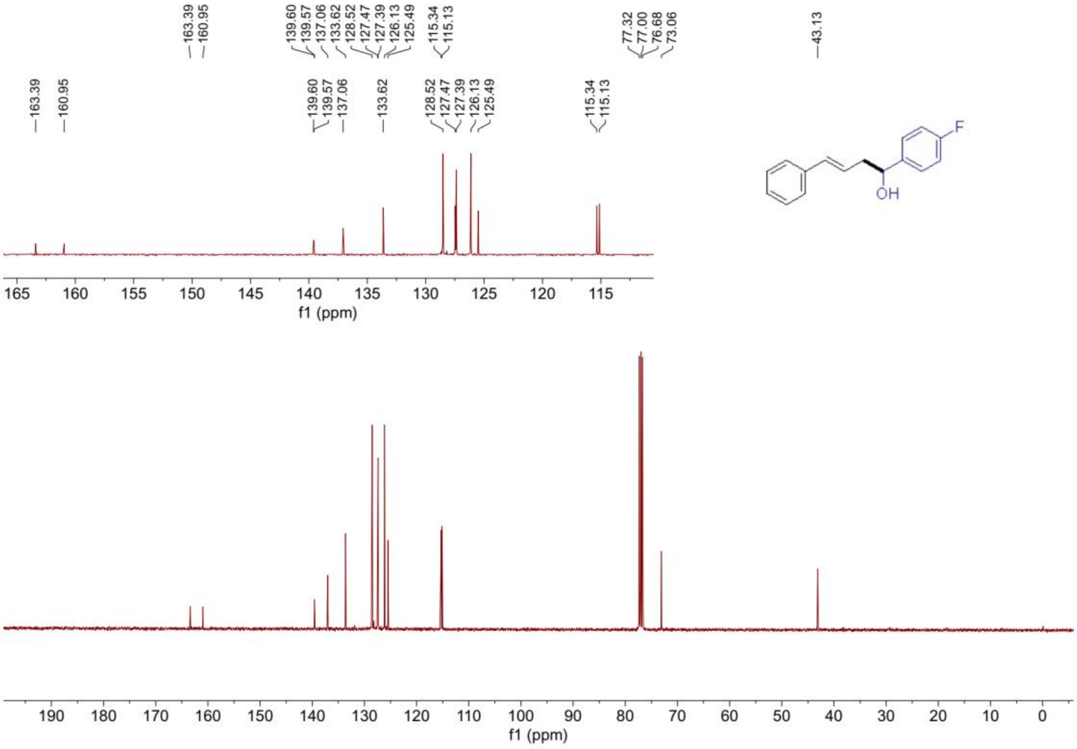

3at ^{1}H NMR

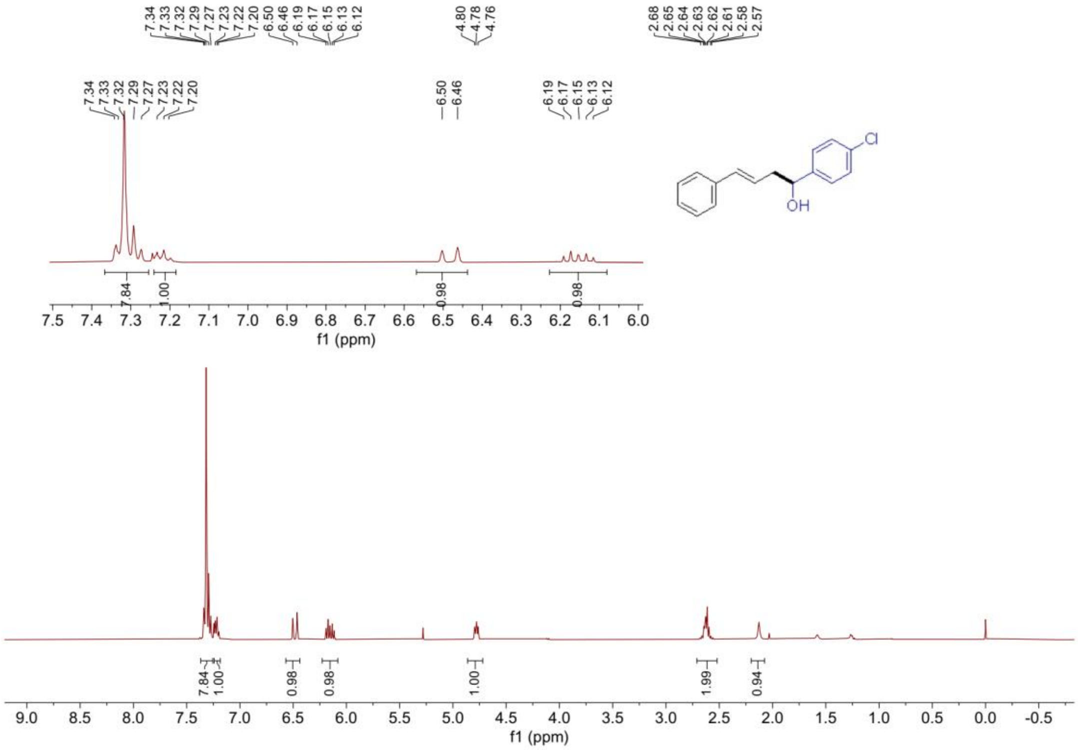

3at ^{13}C NMR

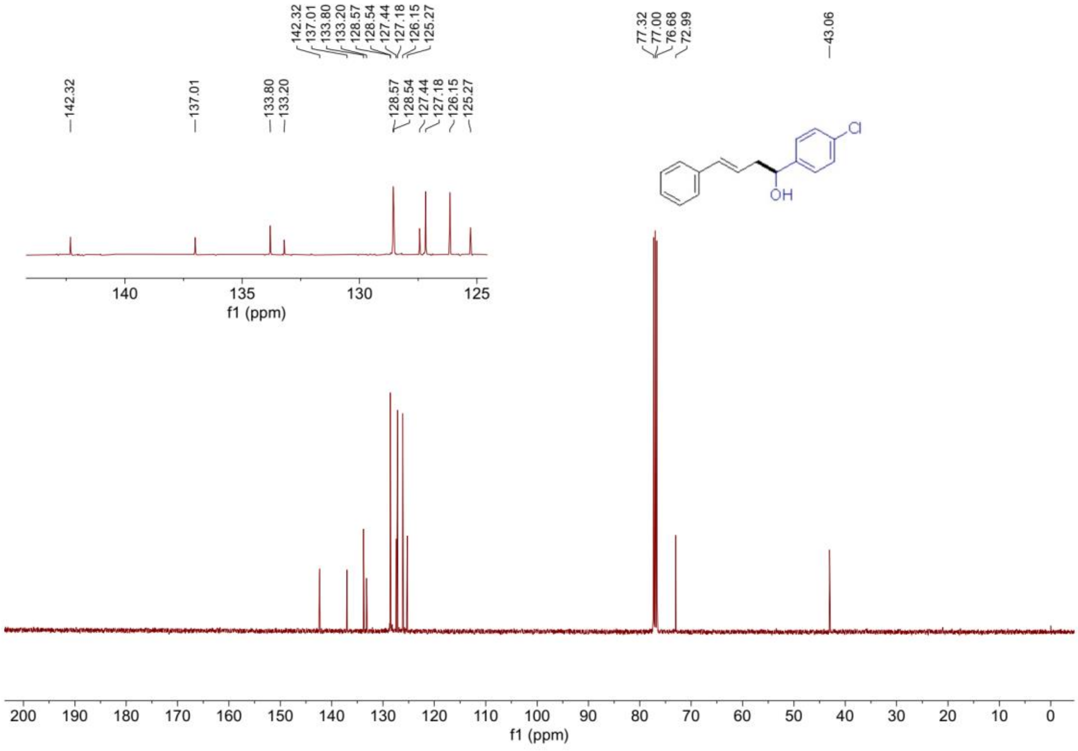

3au ^{1}H NMR

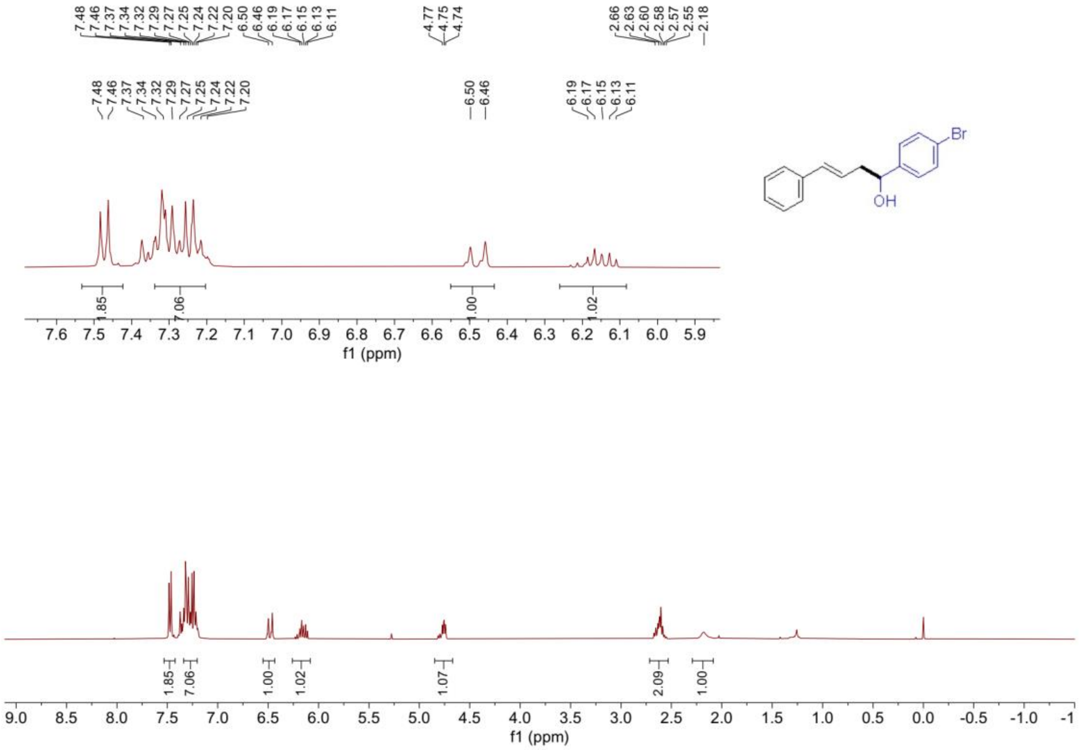

3au ^{13}C NMR

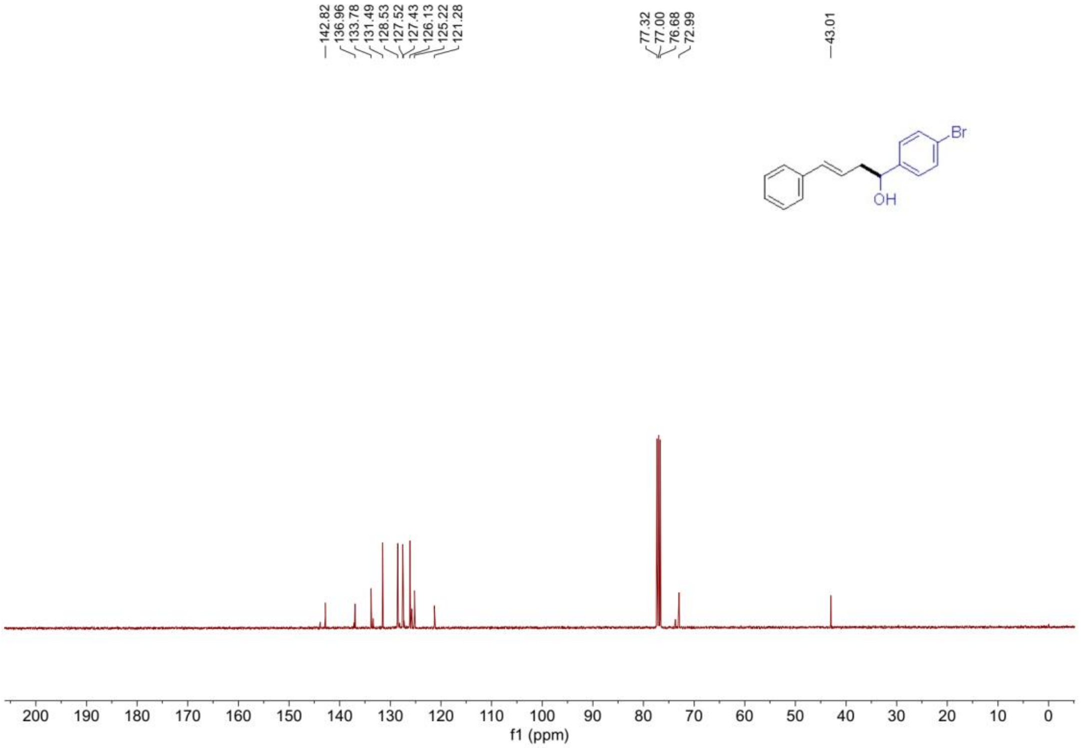

3av ^{1}H NMR

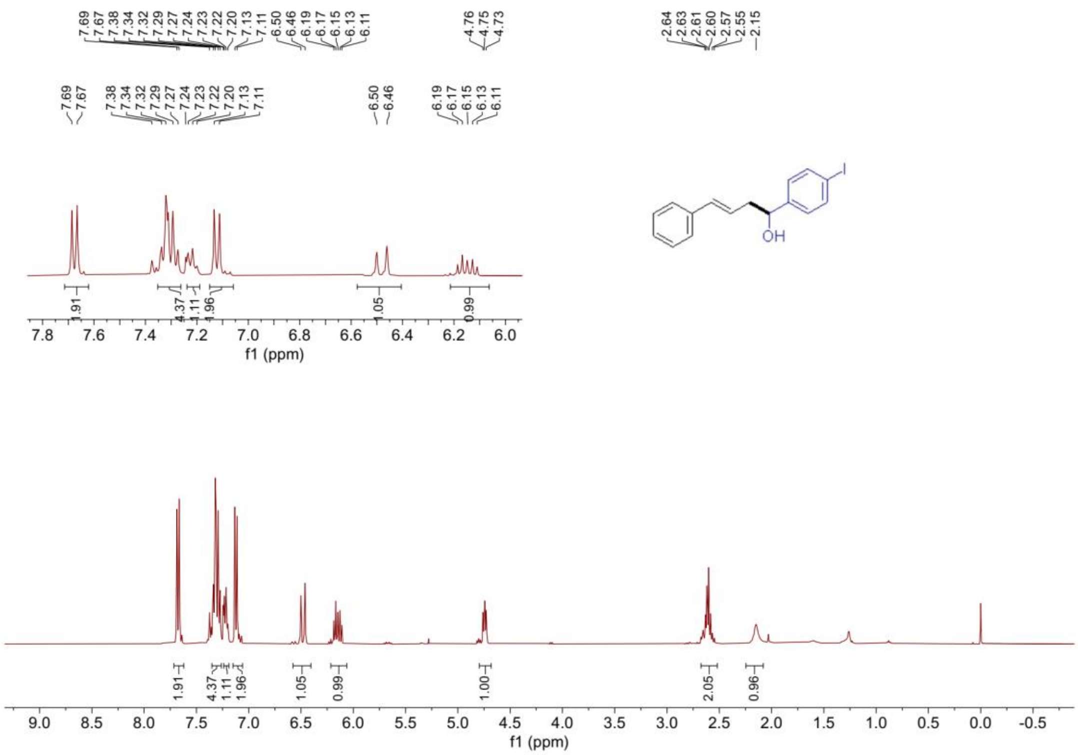

3av ^{13}C NMR

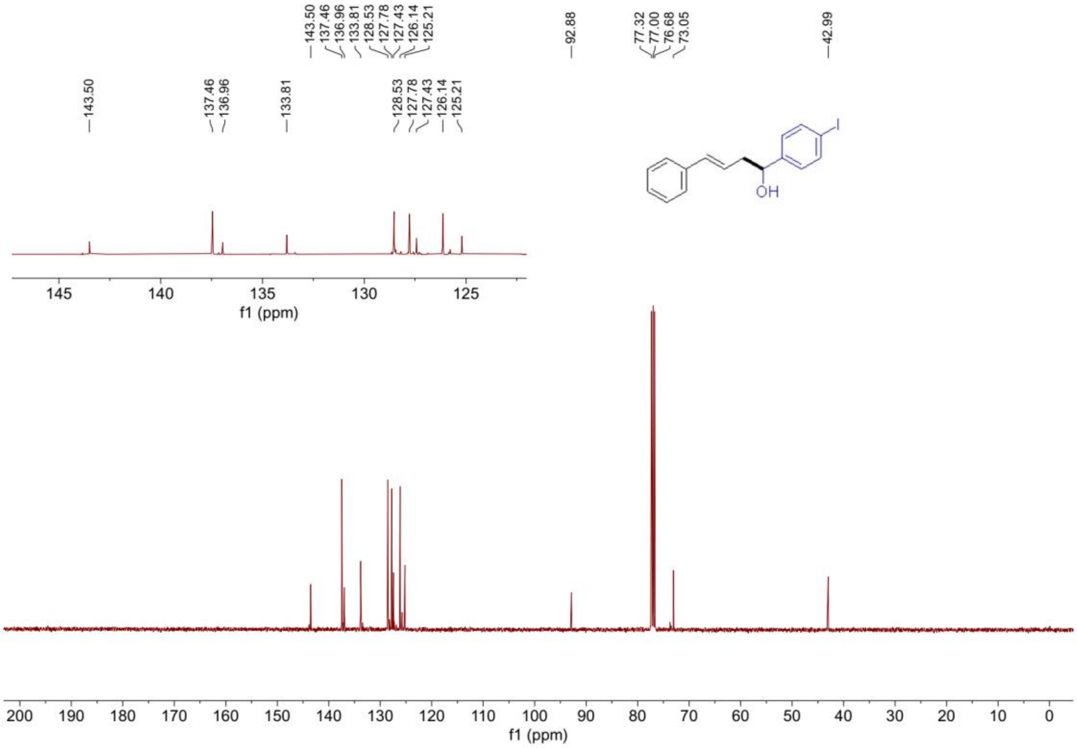

3aw ^{1}H NMR

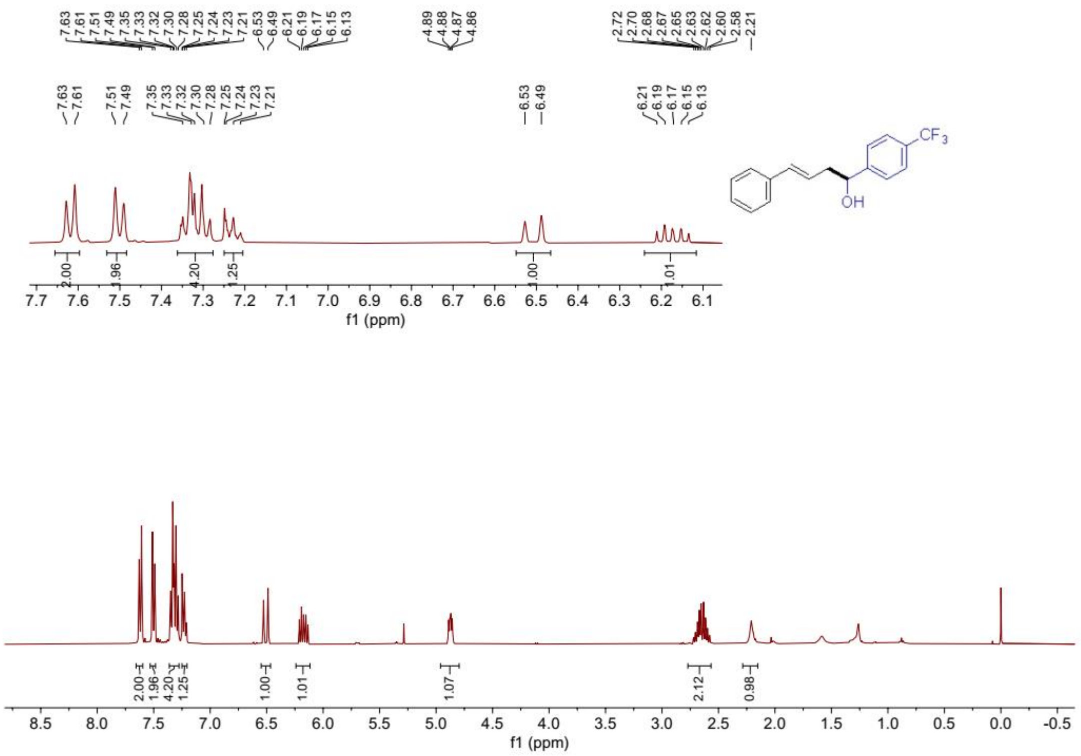

3aw ^{13}C NMR

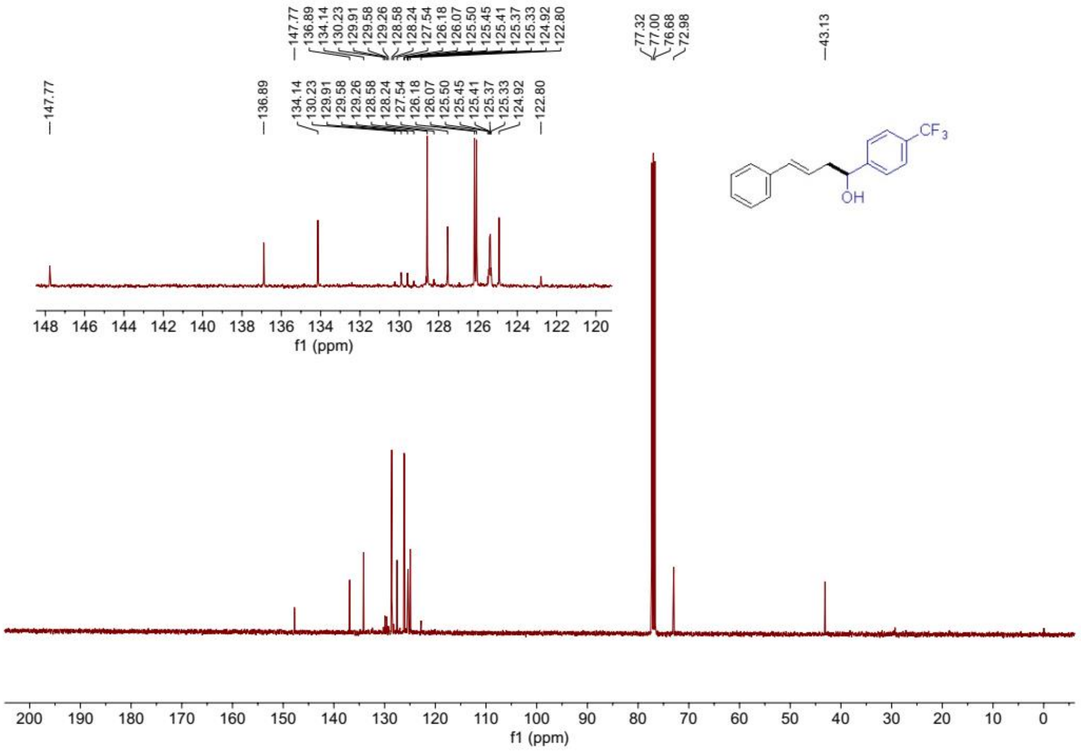

3ax ^{1}H NMR

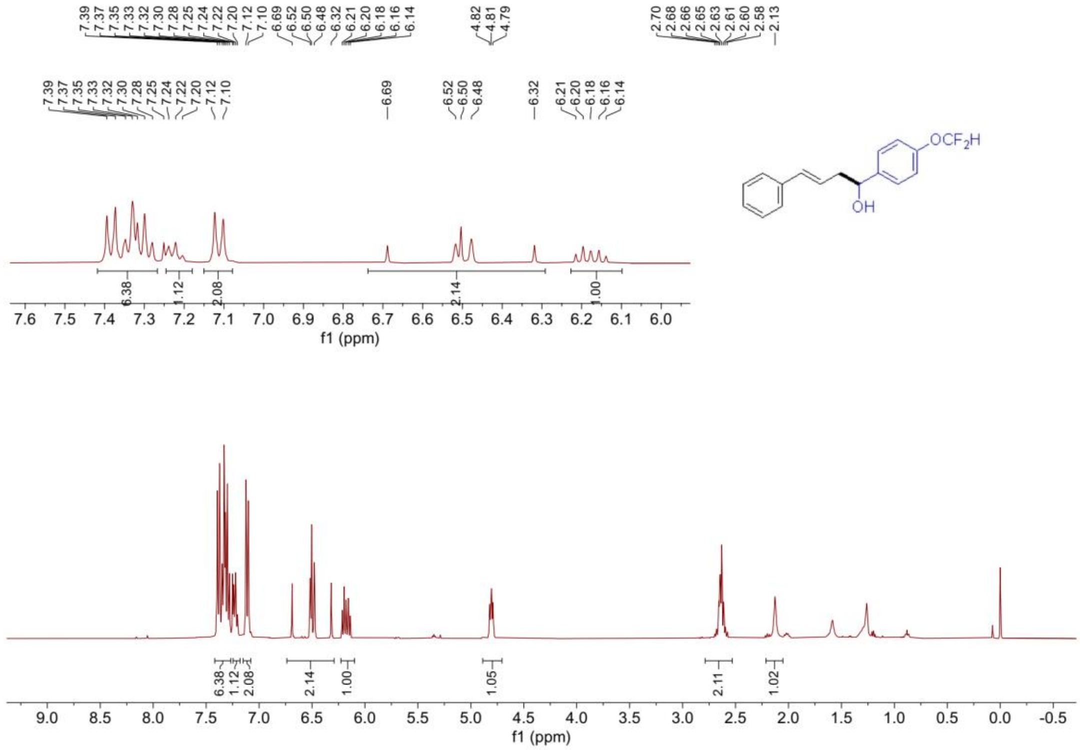

3ax ^{13}C NMR

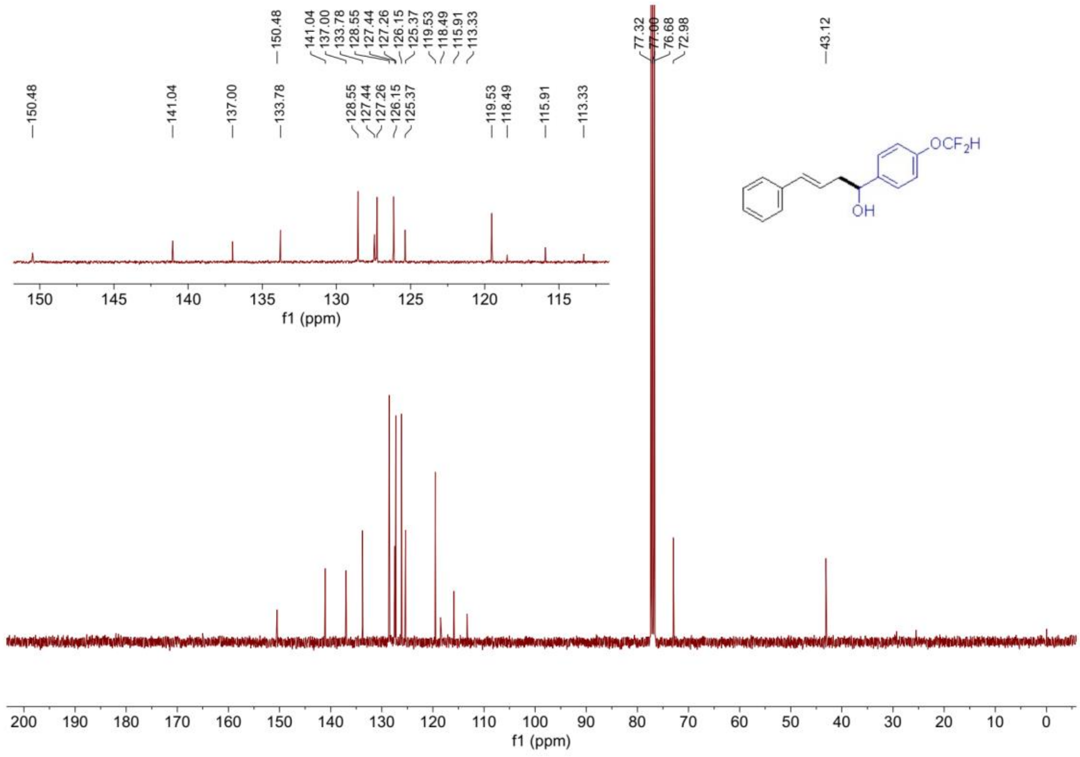

3ay ^{1}H NMR

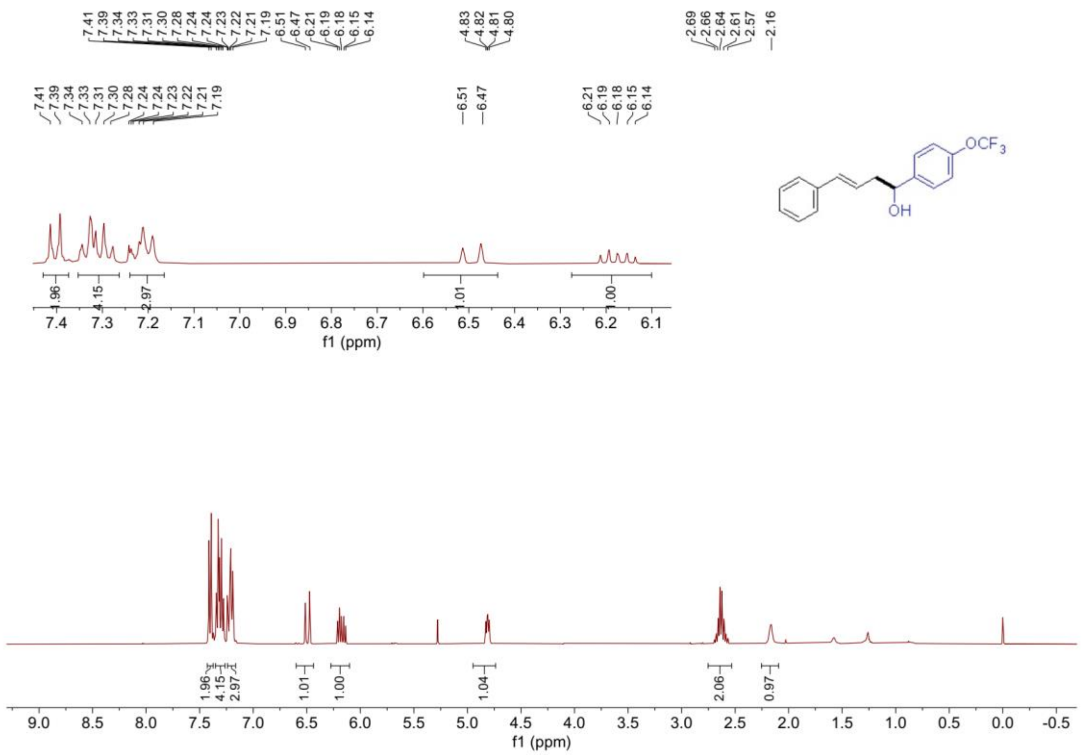

3ay ^{13}C NMR

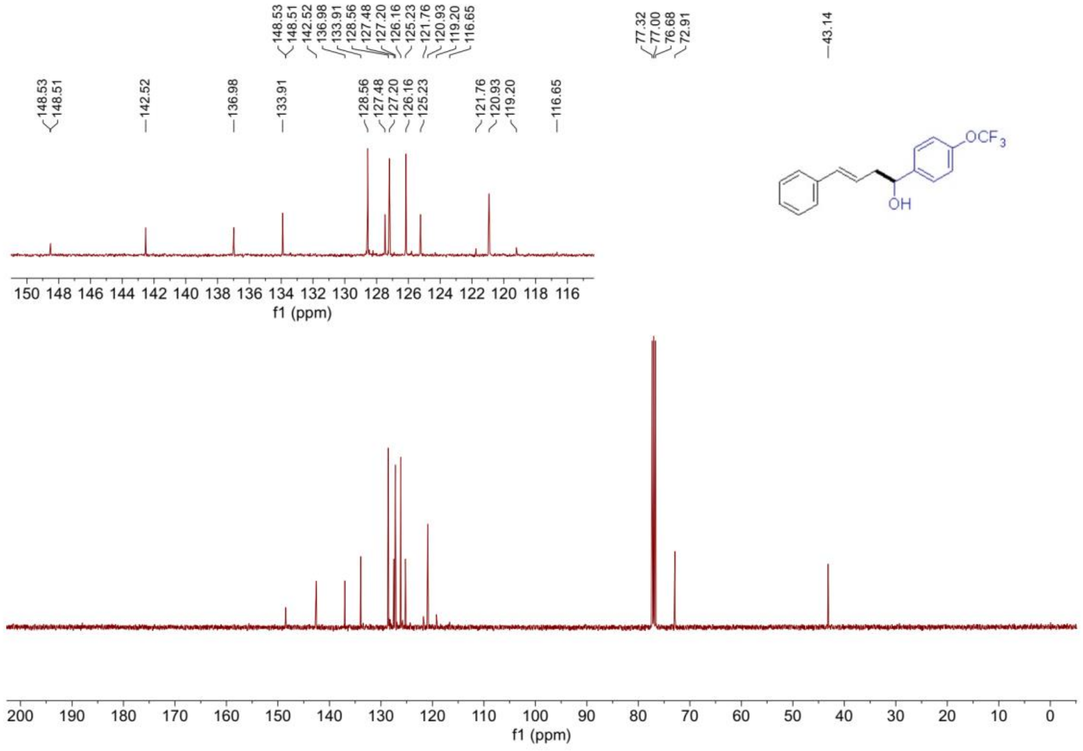

3az ^{1}H NMR

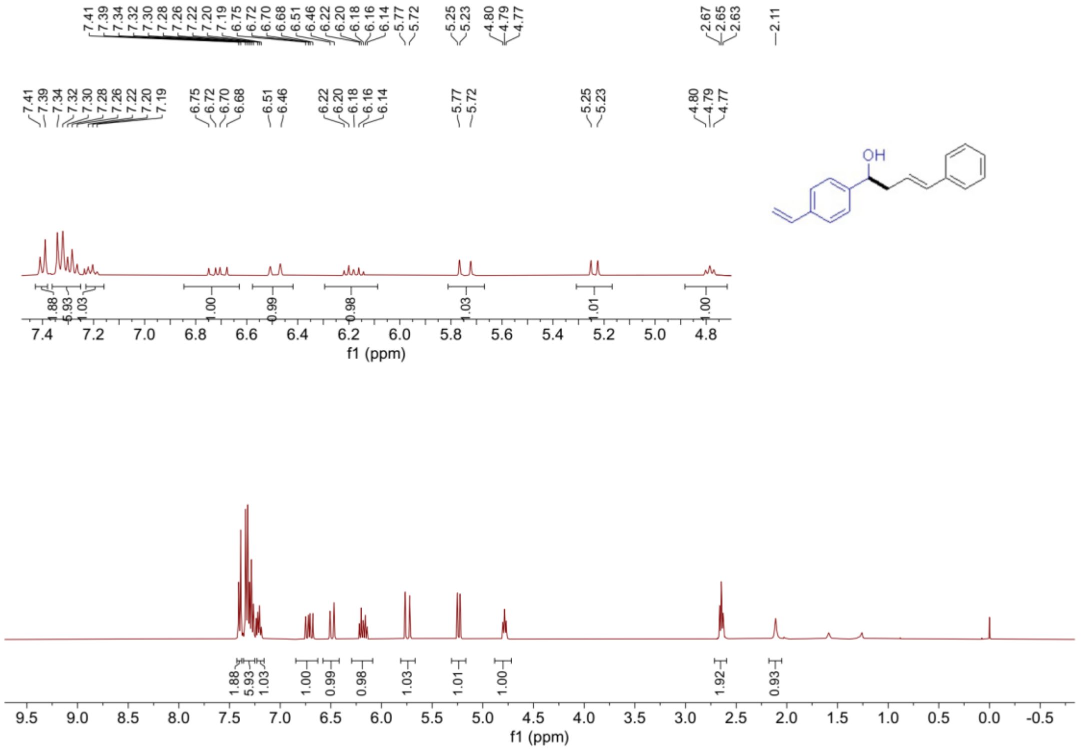

3az ^{13}C NMR

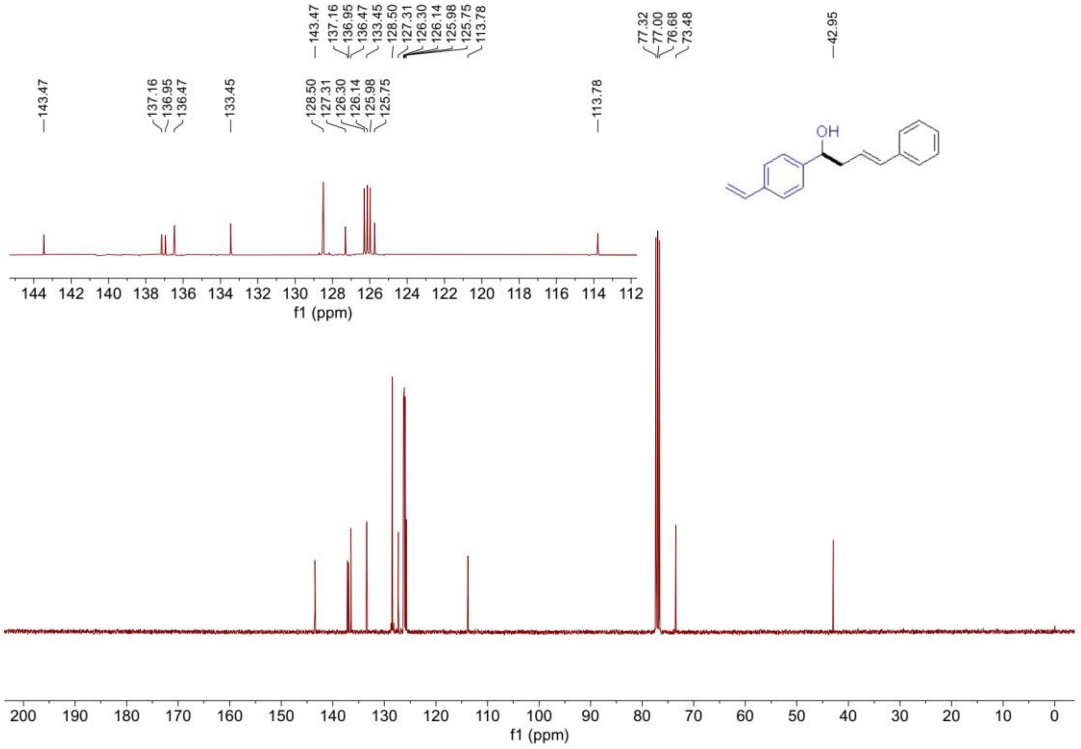

3ba ^{1}H NMR

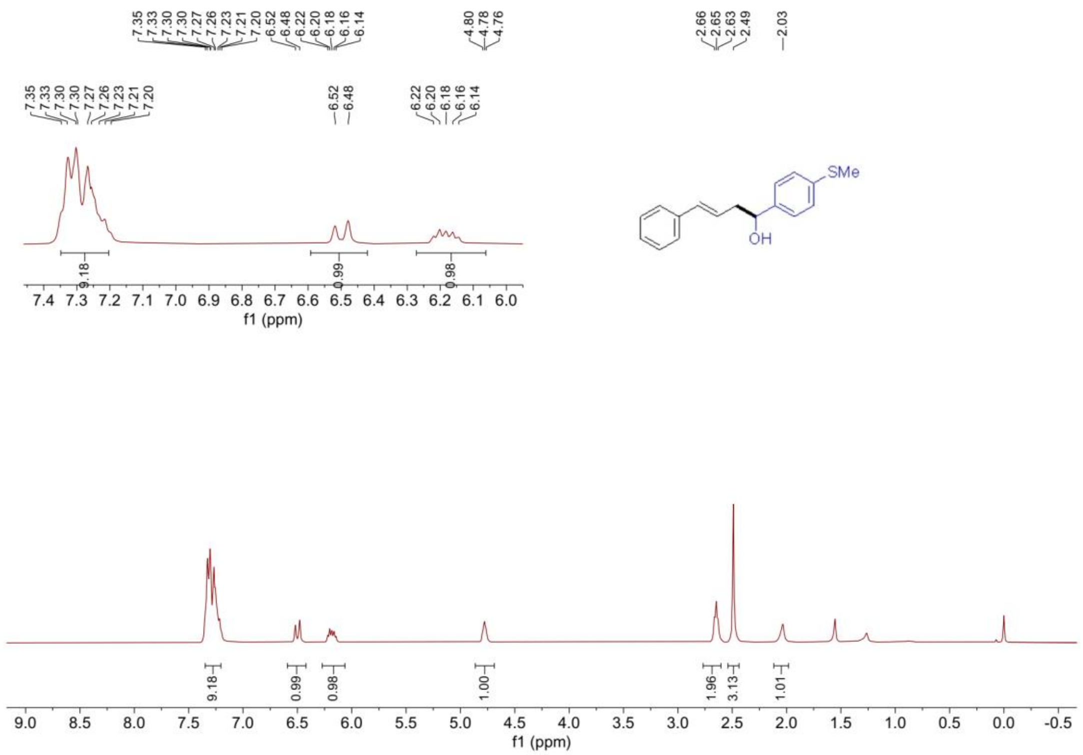

3ba ^{13}C NMR

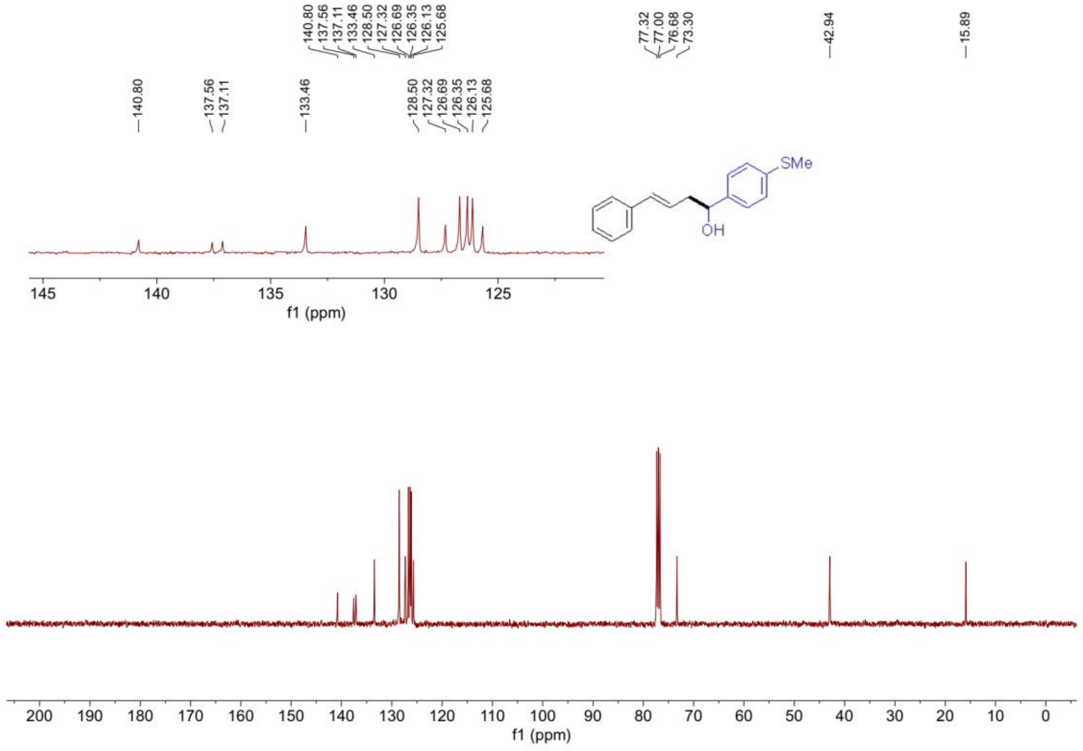

3bb ^{1}H NMR

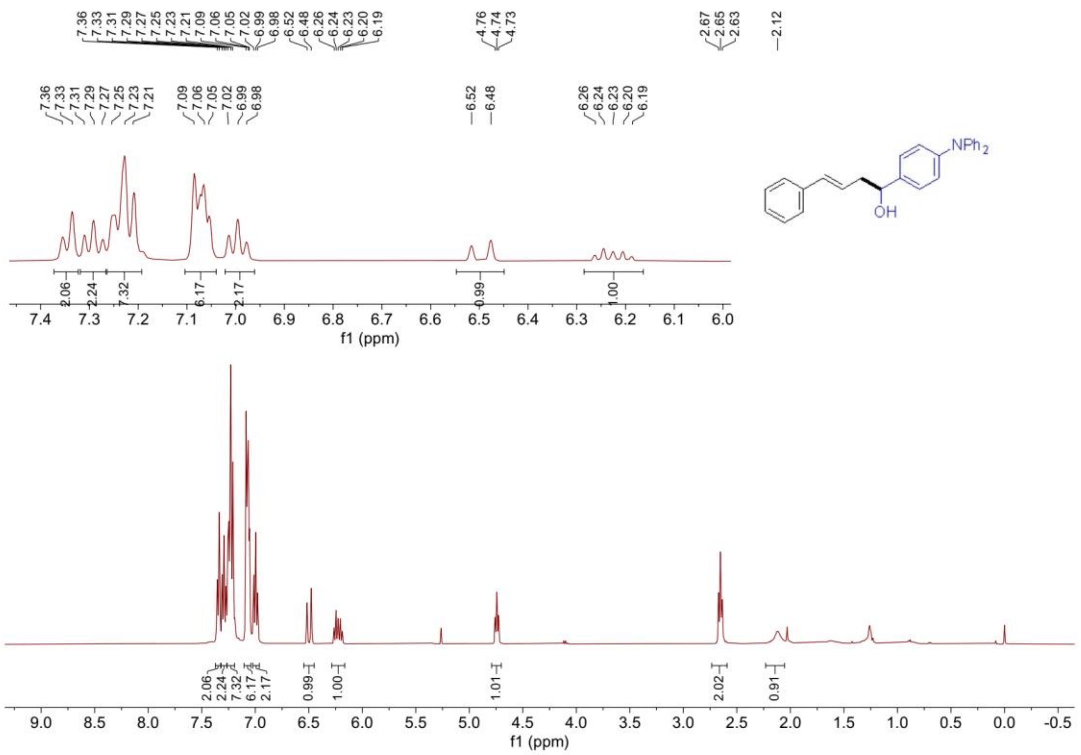

3bb ^{13}C NMR

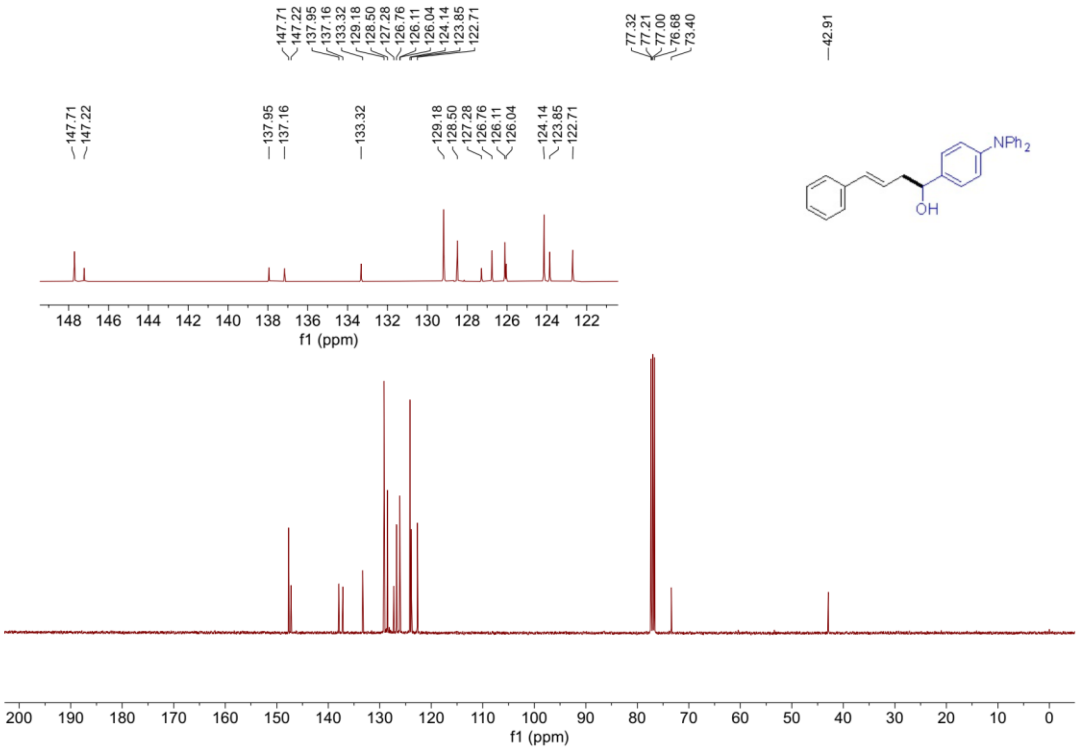

3bc ^{1}H NMR

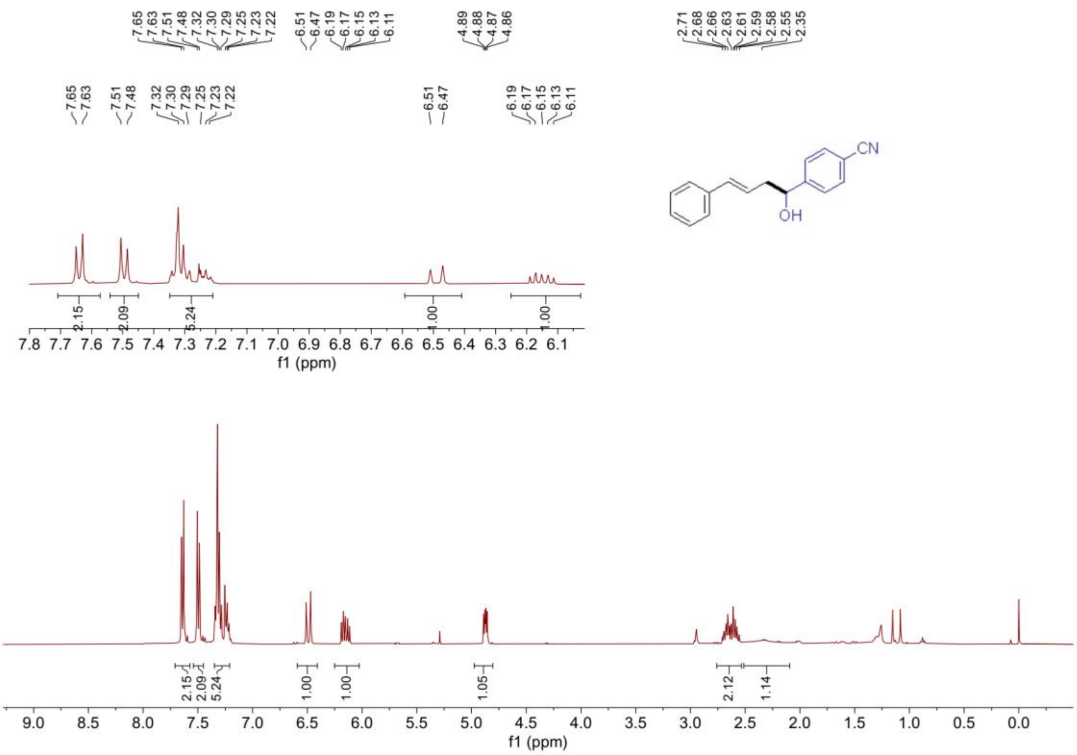

3bc ^{13}C NMR

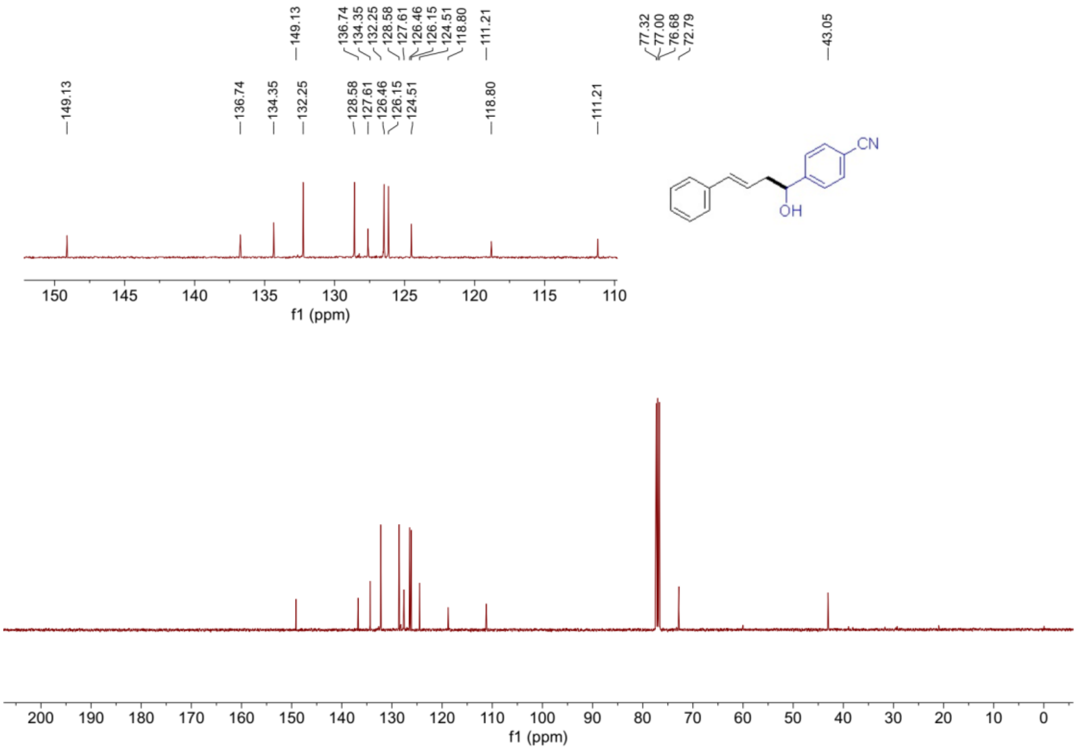

3bd ^{1}H NMR

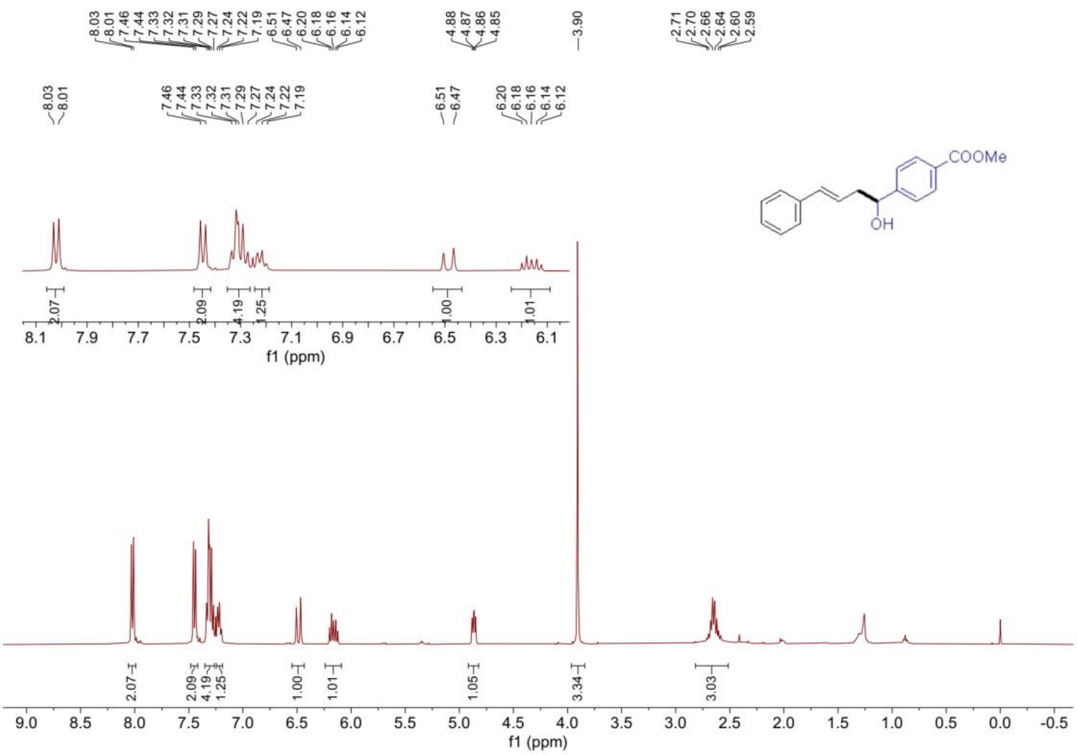

3bd ^{13}C NMR

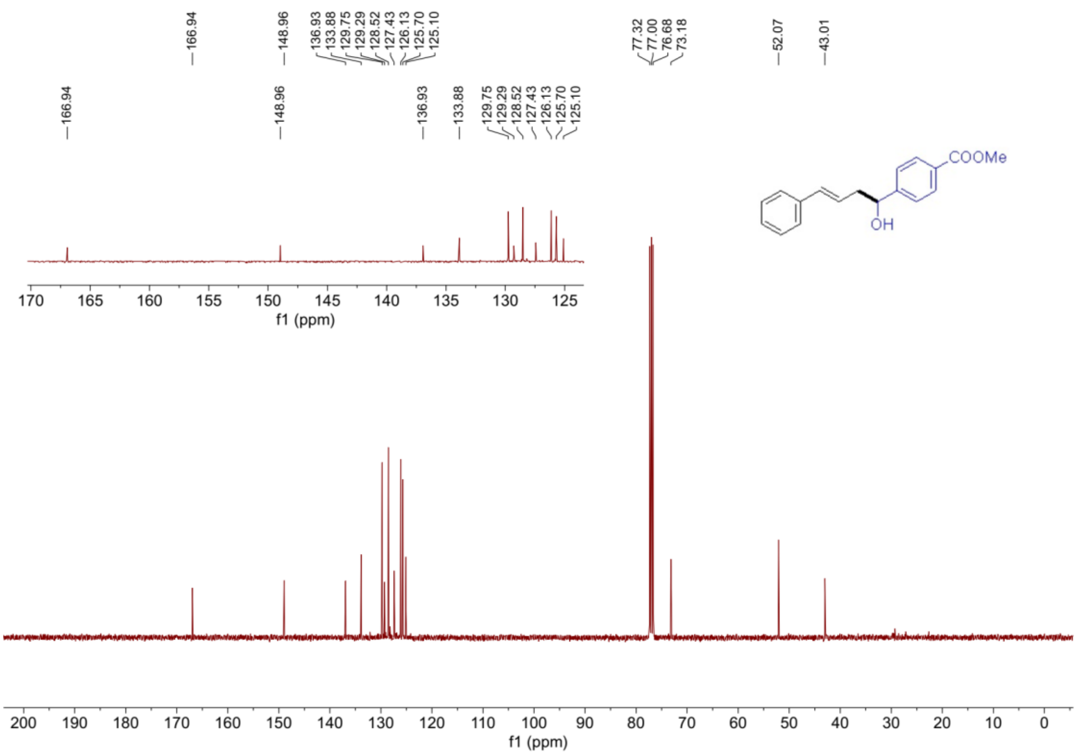

3be ^{1}H NMR

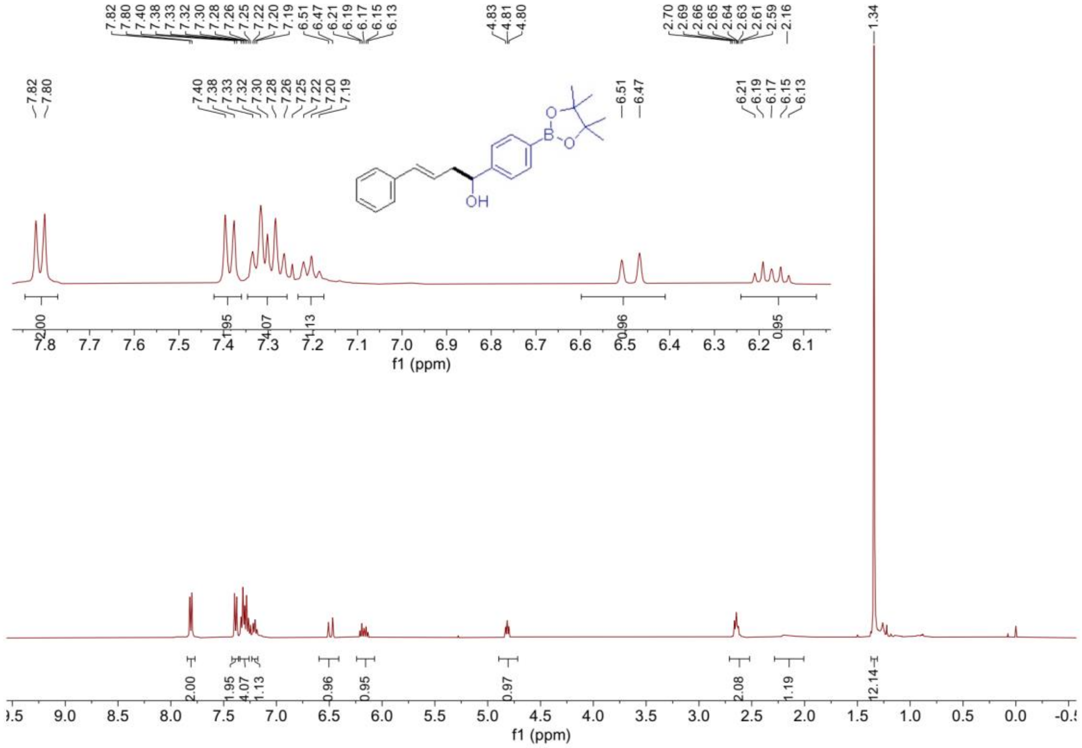

3be ^{13}C NMR

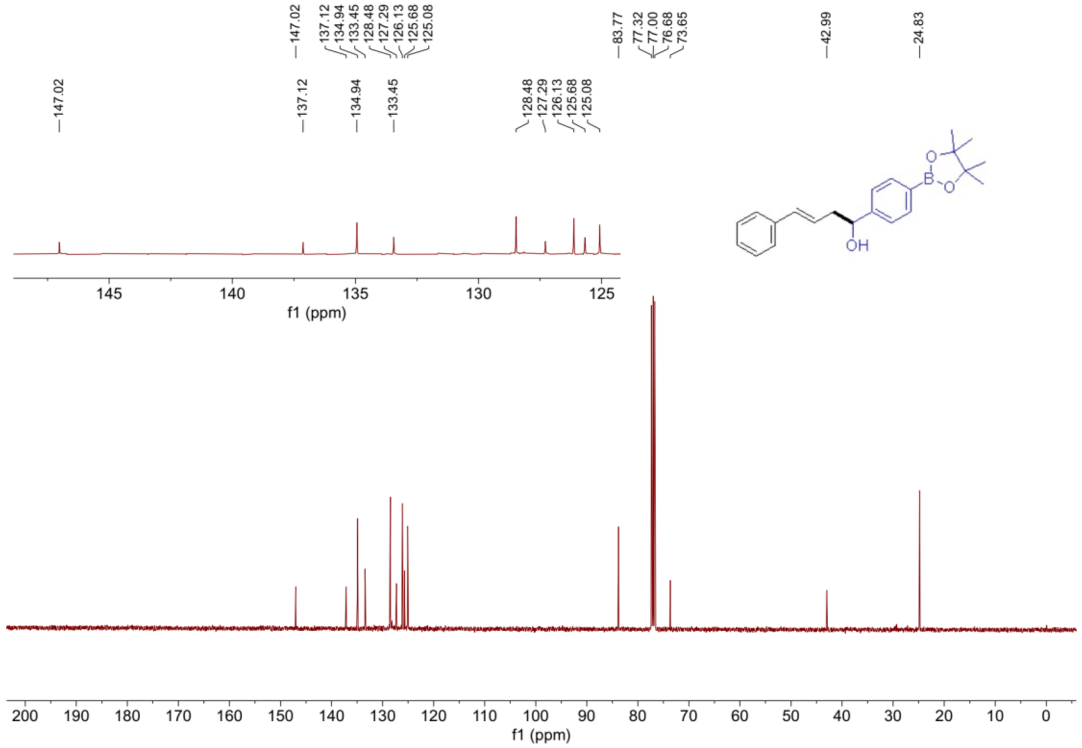

3bf ^{1}H NMR

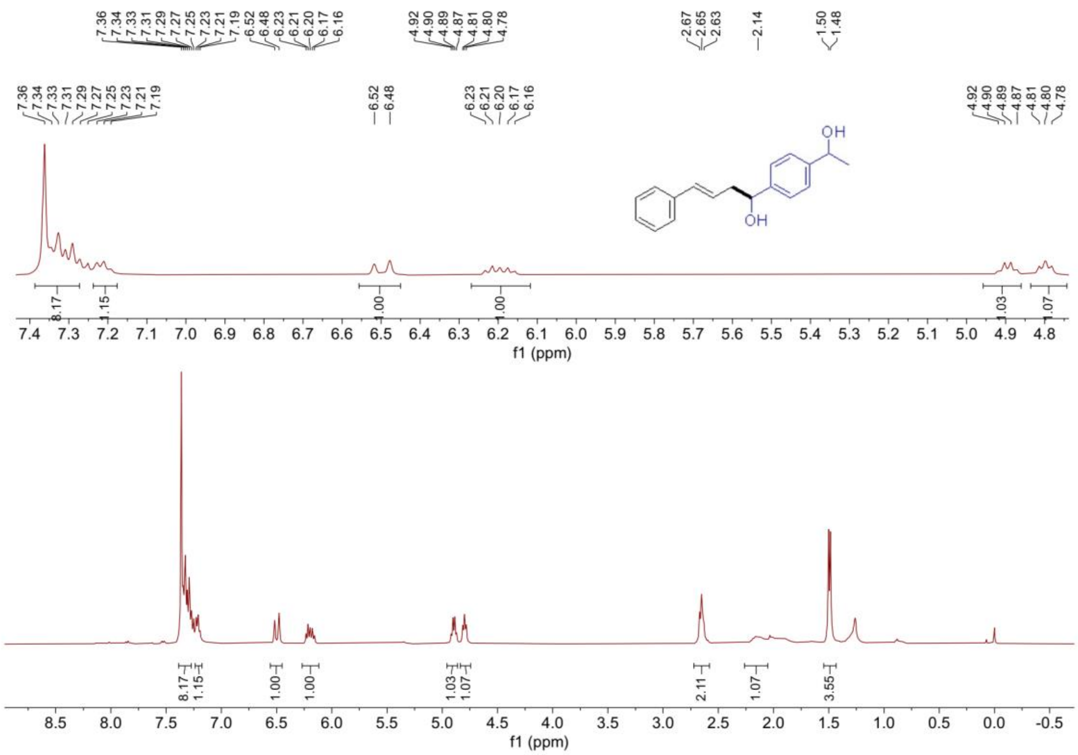

3bf ^{13}C NMR

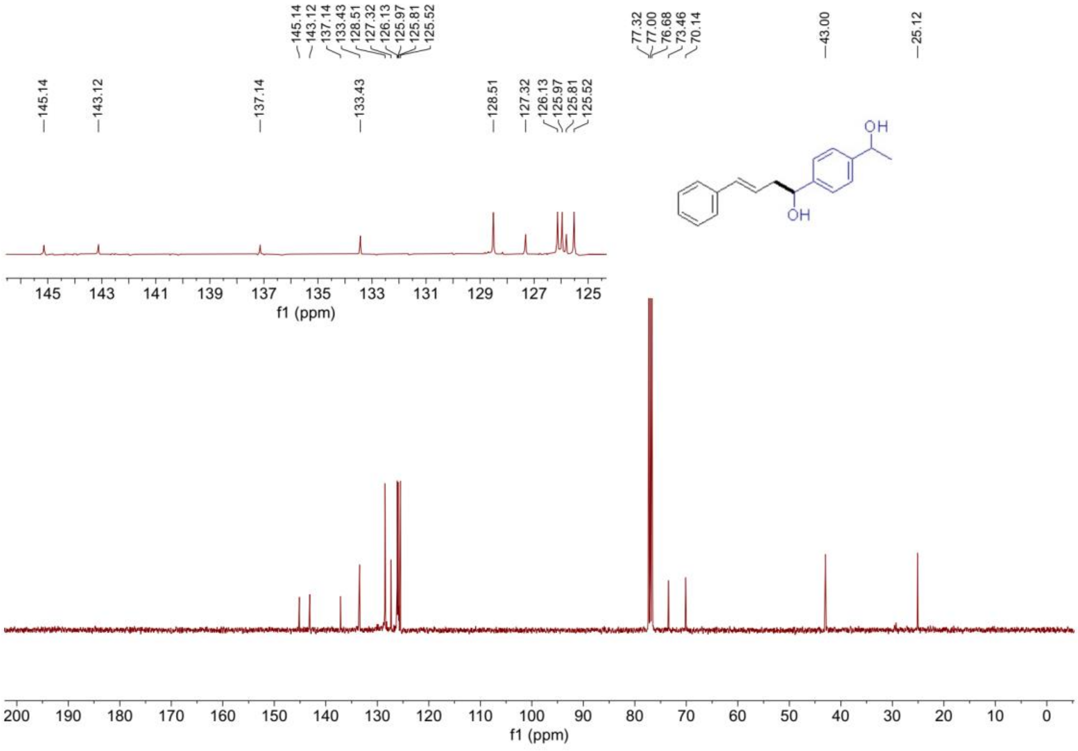

3bg ^{1}H NMR

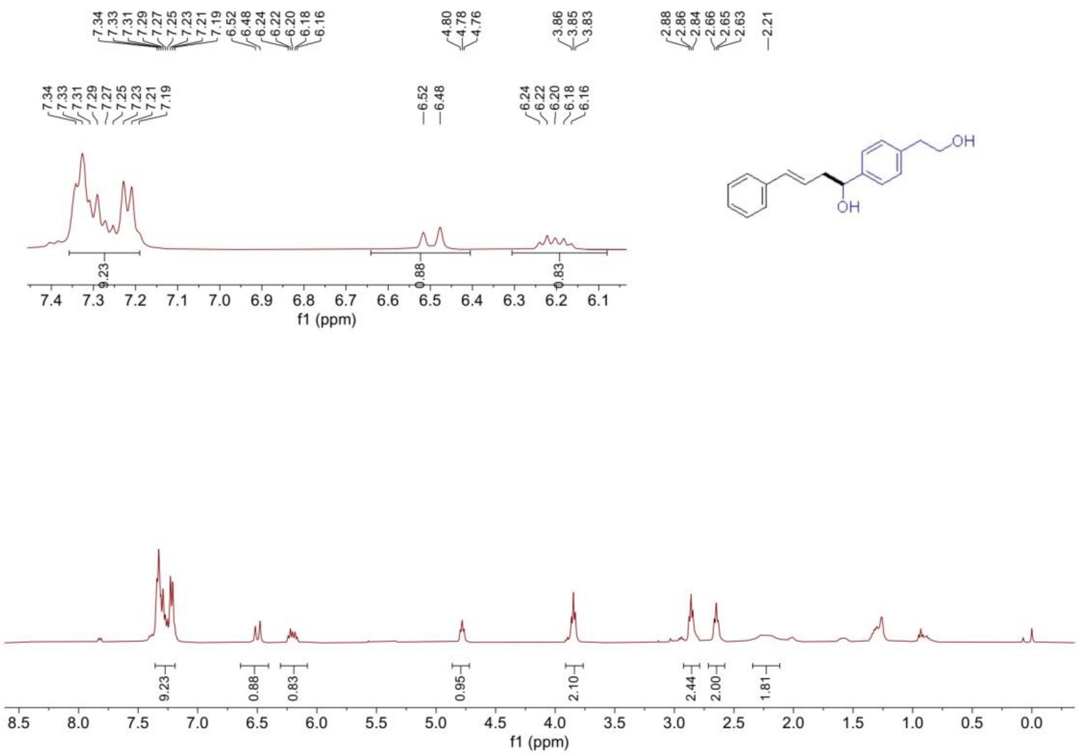

3bg ^{13}C NMR

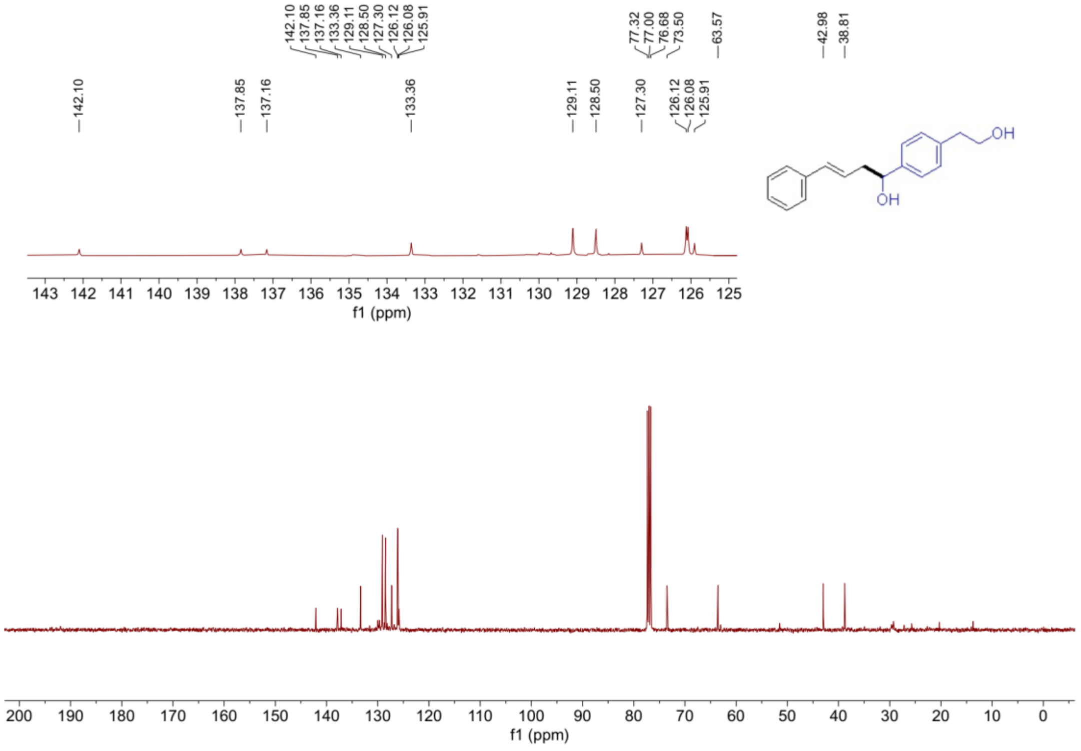

3bh ^{1}H NMR

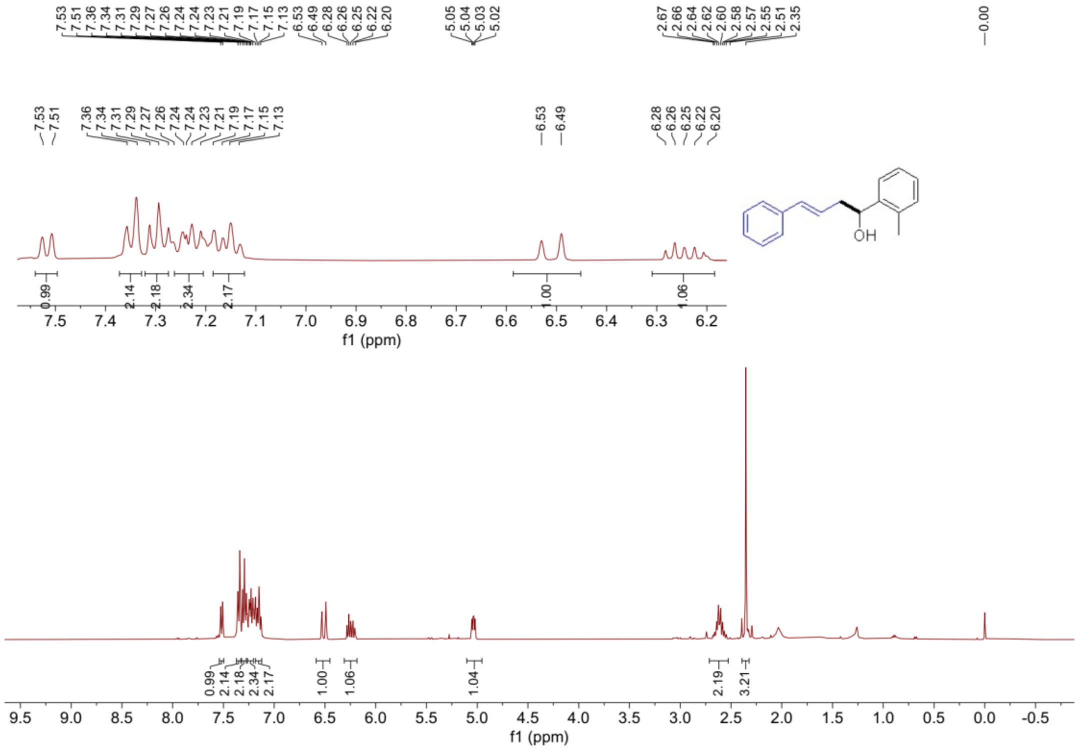

3bh ^{13}C NMR

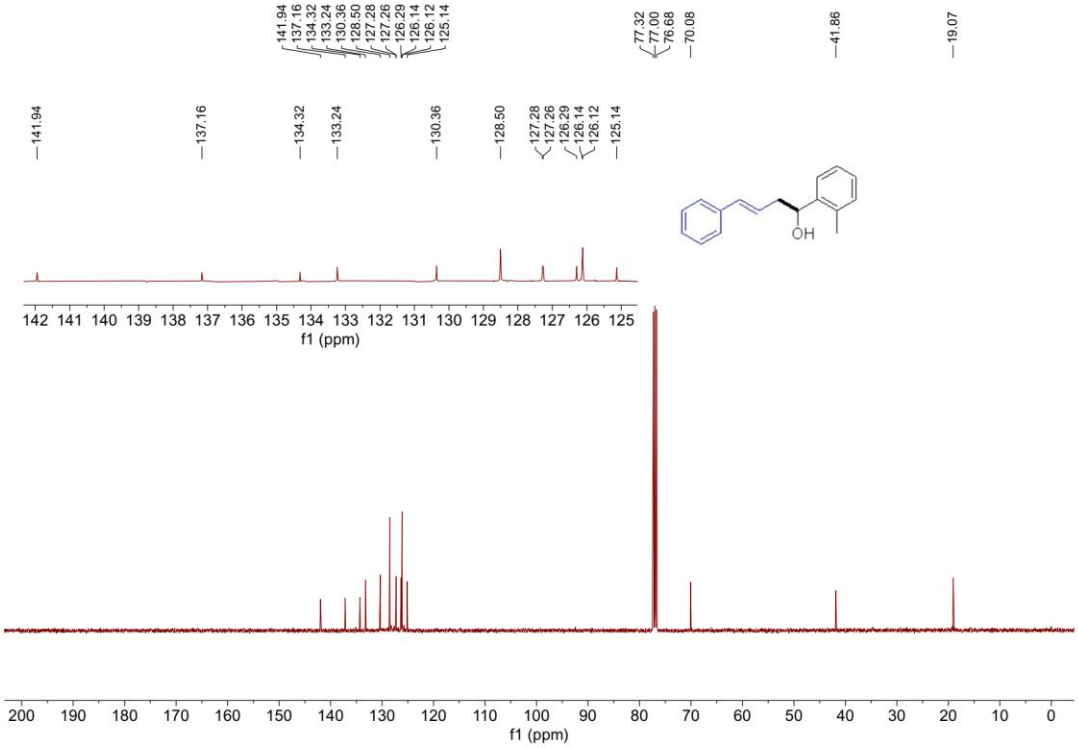

3bi ^{1}H NMR

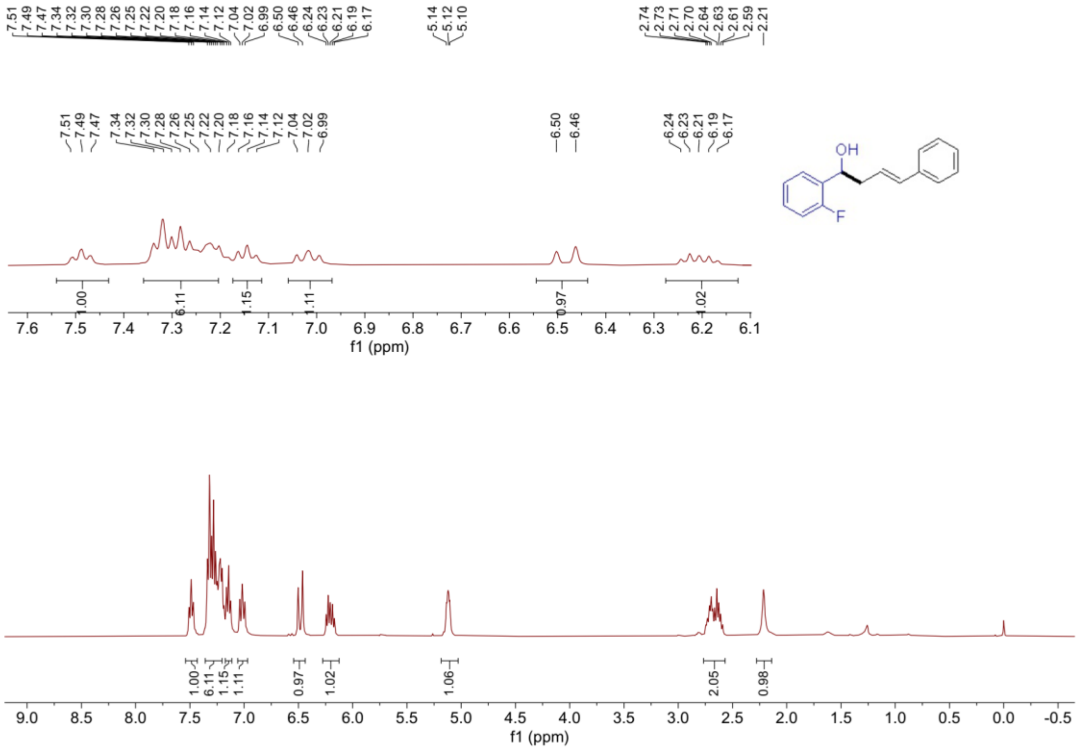

3bi ^{13}C NMR

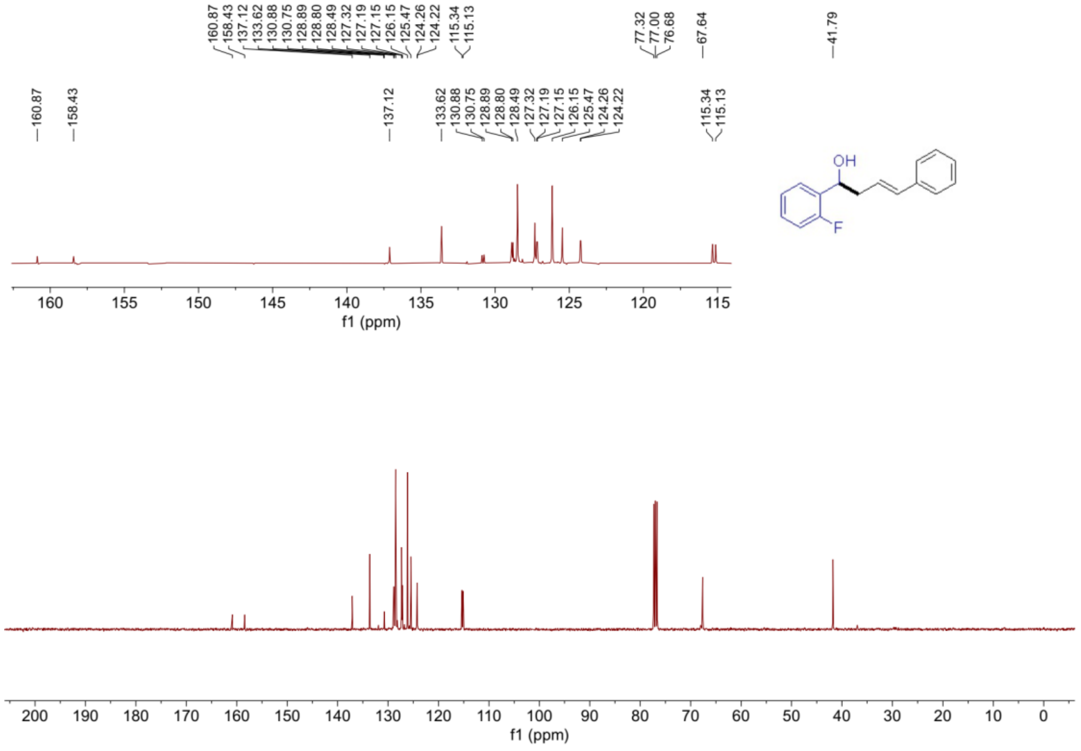

3bj ^{1}H NMR

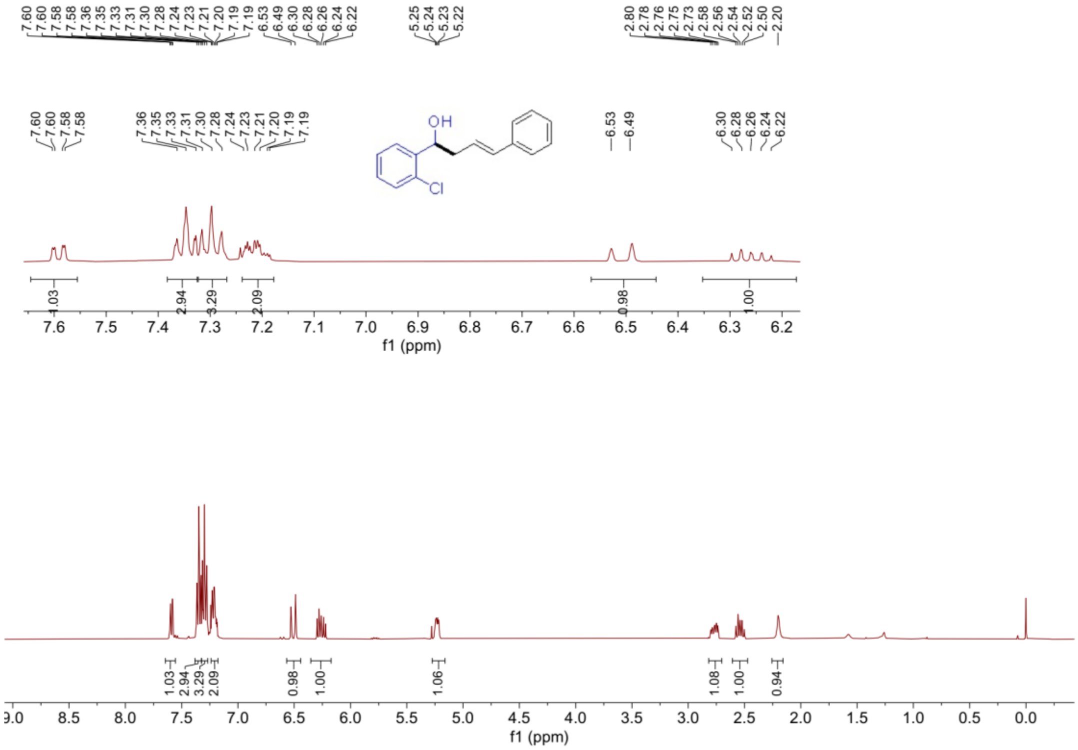

3bj ^{13}C NMR

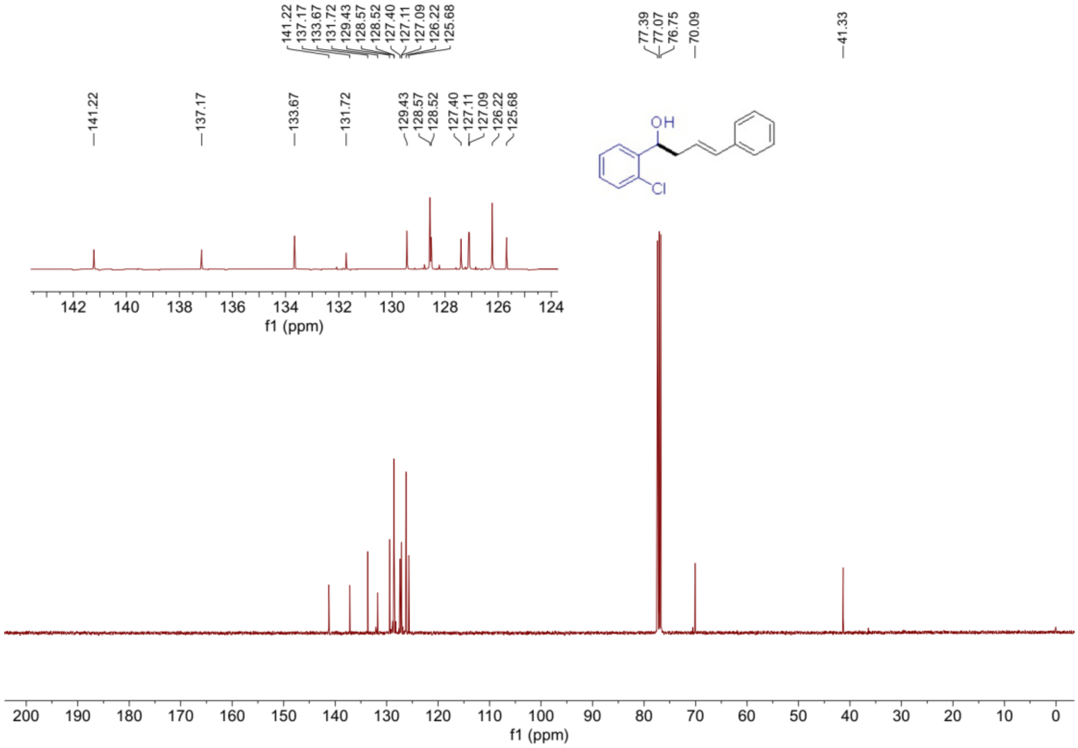

3bk ^{1}H NMR

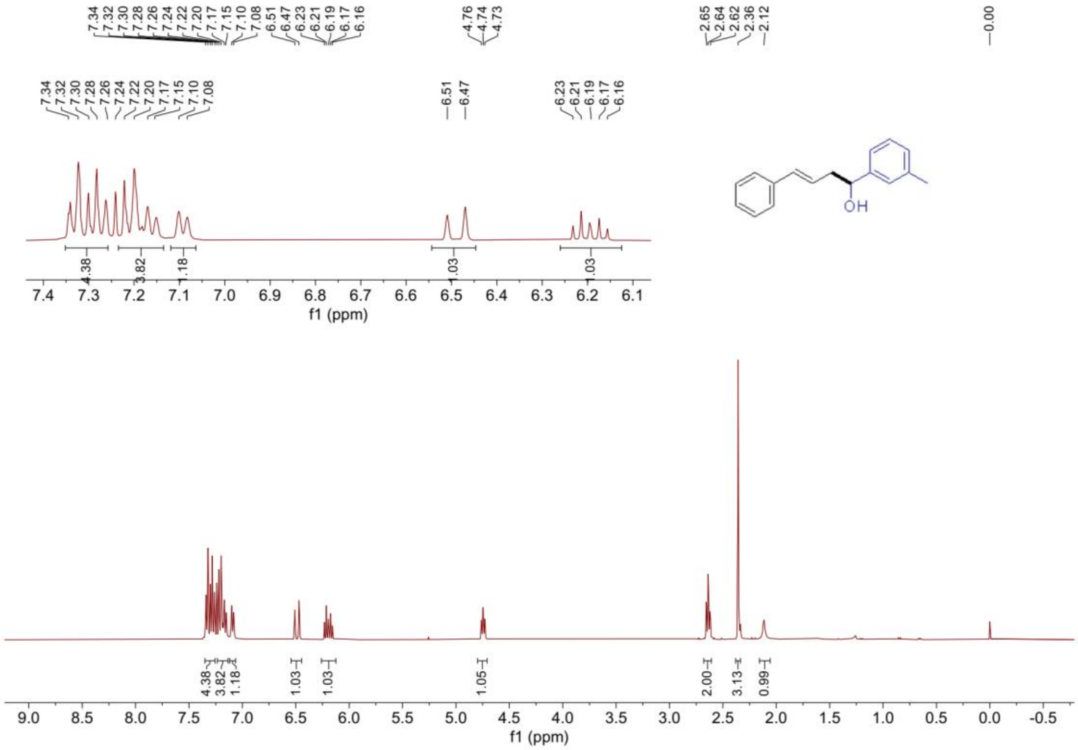

3bk ^{13}C NMR

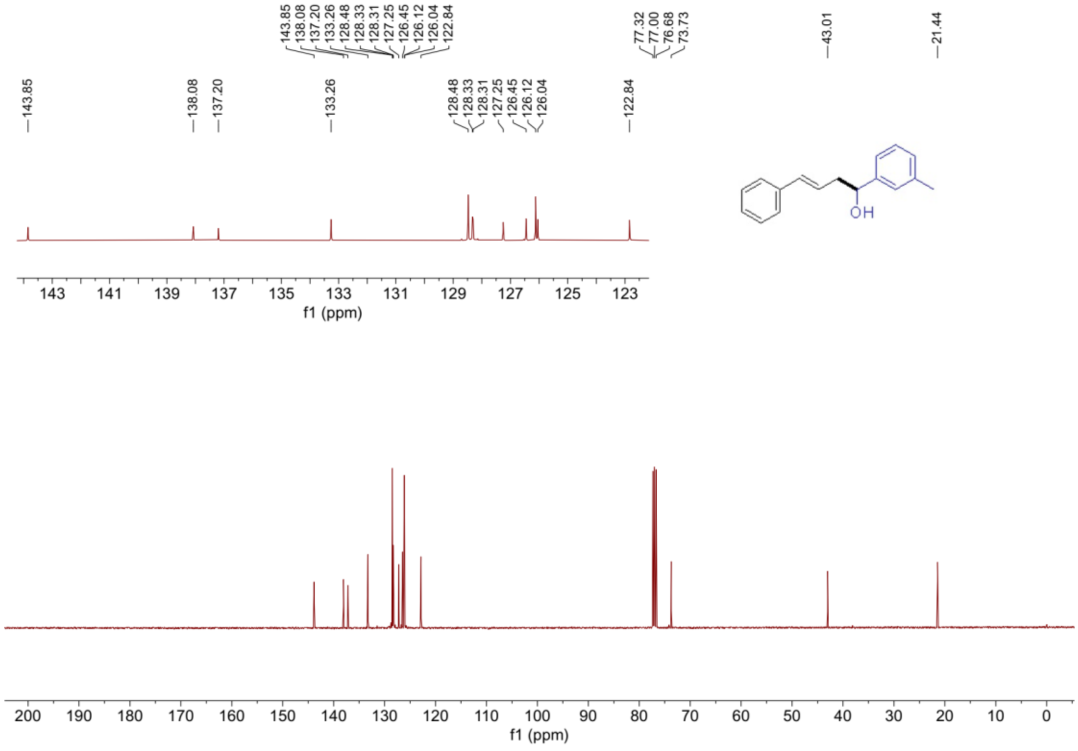

3bl ^{1}H NMR

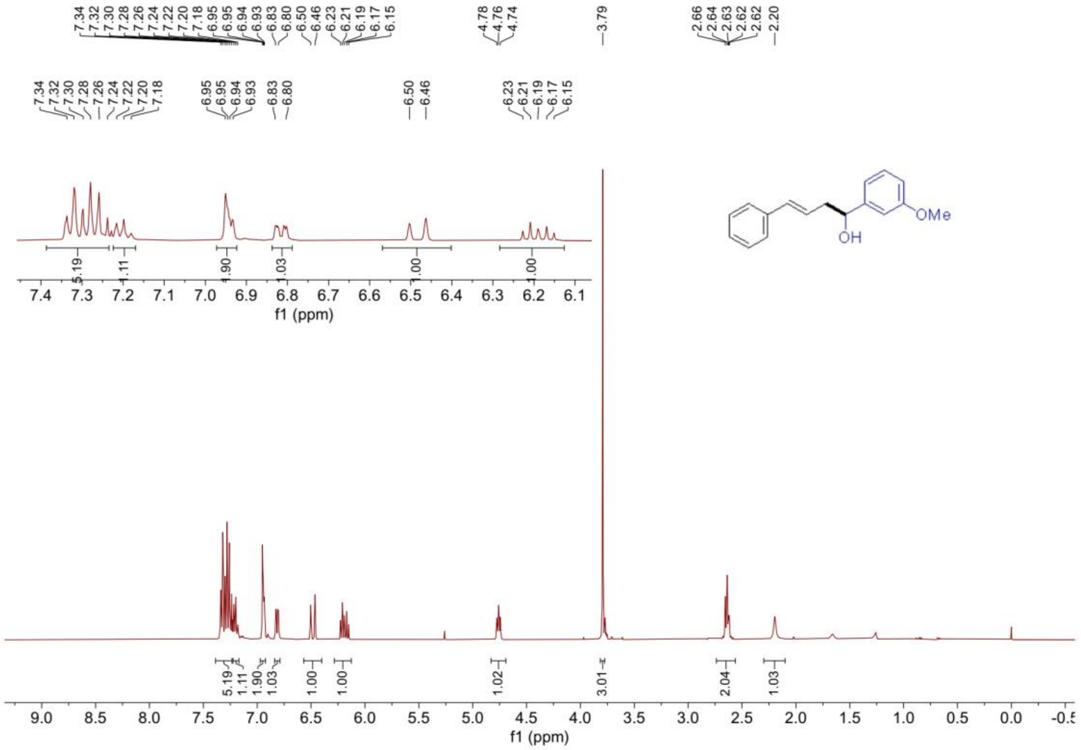

3bl ^{13}C NMR

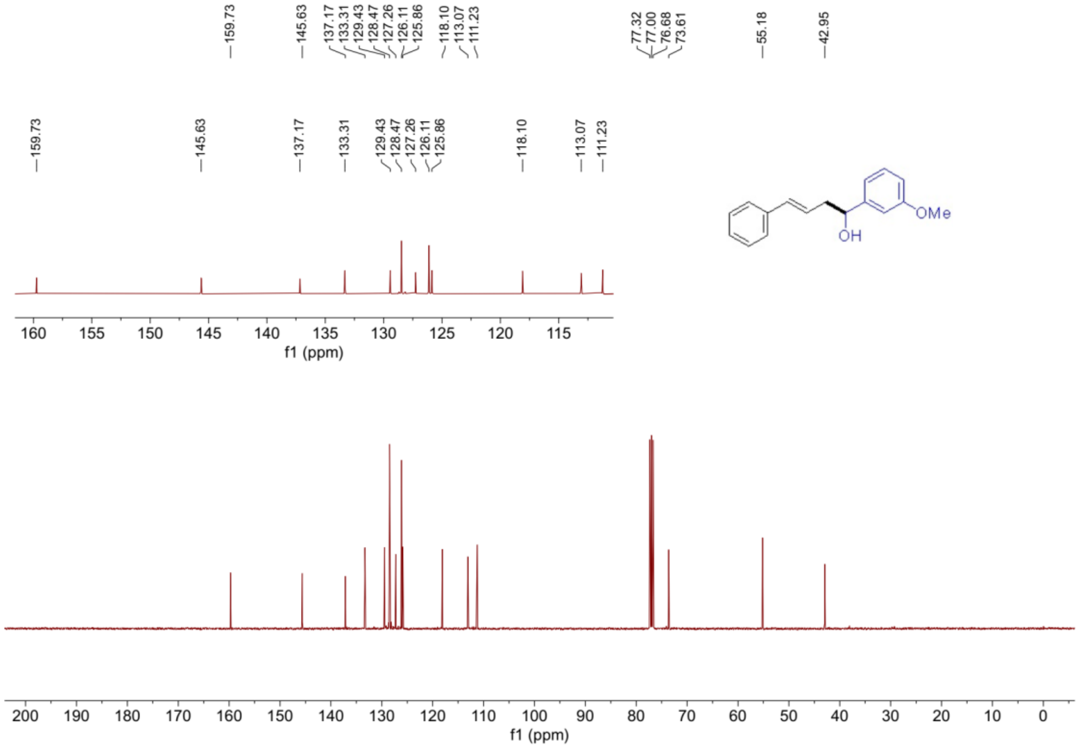

3bm ^{1}H NMR

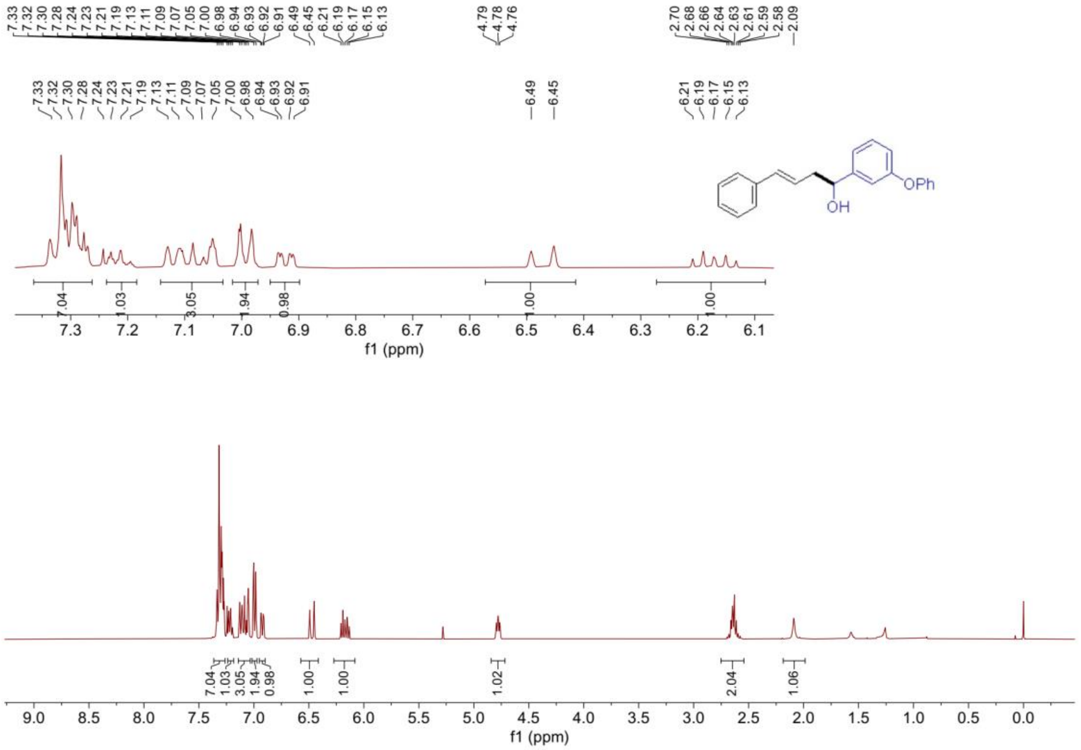

3bm ^{13}C NMR

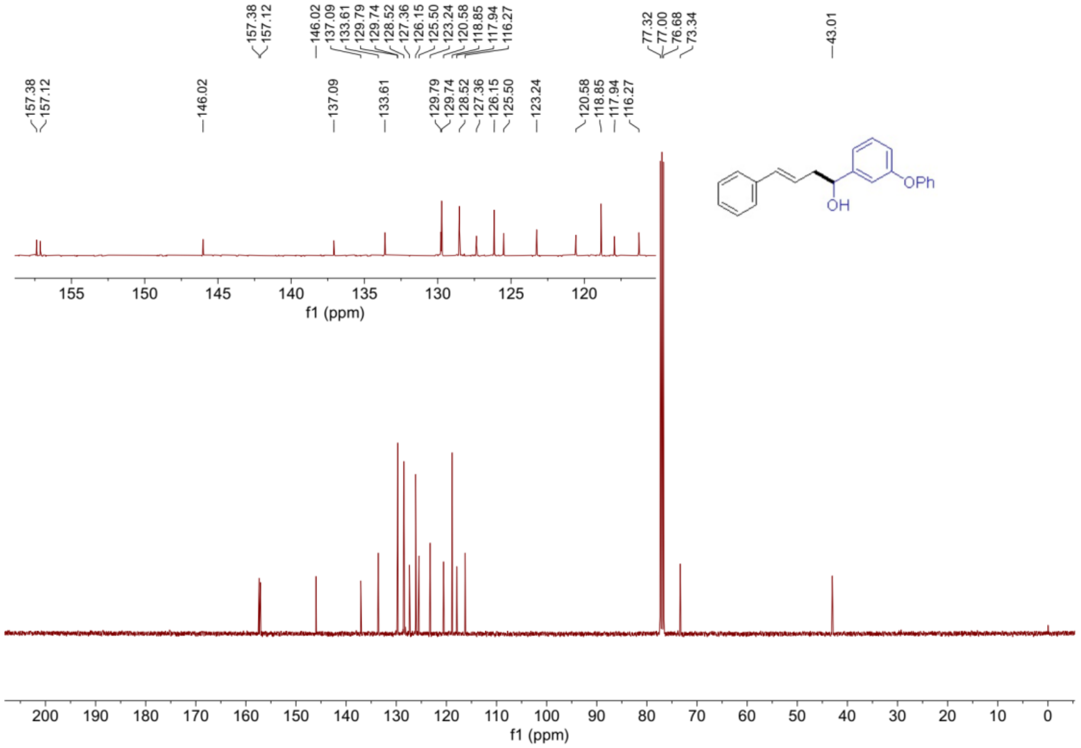

3bn ^{1}H NMR

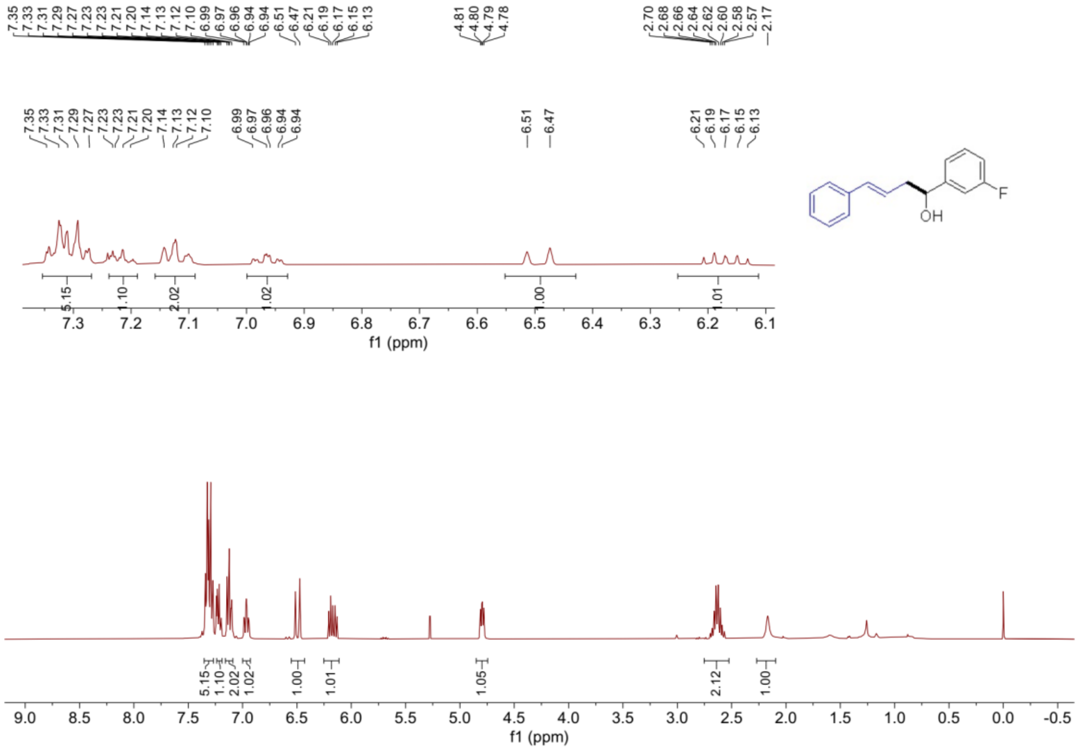

3bn ^{13}C NMR

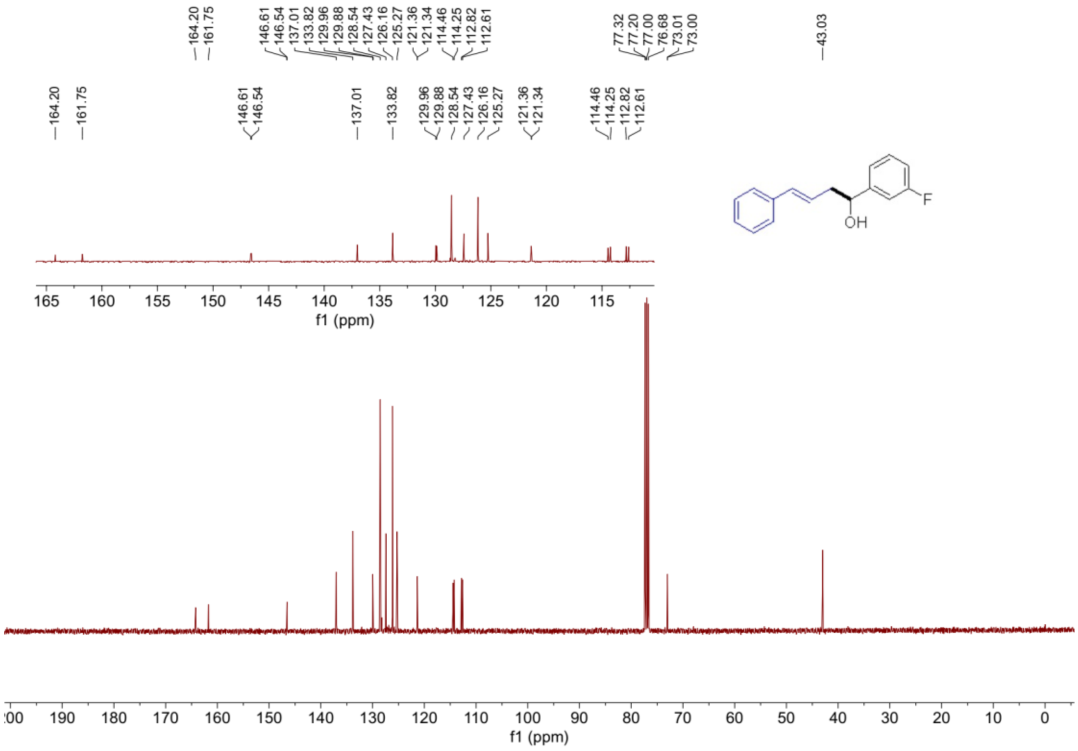

3bo ^{1}H NMR

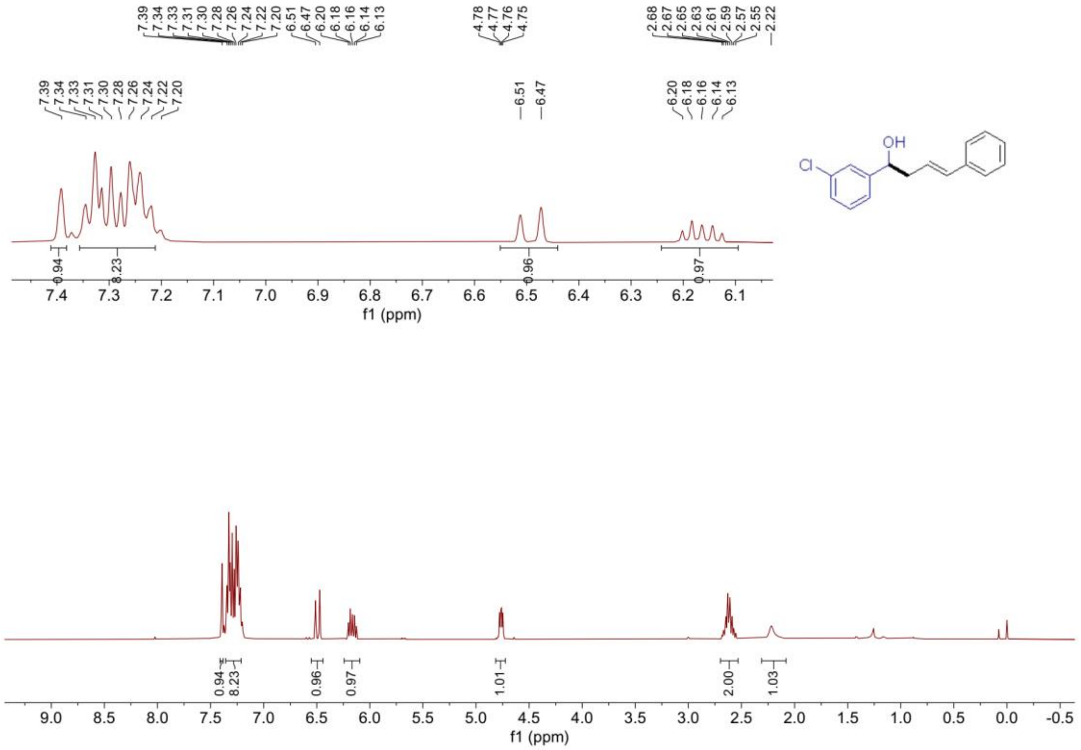

3bo ^{13}C NMR

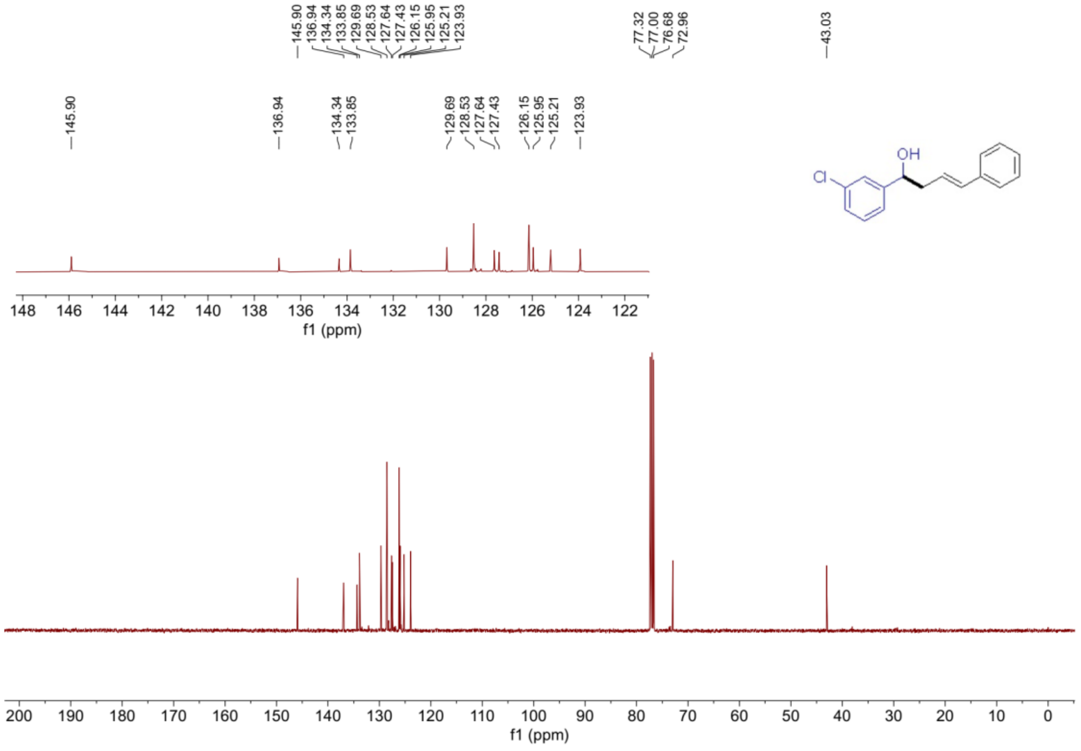

3bp ^{1}H NMR

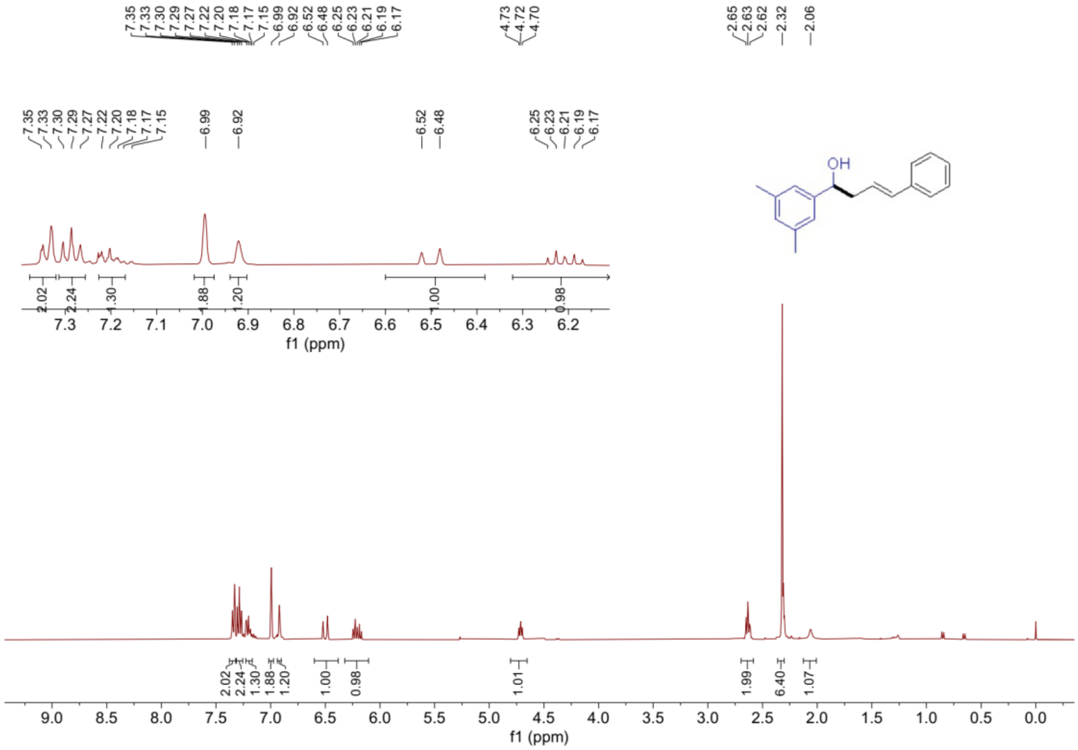

3bp ^{13}C NMR

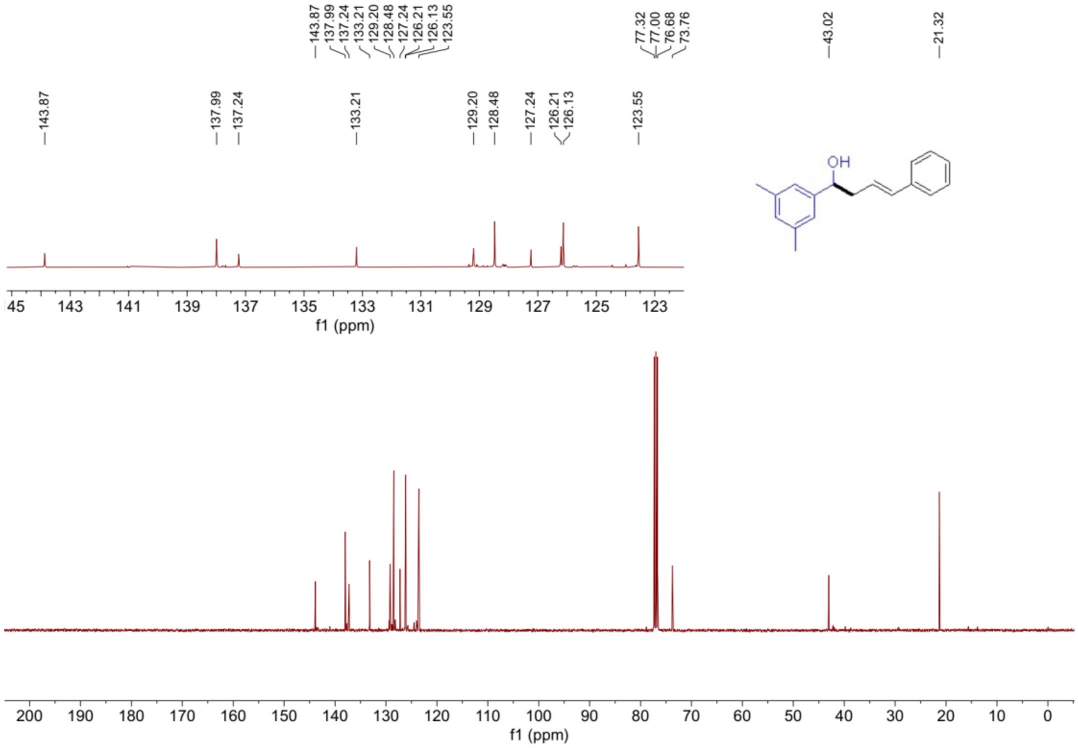

3bq ^{1}H NMR

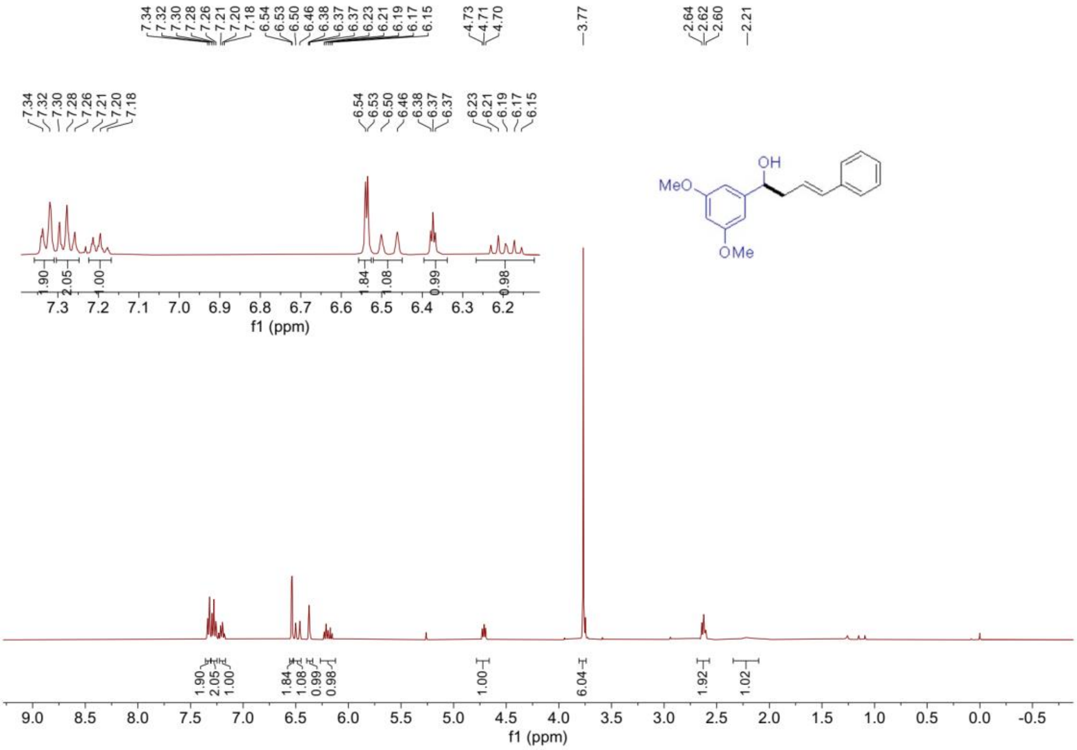

3bq ^{13}C NMR

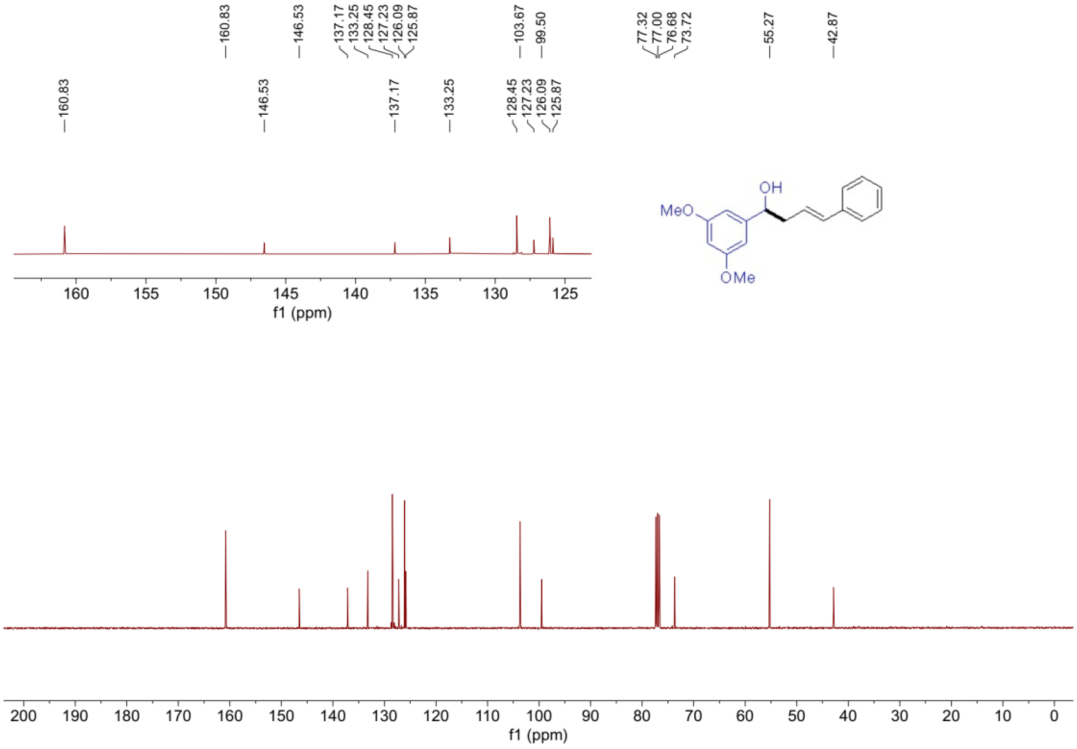

3br ^{1}H NMR

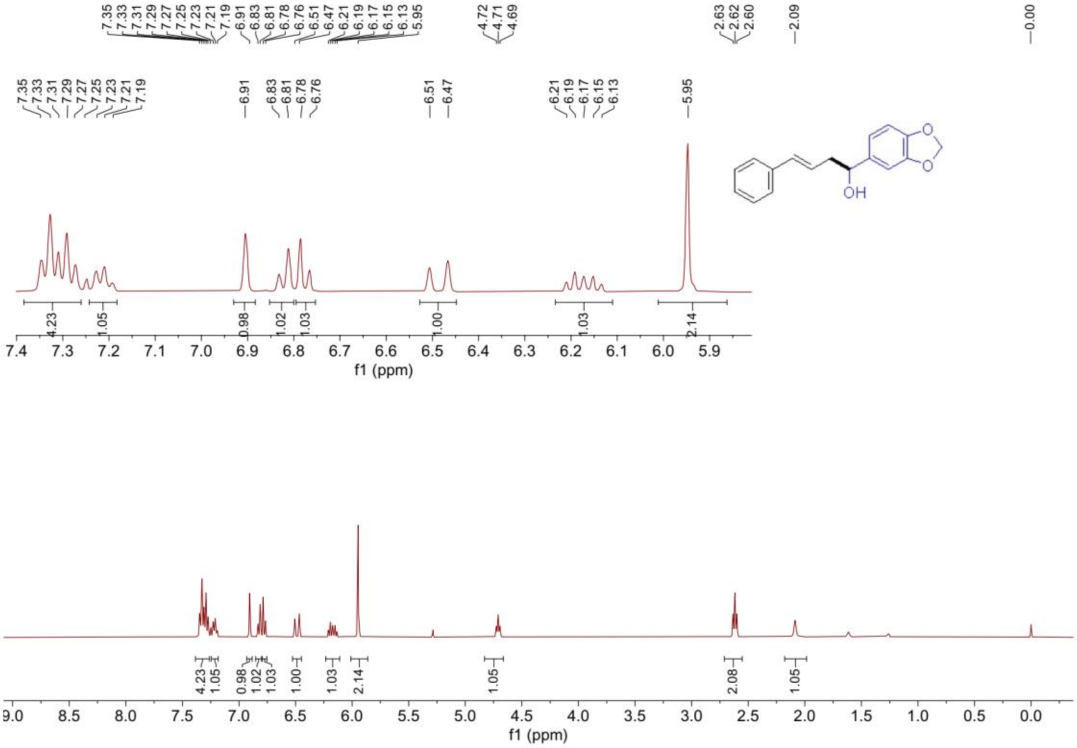

3br ^{13}C NMR

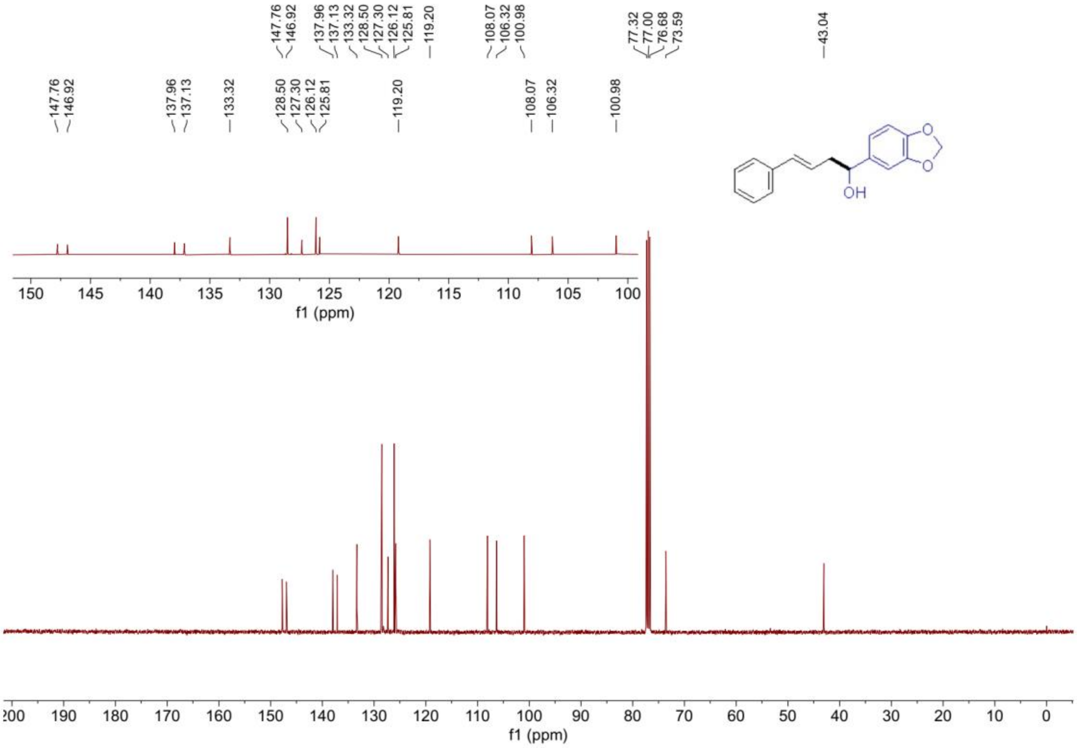

3bs ^{1}H NMR

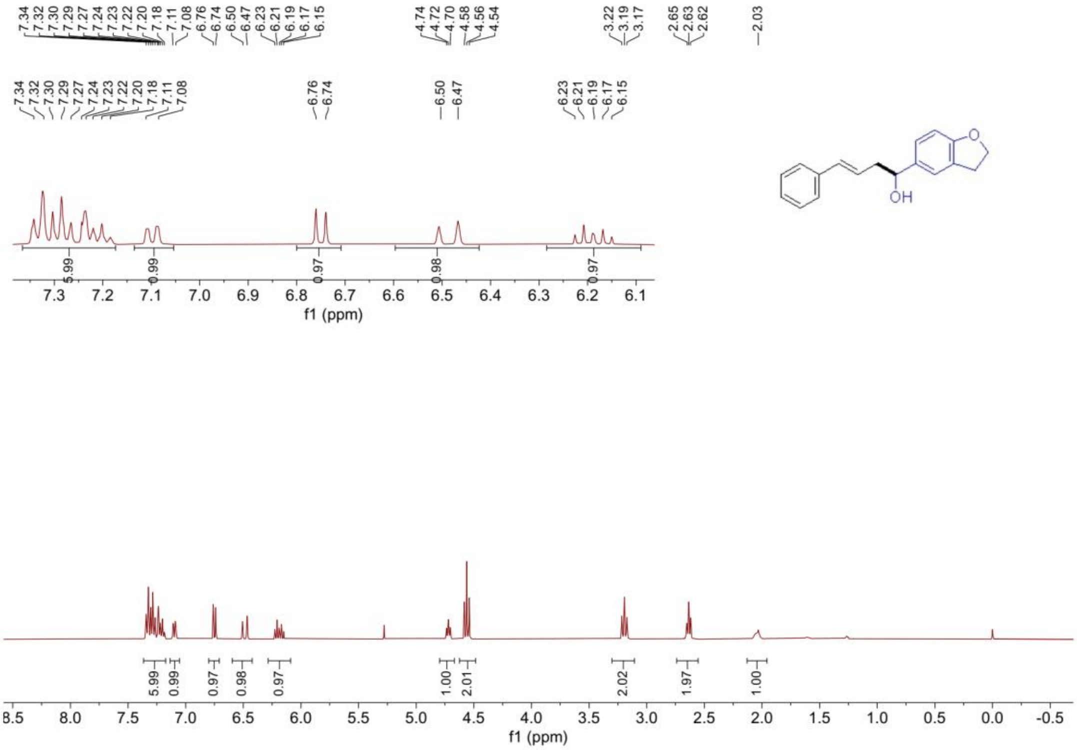

3bs ^{13}C NMR

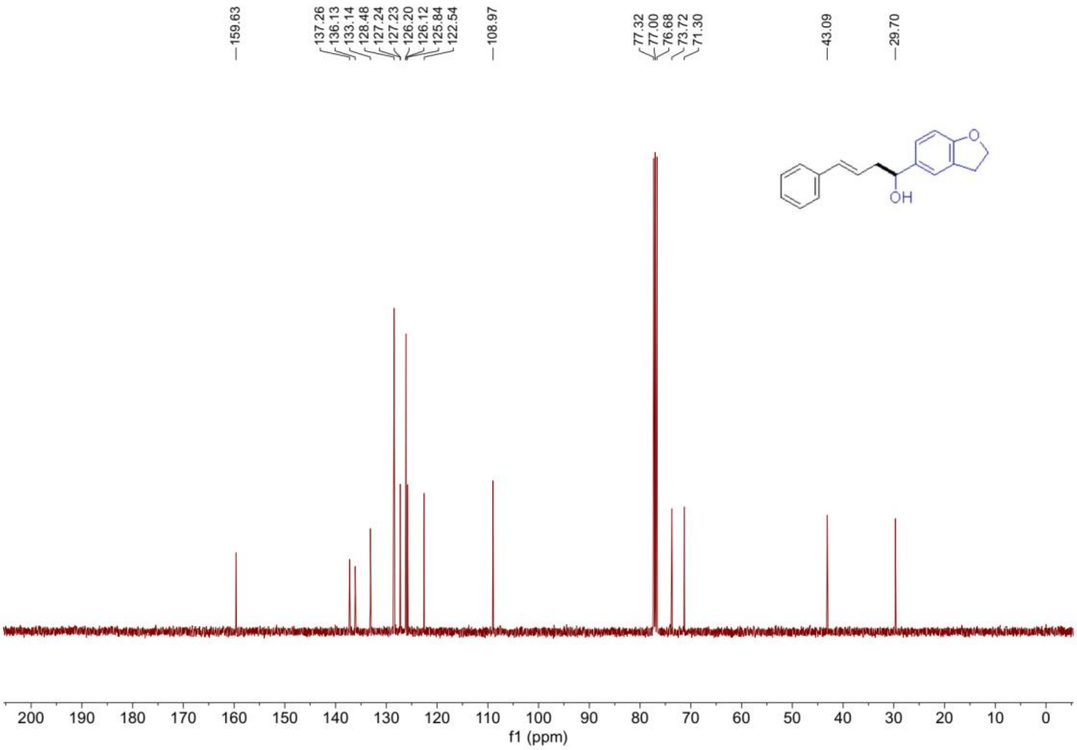

3bt ^{1}H NMR

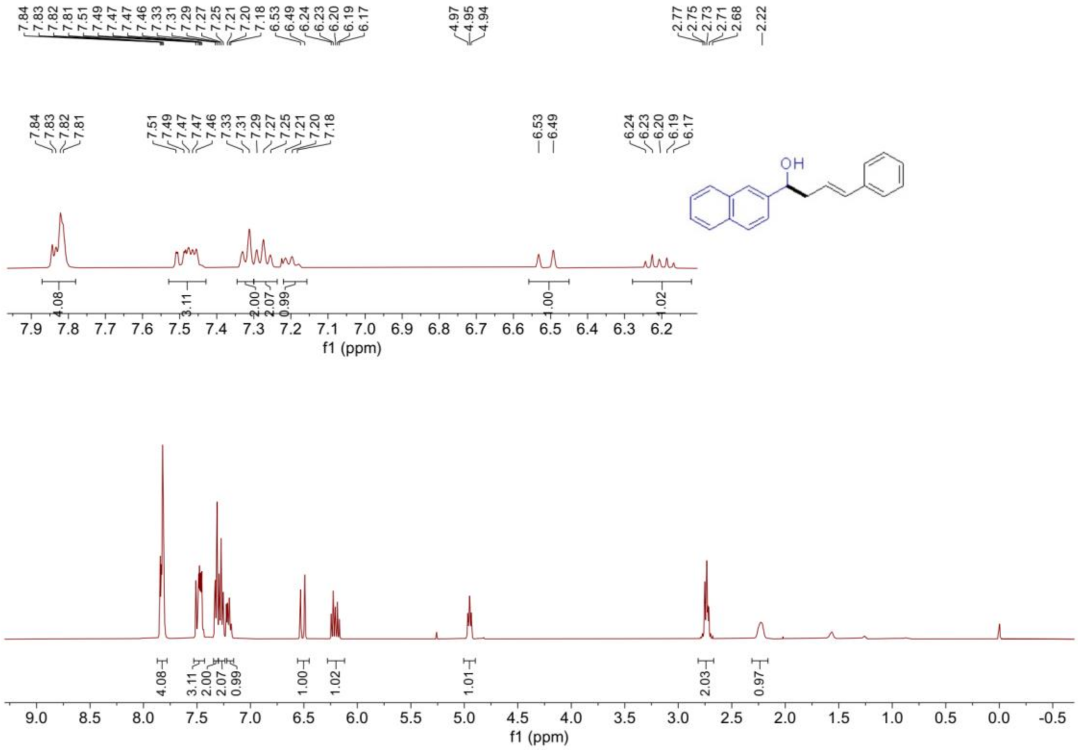

3bt ^{13}C NMR

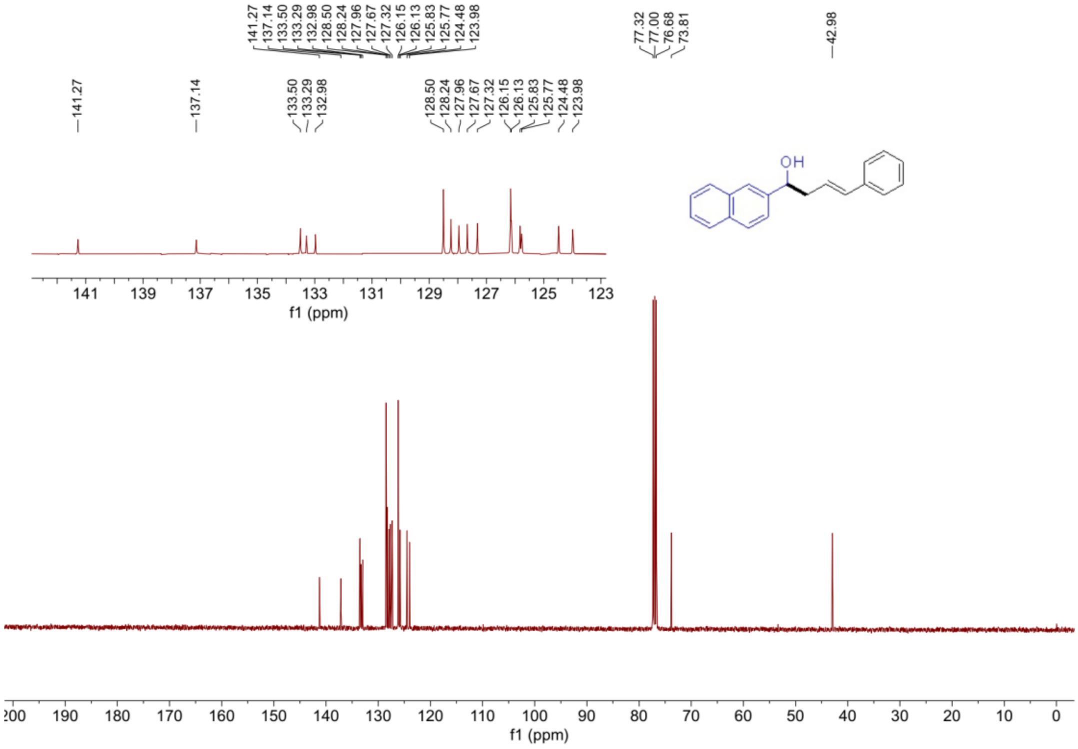

3bu ^{1}H NMR

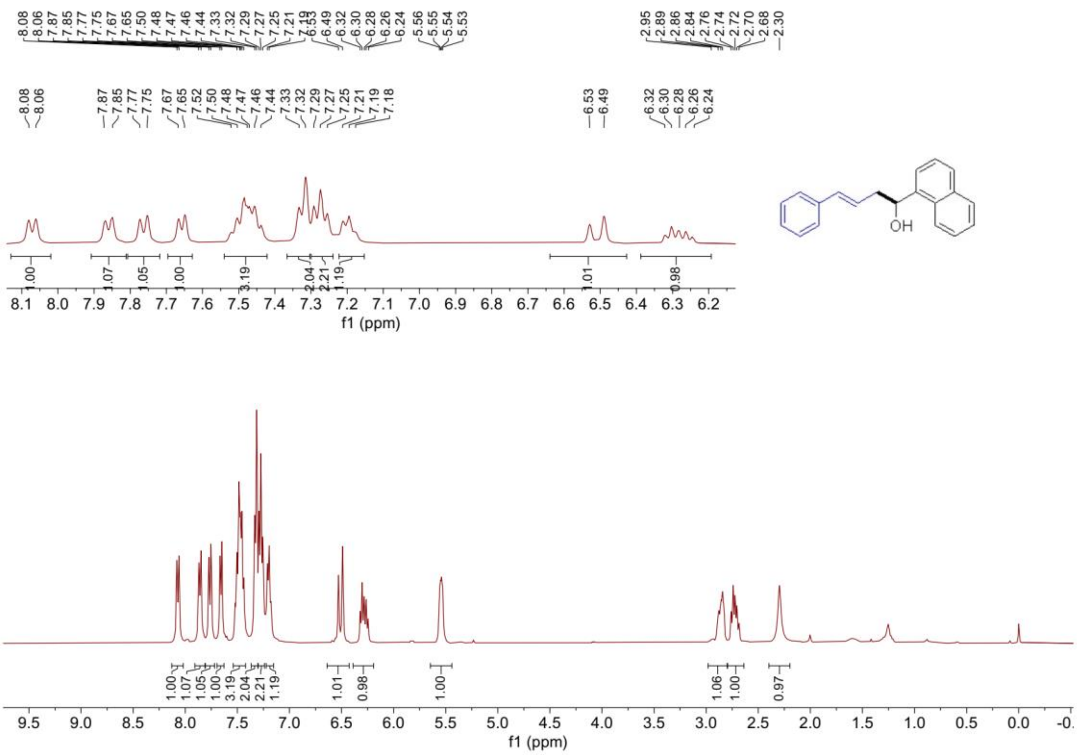

3bu ^{13}C NMR

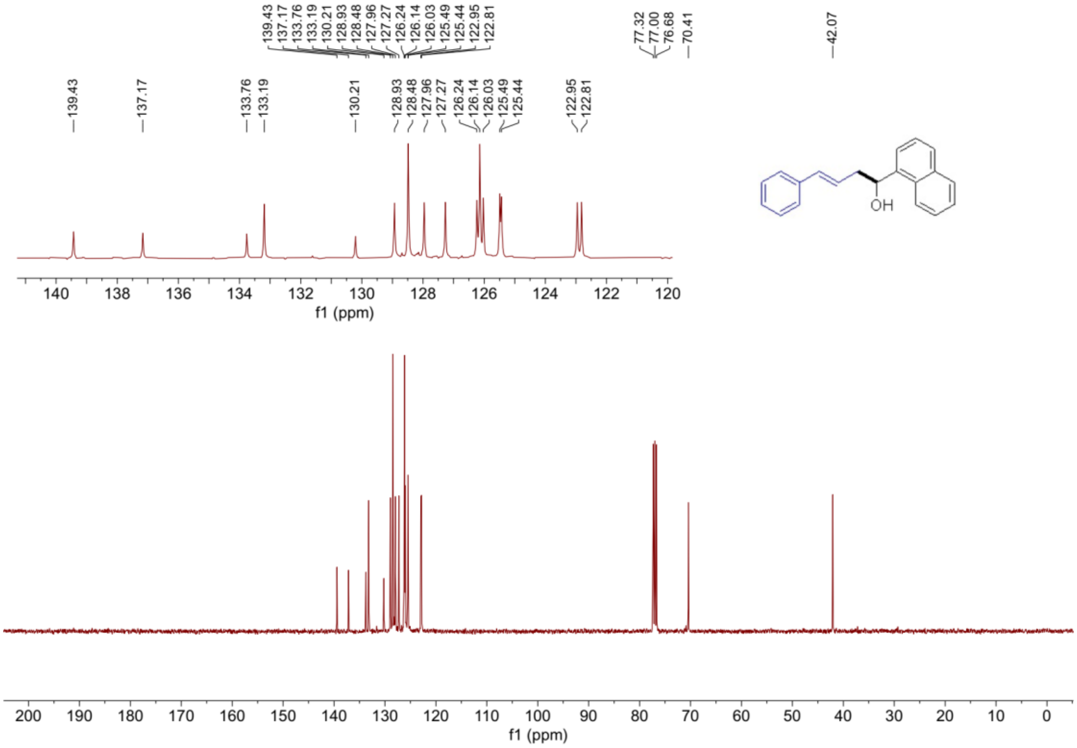

3bv ^{1}H NMR

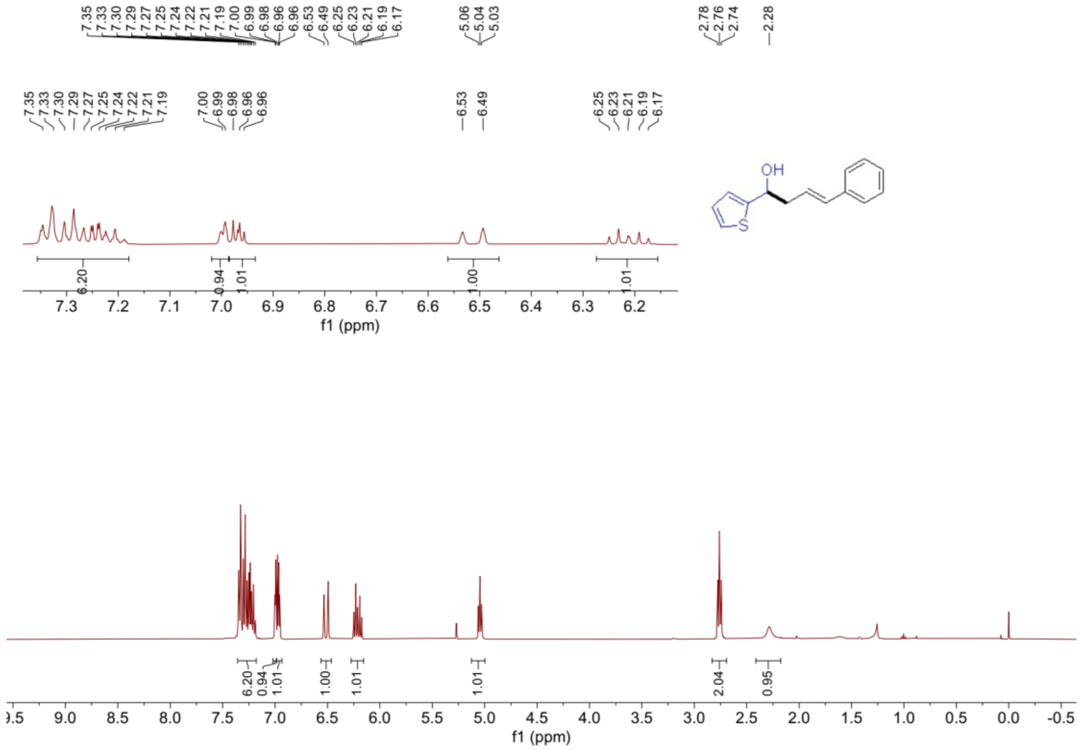

3bv ^{13}C NMR

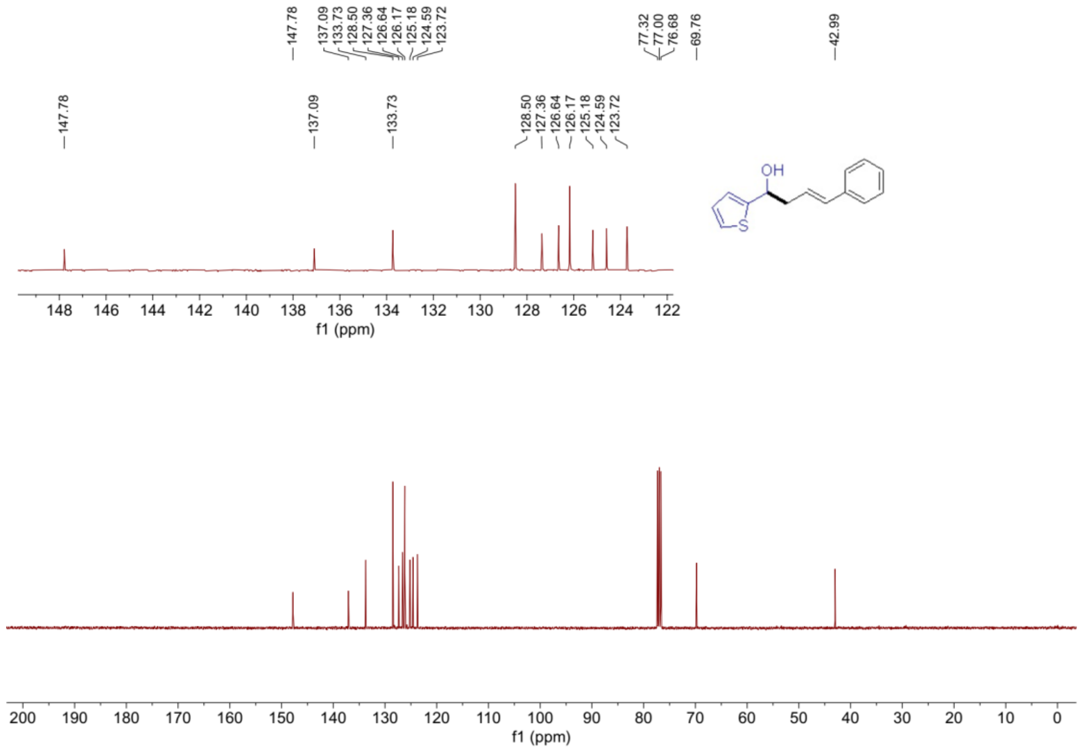

3bw ^{1}H NMR

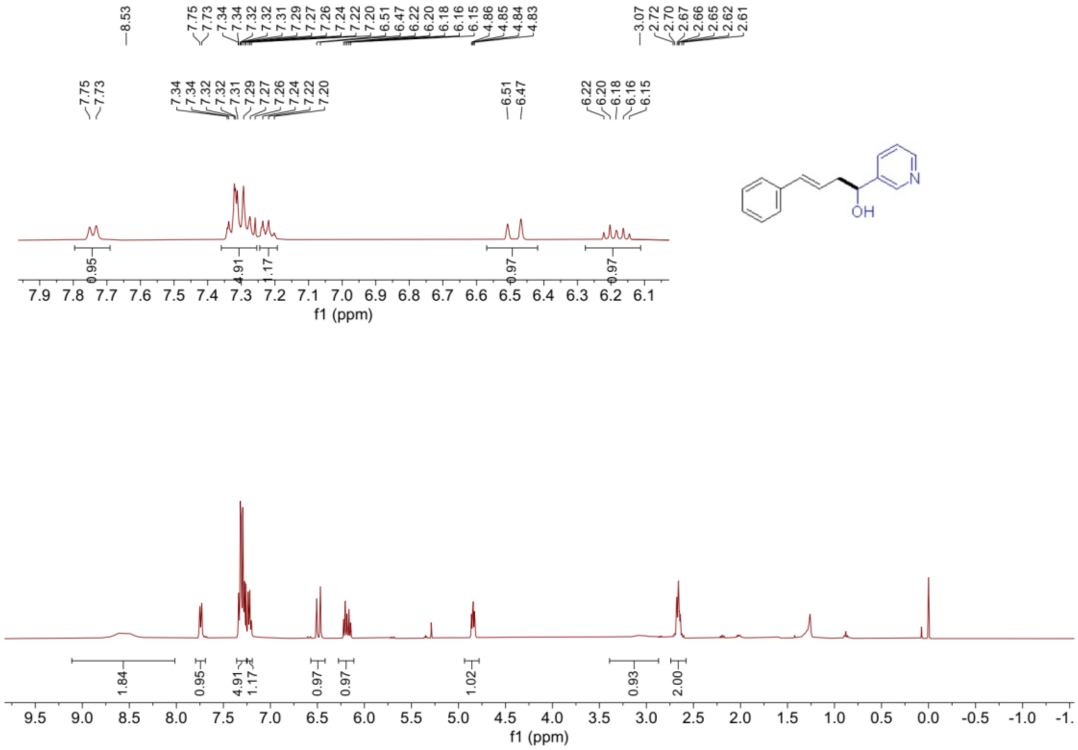

3bw ^{13}C NMR

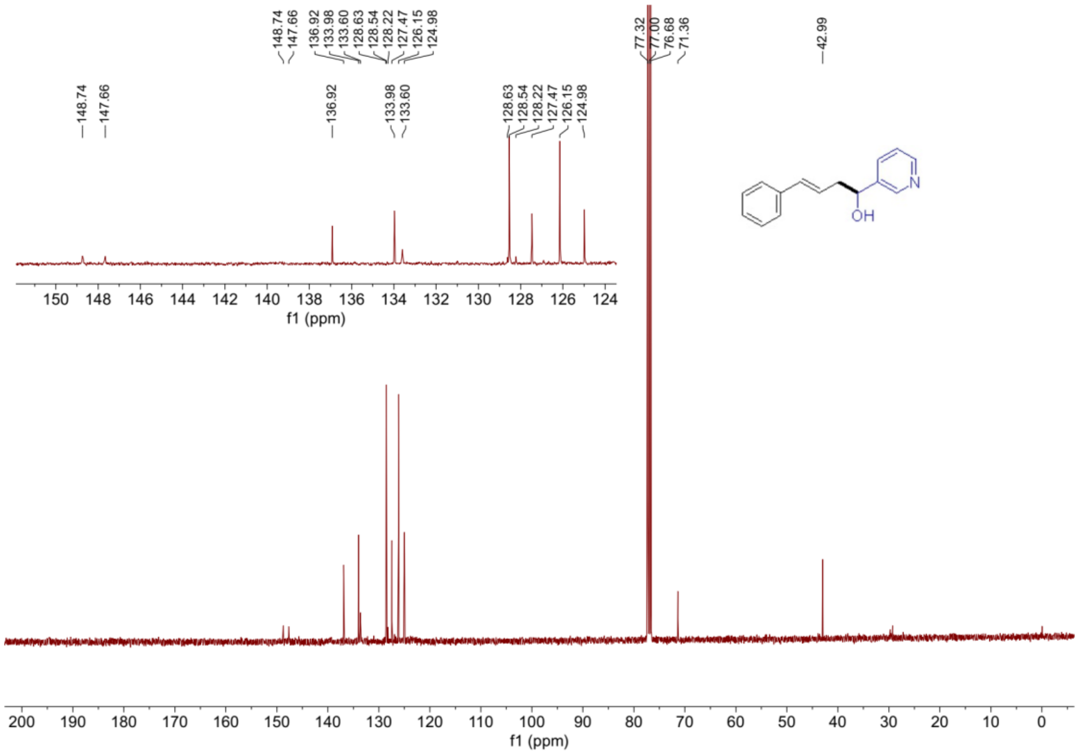

3bx ^{1}H NMR

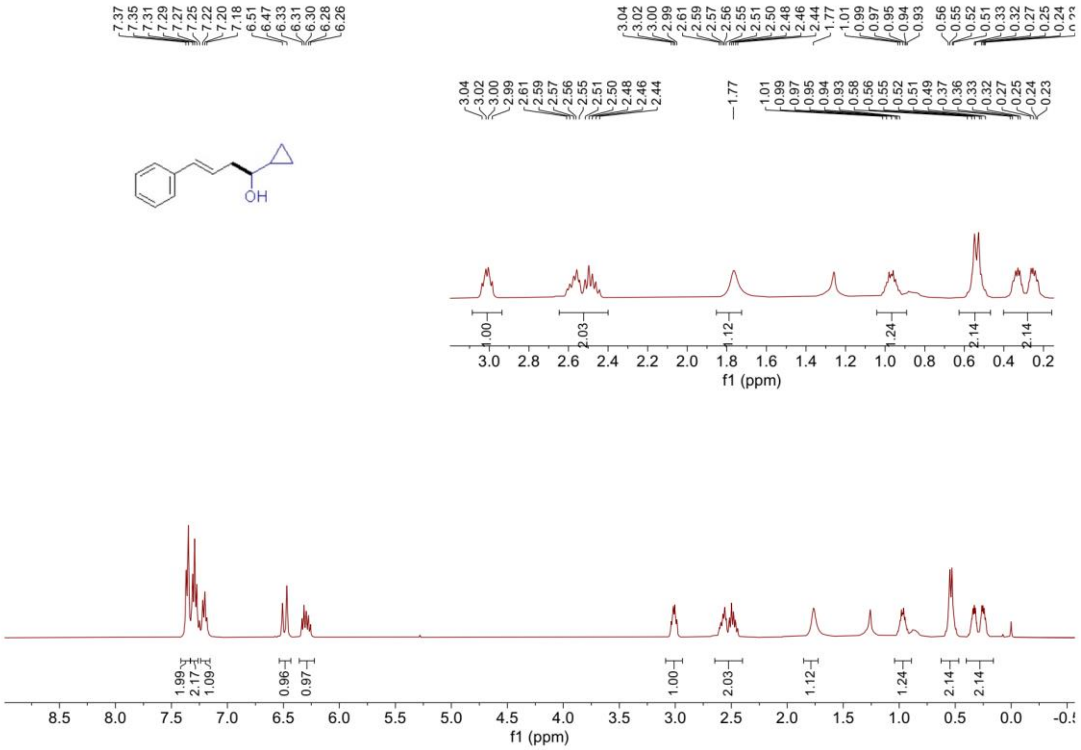

3bx ^{13}C NMR

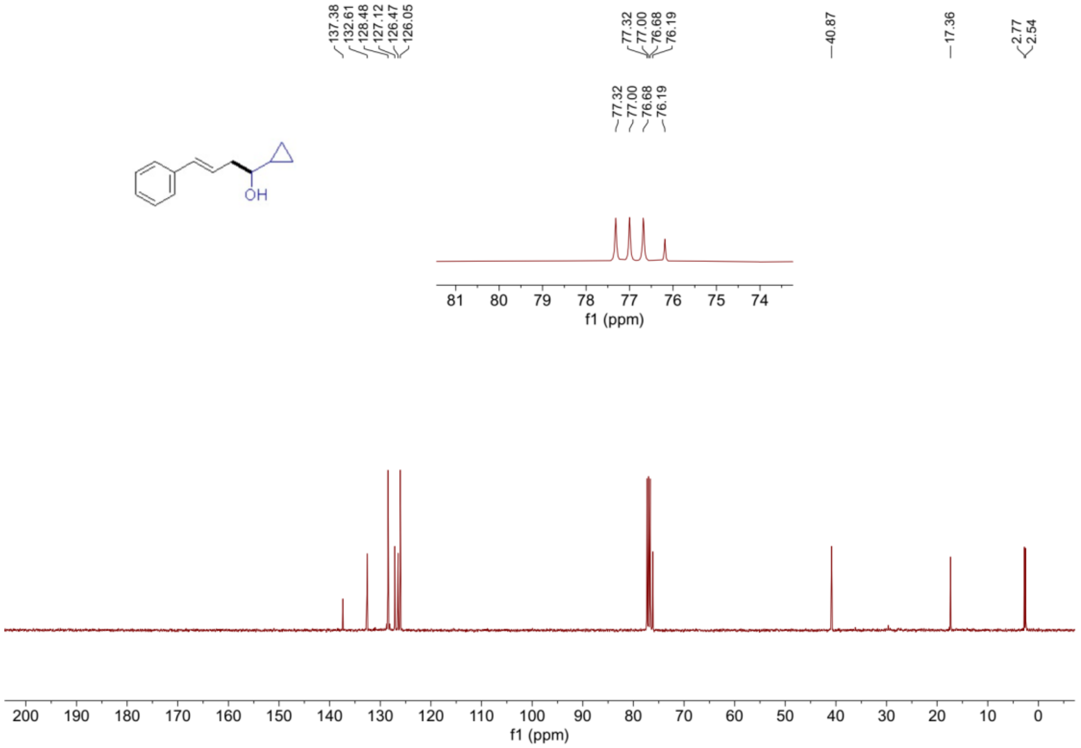

3by isomer I ^{1}H NMR

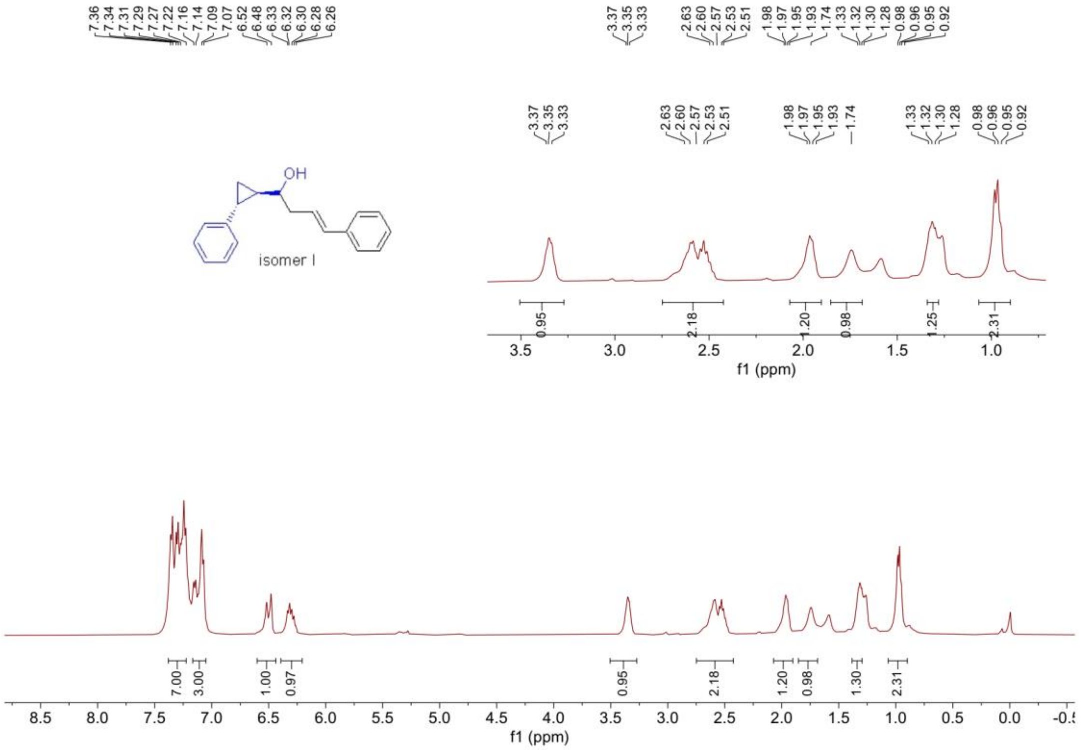

3by isomer I ^{13}C NMR

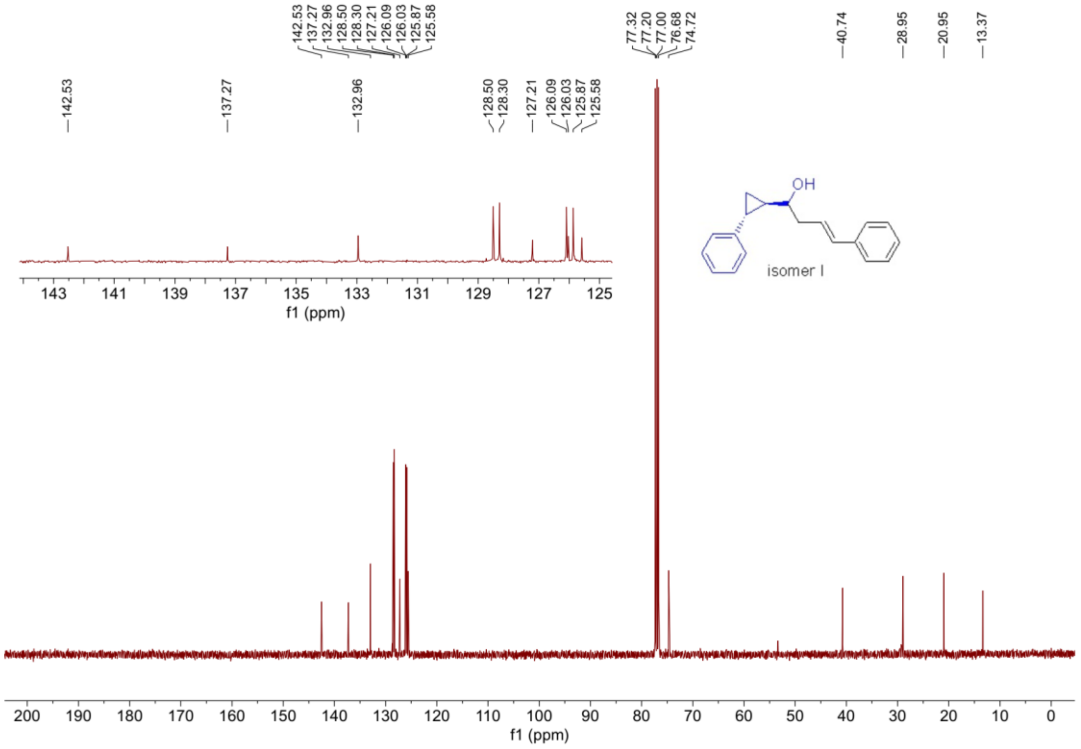

3by isomer II ^{1}H NMR

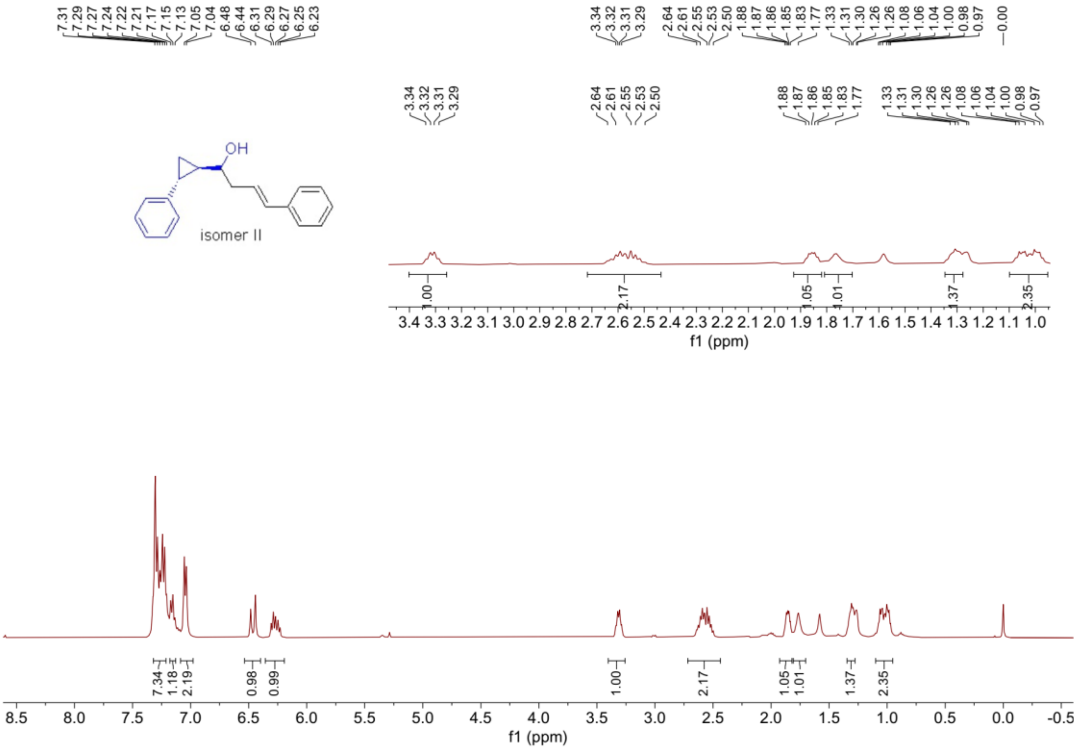

3by isomer II ^{13}C NMR

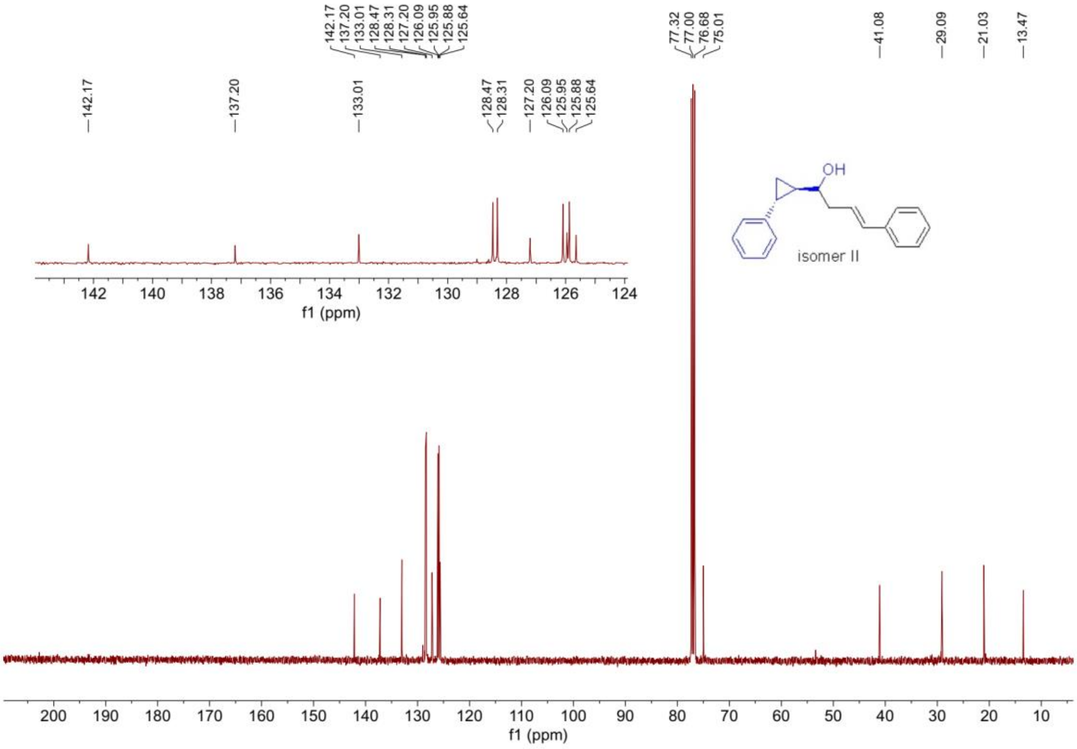

3bz ^{1}H NMR

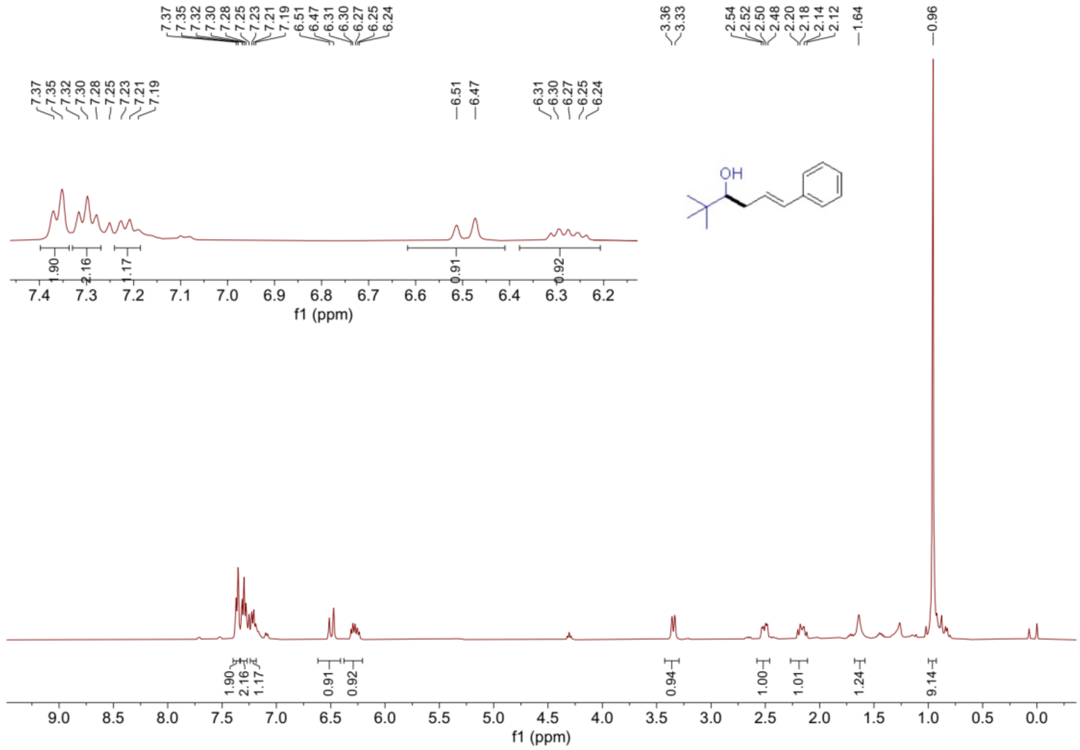

3bz ^{13}C NMR

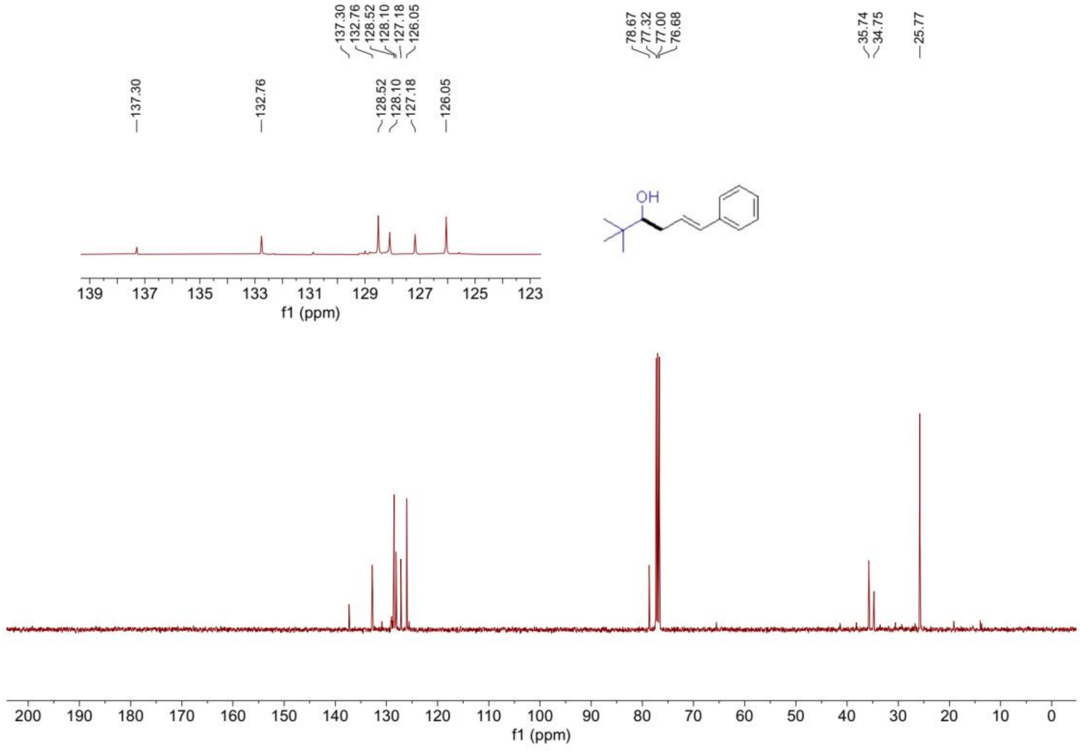

3ca ^{1}H NMR

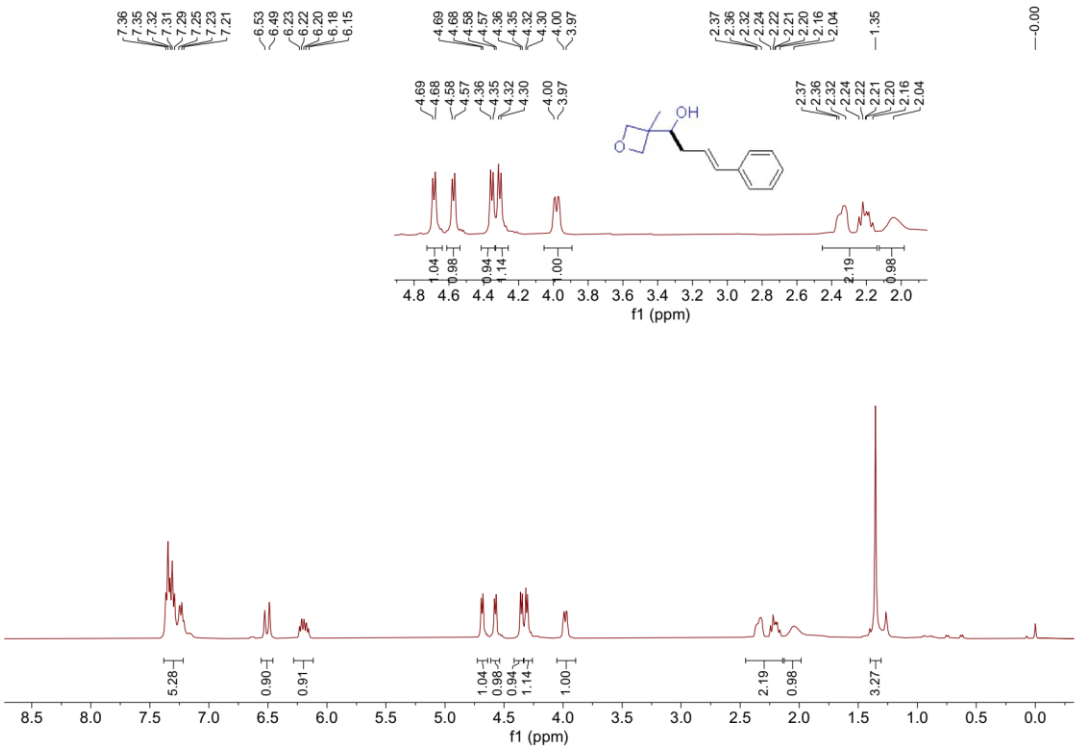

3ca ^{13}C NMR

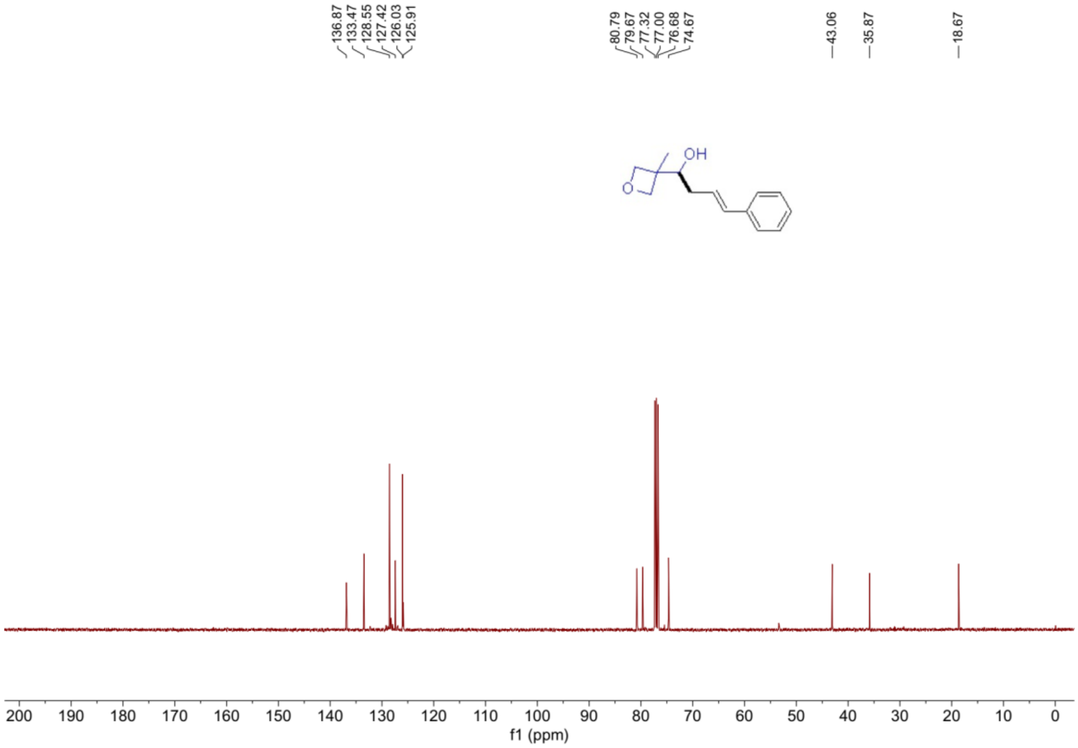

3cb ^{1}H NMR

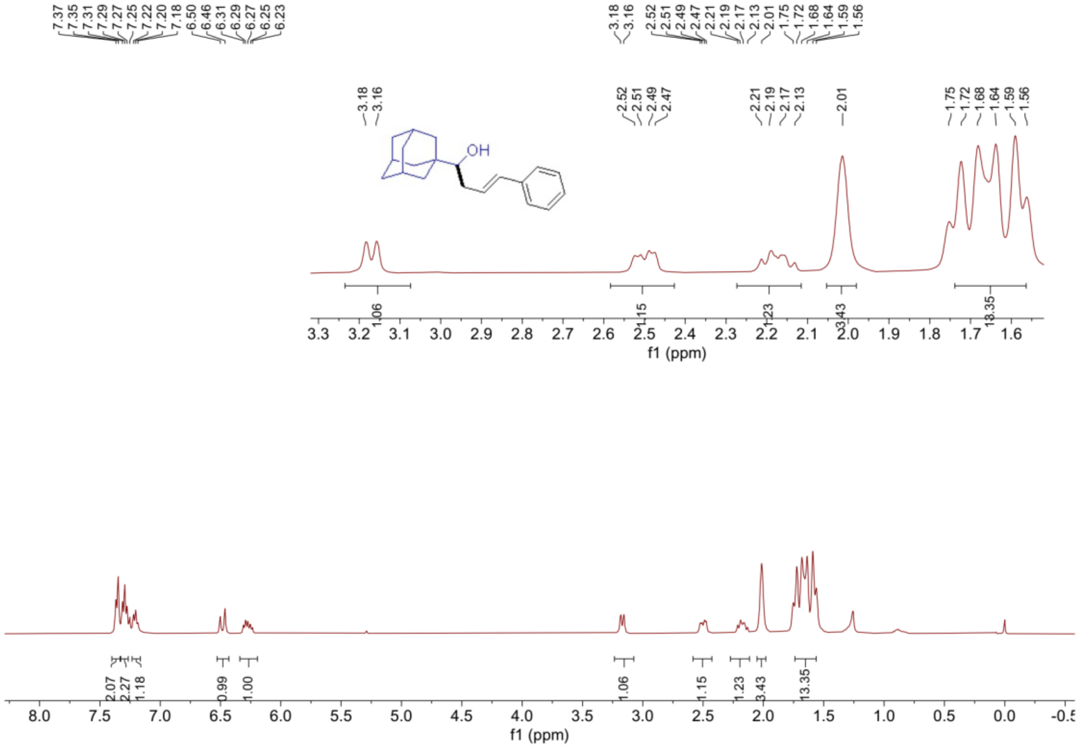

3cb ^{13}C NMR

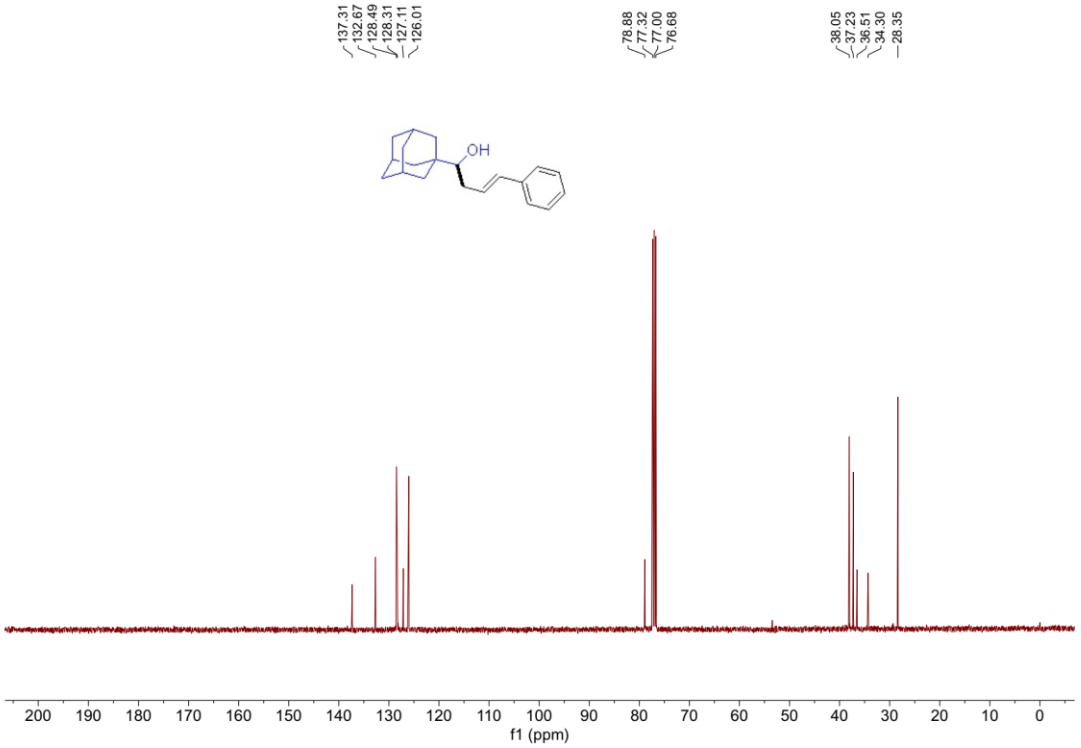

3cc ^{1}H NMR

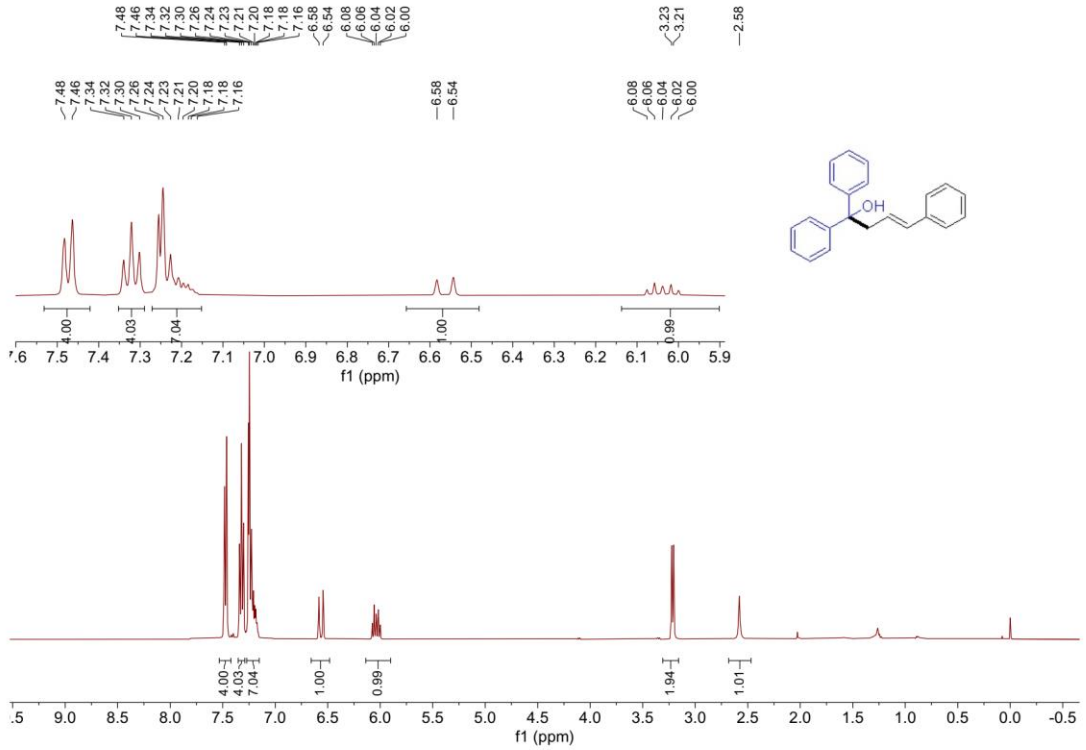

3cc ^{13}C NMR

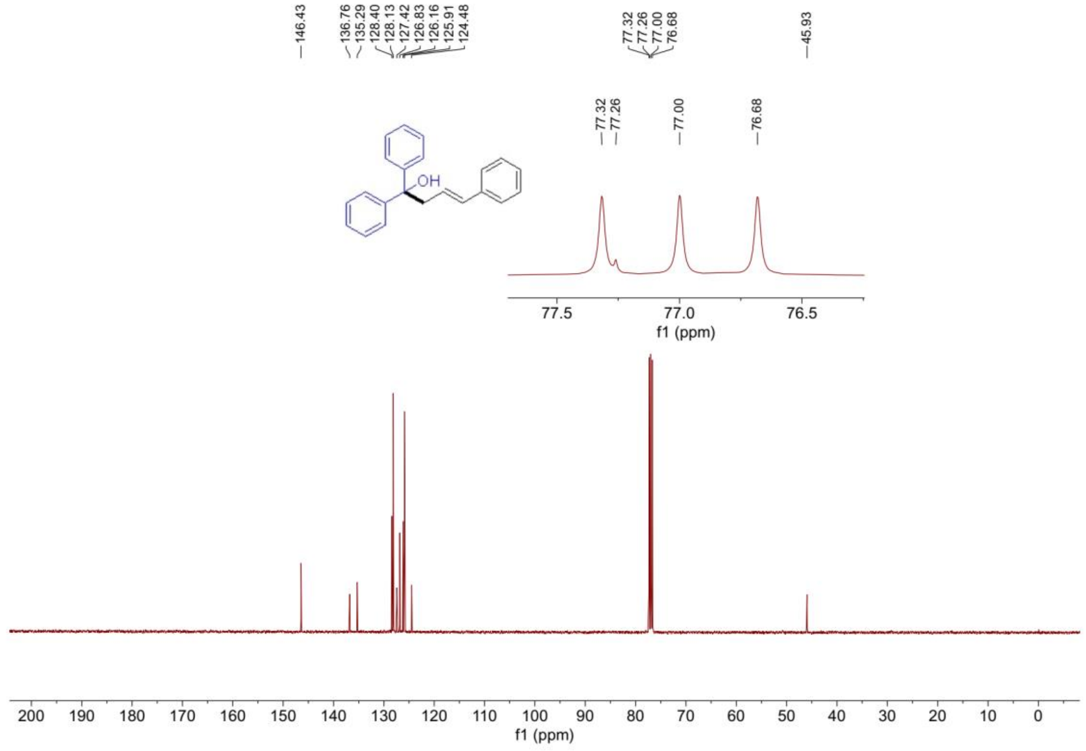

3cd ^{1}H NMR

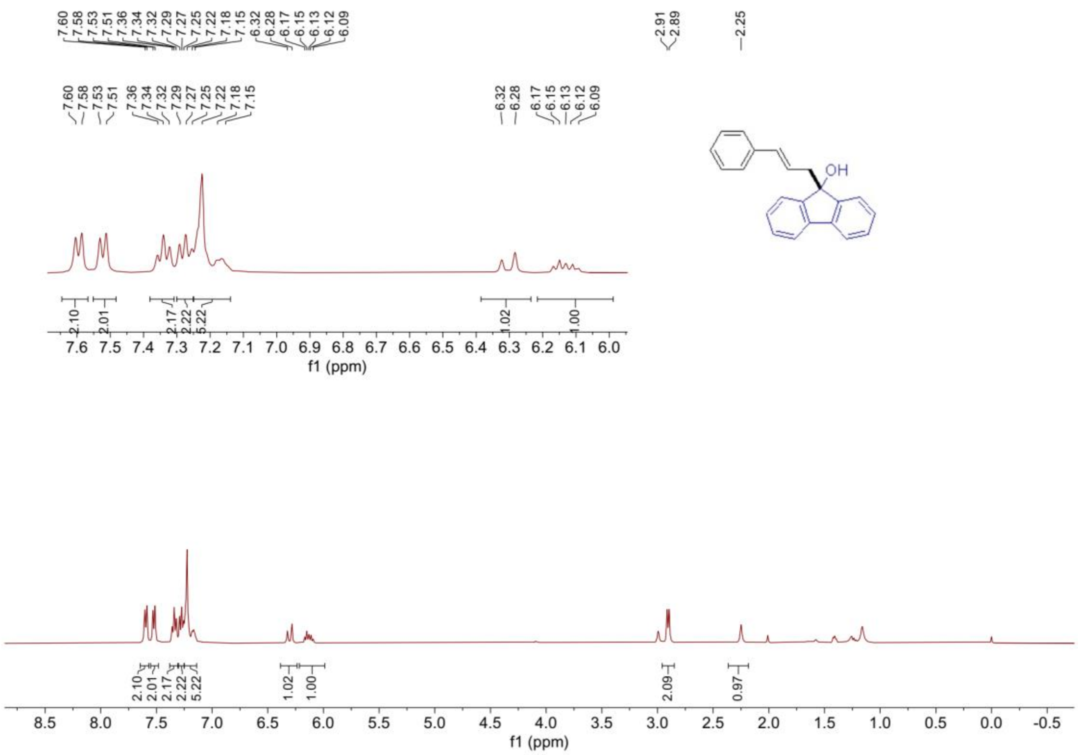

3cd ^{13}C NMR

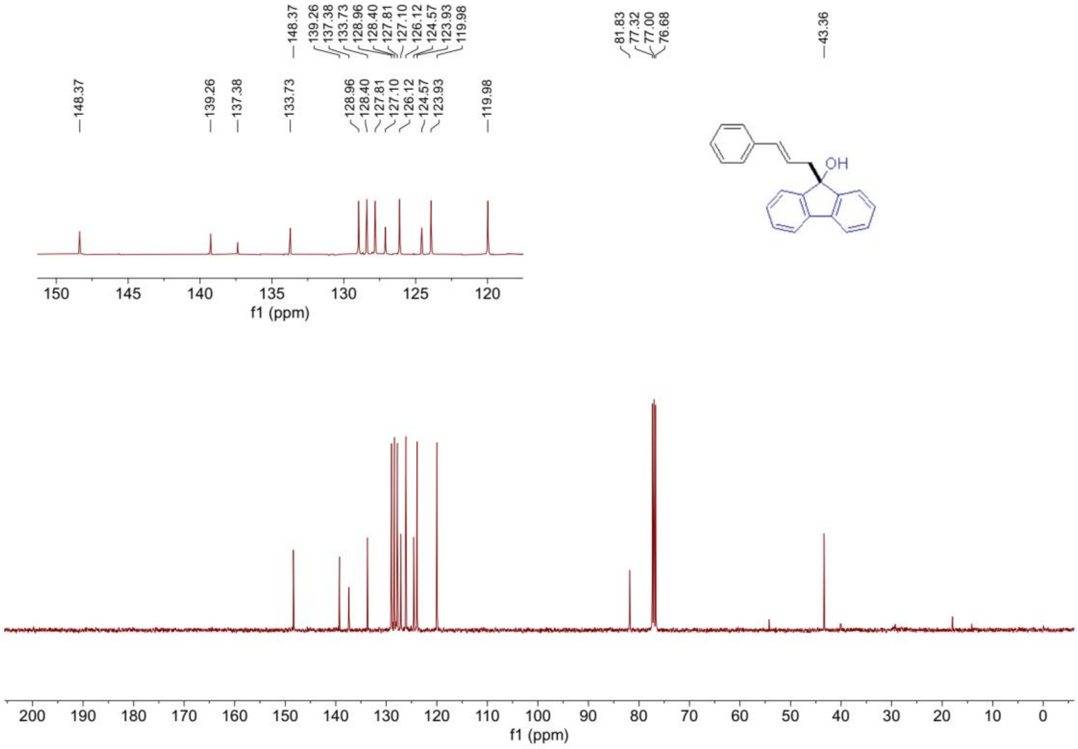

3ce ^{1}H NMR

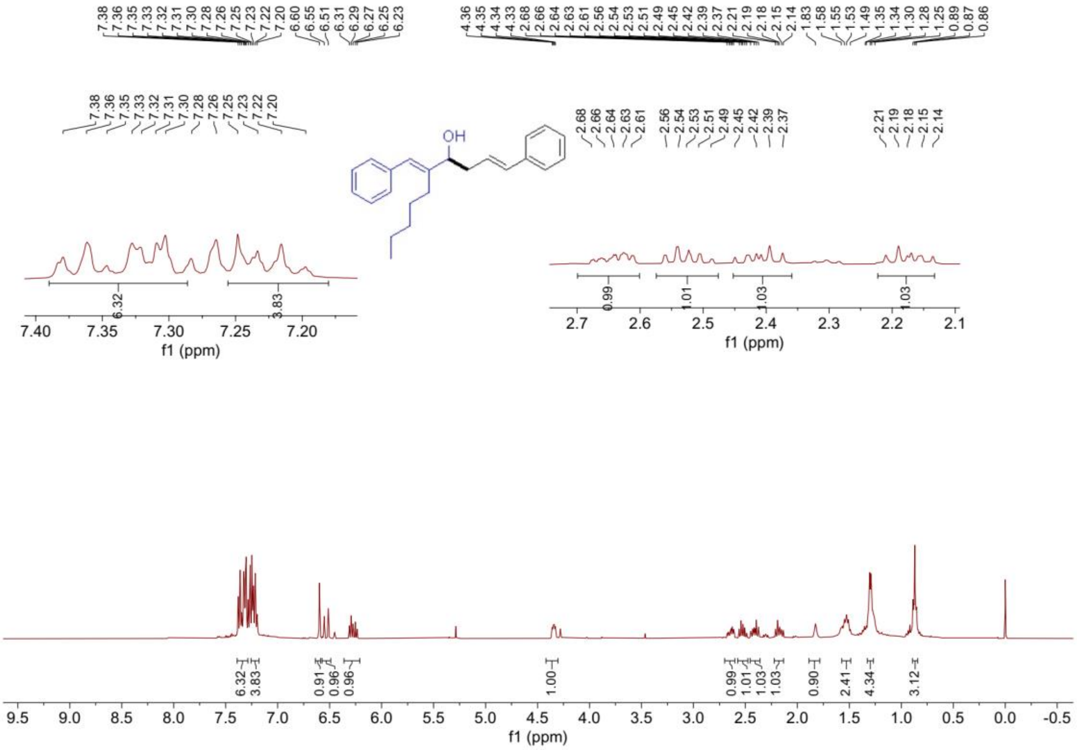

3ce ^{13}C NMR

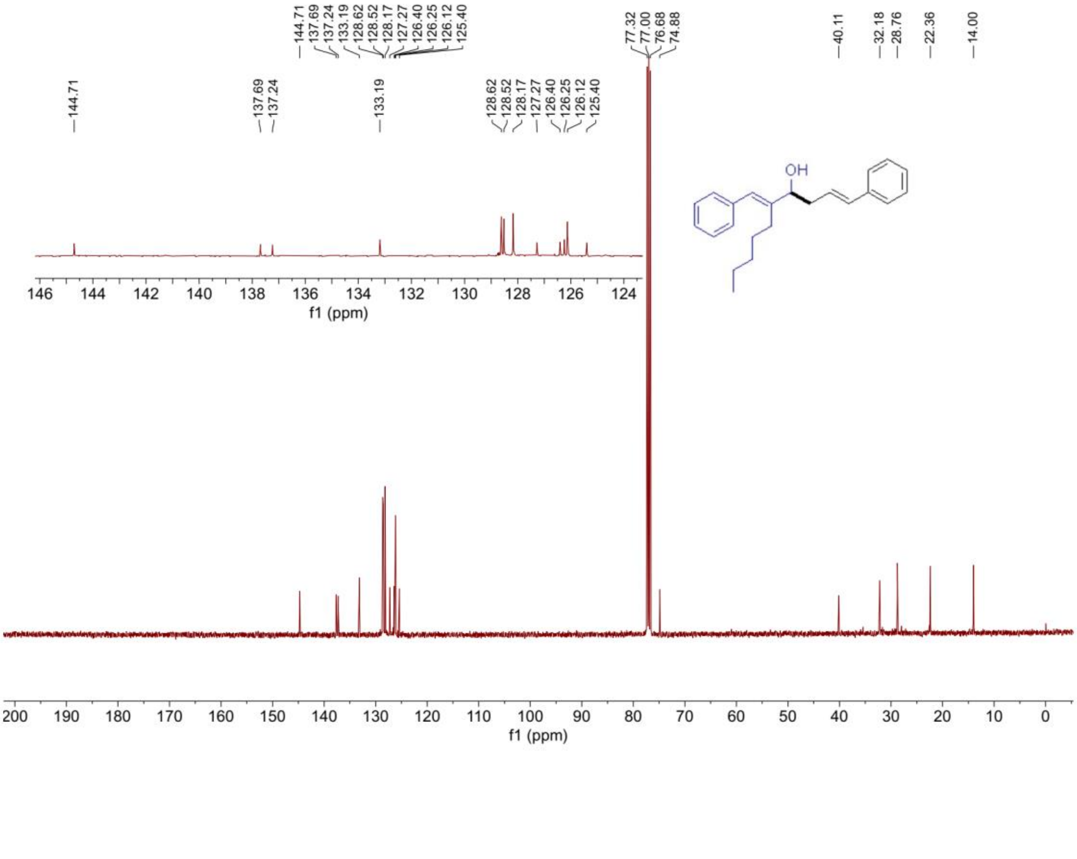

3cf ^{1}H NMR

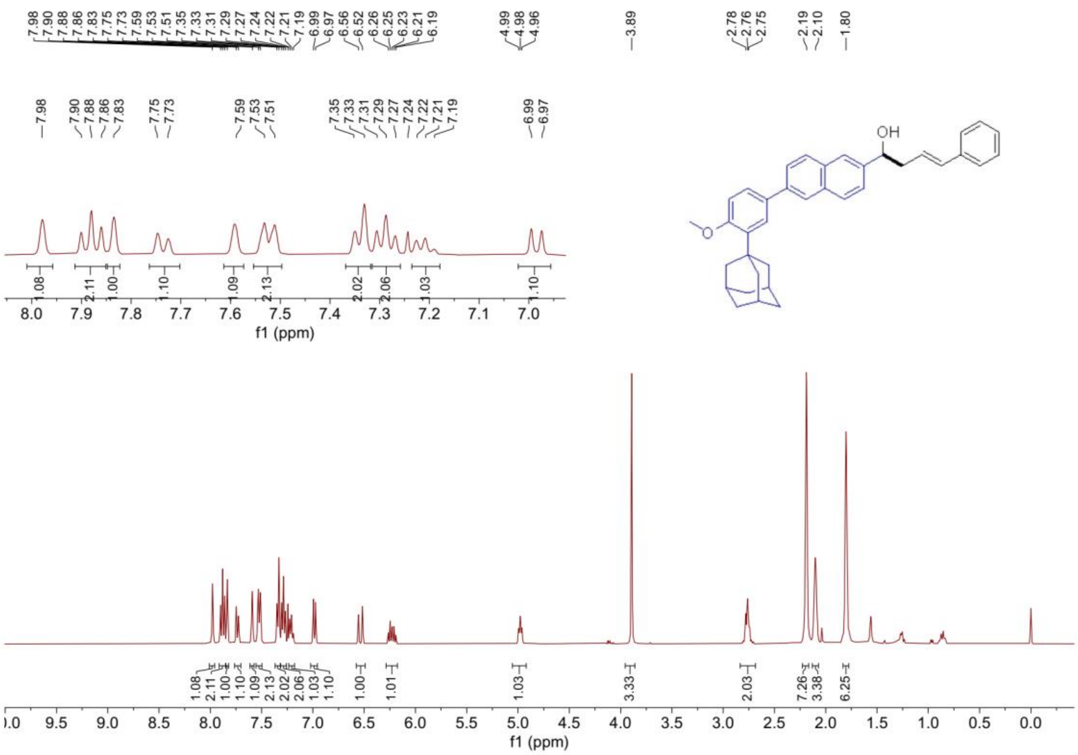

3cf ^{13}C NMR

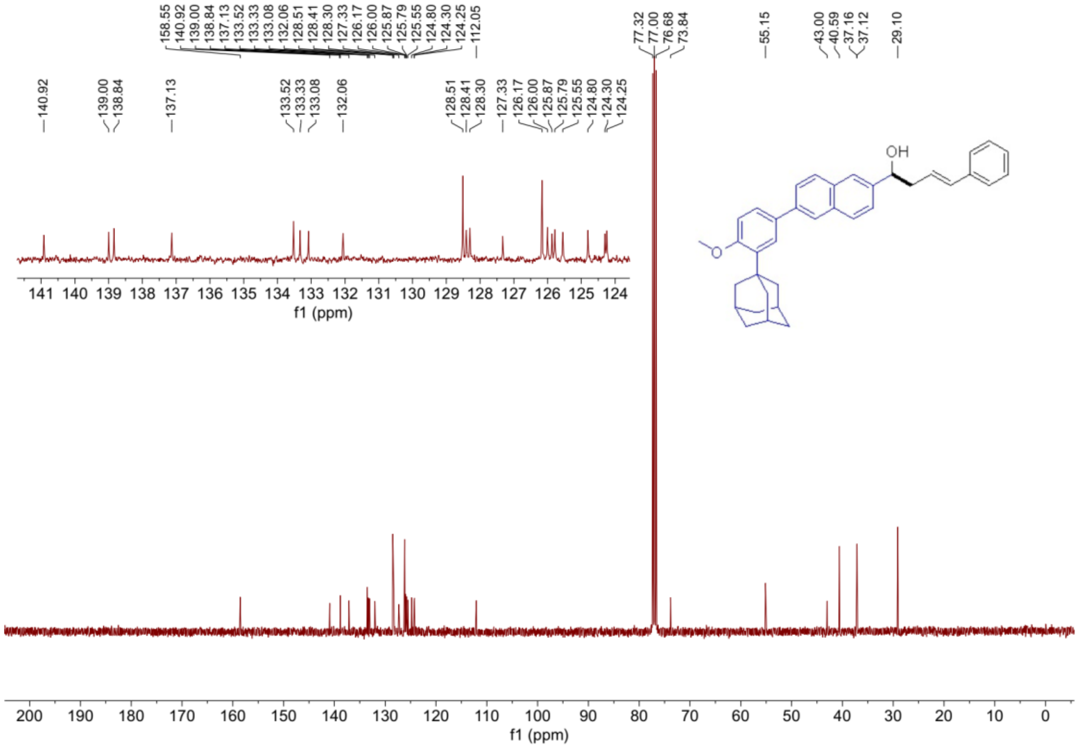

4 ^{1}H NMR

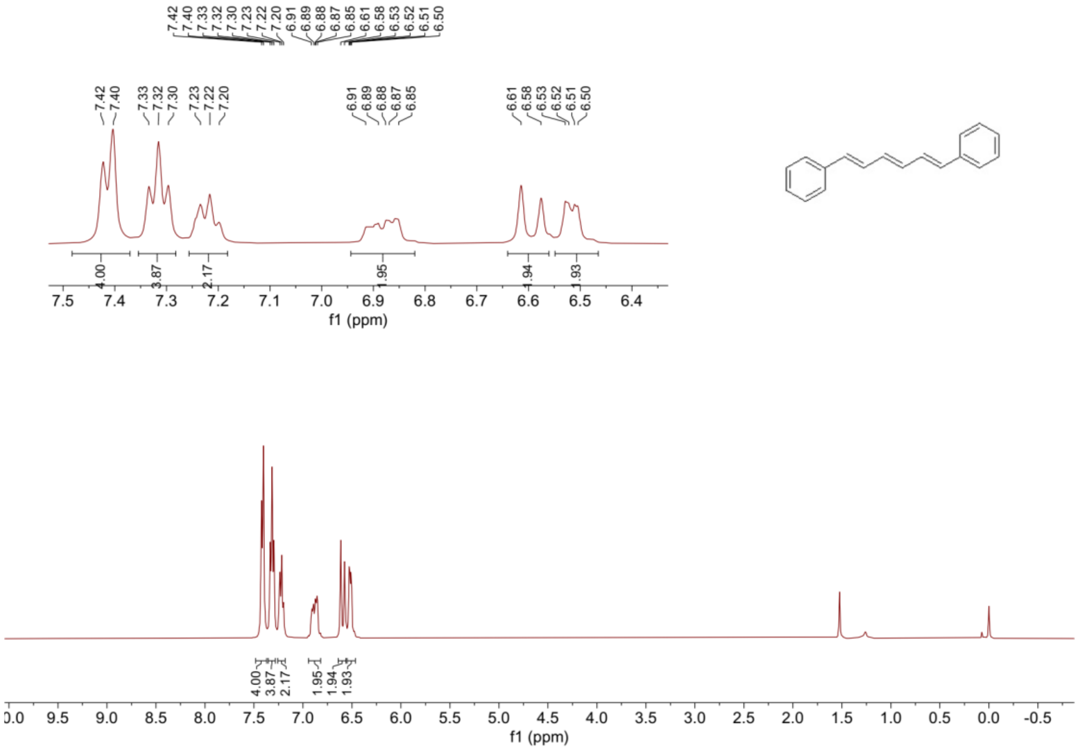

4 ^{13}C NMR

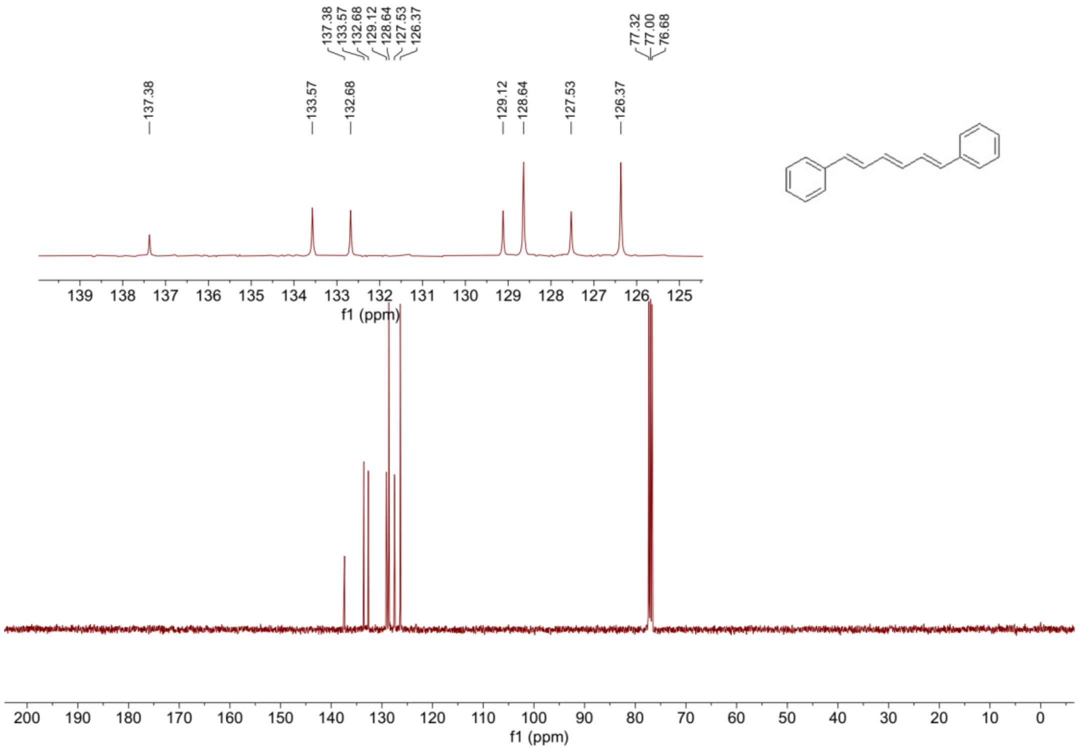

5 isomer I ^{1}H NMR

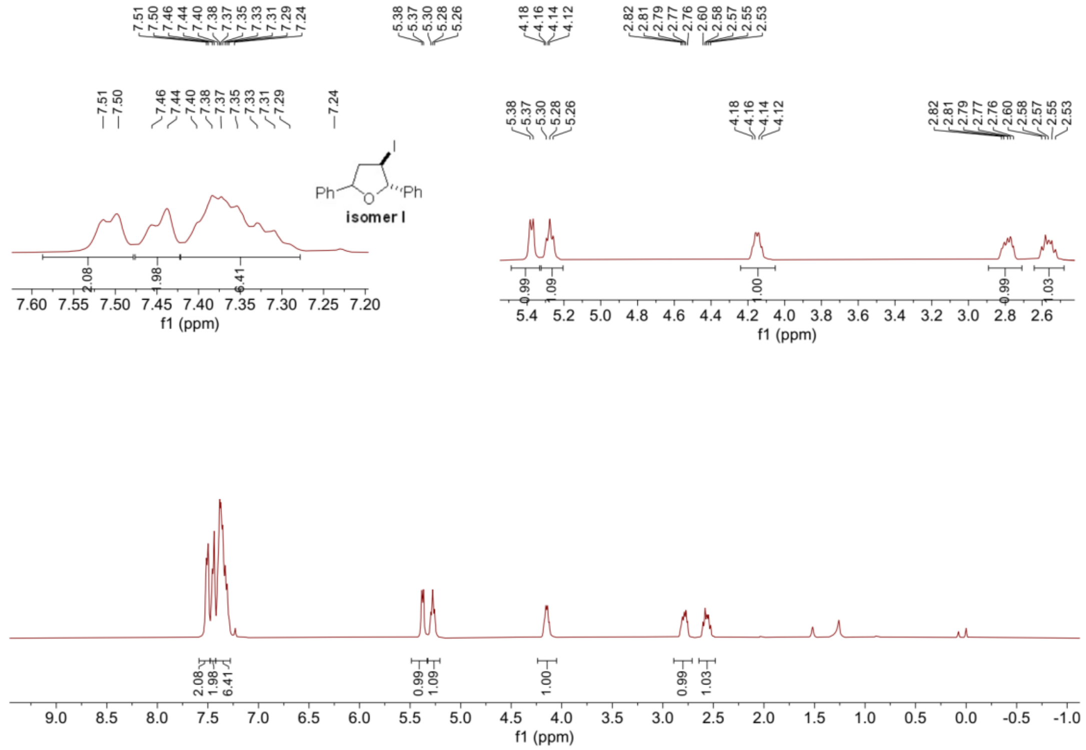

5 isomer I ^{13}C NMR

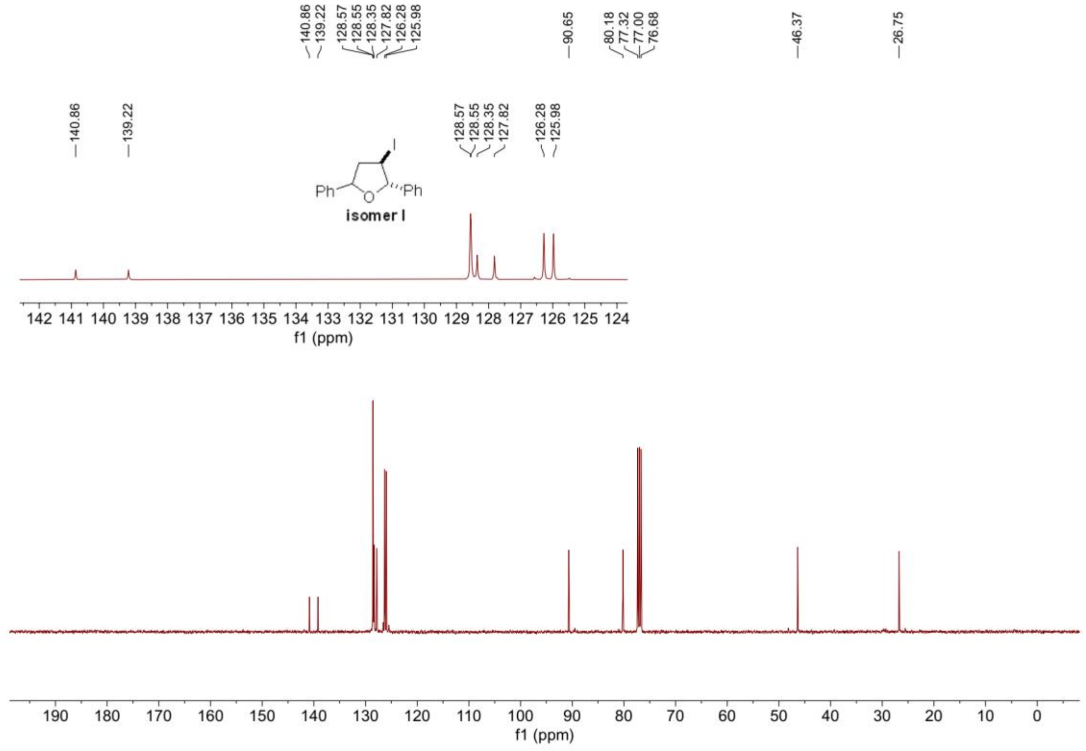

5 isomer II ^{1}H NMR

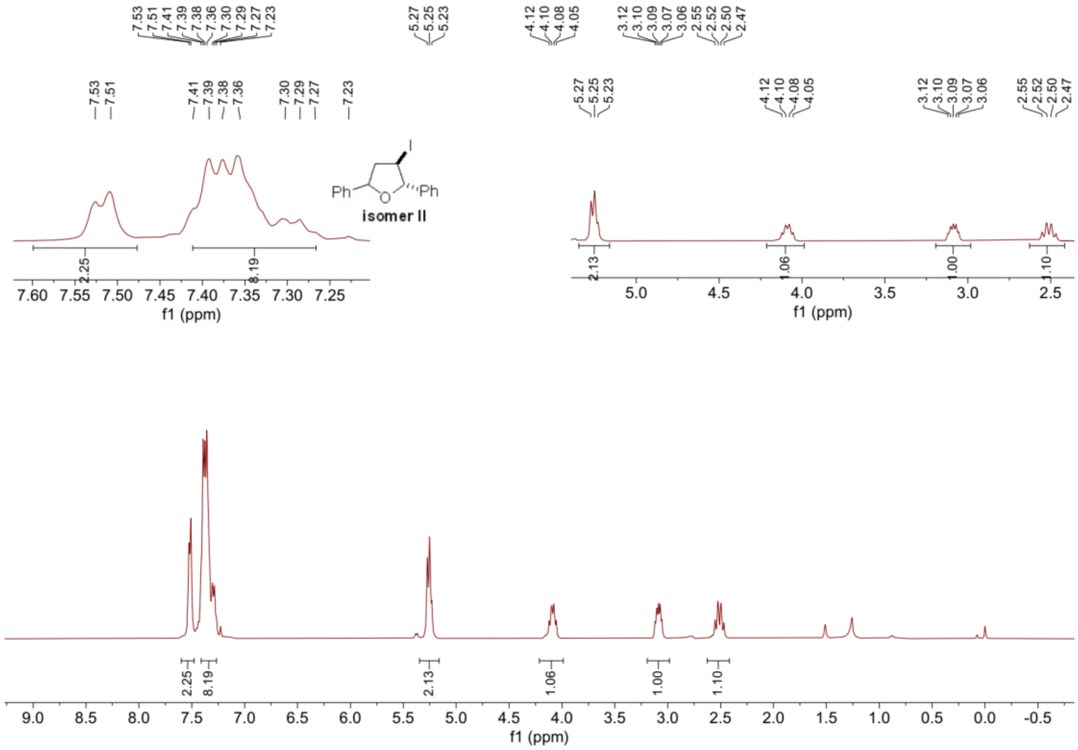

5 isomer II ^{13}C NMR

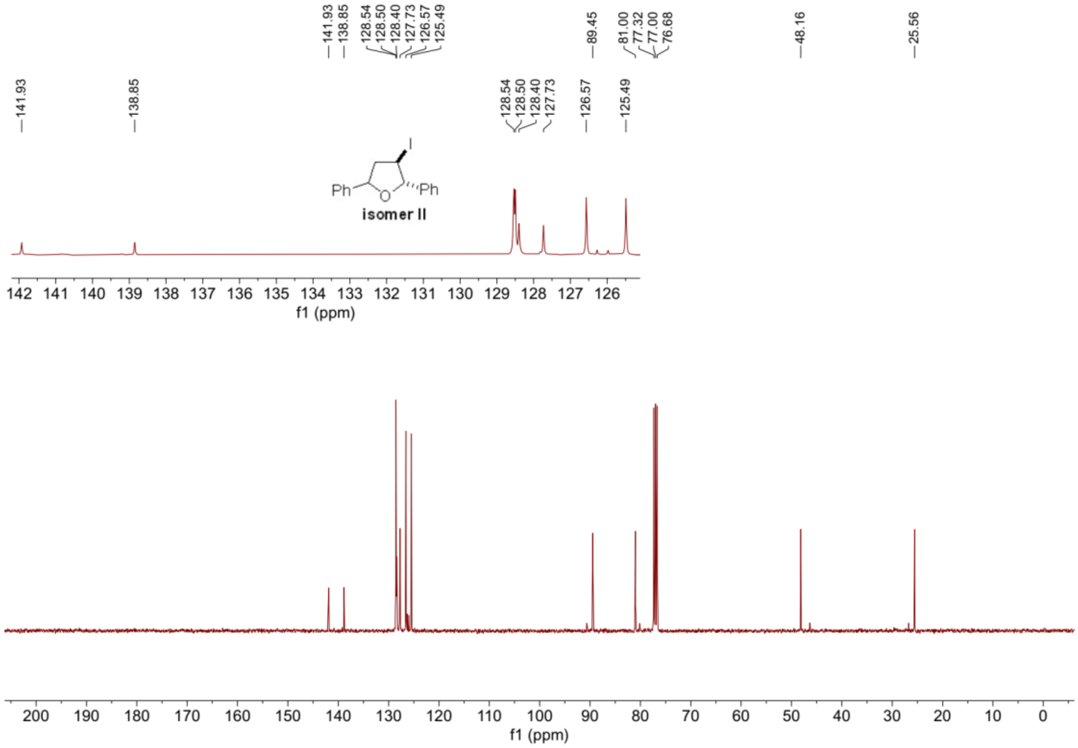

References

1. N. O. Thiel, B. Kaewmee, T. T. Ngoc, J. F. Teichert, *Chem. Eur. J.* **2020**, *26*, 1597–1603.

2. M. Hu, S. Ge, *Nat. Commun.* **2020**, *11*, 765.

3. Zhang, L. Li, J. Li, J. Shi, K. Xu, W. Gao, L. Zong, G. Li, M. Findlater, *Angew. Chem. Int. Ed.* **2021**, *60*, 7275−7282.

4. K.-Y. Ye, T. M[c]Callum, S. Lin, *J. Am. Chem. Soc.* **2019**, *141*, 9548−9554.

5. S. Zhang, Y. Liang, K. Liu, X. Zhan, W. Fan, M.-B. Li, M. Findlater, *J. Am. Chem. Soc.* **2023,** *145*, 14143–14154.

6. F. Lian, K. Xu, C. Zeng, *CCS Chem.* **2023**, *5*, 1973−1981.

7. A. Nagaki, Y. Tsuchihashi, S. Haraki, J. Yoshida, *Org. Biomol. Chem.* **2015**, *13*, 7140−7145.

8. H. Wang, Z. Wang, G. Zhao, V. Ramadoss, L. Tian, Y. Wang, *Org. Lett.* **2022**, *24*, 3668–3673.

9. S. L. MacNeil, O. B. Familoni, V. Snieckus, *J. Org. Chem.* **2001**, *66*, 3662−3670.

10. L. Li, N. Navasero, *Org. Lett.* **2004**, *6*, 3091-3094.

11. Y. Wang, J. Zhu, A. C. Durham, H. Lindberg, Y.-M. Wang, *J. Am. Chem. Soc.* **2019**, *141*, 19594−19599.

12. S. Banerjee, T. Kobayashi, K. Takai, S. Asako, L. Ilies, *Org. Lett.* **2022**, *24*, 7242−7246.
